# Supplementary material for: Saturated fatty acids induce lipotoxicity in lymphatic endothelial cells contributing to secondary lymphedema development
Source: EMBO Mol Med. 2025 Aug 4;17(9):2384–408. doi: 10.1038/s44321-025-00286-4 (PMC12423331; doi:10.1038/s44321-025-00286-4)
Supplement: Supplementary file 1 — Appendix [file 44321_2025_286_MOESM1_ESM.pdf]

Appendix

Saturated fatty acids induce lipotoxicity in lymphatic endothelial cells contributing to secondary lymphedema development - Gomes *et al.*

Table of Content

Appendix Table S1: Demographics of the study population - **page 3**

Appendix Table S2: Mice diet composition - **page 4**

Appendix Table S3: List of statistically significant P-values derived from the various statistical tests applied throughout this study **- page 5 to page 11**

Appendix Figure S1: Altered fatty acid composition in plasma lipid fractions of lymphedema patients compared to controls - **page 12**

Appendix Figure S2: Associations between plasma PUFA/SFA ratio and BMI in controls and lymphedema patients - **page 13**

Appendix Figure S3: PUFA/SFA ratio in plasma FFAs stratified by sex - **page 14**

Appendix Figure S4: Fatty acid composition in plasma lipid fractions of lymphedema patients and controls - **page 15**

Appendix Figure S5: Dietary interventions in the mouse tail lymphedema model - **page 16**

Appendix Figure S6: Effects of palmitic acid (PA) and stearic acid (SA) on endothelial cell viability - **page 17**

Appendix Figure S7: Stearic acid (SA) reduces colony-forming ability in endothelial cells - **page 18**

Appendix Figure S8: Lipid droplet formation in endothelial cells treated with stearic acid (SA) - **page 19**

Appendix Figure S9: Stearic acid (SA) and hydrogen peroxide (H_2O_2) induce apoptosis and reactive oxygen species (ROS) in endothelial cells - **page 20**

Appendix Figure S10: Effect of stearic acid (SA) on cytosolic reactive oxygen species (ROS) in Human Dermal Microvascular Endothelial Cells (HDMECs) - **page 21**

Appendix Figure S11: Stearic acid (SA) induces ER stress in lymphatic endothelial cells, which is attenuated by FABP4 inhibition - **page 22**

Appendix

Saturated fatty acids induce lipotoxicity in lymphatic endothelial cells contributing to secondary lymphedema development - Gomes *et al.*

Table of Content

Appendix Figure S12: Increased oxidative DNA damage in lymphatic endothelial cells of lymphedematous mice tissue under high-fat diet (HFD) - **page 23**

Appendix Figure S13: Modulation of apoptotic and anti-apoptotic proteins by stearic acid (SA) and oxidative stress in lymphatic endothelial cells - **page 24**

Appendix Figure S14: Effects of stearic acid (SA) and linoleic acid (LA) on lymphatic endothelial cell viability **- page 25**

Appendix Figure S15: Immunofluorescence analysis of oxidative stress, ER stress, and apoptosis markers in mouse tail tissue - **page 26**

Appendix Figure S16: Circulating levels of fatty acid-binding protein 4 (FABP4) in plasma samples from patients with lymphedema and non-lymphedema controls - **page 27**

Appendix Figure S17: Fatty acid-binding protein 4 (FABP4) expression and the impact of FABP4 inhibition on endothelial cell viability - **page 28**

Appendix Figure S18: Diet-dependent lipid accumulation in lymphedematous tissue - **page 29**

Appendix Figure S19: High saturated fat diet (HSFD) enhances perilymphatic FABP4 expression in a mouse model of lymphedema - **page 30**

Appendix Figure S20: FABP4 inhibition and dietary intervention reduce oxidative DNA damage in lymphedematous tissue under high saturated fat diet (HSFD) - **page 31**

Appendix Figure S21: Immunofluorescence analysis of oxidative stress, ER stress, and apoptosis markers in lymphedematous mice fed a chow diet (CD) - **page 32**

Appendix Figure S22: Protein levels detected by immunofluorescence staining in control mice 28 days post-sham surgery - **page 33**

Appendix Figure S23: Representative images of cell confluency and viability assessment - **page 34**

Appendix Table S1. Demographics of the study population.

	Value	Range
**Healthy controls**		
Sample size (*n*)	7	–
Female (*n*)	4	–
Male (*n*)	3	–
Age (years)	43 ± 6.27	32-63
BMI (kg/m^2)	26.7 ± 13.78	19.9-38.6
**Breast cancer controls**		
Sample size (*n*)	15	–
Female (*n*)	15	–
Male (*n*)	0	–
Age (years)	62.6 ± 3.30	37-88
BMI (kg/m^2)	27.5 ± 1.26	18.8-36.3
**Patients with secondary lymphedema**		
Sample size (*n*)	57	–
Female (*n*)	51	–
Male (*n*)	6	–
Age (years)	63 ± 13.34	32-88
BMI (kg/m^2)	33.6 ± 10.17	21.1-59.1
Breast cancer-related lymphedema (*n*)	38	–
Other cancers-related lymphedema (*n*)	14	–
Non-cancer-related lymphedema *(n)*	5	–
Upper extremity lymphedema (*n*)	41	–
Lower extremity lymphedema (*n*)	16	–

Abbreviation: BMI, body mass index. Lymphedema related to other cancers includes cases secondary to gynecological cancers, prostate cancer, melanoma, bladder cancer, and B-cell lymphoma.

Appendix Table S2. Mice diet composition.

	Chow Diet (CD)	High Fat Diet (HFD)	High Saturated Fat Diet (HSFD)
**Protein (% energy)**	24	20	20
**Carbohydrate (% energy)**	63	20	20
**Fat (% energy)**	13	60	60
**Saturated fat (% of total fat)**	15	32.2	59.2
**Monounsaturated fat (% of total fat)**	17.5	35.9	32.1
**Polyunsaturated fat (% of total fat)**	52.5	31.9	8.7
**Unknown (% of total fat)**	15	-	-
**UFA/SFA**	4.6	2.1	0.68
**PUFA/SFA**	3.5	0.99	0.14
**Palmitic acid (PA) (% of total fat)**	12.5	19.6	24.1
**Stearic acid (SA) (% of total fat)**	2.5	10.6	33.3
**Oleic acid (AO) (% of total fat)**	17.5	33.9	31.8
**Linoleic acid (LA) (% of total fat)**	50	28.6	7.8

Note: The composition of the chow diet was obtained from the manufacturer's 2023 data sheet for 5053 PicoLab Rodent Diet 20 (LabDiet). The compositions of the high fat and high saturated fat diets were obtained from manufacturer's 2023 data sheets for D12492 and D12113001, respectively (Research Diets Inc).

Appendix Table S3. List of statistically significant P-values derived from the various statistical tests applied throughout this study.

Figure Panel and Comparison	Statistical test and p-value	Figure Panel and Comparison	Statistical test and p-value
Fig. 1A	Two-tailed unpaired t-test	LE CD vs. LE HSFD	<0.0001
Control vs. Lymphedema	0.0004	Fig. 2F	One-way ANOVA with Tukey's post hoc
Fig. 1B	Two-tailed unpaired t-test	Sham HSFD vs. LE HSFD	<0.0001
Control vs. Lymphedema	0.0012	LE CD vs. LE HSFD	<0.0001
Fig. 1C	Two-tailed unpaired t-test	Fig. 2G	One-way ANOVA with Tukey's post hoc
Control vs. Lymphedema	0.0046	Sham HSFD vs. LE HSFD	<0.0001
Fig. 1D	Two-tailed unpaired t-test	LE CD vs. LE HSFD	<0.0001
Control vs. Lymphedema	0.2165	Fig. 2H	One-way ANOVA with Tukey's post hoc
Fig. 2C (4 days)	Two-way ANOVA with Šídák's post hoc	Sham HSFD vs. LE HSFD	<0.0001
LE HSFD vs. LE CD	0.0465	LE CD vs. LE HSFD	0.0003
Fig. 2C (6 days)	Two-way ANOVA with Šídák's post hoc	Fig. 2I	One-way ANOVA with Tukey's post hoc
LE HSFD vs. LE CD	0.0100	Sham HSFD vs. LE HSFD	0.0014
Fig. 2C (8 days)	Two-way ANOVA with Šídák's post hoc	LE CD vs. LE HSFD	0.0001
LE CD vs. LE HSFD	0.0049	Fig. 2J	One-way ANOVA with Tukey's post hoc
LE HFD vs. LE CD	0.0125	Sham CD vs. LE CD	0.0318
Fig. 2C (10 days)	Two-way ANOVA with Šídák's post hoc	Sham HSFD vs. LE HSFD	<0.0001
LE HSFD vs. LE CD	0.0478	LE CD vs. LE HSFD	0.0041
Fig. 2C (24 days)	Two-way ANOVA with Šídák's post hoc	Fig. 3A	One-way ANOVA with Tukey's post hoc
LE HSFD vs. LE CD	0.0245	Vehicle vs. SA 10μM	0.0004
Fig. 2C (26 days)	Two-way ANOVA with Šídák's post hoc	Vehicle vs. SA 50μM	<0.0001
LE CD vs. LE HSFD	0.0083	Vehicle vs. SA 100μM	<0.0001
LE HFD vs. LE HSFD	0.0128	Fig. 3B	One-way ANOVA with Tukey's post hoc
Fig. 2C (28 days)	Two-way ANOVA with Šídák's post hoc	SA 10μM: HUVECs vs. HDLECs	<0.0001
LE CD vs. LE HSFD	0.0033	SA 10μM: HDMECs vs. HDLECs	<0.0001
LE HFD vs. LE HSFD	0.0047	PA 250μM: HUVECs vs. HDLECs	0.0063
Fig. 2E	One-way ANOVA with Tukey's post hoc	PA 250μM: HDMECs vs. HDLECs	0.0007
Sham HSFD vs. LE HSFD	<0.0001		

Figure Panel and Comparison	Statistical test and p-value
Fig. 3C	One-way ANOVA with Tukey's post hoc
Vehicle vs. SA 10μM	0.0284
Fig. 3D	One-way ANOVA with Tukey's post hoc
Vehicle vs. SA 10μM	<0.0001
SA 1μM vs. SA 10μM	0.0011
Fig. 3E	One-way ANOVA with Tukey's post hoc
Vehicle vs. SA 10μM	0.0026
Fig. 3F	One-way ANOVA with Tukey's post hoc
Vehicle vs. SA	0.0177
α-Tocopherol vs. SA	0.0137
Fig. 4B	One-way ANOVA with Tukey's post hoc
Sham HSFD vs. LE HSFD	0.0002
LE CD vs. LE HSFD	0.0002
Fig. 4C	One-way ANOVA with Tukey's post hoc
Vehicle vs. SA 10μM	0.0284
Vehicle + H_2O_2 vs. SA 10μM + H_2O_2	0.0019
SA 1μM + H_2O_2 vs. SA 10μM + H_2O_2	0.0476
Fig. 4D	One-way ANOVA with Tukey's post hoc
Vehicle vs. SA 10μM	0.0006
SA 1μM vs. SA 10μM	0.0067
Vehicle + H_2O_2 vs. SA 10μM + H_2O_2	0.0119
SA 1μM + H_2O_2 vs. SA 10μM + H_2O_2	0.0405
Fig. 4E	One-way ANOVA with Tukey's post hoc
Vehicle vs. SA 10μM	0.0016
Vehicle + H_2O_2 vs. SA 1μM + H_2O_2	0.0027
Vehicle + H_2O_2 vs. SA 1μM + H_2O_2	<0.0001
Fig. 5A (6 days)	Two-way ANOVA with Šídák's post hoc
LE CD vs. LE HSFD	0.0430
Fig. 5A (8 days)	Two-way ANOVA with Šídák's post hoc
LE CD vs. LE HSFD	0.0168
Fig. 5A (24 days)	Two-way ANOVA with Šídák's post hoc
LE CD vs. LE HSFD	0.0278
LE HSFD vs. LE HSFD→CD	0.0493
Fig. 5A (26 days)	Two-way ANOVA with Šídák's post hoc
LE CD vs. LE HSFD	0.0085
LE HSFD vs. LE HSFD→CD	0.0358
Fig. 5A (28 days)	Two-way ANOVA with Šídák's post hoc
LE CD vs. LE HSFD	0.0009
LE HSFD vs. LE HSFD→CD	0.0098
Fig. 5B	One-way ANOVA with Tukey's post hoc
LE CD vs. LE HSFD	0.0061
LE HSFD vs. LE HSFD→CD	0.0232
Fig. 5D	One-way ANOVA with Tukey's post hoc
LE CD vs. LE HSFD	0.0012
LE HSFD vs. LE HSFD→CD	0.0124
Fig. 5E	One-way ANOVA with Tukey's post hoc
LE CD vs. LE HSFD	0.0005
LE HSFD vs. LE HSFD→CD	0.0008
Fig. 5F	One-way ANOVA with Tukey's post hoc
LE CD vs. LE HSFD	0.0001
LE HSFD vs. LE HSFD→CD	0.0027
Fig. 5G	One-way ANOVA with Tukey's post hoc
LE CD vs. LE HSFD	0.0006
LE HSFD vs. LE HSFD→CD	0.0039

Figure Panel and Comparison	Statistical test and p-value
Fig. 5H	One-way ANOVA with Tukey's post hoc
LE CD vs. LE HSFD	<0.0001
LE HSFD vs. LE HSFD→CD	0.0005
Fig. 5I	One-way ANOVA with Tukey's post hoc
LE CD vs. LE HSFD	0.0447
LE HSFD vs. LE HSFD→CD	0.0446
Fig. 6A	Two-tailed unpaired t-test
Control vs. Lymphedema	<0.0001
Fig. 6B	One-way ANOVA with Tukey's post hoc
Vehicle vs. SA 50µM	<0.0001
SA 50µM vs. SA 50µM + BMS 5µM	0.0012
Vehicle vs. PA 250µM	<0.0001
PA 250µM vs. PA 250µM + BMS 5µM	0.0088
Fig. 6F	One-way ANOVA with Tukey's post hoc
Sc-siRNA Vehicle vs. Sc-siRNA SA 10µM	<0.0001
siFABP4 Vehicle vs. siFABP4 SA 10µM	0.0006
Sc-siRNA SA 10µM vs. siFABP4 SA 10µM	<0.0001
Fig. 7A (4 days)	Two-way ANOVA with Šídák's post hoc
LE CD + PBS vs. LE HSFD + PBS	0.0308
Fig. 7A (6 days)	Two-way ANOVA with Šídák's post hoc
LE CD + PBS vs. LE HSFD + PBS	0.0213
Fig. 7A (8 days)	Two-way ANOVA with Šídák's post hoc
LE CD + PBS vs. LE HSFD + PBS	0.0049
Fig. 7A (24 days)	Two-way ANOVA with Šídák's post hoc
LE CD + PBS vs. LE HSFD + PBS	0.0319
Fig. 7A (26 days)	Two-way ANOVA with Šídák's post hoc
LE CD + PBS vs. LE HSFD + PBS	0.0066

Figure Panel and Comparison	Statistical test and p-value
Fig. 7A (28 days)	Two-way ANOVA with Šídák's post hoc
LE CD + PBS vs. LE HSFD + PBS	0.0007
Fig. 7B	One-way ANOVA with Tukey's post hoc
LE CD + PBS vs. LE HSFD + PBS	0.0009
LE CD + BMS vs. LE HSFD + PBS	0.0020
LE HSFD + PBS vs. LE HSFD + BMS	0.0250
Fig. 7D	One-way ANOVA with Tukey's post hoc
LE CD + PBS vs. LE HSFD + PBS	0.0012
LE CD + PBS vs. LE HSFD + BMS	0.0439
Fig. 7E	One-way ANOVA with Tukey's post hoc
LE CD + PBS vs. LE HSFD + PBS	0.0032
LE HSFD + PBS vs. LE HSFD + BMS	0.0260
Fig. 7F	One-way ANOVA with Tukey's post hoc
LE CD + PBS vs. LE HSFD + PBS	<0.0001
LE HSFD + PBS vs. LE HSFD + BMS	0.0125
LE CD + PBS vs. LE HSFD + BMS	0.0270
Fig. 7G	One-way ANOVA with Tukey's post hoc
LE CD + PBS vs. LE HSFD + PBS	<0.0001
LE HSFD + PBS vs. LE HSFD + BMS	0.0011
LE CD + PBS vs. LE HSFD + BMS	0.0260
Fig. 7H	One-way ANOVA with Tukey's post hoc
LE CD + PBS vs. LE HSFD + PBS	0.0004
LE HSFD + PBS vs. LE HSFD + BMS	0.0383
Fig. 7I	One-way ANOVA with Tukey's post hoc
LE CD + PBS vs. LE HSFD + PBS	0.0439
Fig. EV1A	Two-way ANOVA with Šídák's post hoc
SFA Control vs. SFA Lymphedema	<0.0001

Figure Panel and Comparison	Statistical test and p-value	Figure Panel and Comparison	Statistical test and p-value
Fig. EV1B	Two-way ANOVA with Šídák's post hoc	Fig. EV2D	Two-tailed unpaired t-test
SFA Control vs. SFA Lymphedema	0.0021	Sham vs. Lymphedema	0.0041
PUFA Control vs. PUFA Lymphedema	0.0304	Fig. EV2G	Two-tailed unpaired t-test
Fig. EV1C	Two-way ANOVA with Šídák's post hoc	Sham vs. Lymphedema	0.0037
PUFA Control vs. PUFA Lymphedema	0.0008	Fig. EV3B	One-way ANOVA with Tukey's post hoc
Fig. EV1D	Two-way ANOVA with Šídák's post hoc	0h – Vehicle vs. SA 10μM	0.2129
MUFA Control vs. MUFA Lymphedema	<0.0001	16h – Vehicle vs. SA 10μM	0.0018
Fig. EV1E	Two-way ANOVA with Šídák's post hoc	32h – Vehicle vs. SA 10μM	0.0004
16:0 Control vs. 16:0 Lymphedema	0.0002	Fig. EV3D	One-way ANOVA with Tukey's post hoc
18:2n6 Control vs. 18:2n6 Lymphedema	<0.0001	0h – Vehicle vs. SA 10μM	0.0810
Fig. EV1F	Two-way ANOVA with Šídák's post hoc	16h – Vehicle vs. SA 10μM	0.5622
18:2n6 Control vs. 18:2n6 Lymphedema	<0.0001	32h – Vehicle vs. SA 10μM	0.5506
Fig. EV1G	Two-way ANOVA with Šídák's post hoc	Fig. EV3B	One-way ANOVA with Tukey's post hoc
18:2n6 Control vs. 18:2n6 Lymphedema	<0.0001	Sham HSFD vs. LE HSFD	0.0003
Fig. EV1H	Two-way ANOVA with Šídák's post hoc	LE HSFD vs. LE HSFD + BMS	0.0004
18:2n6 Control vs. 18:2n6 Lymphedema	0.0105	LE HSFD vs. LE HSFD→CD	<0.0001
Fig. EV2A	One-way ANOVA with Tukey's post hoc	Fig. EV3D	One-way ANOVA with Tukey's post hoc
CD vs. HSFD	0.0194	LE CD vs. LE HSFD	<0.0001
Fig. EV2B	Two-way ANOVA with Šídák's post hoc	LE CD vs. LE HSFD + BMS	0.0062
18:0 CD vs. 18:0 HFD	0.0011	LE CD vs. LE HSFD→CD	0.0075
18:0 CD vs. 18:0 HSFD	0.0065	Fig. EV3E	One-way ANOVA with Tukey's post hoc
18:1n9 CD vs. 18:1n9 HSFD	<0.0001	LE CD vs. LE HSFD	<0.0001
18:1n9 HFD vs. 18:1n9 HSFD	0.0014	LE HSFD vs. LE HSFD + BMS	<0.0001
18:2n6 CD vs. 18:2n6 HSFD	<0.0001	LE HSFD vs. LE HSFD→CD	<0.0001
18:2n6 HFD vs. 18:2n6 HSFD	<0.0001	Fig. EV3F	One-way ANOVA with Tukey's post hoc
18:2n6 CD vs. 18:2n6 HFD	0.0364	LE CD vs. LE HSFD	0.0127
22:6n3 CD vs. 18:2n6 HFD	0.0123		

Figure Panel and Comparison	Statistical test and p-value
Fig. EV5C	One-way ANOVA with Tukey's post hoc
LE CD vs. LE HSFD	0.0002
LE HSFD vs. LE HSFD + BMS	0.0059
LE HSFD vs. LE HSFD→CD	0.0067
Fig. EV5D	One-way ANOVA with Tukey's post hoc
LE CD vs. LE HSFD	0.0324
Appendix Fig. S1A	One-way ANOVA with Tukey's post hoc
BC CT vs. BCL	0.0004
BC CT vs. Non-BCL	<0.0001
Appendix Fig. S1B	One-way ANOVA with Tukey's post hoc
BC CT vs. BCL	0.0168
Appendix Fig. S1C	One-way ANOVA with Tukey's post hoc
Healthy CT vs. BCL	0.0334
Appendix Fig. S1D	One-way ANOVA with Tukey's post hoc
Healthy CT vs. BC CT	0.0020
Healthy CT vs. BCL	0.0002
Appendix Fig. S2A	Two-way ANOVA with Tukey's post hoc
<24.9 – Controls vs. Lymphedema	0.0343
>30 – Controls vs. Lymphedema	0.0067
Appendix Fig. S2B	Two-way ANOVA with Tukey's post hoc
<24.9 – Control vs. Lymphedema	0.0006
25-29.9 – Control vs. Lymphedema	0.0305
Appendix Fig. S3A	Two-way ANOVA with Tukey's post hoc
Female – Control vs. Lymphedema	0.0004
Appendix Fig. S3B	Two-way ANOVA with Tukey's post hoc
Female – Control vs. Lymphedema	0.0005
Appendix Fig. S3C	Two-way ANOVA with Tukey's post hoc
Female – Control vs. Lymphedema	0.0462
Male – Control vs. Lymphedema	0.0382
Appendix Fig. S4A	Two-way ANOVA with Šídák's post hoc
16:0 – BC CT vs. BCL	0.0033
16:0 – BC CT vs. Non-BCL	0.0012
18:2n6 – BC CT vs. Non-BCL	0.0028
Appendix Fig. S4B	Two-way ANOVA with Šídák's post hoc
18:2n6 – BC CT vs. BCL	0.0010
Appendix Fig. S4C	Two-way ANOVA with Šídák's post hoc
18:2n6 – CT vs. BCL	<0.0001
18:2n6 – CT vs. BCL	<0.0001
18:2n6 – BC CT vs. Non-BCL	0.0143
18:2n6 – BC CT vs. Non-BCL	<0.0001
Appendix Fig. S4D	Two-way ANOVA with Šídák's post hoc
16:0 – BC CT vs. BCL	0.0263
18:2n6 – BC CT vs. BCL	0.0270
Appendix Fig. S6A	One-way ANOVA with Tukey's post hoc
Vehicle vs. PA 250μM	0.0042
Vehicle vs. PA 500μM	<0.0001
Appendix Fig. S6B	One-way ANOVA with Tukey's post hoc
Vehicle vs. PA 250μM	0.0007
Vehicle vs. PA 500μM	<0.0001
Appendix Fig. S6C	One-way ANOVA with Tukey's post hoc
Vehicle vs. PA 50μM	0.0004
Vehicle vs. PA 100μM	<0.0001
Vehicle vs. PA 250μM	<0.0001
Vehicle vs. PA 500μM	<0.0001

Figure Panel and Comparison	Statistical test and p-value
Appendix Fig. S6D	One-way ANOVA with Tukey's post hoc
Vehicle vs. SA 50µM	0.0025
Vehicle vs. SA 100µM	0.0004
Appendix Fig. S6E	One-way ANOVA with Tukey's post hoc
Vehicle vs. SA 50µM	0.0022
Vehicle vs. SA 100µM	0.0001
Appendix Fig. S6F	One-way ANOVA with Tukey's post hoc
Vehicle vs. SA 50µM	0.0006
Vehicle vs. SA 100µM	<0.0001
Appendix Fig. S7B	One-way ANOVA with Tukey's post hoc
Vehicle vs. SA 100nM	0.0007
Vehicle vs. SA 500nM	<0.0001
SA 100nM vs. SA 500nM	0.0017
Appendix Fig. S7F	One-way ANOVA with Tukey's post hoc
Vehicle vs. SA 100nM	0.0047
Vehicle vs. SA 500nM	<0.0001
SA 100nM vs. SA 500nM	<0.0001
Appendix Fig. S8C	One-way ANOVA with Tukey's post hoc
HDLECs - Vehicle vs. SA 10µM	0.0022
HUVECs - Vehicle vs. SA 10µM	0.0015
Appendix Fig. S11B	One-way ANOVA with Tukey's post hoc
Vehicle vs. SA 10µM	0.0006
SA 10µM vs. BMS 5µM + SA 10µM	0.0004
Appendix Fig. S11C	One-way ANOVA with Tukey's post hoc
Vehicle vs. SA 10µM	<0.0001
Vehicle vs. SA 20µM	<0.0001
Vehicle vs. SA 30µM	<0.0001

Figure Panel and Comparison	Statistical test and p-value
Vehicle vs. SA 50µM	<0.0001
SA 10µM vs. BMS 5µM + SA 10µM	<0.0001
SA 20µM vs. BMS 5µM + SA 20µM	<0.0001
SA 30µM vs. BMS 5µM + SA 30µM	<0.0001
SA 50µM vs. BMS 5µM + SA 50µM	<0.0001
Appendix Fig. S12B	Two-tailed unpaired t-test
Sham vs. Lymphedema	0.0100
Appendix Fig. S13B	One-way ANOVA with Tukey's post hoc
Vehicle + H_2O_2 vs. SA 10µM + H_2O_2	0.0109
Appendix Fig. S13C	One-way ANOVA with Tukey's post hoc
Vehicle + H_2O_2 vs. SA 10µM + H_2O_2	0.0062
Appendix Fig. S13D	One-way ANOVA with Tukey's post hoc
Vehicle + H_2O_2 vs. SA 10µM + H_2O_2	0.0038
Appendix Fig. S13E	One-way ANOVA with Tukey's post hoc
Vehicle + H_2O_2 vs. SA 10µM + H_2O_2	0.0009
Appendix Fig. S14	One-way ANOVA with Tukey's post hoc
Vehicle vs. SA 50µM	<0.0001
LA 50µM vs. SA 50µM	<0.0001
SA 50µM vs. LA + SA	<0.0001
Appendix Fig. S16	One-way ANOVA with Tukey's post hoc
Healthy CT vs. Non-BCL	0.0127
BC CT vs. BCL	<0.0001
BC CT vs. Non-BCL	<0.0001
Appendix Fig. S17B	One-way ANOVA with Tukey's post hoc
Vehicle vs. BMS 10µM	<0.0001
Vehicle vs. BMS 20µM	<0.0001
Vehicle vs. BMS 50µM	<0.0001

Figure Panel and Comparison	Statistical test and p-value
Appendix Fig. S18B	Two-way ANOVA with Tukey's post hoc
CD - Sham vs. Lymphedema	0.0002
HFD - Sham vs. Lymphedema	0.0001
HSFD - Sham vs. Lymphedema	0.0001
Appendix Fig. S19B	Two-way ANOVA with Tukey's post hoc
HFD - Sham vs. Lymphedema	0.0111
HSFD - Sham vs. Lymphedema	0.0054
Sham CD vs. Sham HFD	0.0002
Sham CD vs. Sham HSFD	<0.0001
Lymphedema CD vs. Lymphedema HFD	<0.0001
Lymphedema CD vs. Lymphedema HSFD	<0.0001
Appendix Fig. S20C	One-way ANOVA with Tukey's post hoc
LE CD vs. LE HSFD	0.0007
LE HSFD vs. LE HSFD→CD	0.0028
Appendix Fig. S20D	One-way ANOVA with Tukey's post hoc
LE CD vs. LE HSFD	0.0032
LE HSFD vs. LE HSFD→CD	0.0096
Appendix Fig. S20E	One-way ANOVA with Tukey's post hoc
LE CD + PBS vs. LE HSFD + PBS	0.0175
Appendix Fig. S20F	One-way ANOVA with Tukey's post hoc
LE CD + PBS vs. LE HSFD + PBS	0.0249

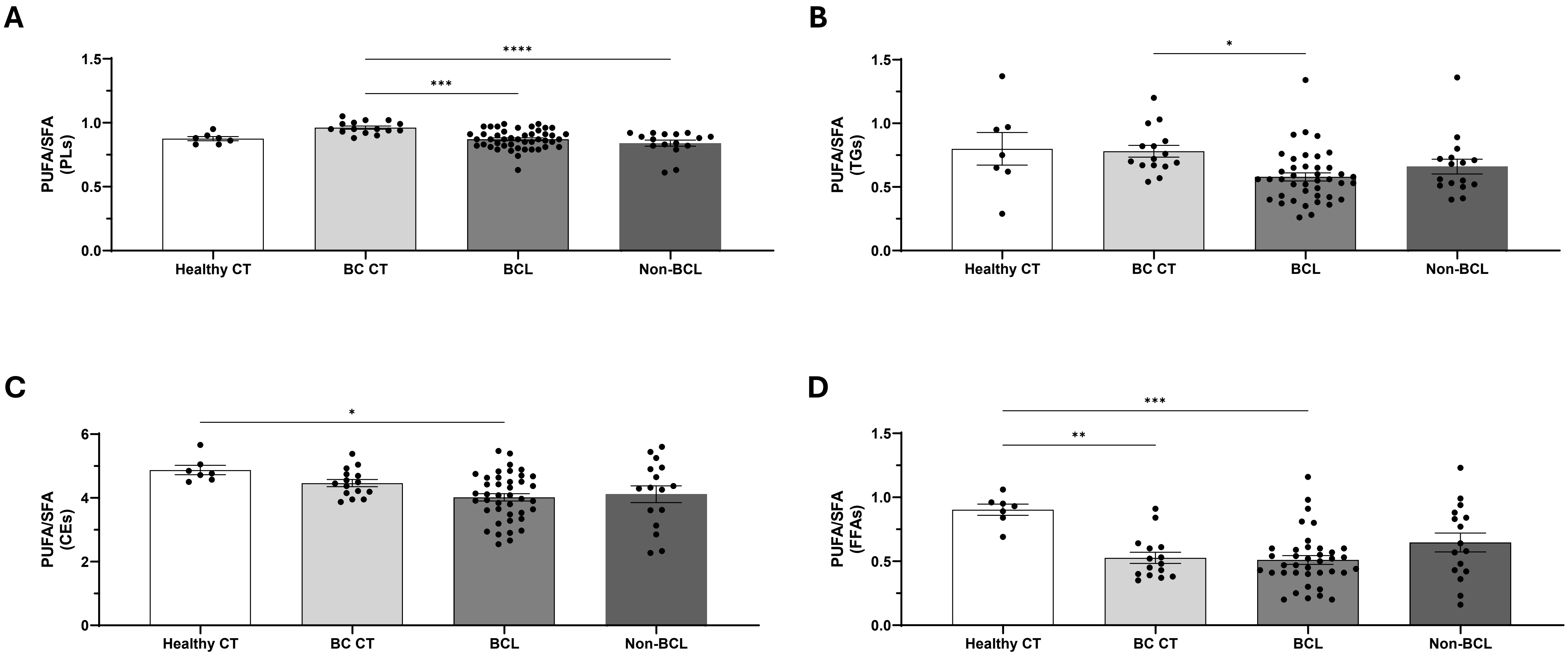

Appendix Figure S1. Altered fatty acid composition in plasma lipid fractions of lymphedema patients compared to controls. Scatter plots show the PUFA/SFA ratio in plasma fatty acids among non-lymphedema healthy controls (Healthy CT, *n*=7), non-lymphedema breast cancer controls (BC CT, *n*=15), breast cancer-related lymphedema (BCL, *n*=46), and non-BCL (*n*=16) for (**A**) phospholipids (PLs), (**B**) triglycerides (TGs), (**C**) cholesteryl esters (CEs), and (**D**) free fatty acids (FFAs). Data are presented as mean ± SEM. Statistical analysis: one-way ANOVA with Tukey's post hoc test. Significance: *P < 0.05, **P < 0.01, ***P < 0.001. PUFA: polyunsaturated fatty acid; SFA: saturated fatty acid.

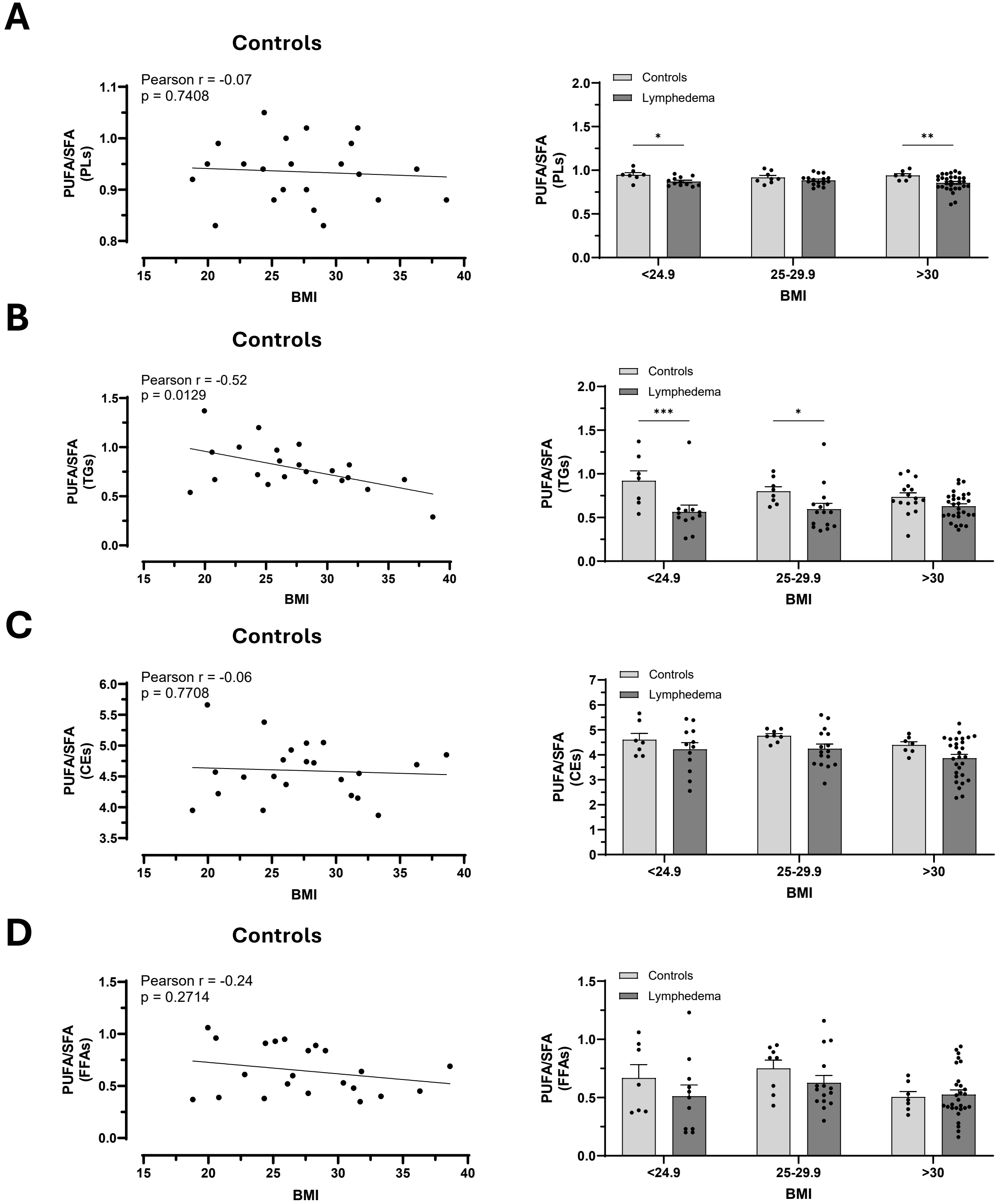

Appendix Figure S2. Associations between plasma PUFA/SFA ratio and BMI in controls and lymphedema patients. Relationship between BMI and plasma PUFA/SFA ratios across different lipid fractions in control (*n*=22) and lymphedema patients (*n*=57) for (**A**) phospholipids (PLs), (**B**) triglycerides (TGs), (**C**) cholesteryl esters (CEs), and (**D**) free fatty acids (FFAs). Left panels: Pearson correlation analysis between PUFA/SFA ratio and BMI in controls. Right panels: PUFA/SFA ratios stratified by BMI ranges of <25 (control *n*=7, lymphedema *n*=12), 25–30 (control *n*=8, lymphedema *n*=16), >30 (control *n*=7, lymphedema *n*=29) for each lipid fraction. Data are presented as mean ± SEM. Statistical analysis: two-tailed unpaired t-test or two-way ANOVA with Tukey's post hoc test, as appropriate. Significance: *P < 0.05, **P < 0.01. PUFA: polyunsaturated fatty acid; SFA: saturated fatty acid; BMI: body mass index.

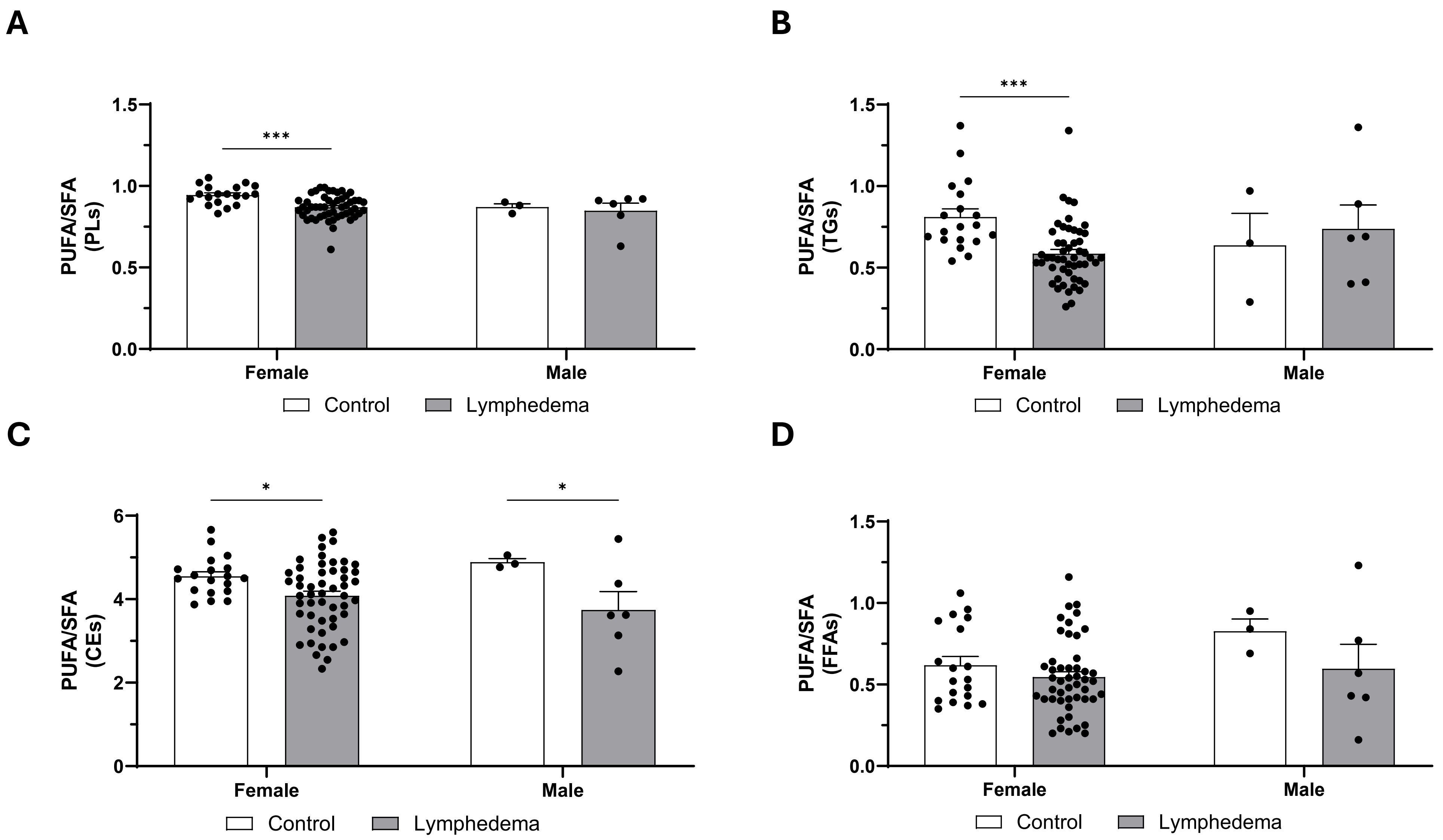

Appendix Figure S3. PUFA/SFA ratio in plasma FFAs stratified by sex. Scatter plots show the PUFA/SFA ratio in plasma fatty acid fractions in female and male participants, comparing non-lymphedema controls (female n=19, male n=3) and patients with secondary lymphedema (female n=51, male n=6) across (**A**) phospholipids (PLs), (**B**) triglycerides (TGs), (**C**) cholesteryl esters (CEs), and (**D**) free fatty acids (FFAs). No significant sex-specific differences in PUFA/SFA ratios were observed within either the control or lymphedema groups. Data are presented as mean ± SEM. Statistical analysis: two-way ANOVA with Tukey's post hoc test. Significance: *$P < 0.05$, ***$P < 0.001$. PUFA: polyunsaturated fatty acid; SFA: saturated fatty acid.

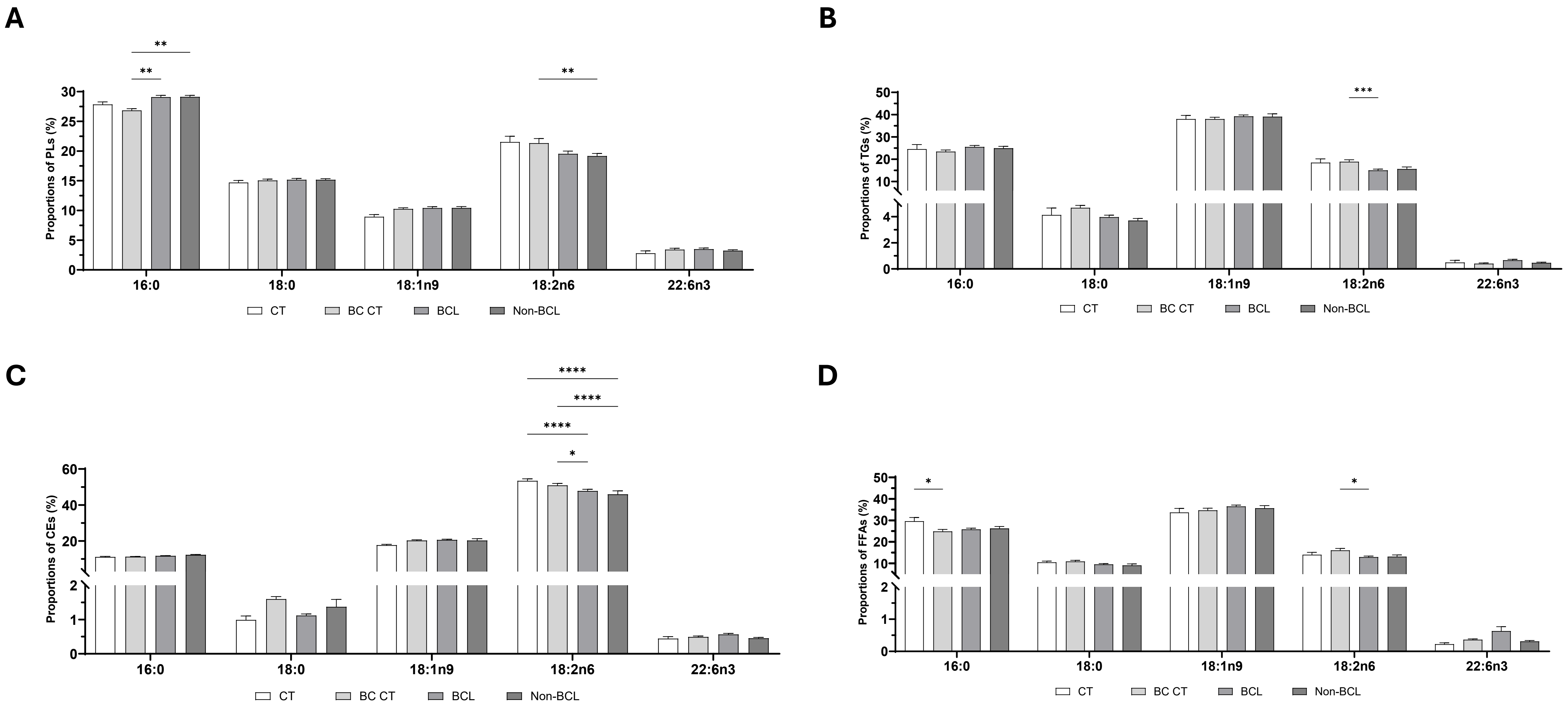

Appendix Figure S4. Fatty acid composition in plasma lipid fractions of lymphedema patients and controls. Relative abundance of major fatty acid species—palmitic acid (16:0), stearic acid (18:0), oleic acid (18:1n9), linoleic acid (18:2n6), and docosahexaenoic acid (22:6n3)—across plasma lipid fractions: (**A**) phospholipids (PLs), (**B**) triglycerides (TGs), (**C**) cholesteryl esters (CEs), and (**D**) free fatty acids (FFAs). Comparisons are shown among non-lymphedema healthy controls (Healthy CT, *n*=7), non-lymphedema breast cancer controls (BC CT, *n*=15), breast cancer-related lymphedema (BCL, *n*=46), and non-BCL (*n*=16) lymphedema patients. Data are presented as mean ± SEM. Statistical analysis: two-way ANOVA with Šídák's post hoc test. Significance: *P < 0.05, **P < 0.01, ***P < 0.001, ****P < 0.0001.

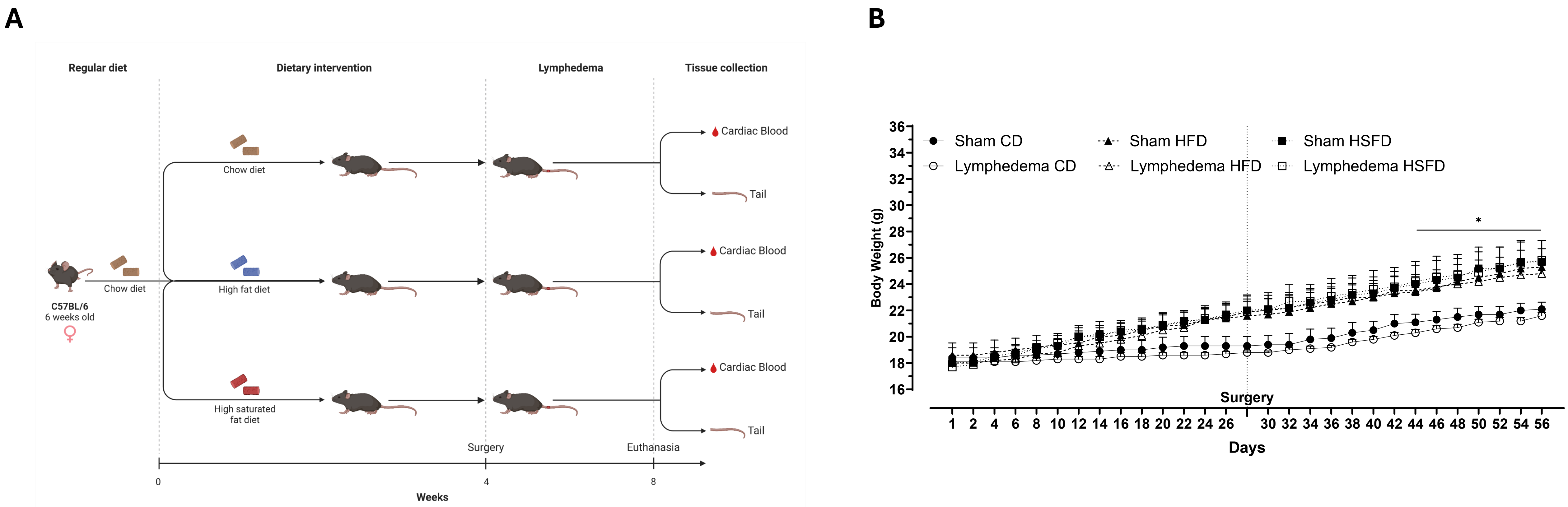

Appendix Figure S5. Dietary interventions in the mouse tail lymphedema model. (**A**) Experimental design illustrating dietary interventions and timeline. Female C57BL/6 mice (6 weeks old) were placed on a standard chow diet (CD), high fat diet (HFD), or high saturated fat diet (HSFD) for 4 weeks before undergoing surgical lymphatic ablation to induce lymphedema. Sham surgery involved only skin incision. Dietary interventions continued for an additional 4 weeks before tissue collection. Blood and tail samples were harvested at the endpoint for analysis. (**B**) Body weight measurements over time for sham and lymphedema mice across dietary interventions. Data are presented as mean ± SEM from *n*=5. Statistical analysis: two-way ANOVA with Šídák's post hoc test. Significance: *P < 0.05. HSFD: high saturated fat diet; HFD: high fat diet; CD: chow diet.

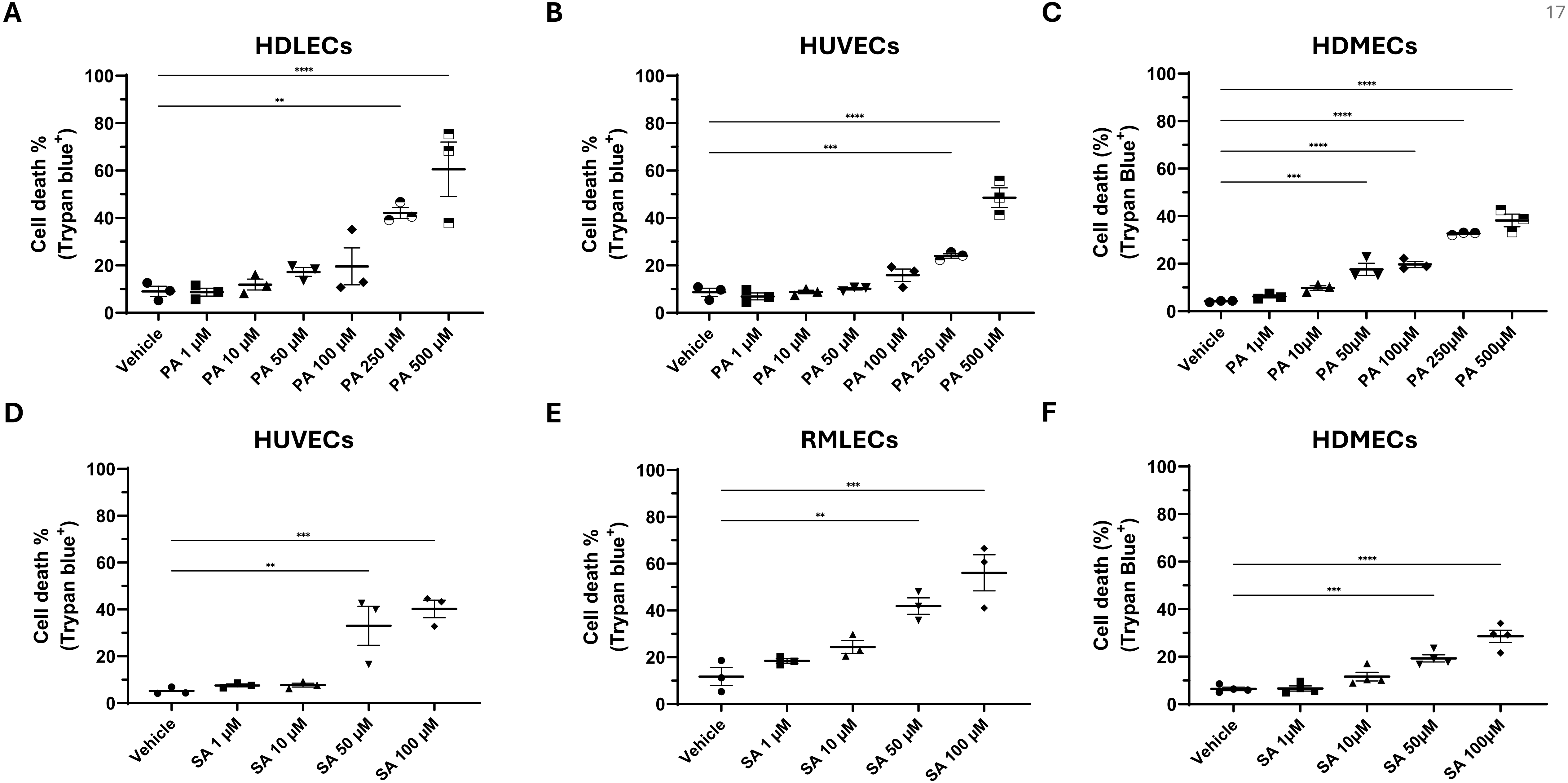

Appendix Figure S6. Effects of palmitic acid (PA) and stearic acid (SA) on endothelial cell viability. Cell death was assessed by trypan blue exclusion assay in various endothelial cell types exposed to increasing concentrations of saturated fatty acids. (**A–C**) Human Dermal Lymphatic Endothelial Cells (HDLECs), Human Umbilical Vein Endothelial Cells (HUVECs), and Human Dermal Microvascular Endothelial Cells (HDMECs) treated with PA. (**D–F**) HUVECs, Rat Mesenteric Lymphatic Endothelial Cells (RMLECs), and HDMECs treated with SA. Data are presented as mean ± SEM from *n*=3. Statistical analysis: one-way ANOVA with Tukey's post hoc test. Significance: *P < 0.05, **P < 0.01, ***P < 0.001, ****P < 0.0001.

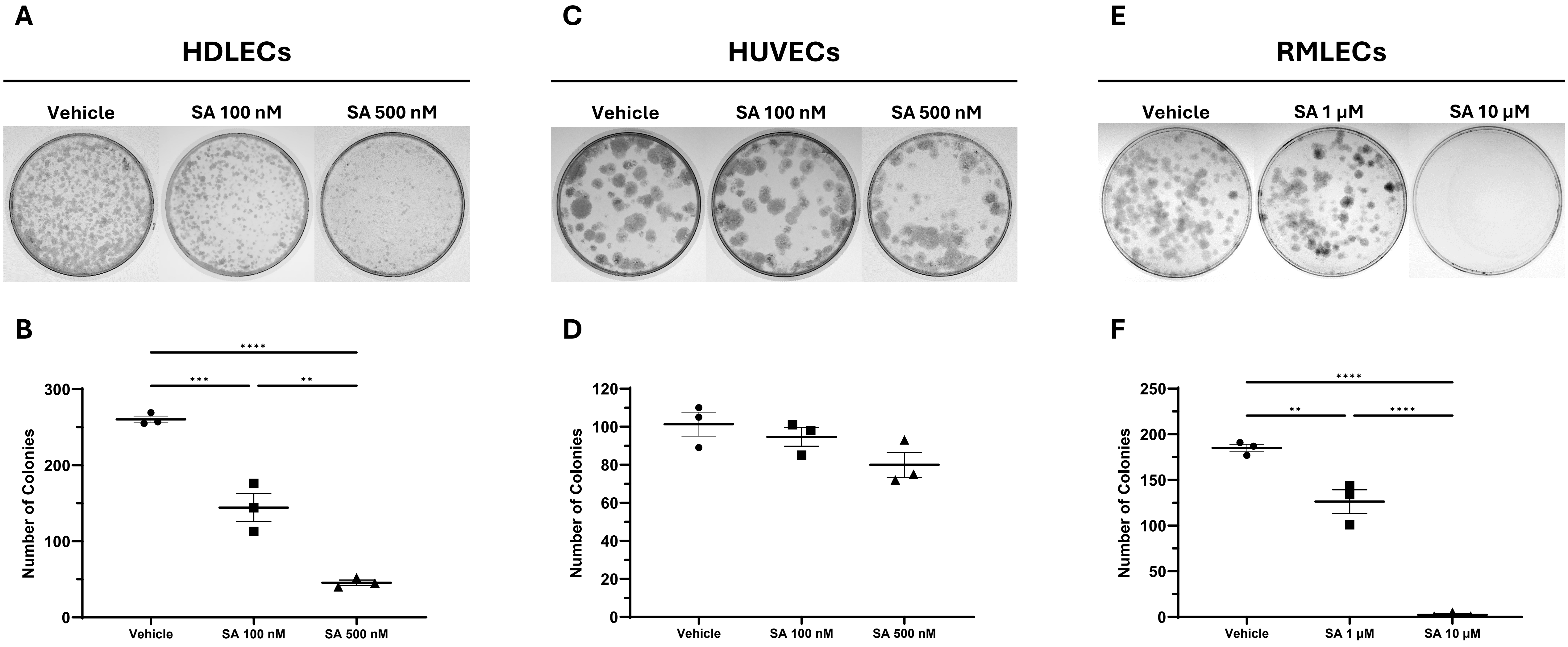

Appendix Figure S7. Stearic acid (SA) reduces colony-forming ability in endothelial cells. The colony-forming ability of endothelial cells was assessed following treatment with increasing concentrations of stearic acid (SA). Representative images and quantification of colonies are shown. (**A, B**) Human Dermal Lymphatic Endothelial Cells (HDLECs) treated with vehicle, 100 nM SA, or 500 nM SA. (**C, D**) Human Umbilical Vein Endothelial Cells (HUVECs) treated with vehicle, 100 nM SA, or 500 nM SA. (**E, F**) Rat Mesenteric Lymphatic Endothelial Cells (RMLECs) treated with vehicle, 1 μM SA, or 10 μM SA. Data are presented as mean ± SEM from *n*=3. Statistical analysis: one-way ANOVA with Tukey's post hoc test. Significance: $^{**}P < 0.01$, $^{***}P < 0.001$, $^{****}P < 0.0001$.

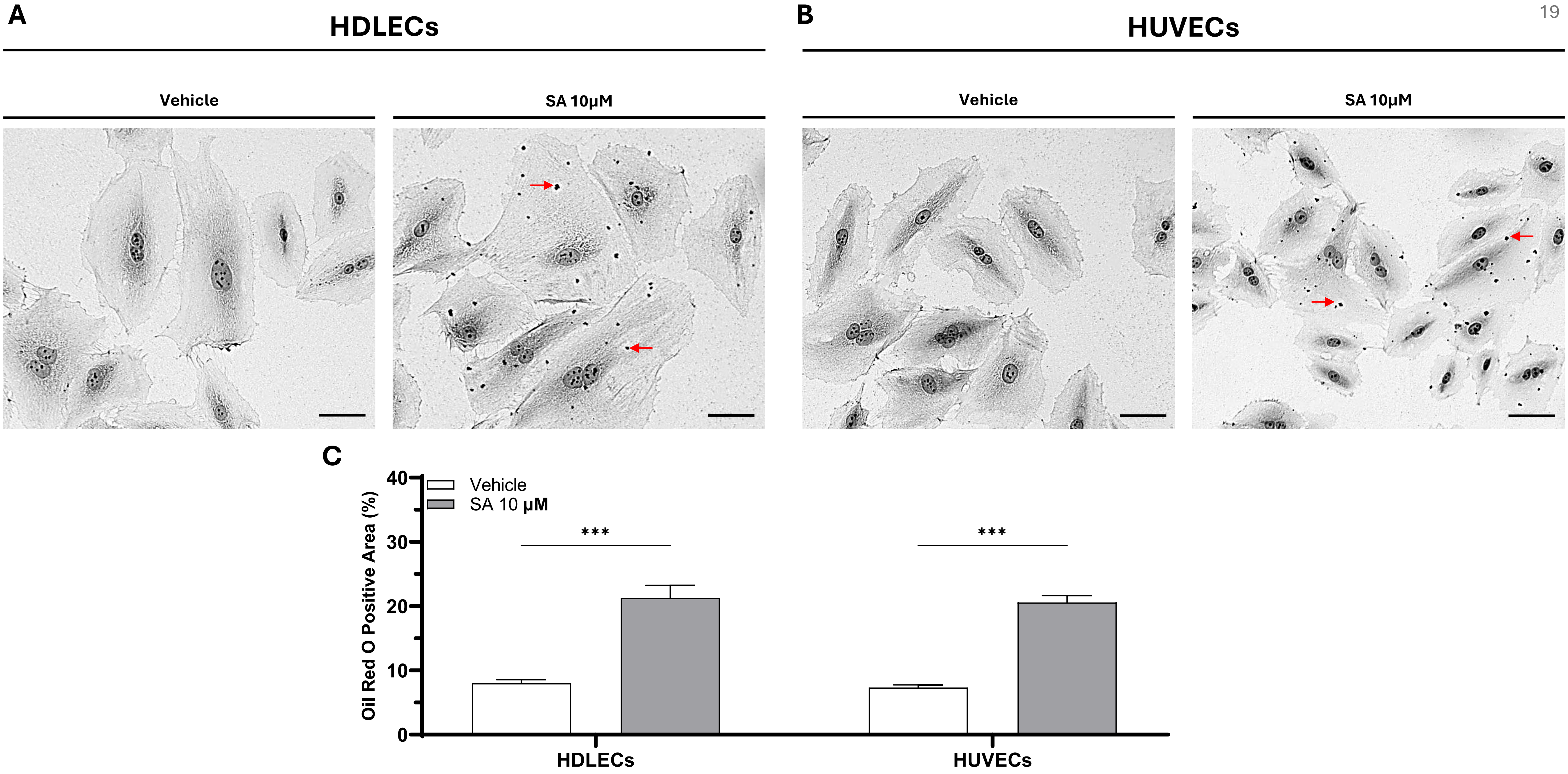

Appendix Figure S8. Lipid droplet formation in endothelial cells treated with stearic acid (SA). Lipid accumulation was evaluated by Oil Red O staining in (**A**) Human Dermal Lymphatic Endothelial Cells (HDLECs) and (**B**) Human Umbilical Vein Endothelial Cells (HUVECs) following treatment with 10 μM SA. Representative images show intracellular lipid droplets (red arrows) in both cell types after SA exposure compared to vehicle-treated controls. (**C**) Quantification of Oil Red O-positive area demonstrates comparable lipid droplet formation in HDLECs and HUVECs, suggesting similar SA uptake between the two cell types. Scale bars: 20 μm. Data are presented as mean ± SEM from *n*=4. Statistical analysis: one-way ANOVA with Tukey's post hoc test. Significance: ***$P < 0.001$.

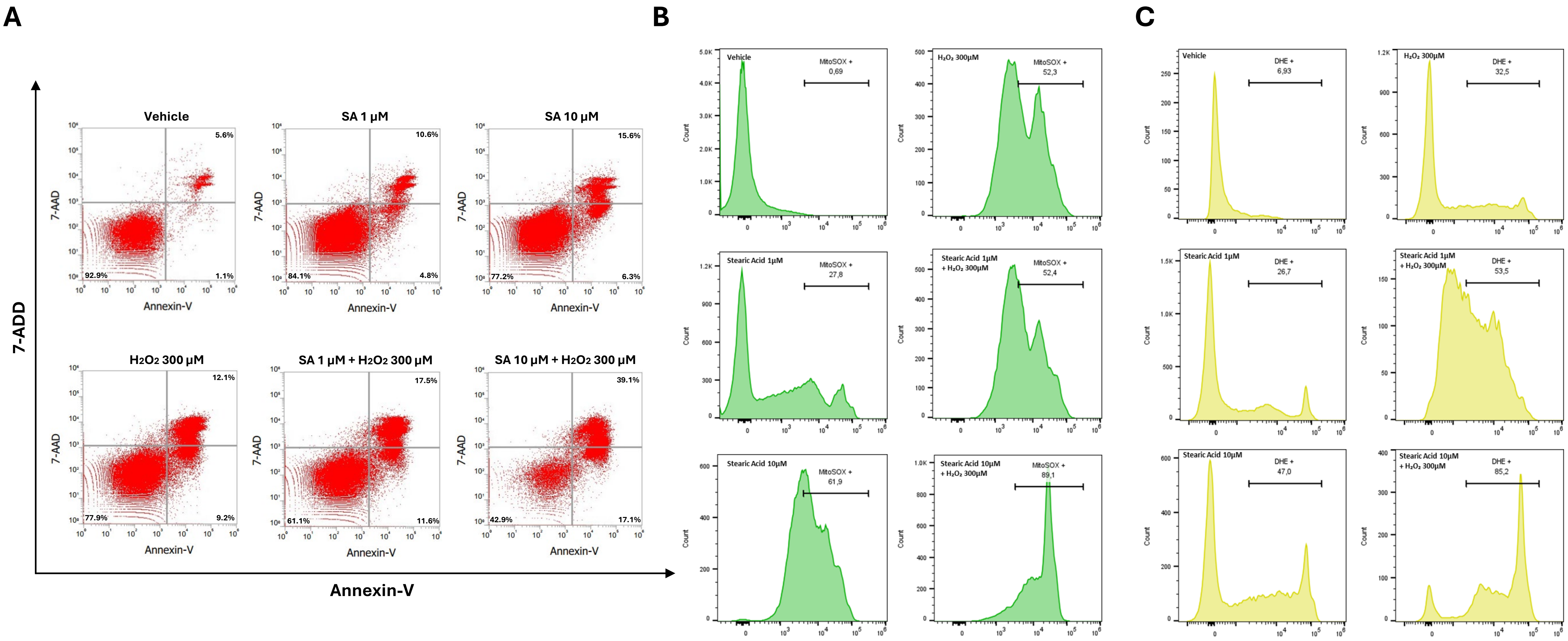

Appendix Figure S9. Stearic acid (SA) and hydrogen peroxide (H_2O_2) induce apoptosis and reactive oxygen species (ROS) in endothelial cells. (**A**) Representative dot plot images from annexin V/7-AAD flow cytometry analysis showing apoptosis in endothelial cells treated with vehicle, SA (1 μM or 10 μM), H_2O_2 (300 μM), or a combination of SA and H_2O_2 for 24 hours. The combination treatment significantly increased annexin V/7-AAD-positive cells, indicating enhanced apoptosis. (**B**) Representative flow cytometry histograms of MitoSOX Red staining measuring mitochondrial ROS levels under the same treatment conditions. A marked increase in mitochondrial ROS was observed in cells treated with the combination of SA and H_2O_2 compared to individual treatments. (**C**) Representative flow cytometry histograms of dihydroethidium (DHE) staining indicating cytosolic ROS levels. Cytosolic ROS were significantly elevated in cells treated with SA and H_2O_2, with the combination treatment showing the highest levels.

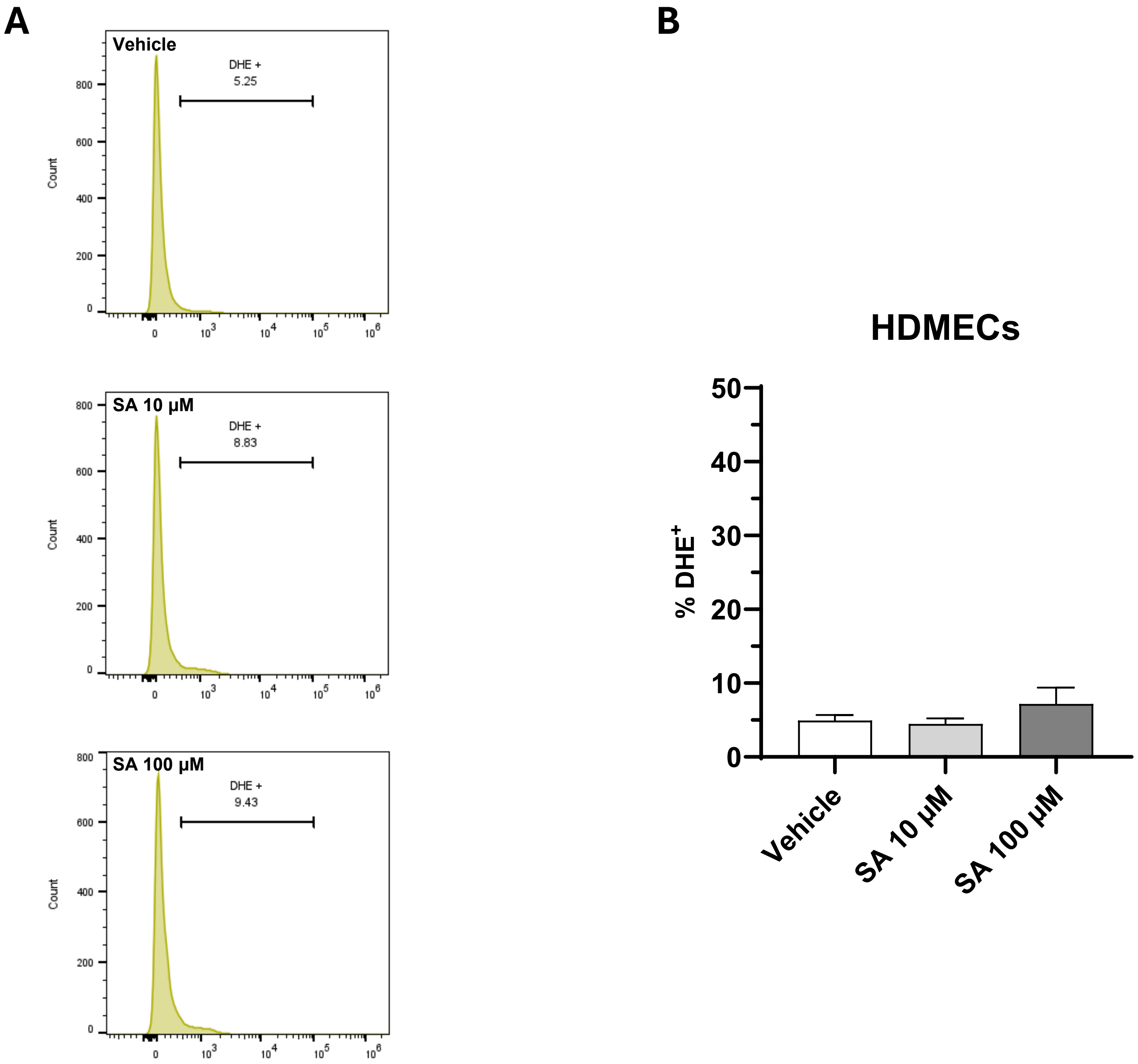

Appendix Figure S10. Effect of stearic acid (SA) on cytosolic reactive oxygen species (ROS) in Human Dermal Microvascular Endothelial Cells (HDMECs). (**A**) Representative flow cytometry histograms of dihydroethidium (DHE) staining indicating cytosolic ROS levels. (**B**) Quantification of DHE^{+} HDMECs following treatment with vehicle, 10 µM SA, or 100 µM SA for 24 hours. No significant increase in cytosolic ROS levels was observed at either concentration. Data are presented as mean ± SEM from *n*=3. Statistical analysis: one-way ANOVA with Tukey's post hoc test.

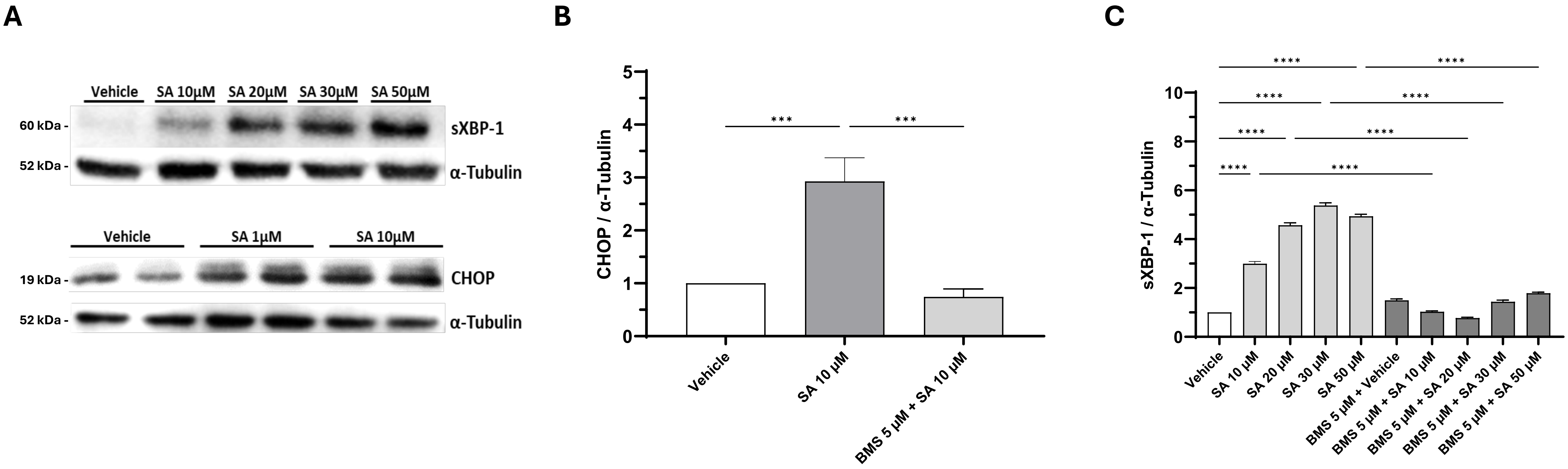

Appendix Figure S11. Stearic acid (SA) induces ER stress in lymphatic endothelial cells, which is attenuated by FABP4 inhibition. (**A**) Representative immunoblots showing CHOP and sXBP-1 expression in human dermal lymphatic endothelial cells (HDLECs) treated with increasing concentrations of SA for 24 hours. (**B**) Quantification of CHOP levels normalized to α-Tubulin in HDLECs treated with vehicle, 10 μM SA, or 10 μM SA in combination with the FABP4 inhibitor BMS-309403 (5 μM). CHOP expression was significantly upregulated by SA and reduced by BMS treatment. (**C**) Quantification of sXBP-1/α-Tubulin levels showing a dose-dependent increase in response to SA, which was consistently attenuated by BMS co-treatment. Data are presented as mean ± SEM from *n*=3. Statistical analysis: one-way ANOVA with Tukey's post hoc test. Significance ***P < 0.001, ****P < 0.0001. CHOP and sXBP-1 are markers of endoplasmic reticulum (ER) stress. FABP4: fatty acid-binding protein 4. Panel A reuses the blots shown in Figure EV4C

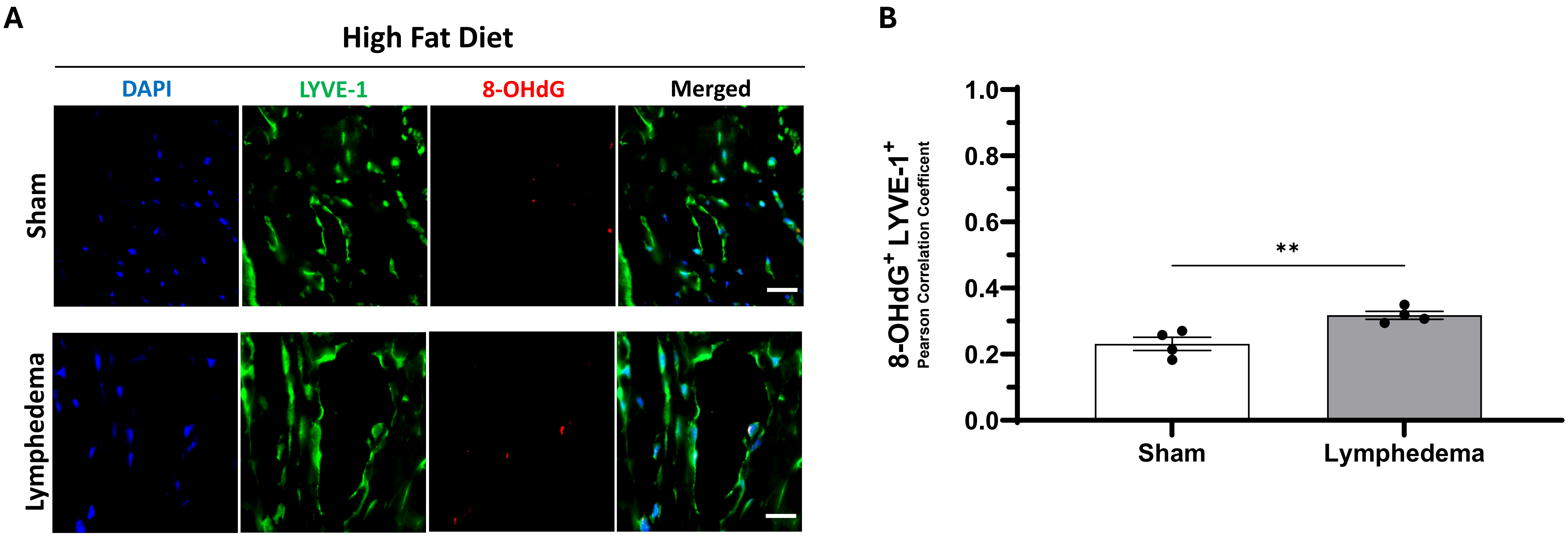

Appendix Figure S12. Increased oxidative DNA damage in lymphatic endothelial cells of lymphedematous mice tissue under high-fat diet (HFD). (**A**) Representative immunofluorescence images of tail skin sections from high-fat diet–fed mice subjected to sham surgery or lymphedema induction. Tissues were stained for DAPI (nuclei, blue), LYVE-1 (lymphatic vessels, green), and 8-hydroxy-2'-deoxyguanosine (8-OHdG, a marker of oxidative DNA damage, red). Merged images highlight colocalization of 8-OHdG with LYVE-1^{+} cells. (**B**) Quantification of 8-OHdG colocalization with LYVE-1^{+} cells using Pearson correlation coefficient analysis reveals significantly higher oxidative DNA damage in lymphatic vessels of lymphedema tissues compared to sham controls. Data are presented as mean ± SEM from *n*=4. Scale bars: 50 μm. Statistical analysis: unpaired two-tailed t-test. Significance: **P < 0.01. 8-OHdG: 8-hydroxy-2' -deoxyguanosine; LYVE-1: lymphatic vessel endothelial hyaluronan receptor-1; DAPI: 4',6-diamidino-2-phenylindole.

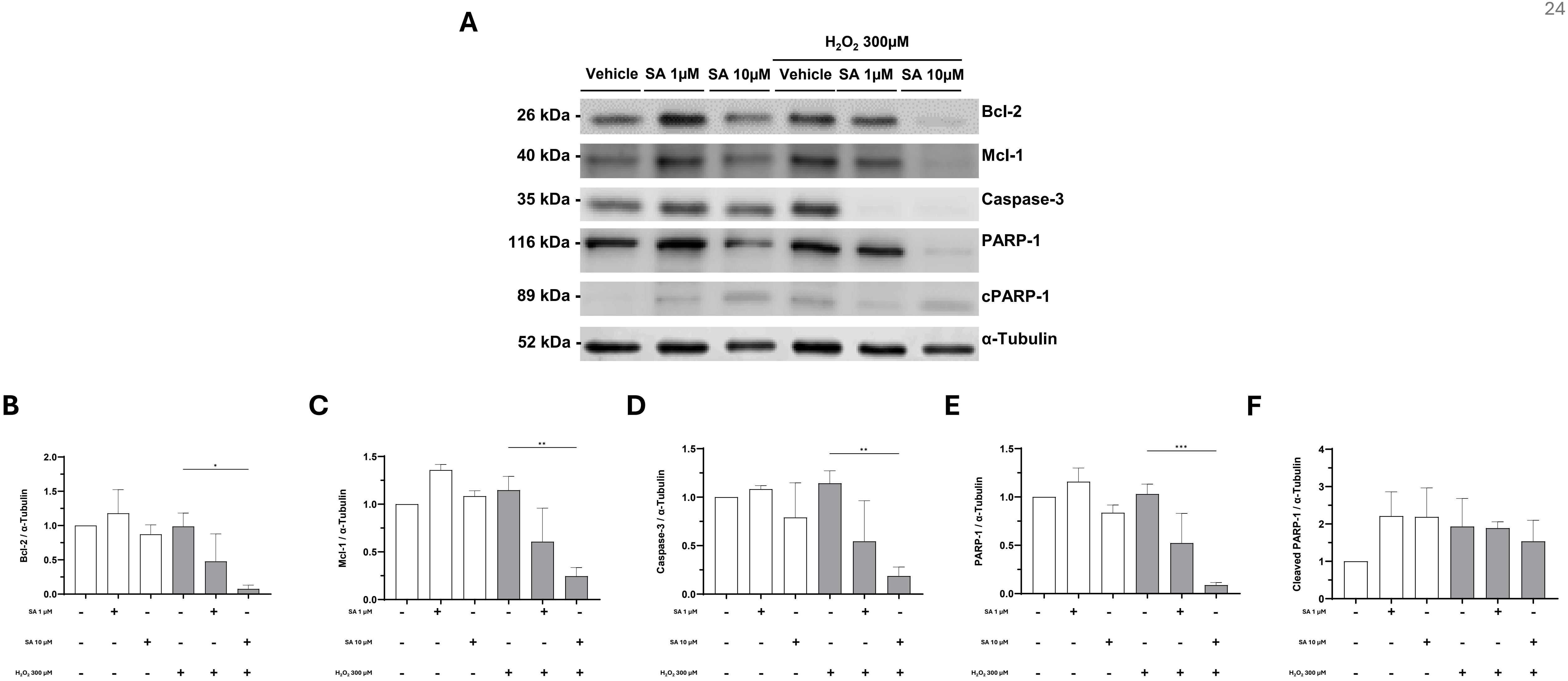

Appendix Figure S13. Modulation of apoptotic and anti-apoptotic proteins by stearic acid (SA) and oxidative stress in lymphatic endothelial cells. (**A**) Representative Western blot images of apoptosis-related proteins in HDLECs treated with SA and H_2O_2. Bar graphs show the relative expression levels of (**A**) Bcl-2, (**B**) Mcl-1, (**C**) total caspase-3, (**D**) total PARP-1, and (**E**) cleaved PARP-1 normalized to α-Tubulin in human dermal lymphatic endothelial cells treated with SA (1 µM and 10 µM), hydrogen peroxide (H_2O_2, 300 µM), or their combinations. Treatment with H_2O_2 alone or in combination with SA led to reductions in anti-apoptotic proteins Bcl-2 and Mcl-1, and in total PARP-1, suggesting enhanced apoptotic signaling. Data are presented as mean ± SEM from *n*=3. Statistical analysis: one-way ANOVA with Tukey's post hoc test. Significance: $^*P < 0.05$, $^{**}P < 0.01$, $^{***}P < 0.001$. Bcl-2: B-cell leukemia/lymphoma 2 protein; Mcl-1: myeloid cell leukemia sequence 1; PARP-1: poly(ADP-ribose) polymerase 1.

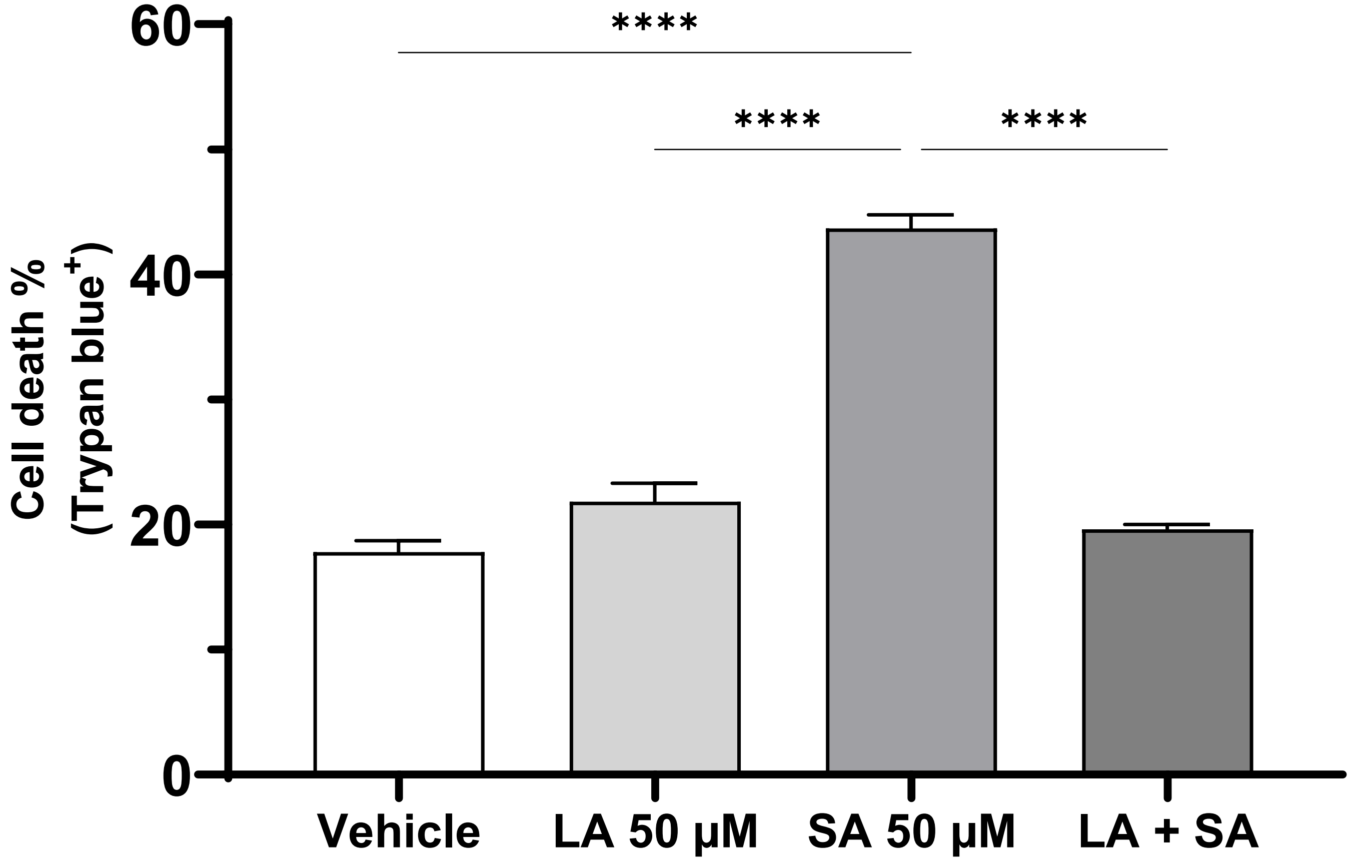

Appendix Figure S14. Effects of stearic acid (SA) and linoleic acid (LA) on lymphatic endothelial cell viability. Cell death percentages were assessed in endothelial cells treated with 50 µM SA and 50 µM LA, either alone or in combination, using the trypan blue exclusion assay. Data are presented as mean ± SEM from *n*=3. Statistical analysis: one-way ANOVA with Tukey's post hoc test. Significance: ****P < 0.0001. SA: stearic acid; LA: linoleic acid.

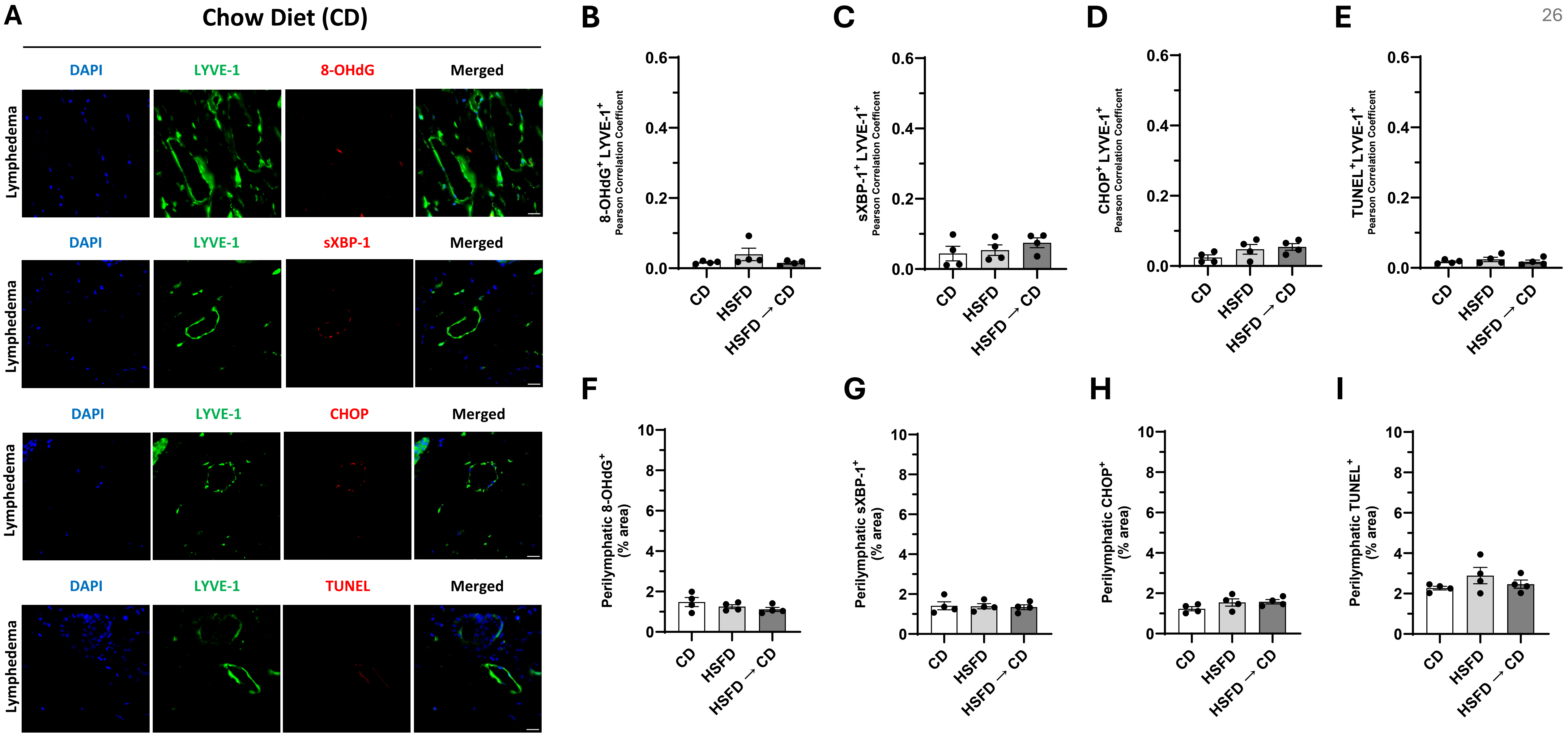

Appendix Figure S15. Immunofluorescence analysis of oxidative stress, ER stress, and apoptosis markers in mouse tail tissue. (**A**) Representative immunofluorescence images of tail tissue from mice fed a chow diet (CD) 28 days after lymphedema surgery, stained for LYVE-1 (green), DAPI (blue), and either 8-OHdG, sXBP-1, CHOP, or TUNEL (red). Scale bars: 50 μm. Quantification of staining in CD-fed mice, mice fed a high saturated fat diet (HSFD), and mice switched from HSFD to CD (HSFD → CD) is shown for the following markers: (**B, F**) colocalization of 8-OHdG with LYVE-1$^+$ lymphatic endothelial cells and perilymphatic 8-OHdG signal intensity; (**C, G**) sXBP-1 colocalization and perilymphatic signal; (**D, H**) CHOP colocalization and perilymphatic signal; and (**E, I**) TUNEL colocalization and perilymphatic signal. No significant differences were observed among groups. Data are presented as mean ± SEM from *n*=4. Statistical analysis: one-way ANOVA with Tukey's post hoc test. 8-OHdG: 8-hydroxy-2'-deoxyguanosine; LYVE-1: lymphatic vessel endothelial hyaluronan receptor-1; DAPI: 4',6-diamidino-2-phenylindole.

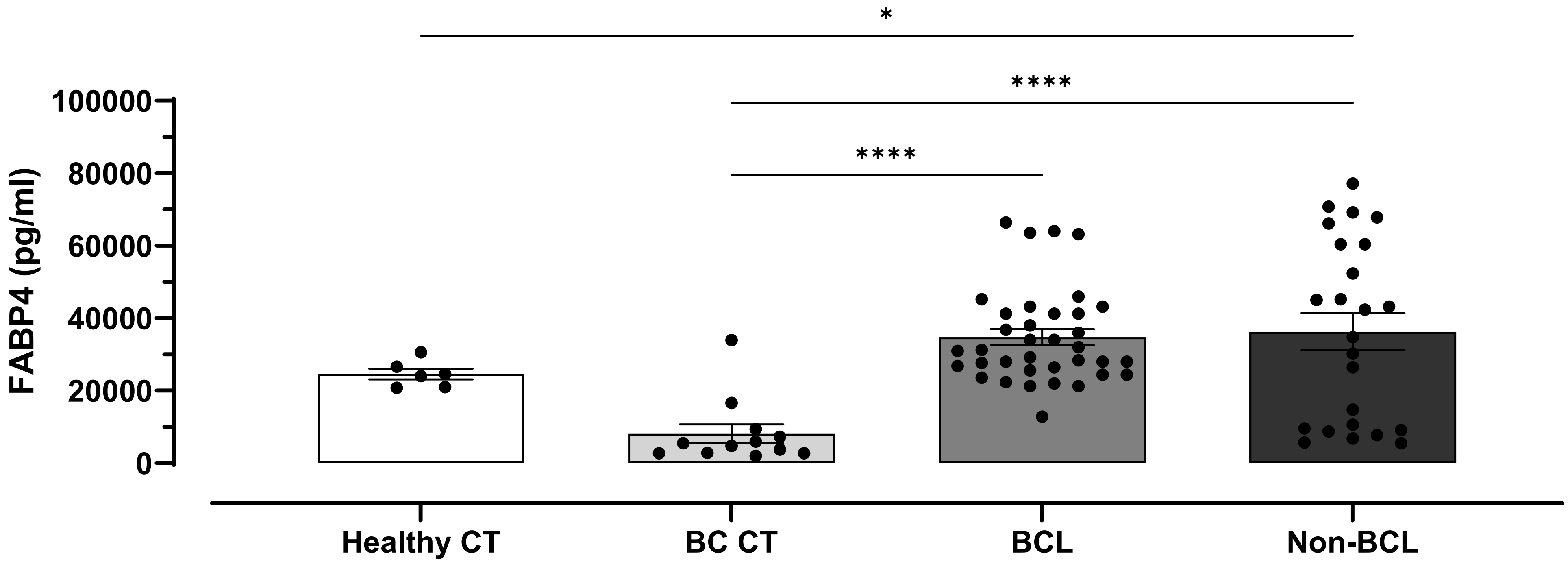

Appendix Figure S16. Circulating levels of fatty acid-binding protein 4 (FABP4) in plasma samples from patients with lymphedema and non-lymphedema controls. Scatter plots depict plasma FABP4 levels in non-lymphedema healthy controls (Healthy CT, *n*=6), non-lymphedema breast cancer controls (BC CT, *n*=12), patients with breast cancer-related lymphedema (BCL, *n*=36), and patients with non-BCL (*n*=24). Data are presented as mean ± SEM. Statistical analysis: one-way ANOVA with Tukey's post hoc test. Significance: *P < 0.05 , ***P < 0.001, ****P < 0.0001.

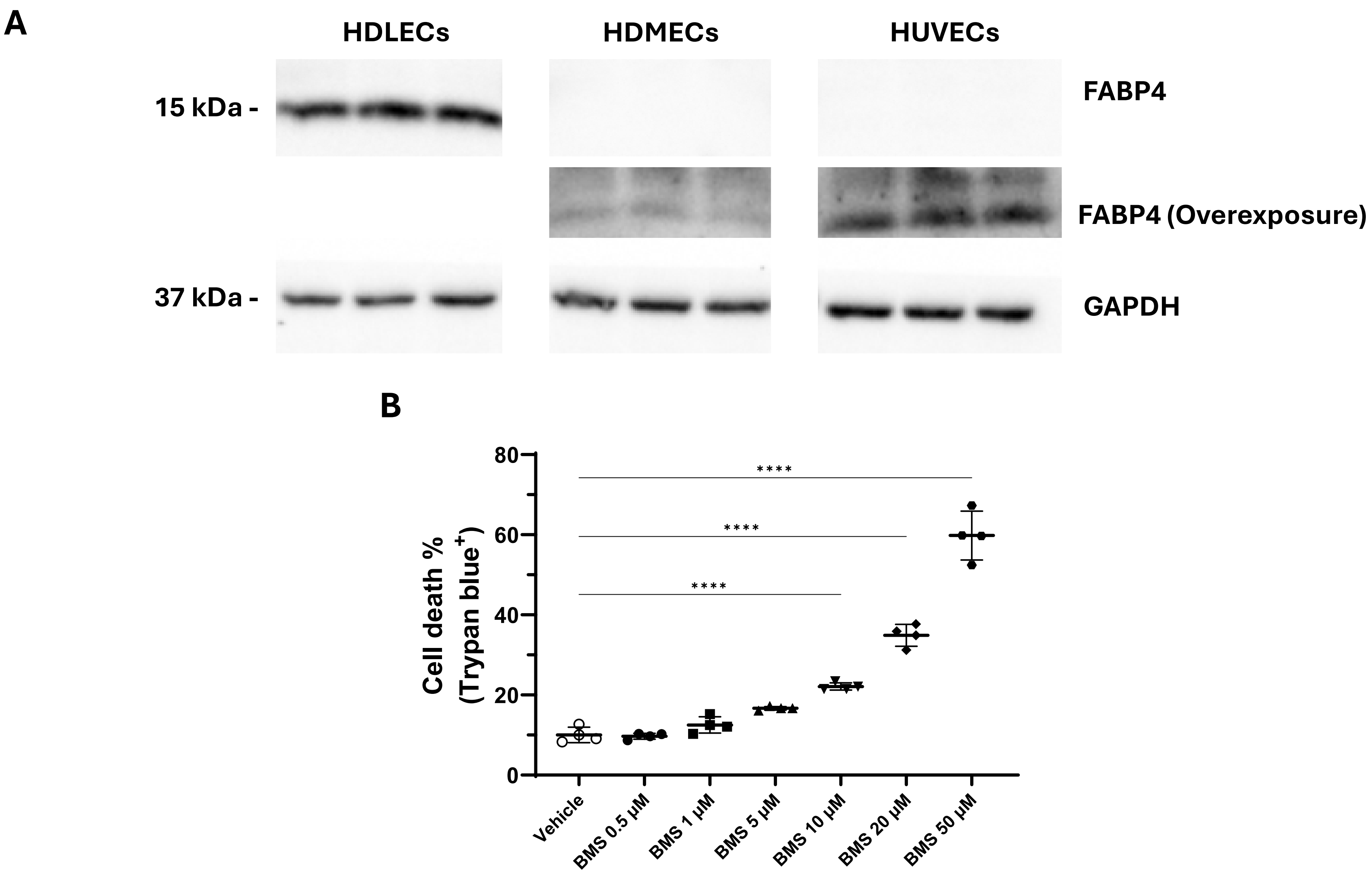

Appendix Figure S17. Fatty acid-binding protein 4 (FABP4) expression and the impact of FABP4 inhibition on endothelial cell viability. (**A**) Representative Western blot showing fatty acid-binding protein 4 (FABP4) expression in Human Dermal Lymphatic Endothelial Cells (HDLECs), Human Dermal Microvascular Endothelial Cells (HDMECs), and Human Umbilical Vein Endothelial Cells (HUVECs). FABP4 was readily detected in HDLECs but was minimal in HDMECs and HUVECs. (**B**) Quantification of cell death in HDLECs treated with increasing concentrations of the FABP4 inhibitor BMS-309403 (BMS), assessed via trypan blue exclusion assay. BMS showed minimal cytotoxicity at lower doses, with a dose-dependent increase in cell death observed at higher concentrations. Data are presented as mean ± SEM from *n*=4. Statistical analysis: one-way ANOVA with Tukey's post hoc test. Significance: ****$P < 0.0001$.

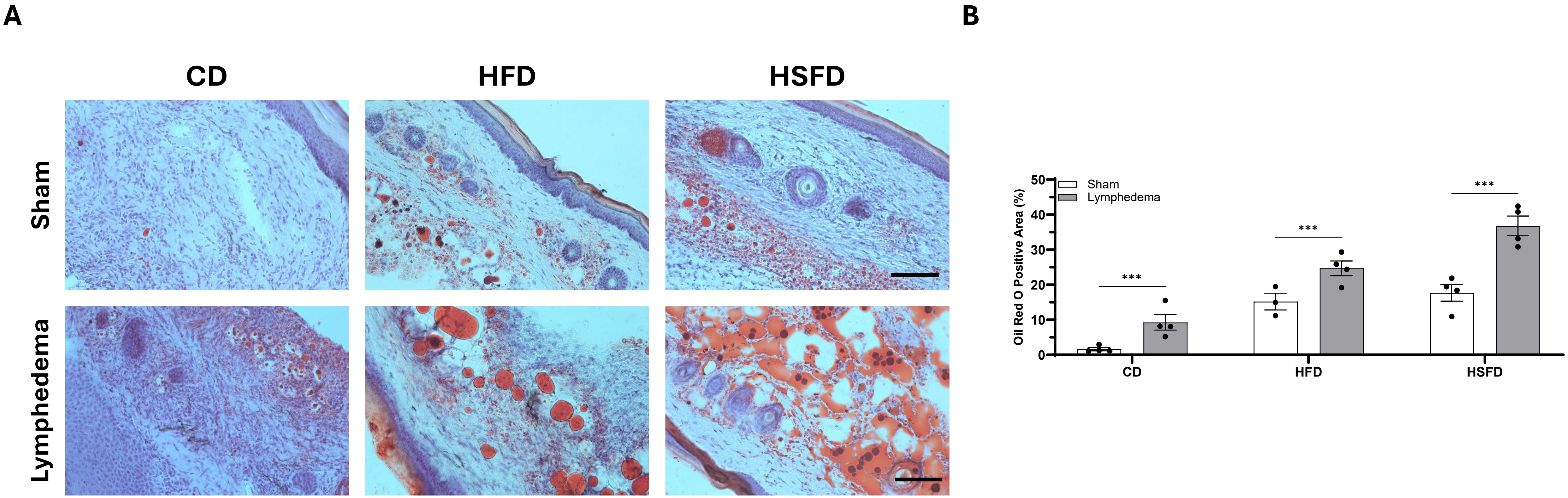

Appendix Figure S18. Diet-dependent lipid accumulation in lymphedematous tissue. Oil Red O staining of tail sections from sham and lymphedema mice fed a chow diet (CD), high-fat diet (HFD), or high saturated fat diet (HSFD). (**A**) Representative images show increased lipid accumulation (red) in lymphedematous skin, with more pronounced deposition observed under HFD and HSFD conditions. Scale bars: 100 µm. (**B**) Quantification of Oil Red O–positive area reveals significantly elevated lipid content in lymphedema tissues compared to sham controls across all diet groups, with the highest levels detected in HSFD-fed mice. Data are presented as mean ± SEM from *n*=4. Scale bars: 100 µm. Statistical analysis: two-way ANOVA with Tukey's post hoc test. Significance: ***P < 0.001. Panel B reuses the HSFD data shown in Figure EV4B.

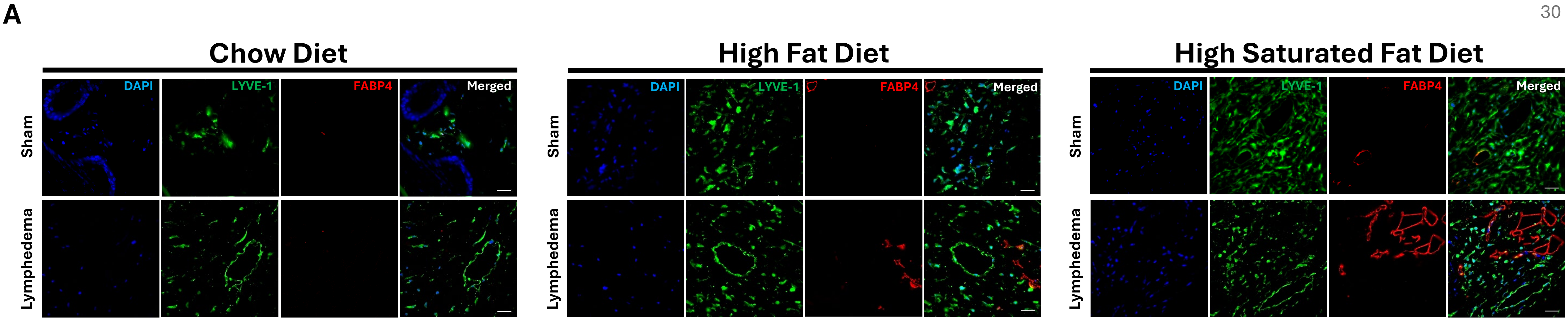

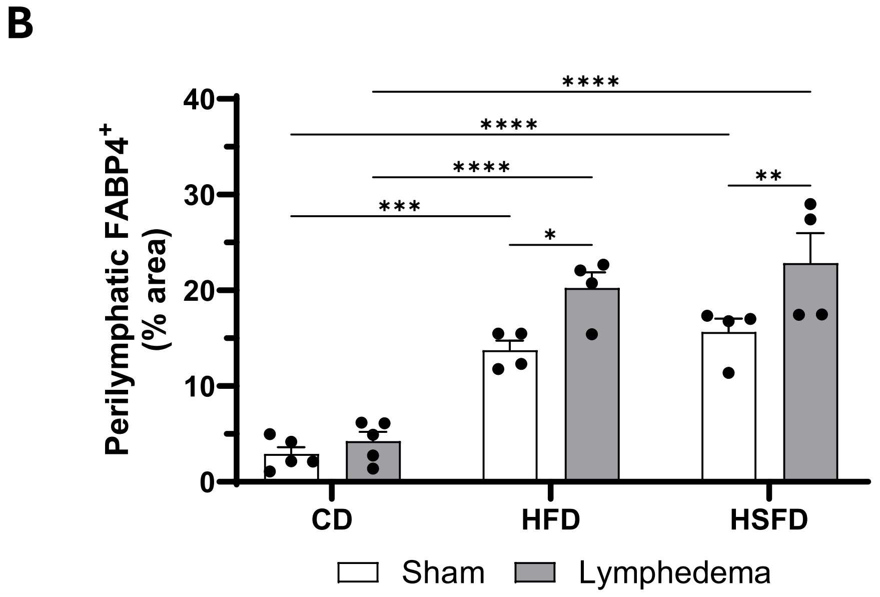

Appendix Figure S19. High saturated fat diet (HSFD) enhances perilymphatic FABP4 expression in a mouse model of lymphedema. (**A**) Representative immunofluorescence images showing perilymphatic fatty acid-binding protein 4 (FABP4, red) colocalized with LYVE-1-positive lymphatic endothelial cells (green) in sham and lymphedema tissues from mice fed a chow diet (CD), high fat diet (HFD), or HSFD. DAPI stains the nucleus (blue). Increased perilymphatic FABP4 expression is evident in lymphedema tissues, particularly in HSFD-fed mice. Scale bars: 50 μm. (**B**) Quantification of perilymphatic FABP4-positive area (% of total area) in sham and lymphedema tissues across dietary groups, showing significant increases in FABP4 expression in lymphedema mice, with the highest levels observed in HSFD-fed mice. Data are presented as mean ± SEM from *n*=4-5. Statistical analysis: two-way ANOVA with Tukey's post hoc test. Significance: *P < 0.05, **P < 0.01, ***P < 0.001, ****P < 0.0001. LYVE-1: lymphatic vessel endothelial hyaluronan receptor-1; FABP4: fatty acid-binding protein 4; DAPI: 4',6-diamidino-2-phenylindole.

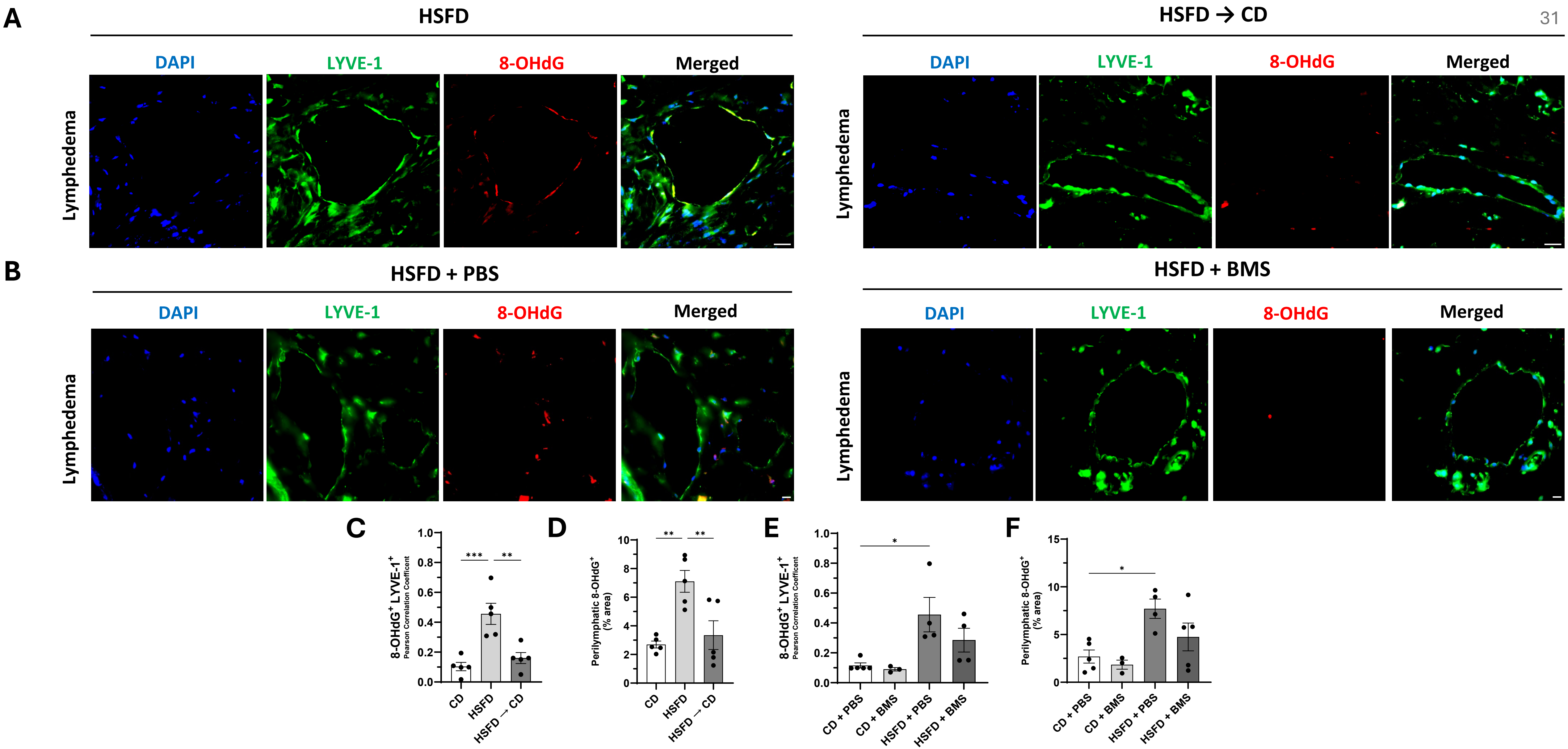

Appendix Figure S20. FABP4 inhibition and dietary intervention reduce oxidative DNA damage in lymphedematous tissue under high saturated fat diet (HSFD). (**A–B**) Representative immunofluorescence images of tail skin from lymphedema mice maintained on HSFD, switched to control diet (HSFD → CD), or treated with the FABP4 inhibitor BMS-309403 (HSFD + BMS). PBS-treated mice also served as controls (HSFD + PBS). Tissues were stained for DAPI (blue), LYVE-1 (green), and 8-OHdG (red). (**C, D**) Quantification of 8-OHdG^{+}/LYVE-1^{+} colocalization and perilymphatic 8-OHdG signal shows increased oxidative DNA damage in HSFD-fed mice, which is reduced by dietary transition. (**E, F**) BMS treatment similarly decreases 8-OHdG colocalization and perilymphatic staining. Data are presented as mean ± SEM from *n*=5. Statistical analysis: one-way ANOVA with Tukey's post hoc test. Significance: *P < 0.05, **P < 0.01, ***P < 0.001. 8-OHdG: 8-hydroxy-2'-deoxyguanosine; LYVE-1: lymphatic vessel endothelial hyaluronan receptor-1; DAPI: 4',6-diamidino-2-phenylindole.

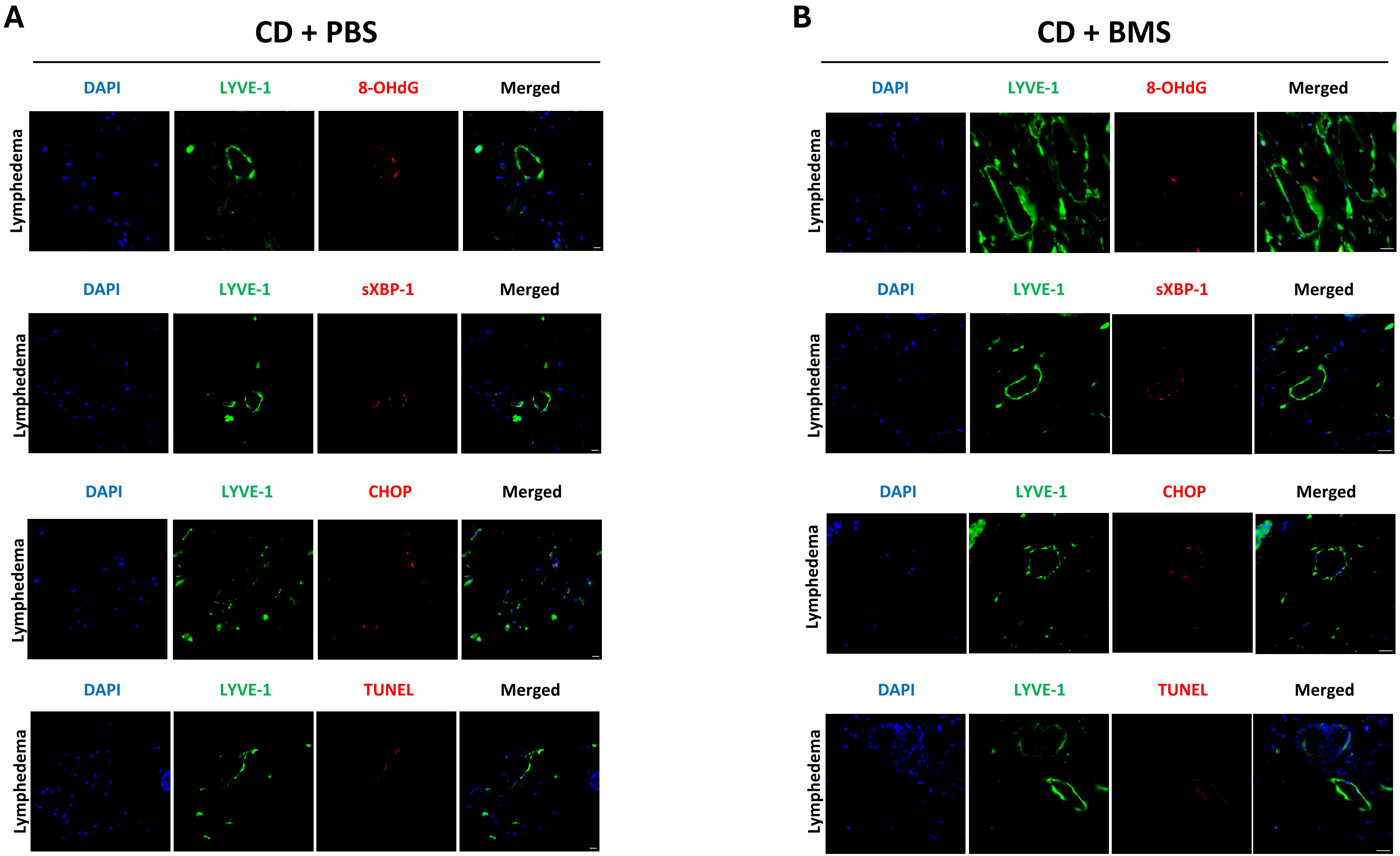

Appendix Figure S21. Immunofluorescence analysis of oxidative stress, ER stress, and apoptosis markers in lymphedematous mice fed a chow diet (CD). Representative immunofluorescence images of tail tissue from lymphedema mice maintained on a chow diet and treated with either vehicle (PBS, **A**) or the FABP4 inhibitor BMS-309403 (**B**). Sections were stained for DAPI (nuclei, blue), LYVE-1 (lymphatic vessels, green), and markers of oxidative stress (8-OHdG), ER stress (sXBP-1, CHOP), and apoptosis (TUNEL) in red. 8-OHdG: 8-hydroxy-2'-deoxyguanosine; sXBP-1: spliced X-box binding protein 1; CHOP: C/EBP homologous protein; TUNEL: terminal deoxynucleotidyl transferase dUTP nick end labeling; LYVE-1: lymphatic vessel endothelial hyaluronan receptor-1; DAPI: 4',6-diamidino-2-phenylindole.

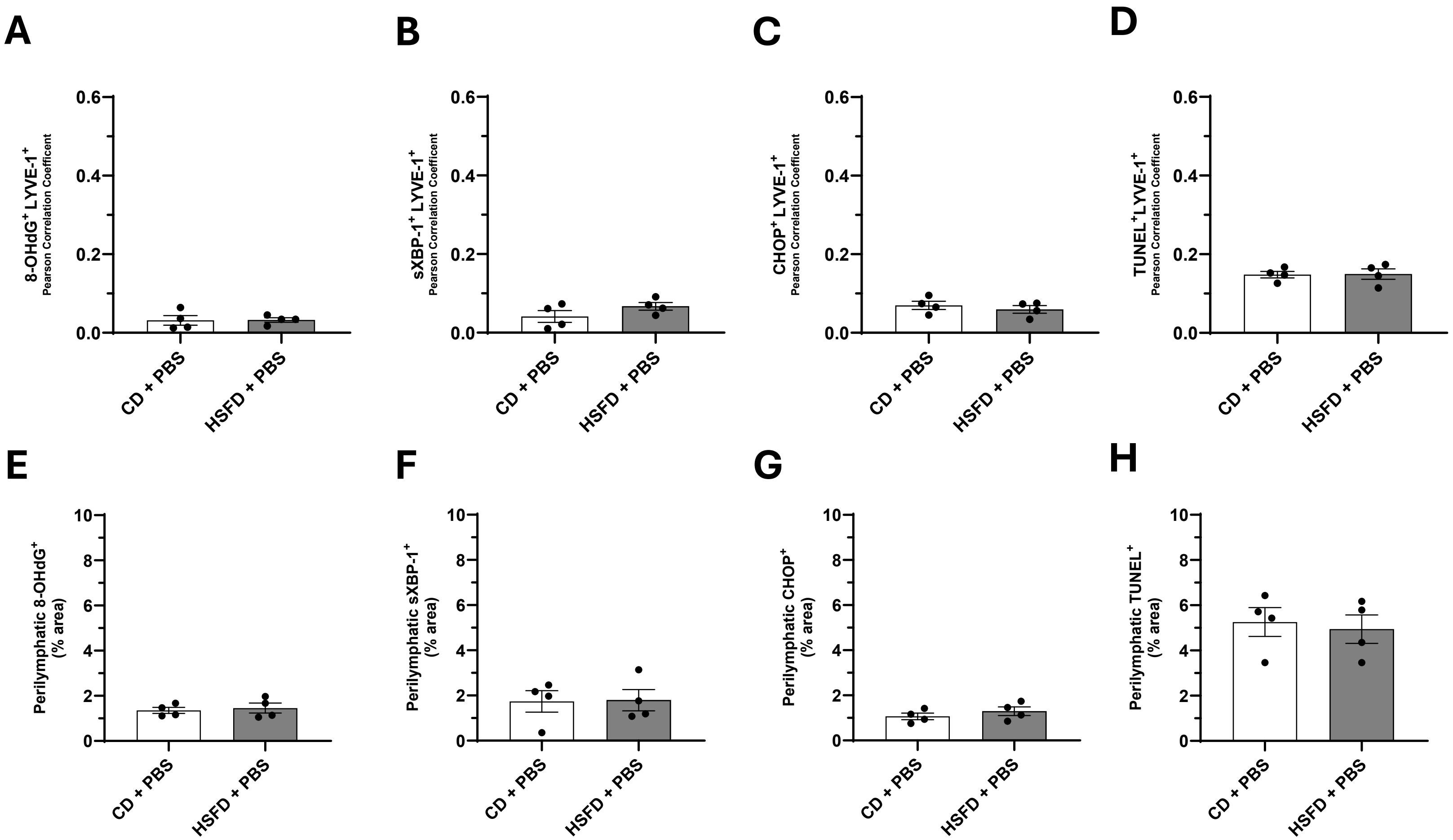

Appendix Figure S22. Protein levels detected by immunofluorescence staining in control mice 28 days post-sham surgery. Scatter plots illustrate the following: (**A, B**) colocalization of 8-OHdG+ and LYVE-1+ cells and perilymphatic 8-OHdG intensities; (**C, D**) colocalization of sXBP-1+ and LYVE-1+ cells and perilymphatic sXBP-1 intensities; (**E, F**) colocalization of CHOP+ and LYVE-1+ cells and perilymphatic CHOP intensities; (**G, H**) colocalization of TUNEL+ and LYVE-1+ cells and perilymphatic TUNEL intensities across experimental groups. Data are presented as mean ± SEM from *n*=4. Statistical analysis: one-way ANOVA with Tukey's post hoc test. LYVE-1: lymphatic vessel endothelial.

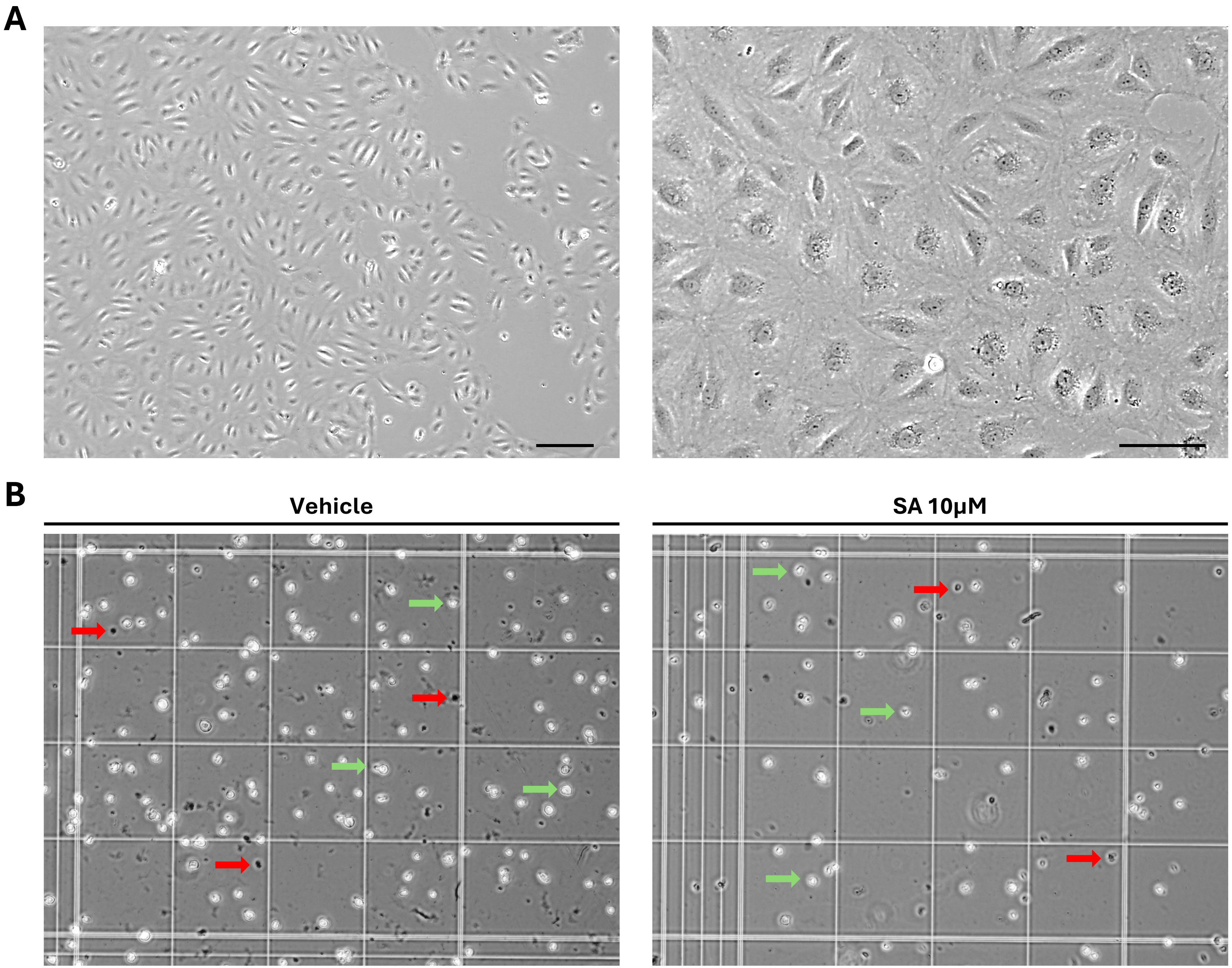

Appendix Figure S23. Representative images of cell confluency and viability assessment. (**A**) Representative phase-contrast images of endothelial cell cultures at ~70–80% confluency prior to treatment. Scale bars: 50 μm and 100 μm respectively. (**B**) Representative hemocytometer images from the trypan blue exclusion assay used to assess cell viability following treatment with vehicle or 10 μM stearic acid (SA). Red arrows indicate non-viable (trypan blue–positive) cells, and green arrows indicate viable (unstained) cells. Cell death was calculated as a percentage of total cells (dead/(live + dead) × 100).
