# Supplementary figures and images for: Saturated fatty acids induce lipotoxicity in lymphatic endothelial cells contributing to secondary lymphedema development (part 1 of 3)
Source: EMBO Mol Med. 2025 Aug 4;17(9):2384–408. doi: 10.1038/s44321-025-00286-4 (PMC12423331; doi:10.1038/s44321-025-00286-4)

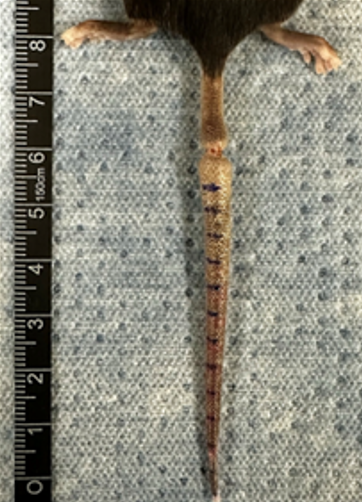

Supplement: Supplementary file 4 — Source data Fig. 2 [file 44321_2025_286_MOESM4_ESM.zip › Figure 2/2A/CD LE.tif]

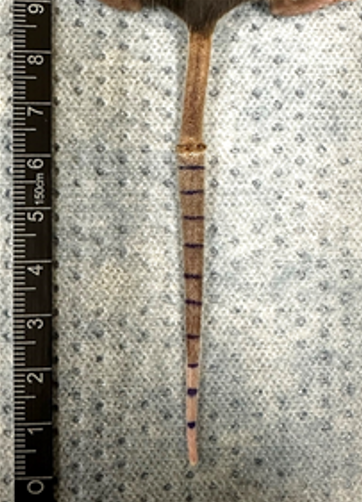

Supplement: Supplementary file 4 — Source data Fig. 2 [file 44321_2025_286_MOESM4_ESM.zip › Figure 2/2A/CD Sham.tif]

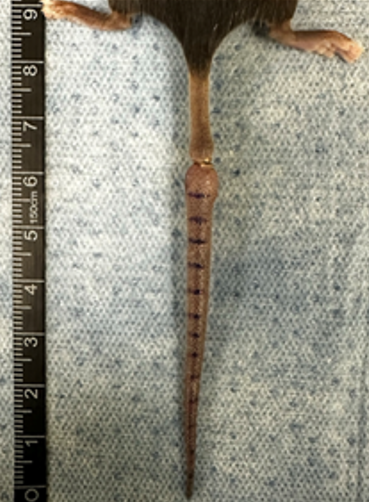

Supplement: Supplementary file 4 — Source data Fig. 2 [file 44321_2025_286_MOESM4_ESM.zip › Figure 2/2A/HFD LE.tif]

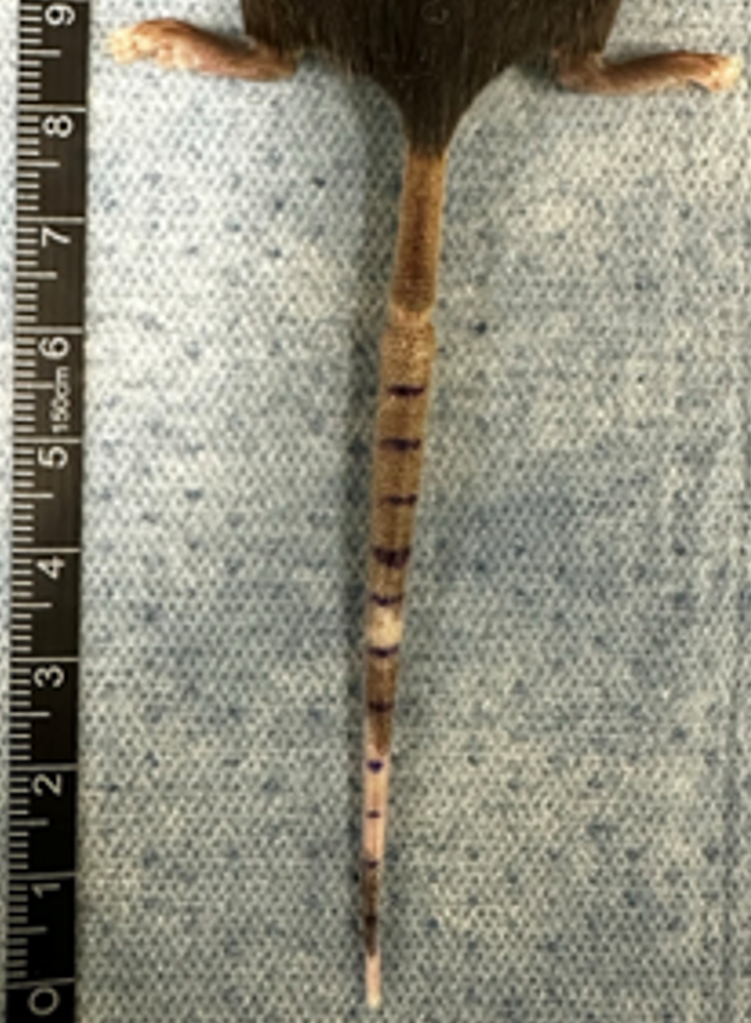

Supplement: Supplementary file 4 — Source data Fig. 2 [file 44321_2025_286_MOESM4_ESM.zip › Figure 2/2A/HFD Sham.tif]

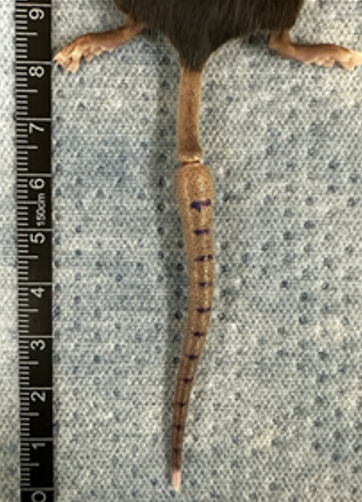

Supplement: Supplementary file 4 — Source data Fig. 2 [file 44321_2025_286_MOESM4_ESM.zip › Figure 2/2A/HSFD LE.tif]

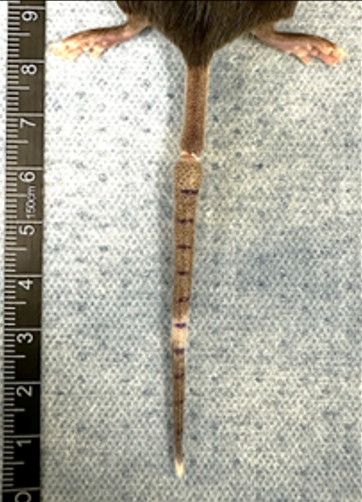

Supplement: Supplementary file 4 — Source data Fig. 2 [file 44321_2025_286_MOESM4_ESM.zip › Figure 2/2A/HSFD Sham.tif]

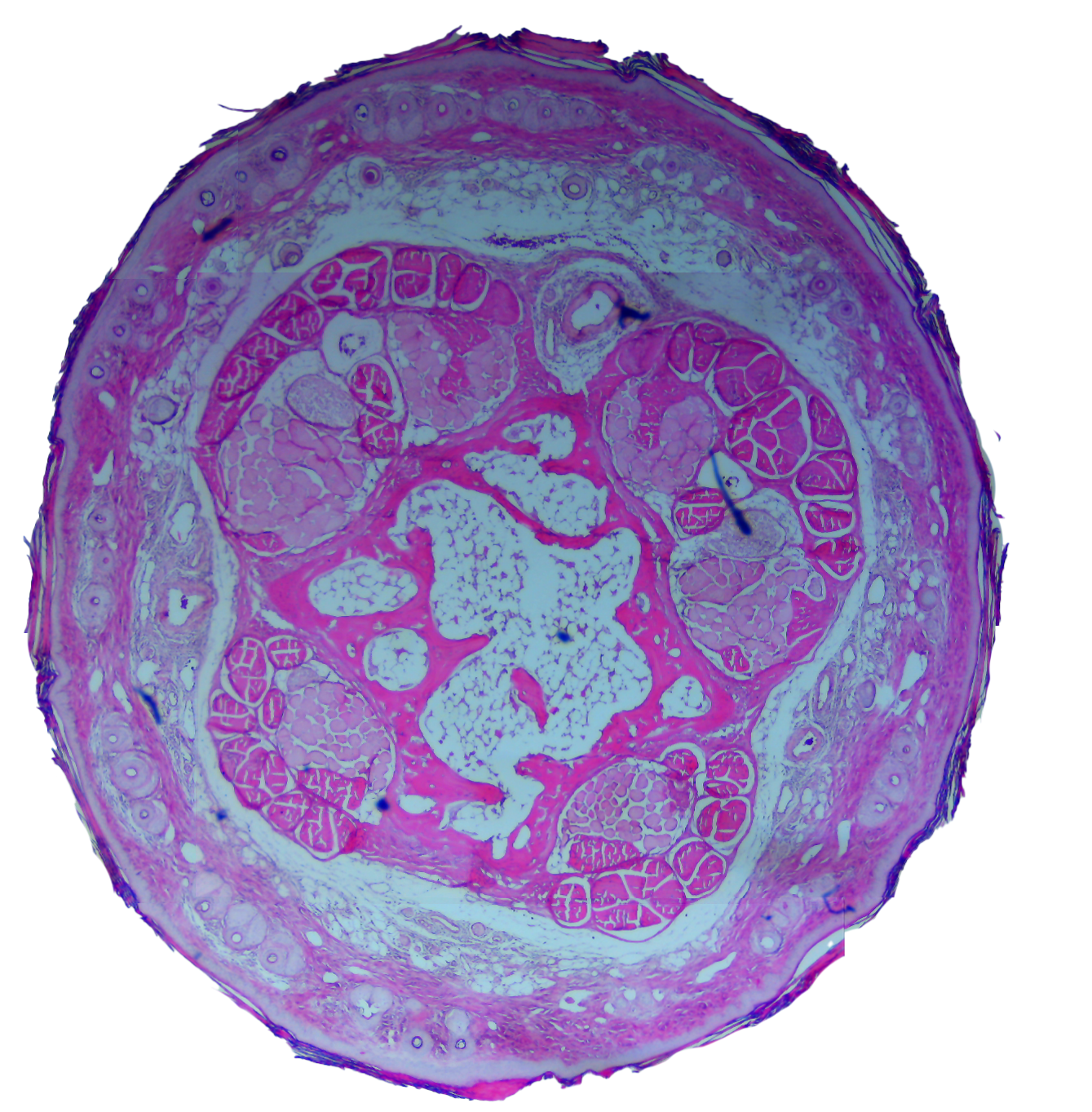

Supplement: Supplementary file 4 — Source data Fig. 2 [file 44321_2025_286_MOESM4_ESM.zip › Figure 2/2B/CD LE.tif]

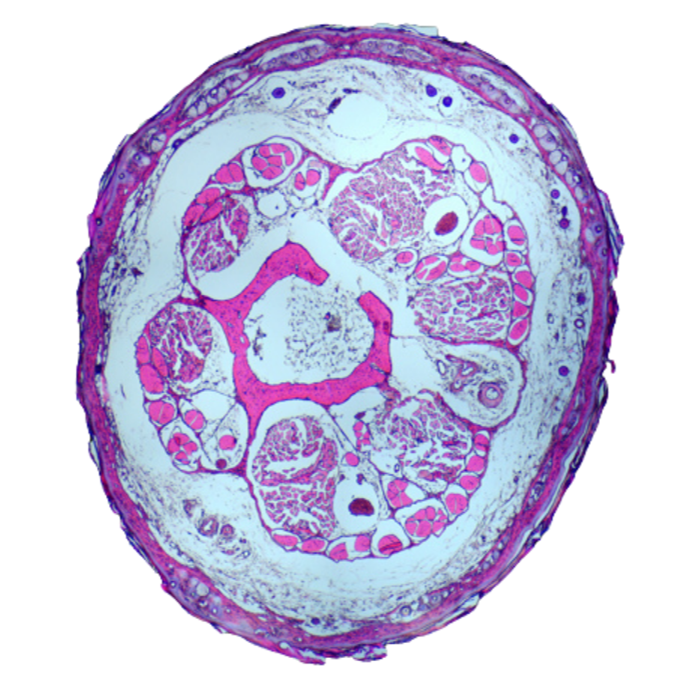

Supplement: Supplementary file 4 — Source data Fig. 2 [file 44321_2025_286_MOESM4_ESM.zip › Figure 2/2B/CD Sham.tif]

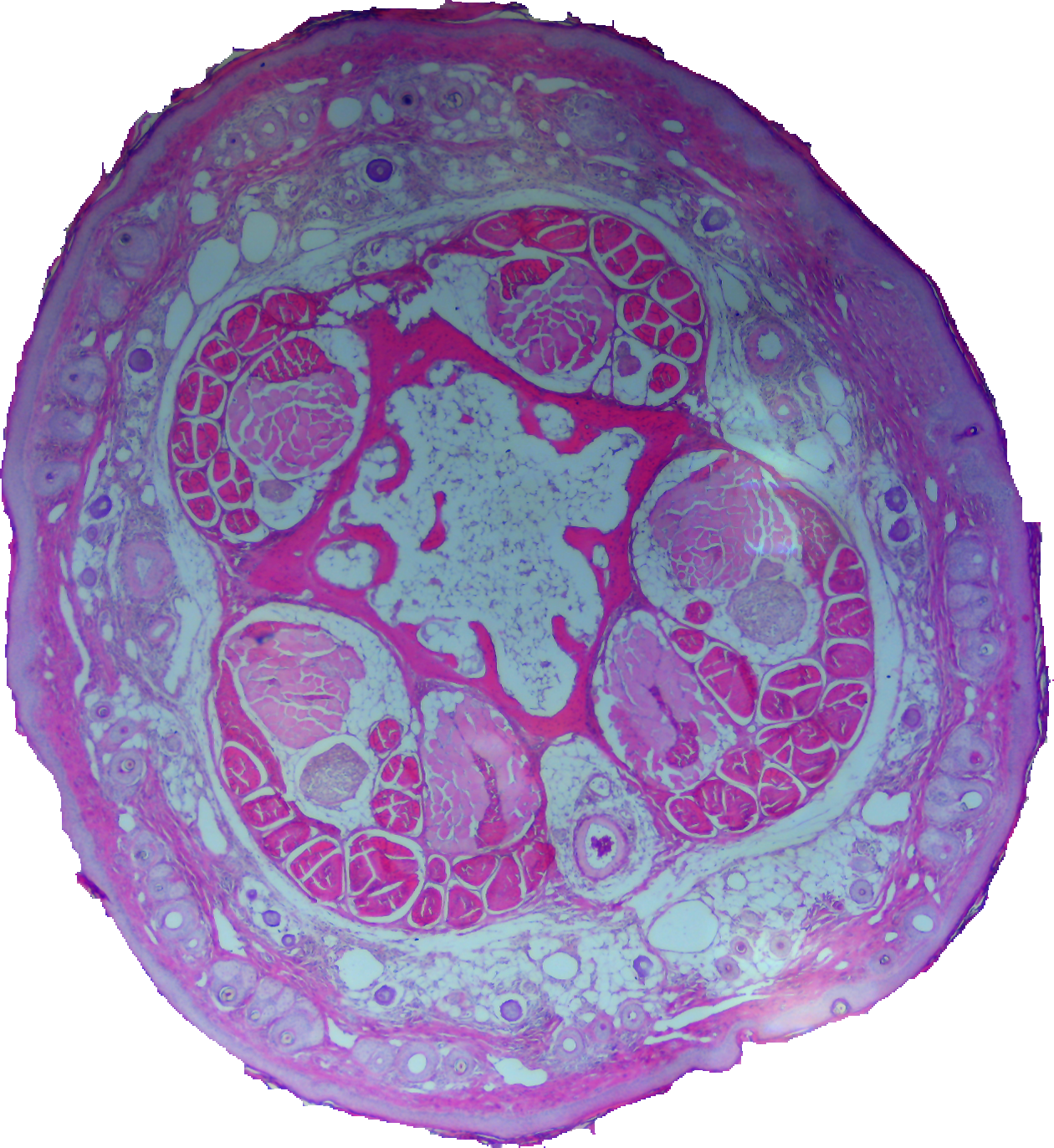

Supplement: Supplementary file 4 — Source data Fig. 2 [file 44321_2025_286_MOESM4_ESM.zip › Figure 2/2B/HFD LE.tif]

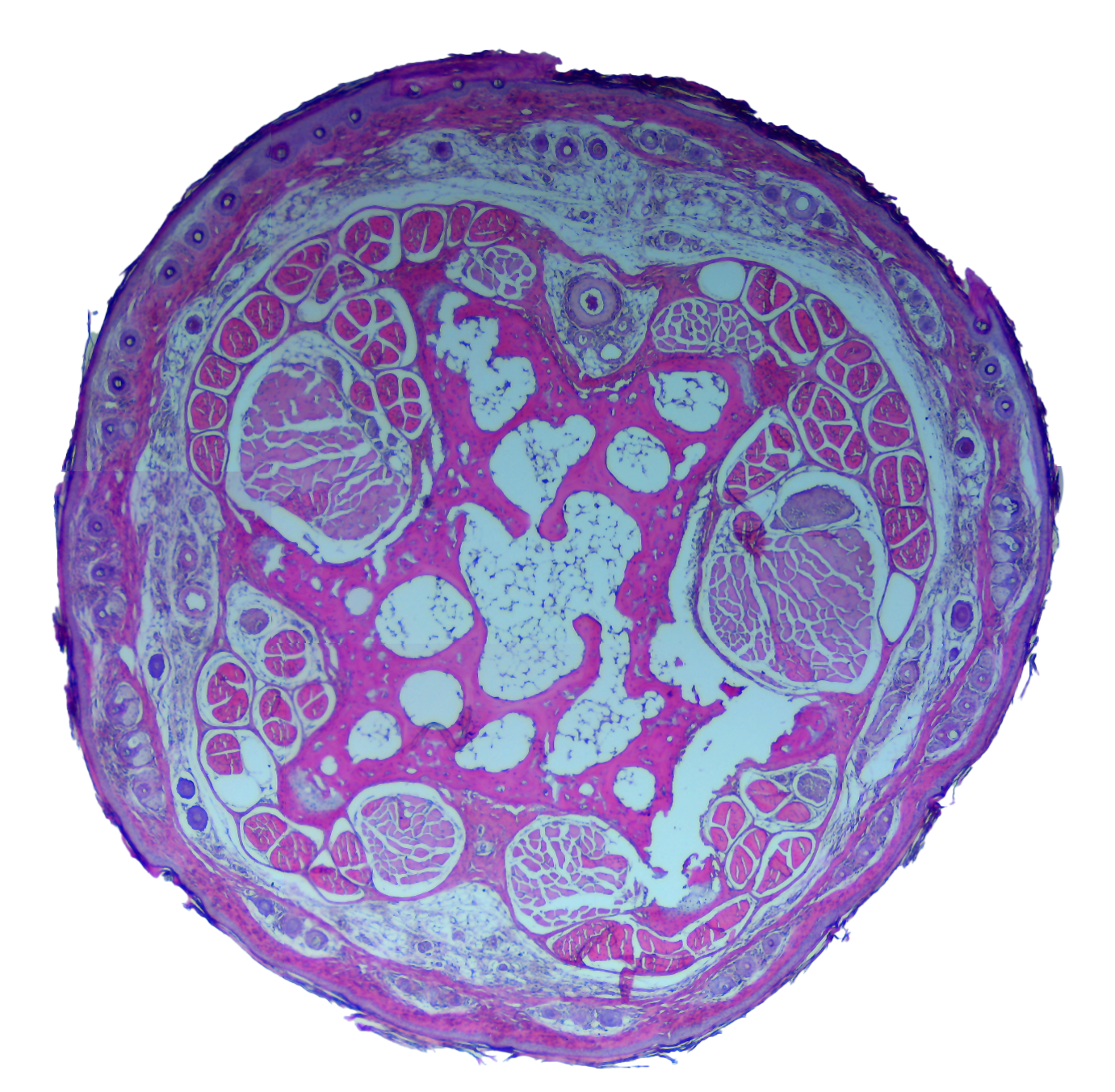

Supplement: Supplementary file 4 — Source data Fig. 2 [file 44321_2025_286_MOESM4_ESM.zip › Figure 2/2B/HFD Sham.tif]

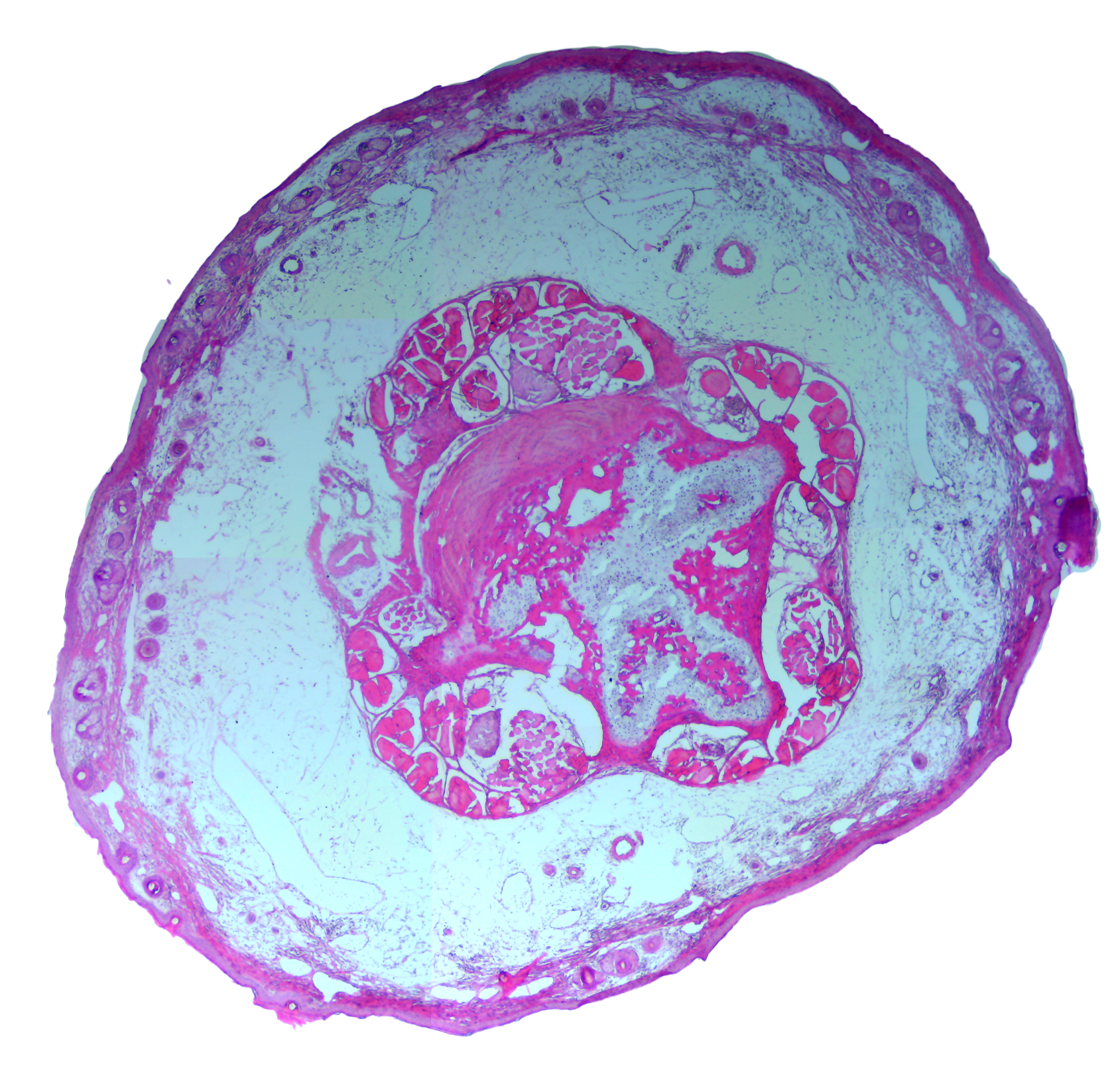

Supplement: Supplementary file 4 — Source data Fig. 2 [file 44321_2025_286_MOESM4_ESM.zip › Figure 2/2B/HSFD LE.tif]

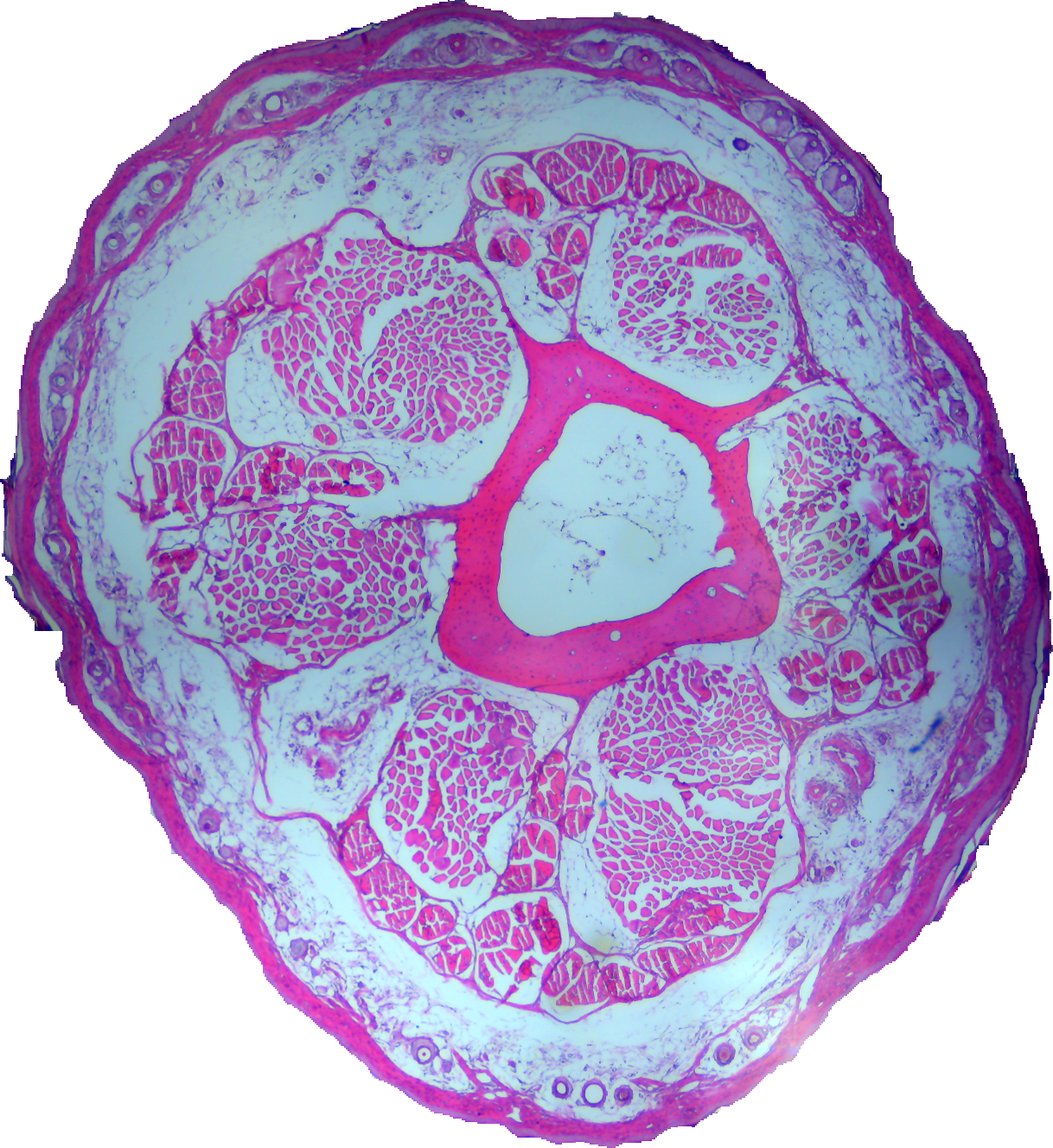

Supplement: Supplementary file 4 — Source data Fig. 2 [file 44321_2025_286_MOESM4_ESM.zip › Figure 2/2B/HSFD Sham.tif]

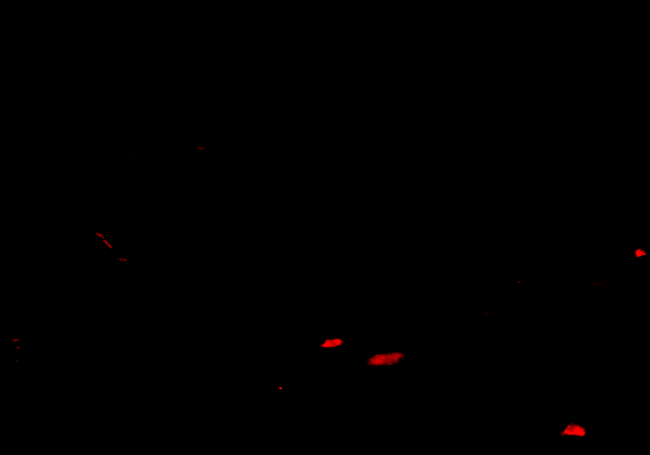

Supplement: Supplementary file 4 — Source data Fig. 2 [file 44321_2025_286_MOESM4_ESM.zip › Figure 2/2D/CD/CHOP_CD_LE_CHOP.tif]

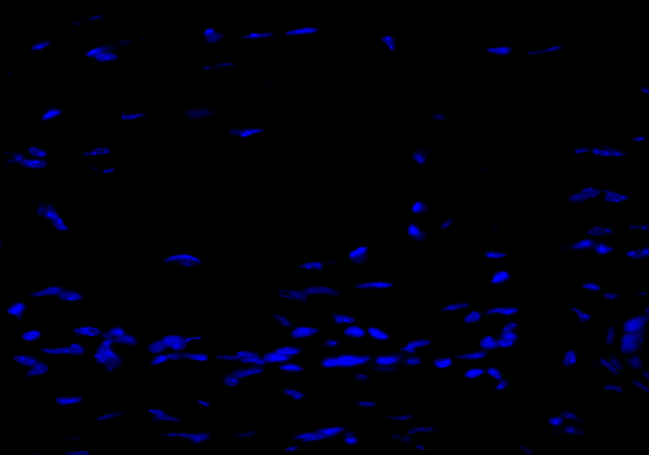

Supplement: Supplementary file 4 — Source data Fig. 2 [file 44321_2025_286_MOESM4_ESM.zip › Figure 2/2D/CD/CHOP_CD_LE_DAPI.tif]

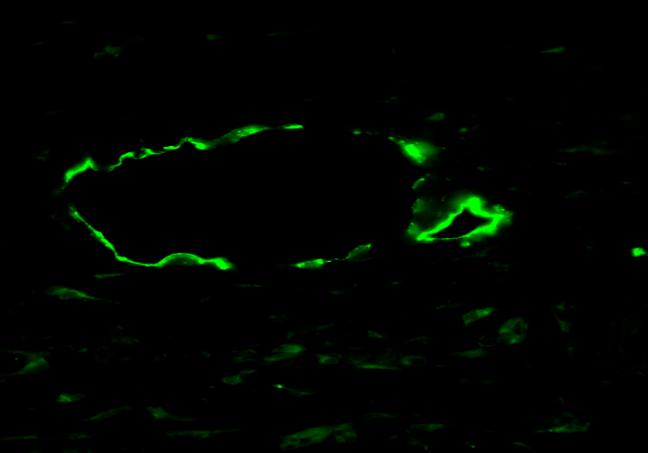

Supplement: Supplementary file 4 — Source data Fig. 2 [file 44321_2025_286_MOESM4_ESM.zip › Figure 2/2D/CD/CHOP_CD_LE_LYVE-1.tif]

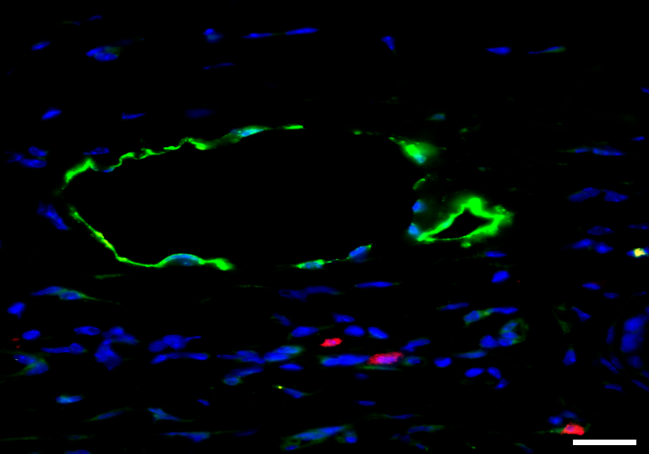

Supplement: Supplementary file 4 — Source data Fig. 2 [file 44321_2025_286_MOESM4_ESM.zip › Figure 2/2D/CD/CHOP_CD_LE_Merged.tif]

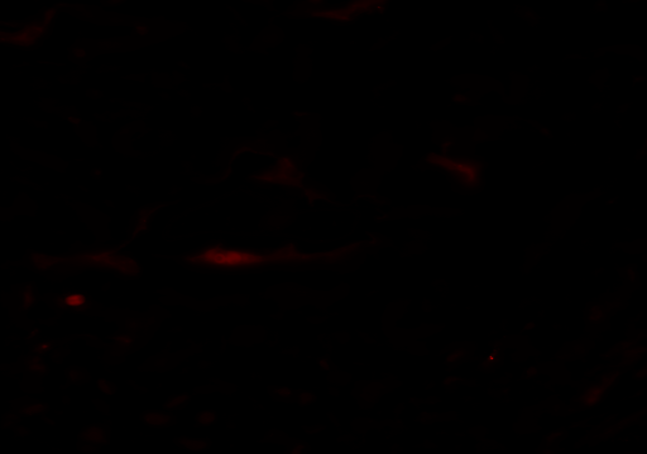

Supplement: Supplementary file 4 — Source data Fig. 2 [file 44321_2025_286_MOESM4_ESM.zip › Figure 2/2D/CD/CHOP_CD_Sham_CHOP.tif]

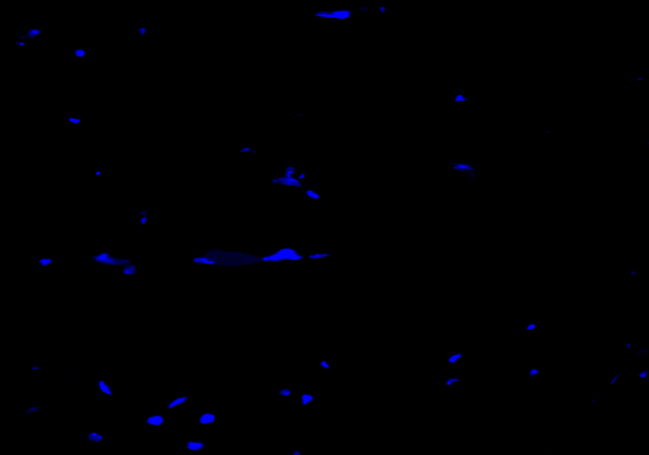

Supplement: Supplementary file 4 — Source data Fig. 2 [file 44321_2025_286_MOESM4_ESM.zip › Figure 2/2D/CD/CHOP_CD_Sham_DAPI.tif]

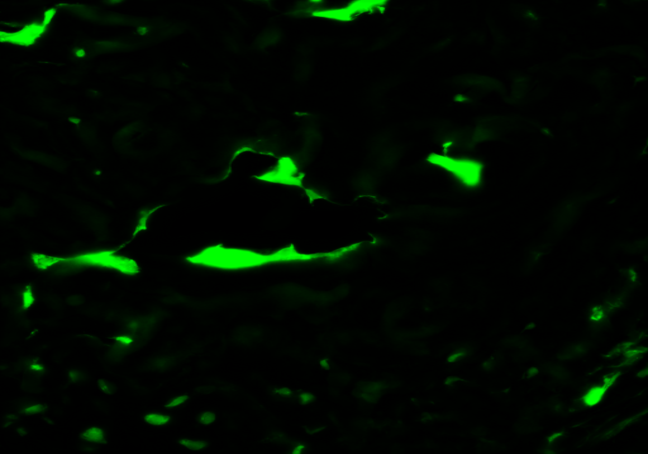

Supplement: Supplementary file 4 — Source data Fig. 2 [file 44321_2025_286_MOESM4_ESM.zip › Figure 2/2D/CD/CHOP_CD_Sham_LYVE-1.tif]

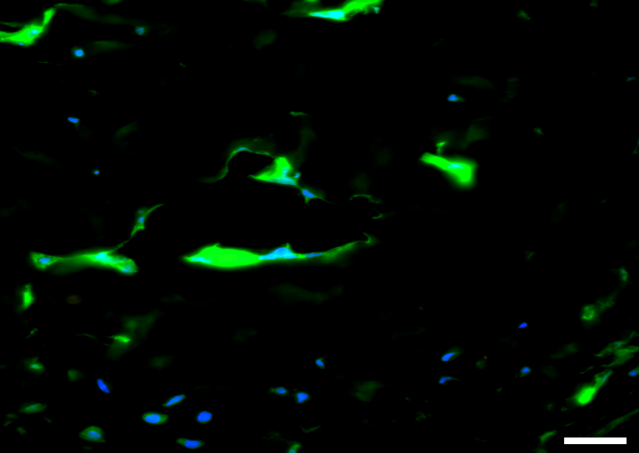

Supplement: Supplementary file 4 — Source data Fig. 2 [file 44321_2025_286_MOESM4_ESM.zip › Figure 2/2D/CD/CHOP_CD_Sham_Merged.tif]

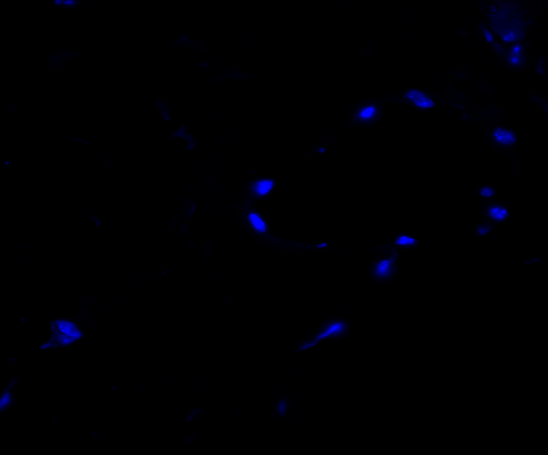

Supplement: Supplementary file 4 — Source data Fig. 2 [file 44321_2025_286_MOESM4_ESM.zip › Figure 2/2D/CD/sXBP-1_CD_LE_DAPI.tif]

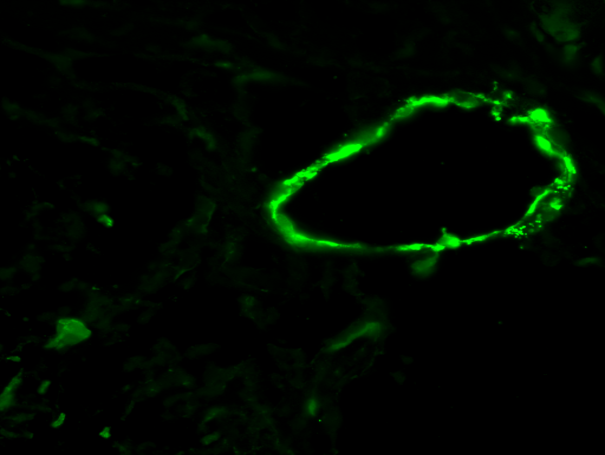

Supplement: Supplementary file 4 — Source data Fig. 2 [file 44321_2025_286_MOESM4_ESM.zip › Figure 2/2D/CD/sXBP-1_CD_LE_LYVE-1.tif]

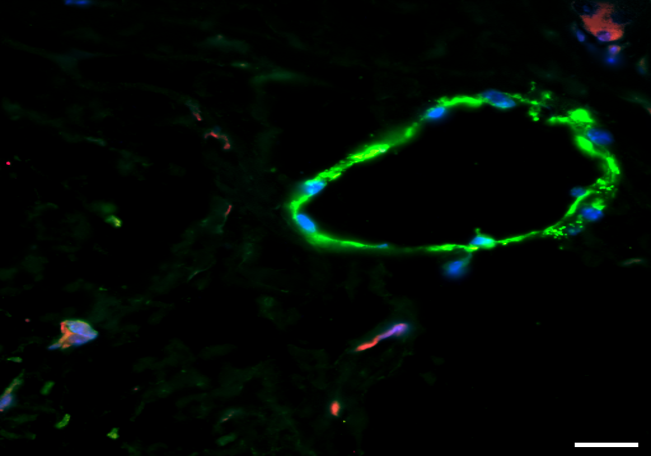

Supplement: Supplementary file 4 — Source data Fig. 2 [file 44321_2025_286_MOESM4_ESM.zip › Figure 2/2D/CD/sXBP-1_CD_LE_Merged.tif]

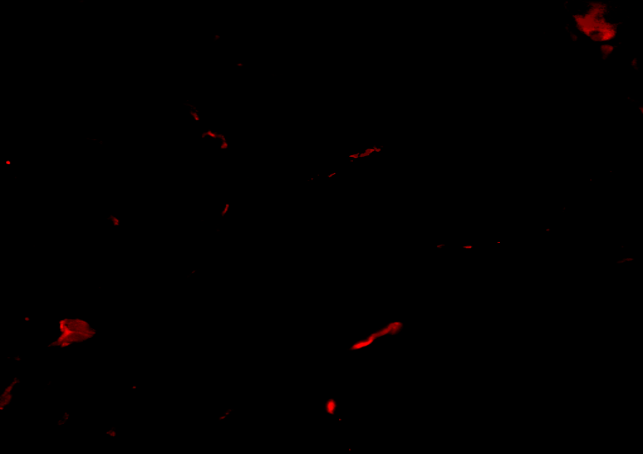

Supplement: Supplementary file 4 — Source data Fig. 2 [file 44321_2025_286_MOESM4_ESM.zip › Figure 2/2D/CD/sXBP-1_CD_LE_sXBP-1.tif]

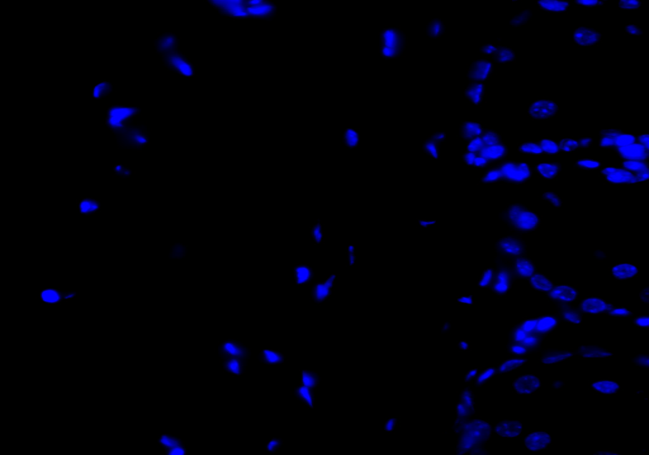

Supplement: Supplementary file 4 — Source data Fig. 2 [file 44321_2025_286_MOESM4_ESM.zip › Figure 2/2D/CD/sXBP-1_CD_Sham_DAPI.tif]

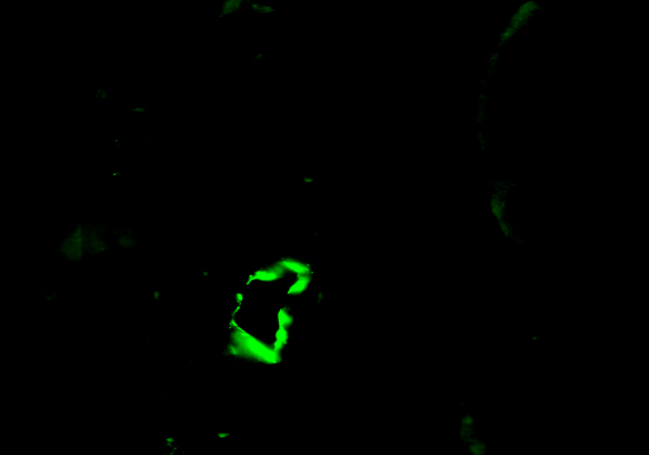

Supplement: Supplementary file 4 — Source data Fig. 2 [file 44321_2025_286_MOESM4_ESM.zip › Figure 2/2D/CD/sXBP-1_CD_Sham_LYVE-1.tif]

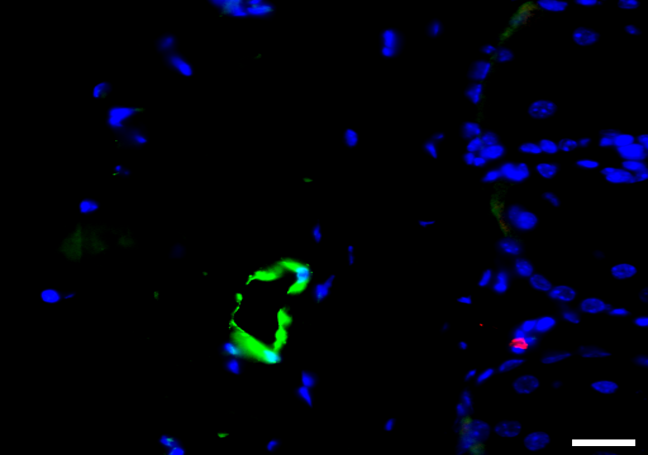

Supplement: Supplementary file 4 — Source data Fig. 2 [file 44321_2025_286_MOESM4_ESM.zip › Figure 2/2D/CD/sXBP-1_CD_Sham_Merged.tif]

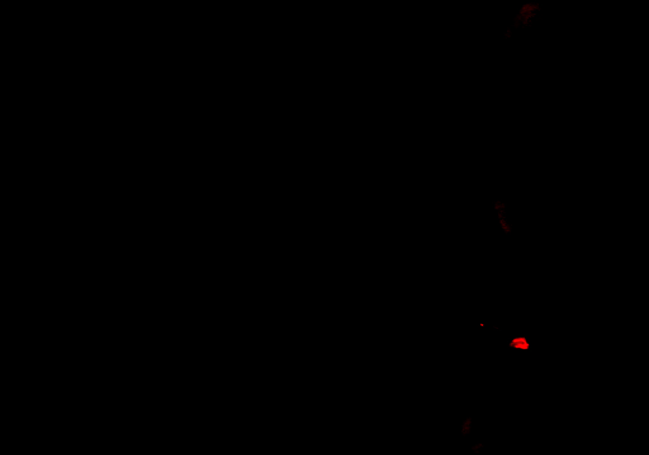

Supplement: Supplementary file 4 — Source data Fig. 2 [file 44321_2025_286_MOESM4_ESM.zip › Figure 2/2D/CD/sXBP-1_CD_Sham_sXBP-1.tif]

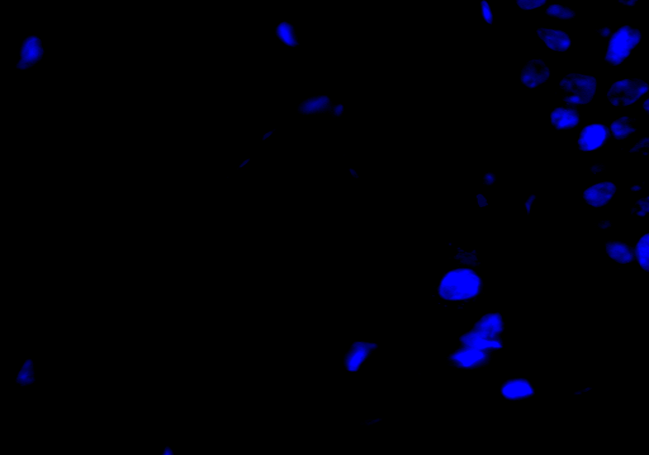

Supplement: Supplementary file 4 — Source data Fig. 2 [file 44321_2025_286_MOESM4_ESM.zip › Figure 2/2D/CD/TUNEL_CD_LE_DAPI.tif]

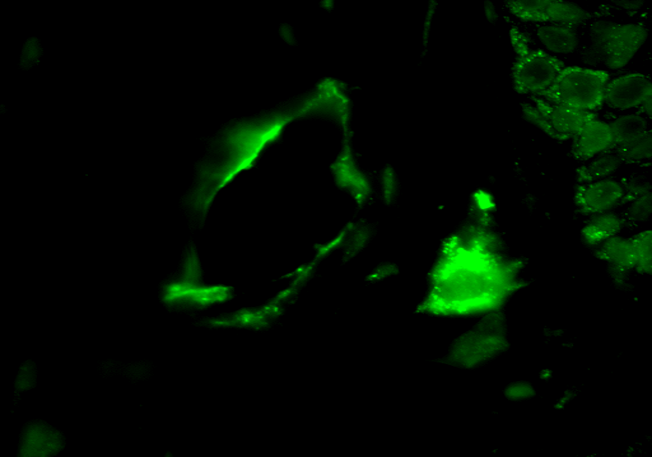

Supplement: Supplementary file 4 — Source data Fig. 2 [file 44321_2025_286_MOESM4_ESM.zip › Figure 2/2D/CD/TUNEL_CD_LE_LEVY-1.tif]

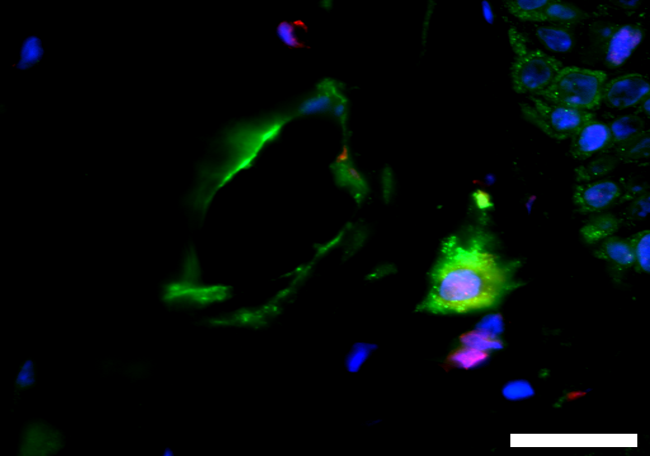

Supplement: Supplementary file 4 — Source data Fig. 2 [file 44321_2025_286_MOESM4_ESM.zip › Figure 2/2D/CD/TUNEL_CD_LE_Merged.tif]

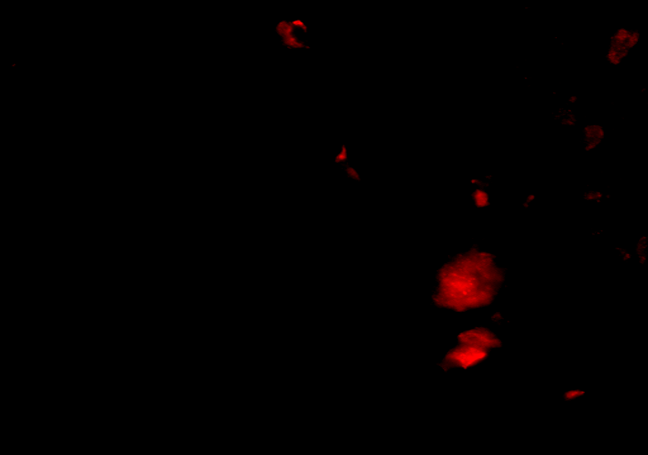

Supplement: Supplementary file 4 — Source data Fig. 2 [file 44321_2025_286_MOESM4_ESM.zip › Figure 2/2D/CD/TUNEL_CD_LE_TUNEL.tif]

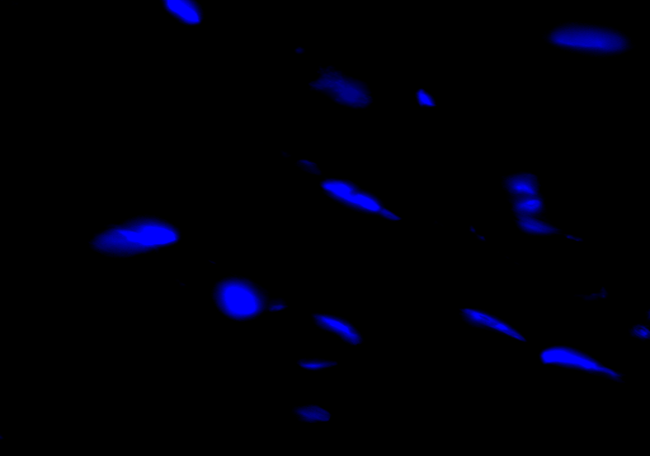

Supplement: Supplementary file 4 — Source data Fig. 2 [file 44321_2025_286_MOESM4_ESM.zip › Figure 2/2D/CD/TUNEL_CD_Sham_DAPI.tif]

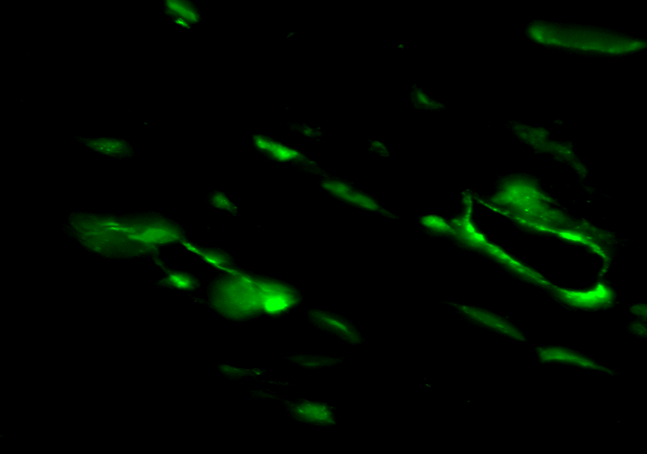

Supplement: Supplementary file 4 — Source data Fig. 2 [file 44321_2025_286_MOESM4_ESM.zip › Figure 2/2D/CD/TUNEL_CD_Sham_LYVE-1.tif]

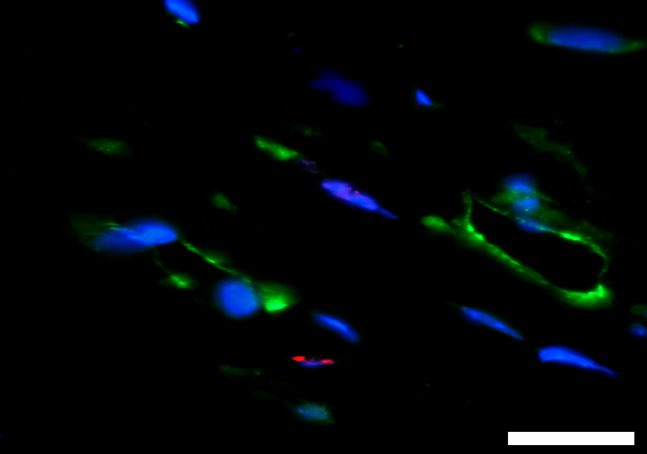

Supplement: Supplementary file 4 — Source data Fig. 2 [file 44321_2025_286_MOESM4_ESM.zip › Figure 2/2D/CD/TUNEL_CD_Sham_Merged.tif]

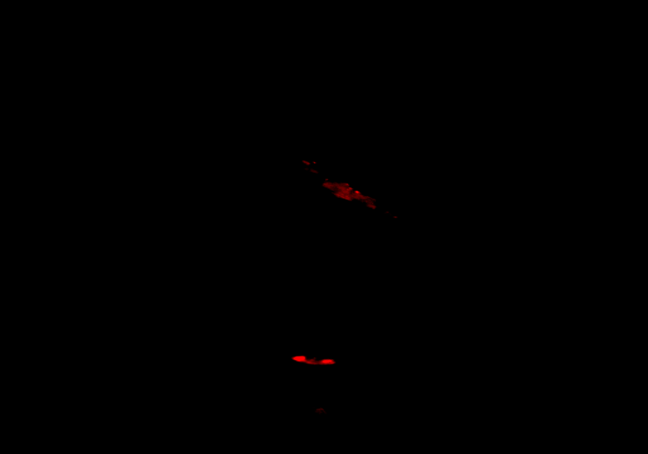

Supplement: Supplementary file 4 — Source data Fig. 2 [file 44321_2025_286_MOESM4_ESM.zip › Figure 2/2D/CD/TUNEL_CD_Sham_TUNEL.tif]

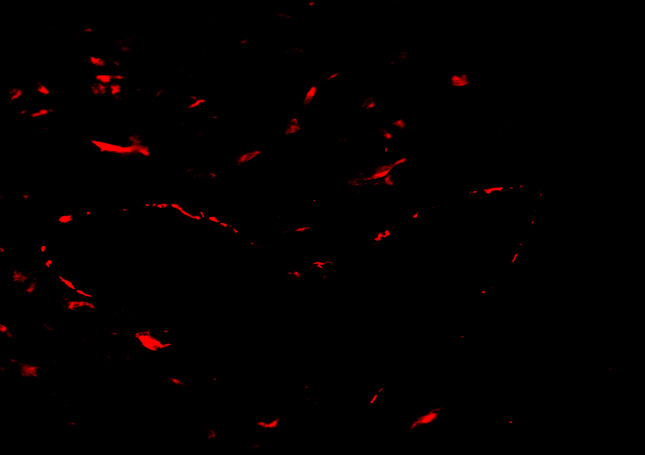

Supplement: Supplementary file 4 — Source data Fig. 2 [file 44321_2025_286_MOESM4_ESM.zip › Figure 2/2D/HSFD/CHOP_HSFD_LE_CHOP.tif]

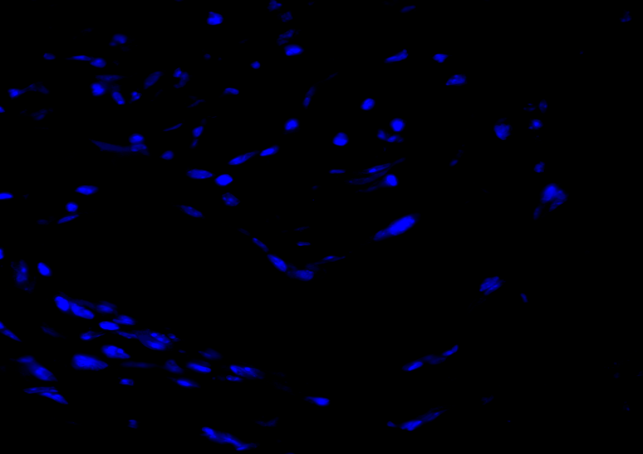

Supplement: Supplementary file 4 — Source data Fig. 2 [file 44321_2025_286_MOESM4_ESM.zip › Figure 2/2D/HSFD/CHOP_HSFD_LE_DAPI.tif]

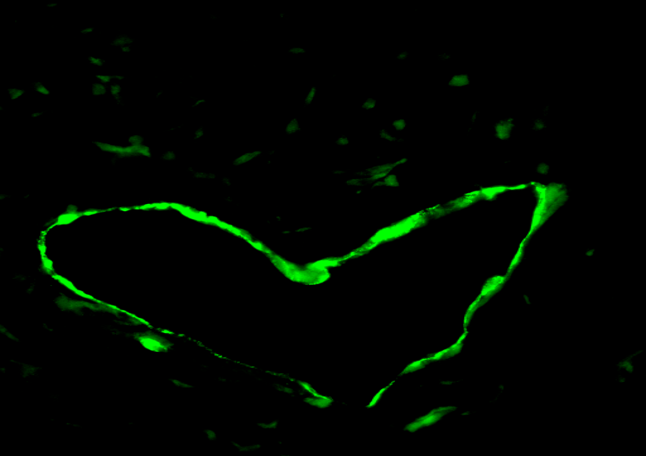

Supplement: Supplementary file 4 — Source data Fig. 2 [file 44321_2025_286_MOESM4_ESM.zip › Figure 2/2D/HSFD/CHOP_HSFD_LE_LYVE-1.tif]

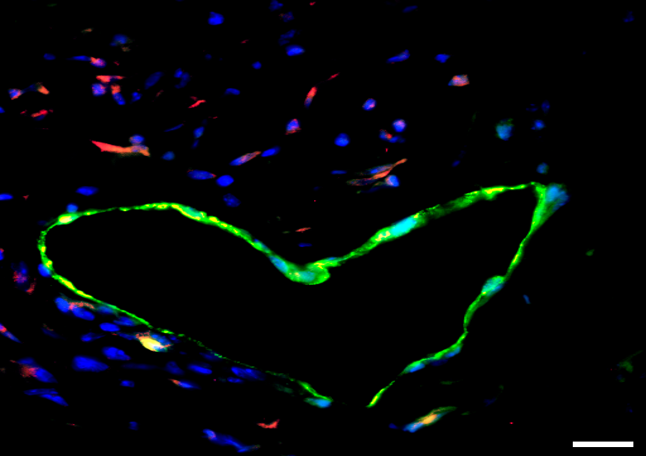

Supplement: Supplementary file 4 — Source data Fig. 2 [file 44321_2025_286_MOESM4_ESM.zip › Figure 2/2D/HSFD/CHOP_HSFD_LE_Merged.tif]

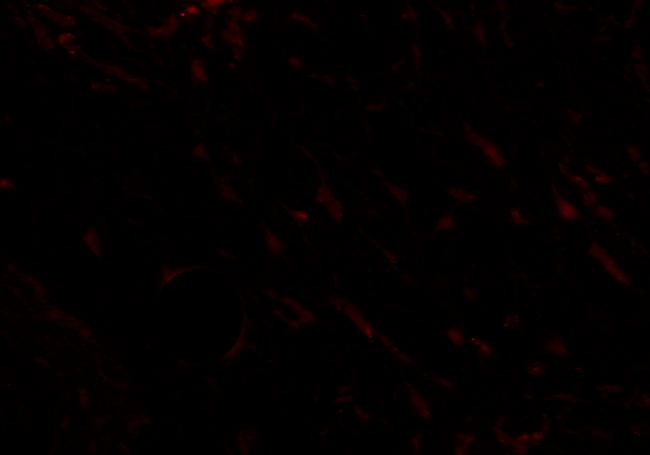

Supplement: Supplementary file 4 — Source data Fig. 2 [file 44321_2025_286_MOESM4_ESM.zip › Figure 2/2D/HSFD/CHOP_HSFD_Sham_CHOP.tif]

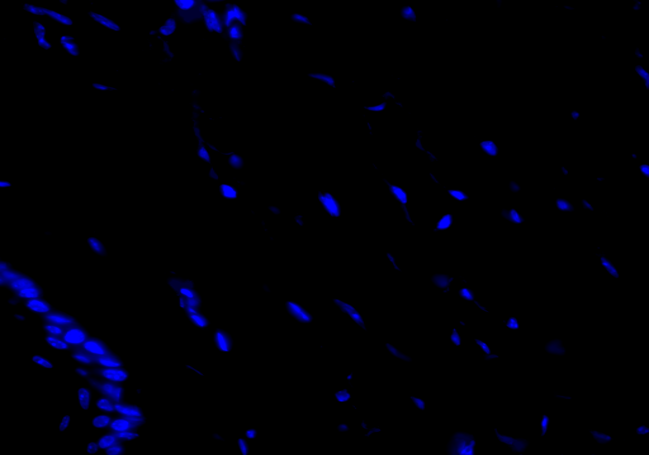

Supplement: Supplementary file 4 — Source data Fig. 2 [file 44321_2025_286_MOESM4_ESM.zip › Figure 2/2D/HSFD/CHOP_HSFD_Sham_DAPI.tif]

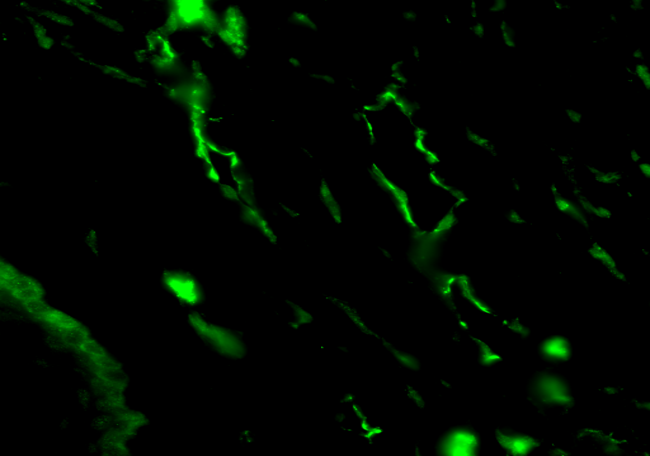

Supplement: Supplementary file 4 — Source data Fig. 2 [file 44321_2025_286_MOESM4_ESM.zip › Figure 2/2D/HSFD/CHOP_HSFD_Sham_LYVE-1.tif]

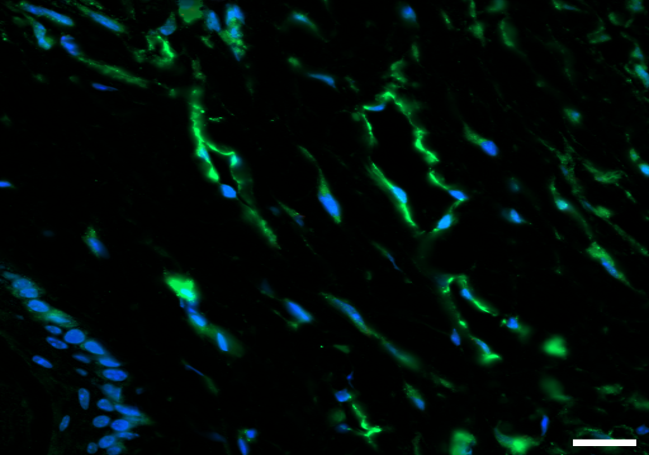

Supplement: Supplementary file 4 — Source data Fig. 2 [file 44321_2025_286_MOESM4_ESM.zip › Figure 2/2D/HSFD/CHOP_HSFD_Sham_Merged.tif]

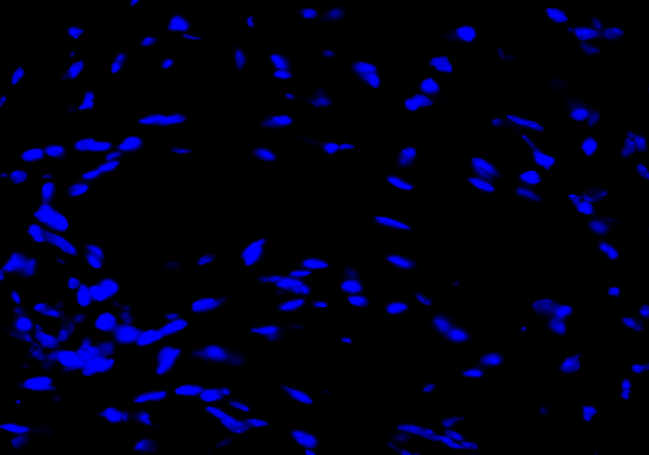

Supplement: Supplementary file 4 — Source data Fig. 2 [file 44321_2025_286_MOESM4_ESM.zip › Figure 2/2D/HSFD/sXBP-1_HSFD_LE_DAPI.tif]

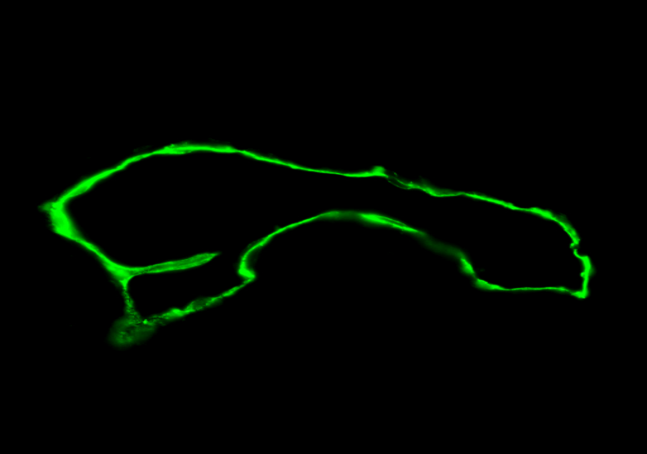

Supplement: Supplementary file 4 — Source data Fig. 2 [file 44321_2025_286_MOESM4_ESM.zip › Figure 2/2D/HSFD/sXBP-1_HSFD_LE_LEVY-1.tif]

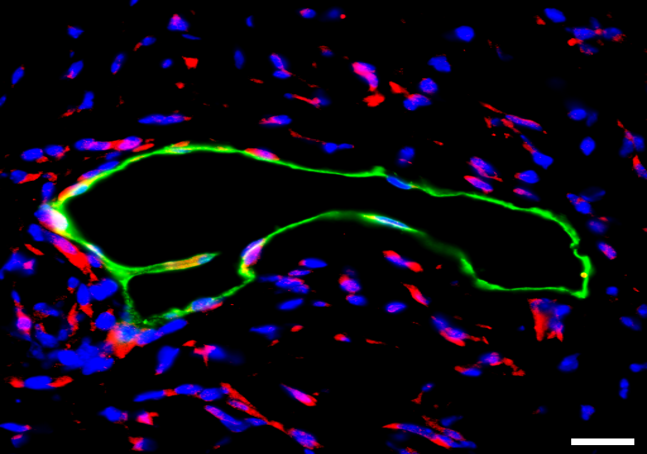

Supplement: Supplementary file 4 — Source data Fig. 2 [file 44321_2025_286_MOESM4_ESM.zip › Figure 2/2D/HSFD/sXBP-1_HSFD_LE_Merged.tif]

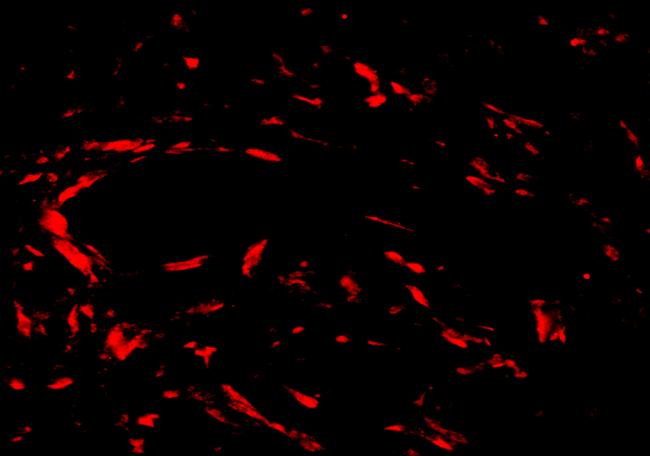

Supplement: Supplementary file 4 — Source data Fig. 2 [file 44321_2025_286_MOESM4_ESM.zip › Figure 2/2D/HSFD/sXBP-1_HSFD_LE_sXBP-1.tif]

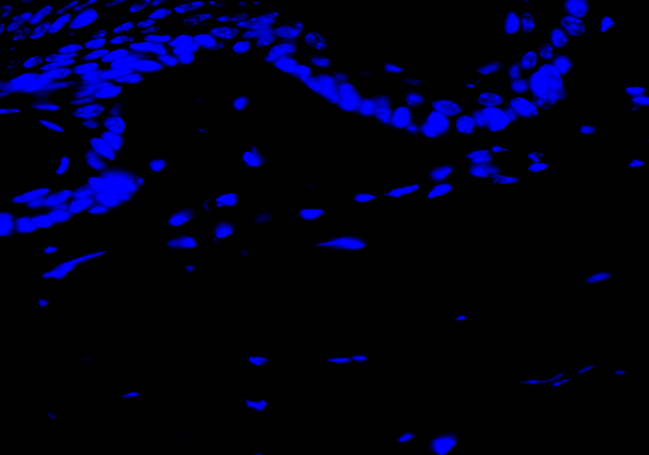

Supplement: Supplementary file 4 — Source data Fig. 2 [file 44321_2025_286_MOESM4_ESM.zip › Figure 2/2D/HSFD/sXBP-1_HSFD_Sham_DAPI.tif]

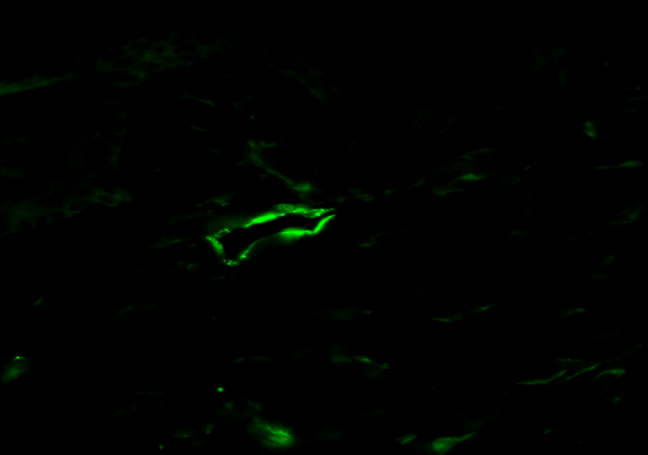

Supplement: Supplementary file 4 — Source data Fig. 2 [file 44321_2025_286_MOESM4_ESM.zip › Figure 2/2D/HSFD/sXBP-1_HSFD_Sham_LYVE-1.tif]

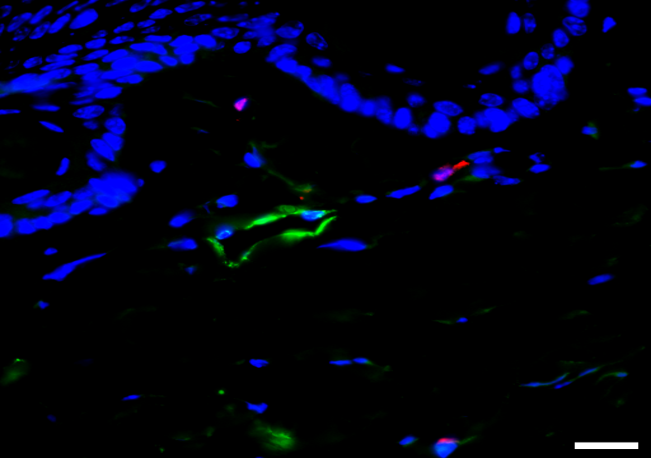

Supplement: Supplementary file 4 — Source data Fig. 2 [file 44321_2025_286_MOESM4_ESM.zip › Figure 2/2D/HSFD/sXBP-1_HSFD_Sham_Merged.tif]

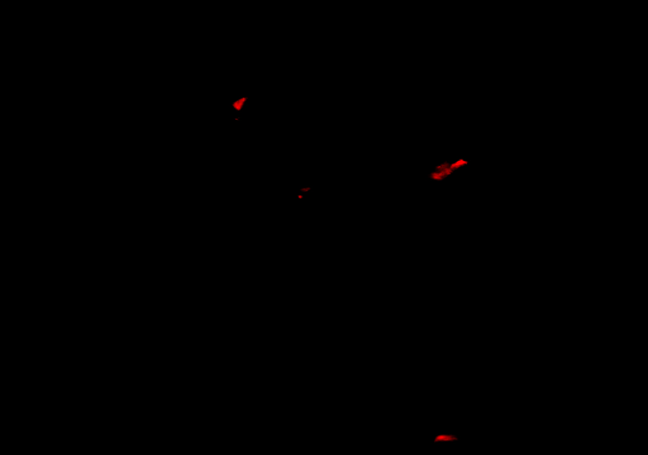

Supplement: Supplementary file 4 — Source data Fig. 2 [file 44321_2025_286_MOESM4_ESM.zip › Figure 2/2D/HSFD/sXBP-1_HSFD_Sham_sXBP-1.tif]

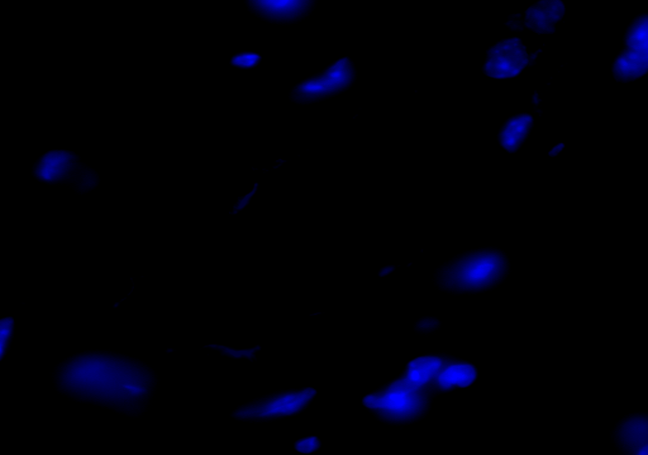

Supplement: Supplementary file 4 — Source data Fig. 2 [file 44321_2025_286_MOESM4_ESM.zip › Figure 2/2D/HSFD/TUNEL_HSFD_LE_DAPI.tif]

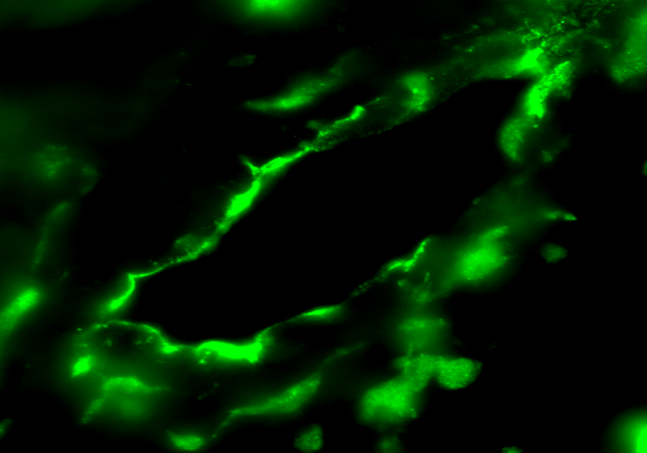

Supplement: Supplementary file 4 — Source data Fig. 2 [file 44321_2025_286_MOESM4_ESM.zip › Figure 2/2D/HSFD/TUNEL_HSFD_LE_LYVE-1.tif]

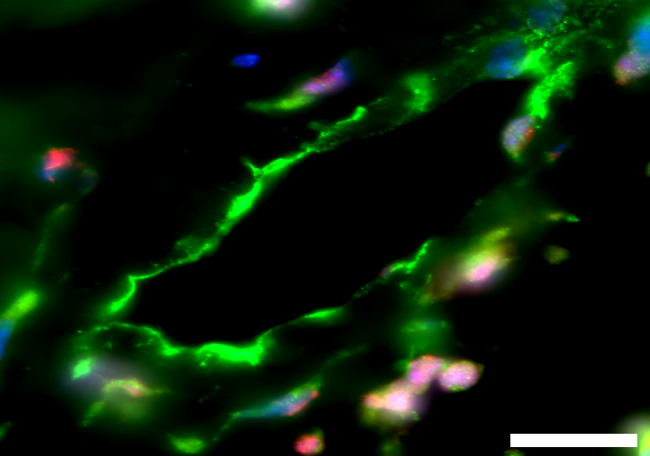

Supplement: Supplementary file 4 — Source data Fig. 2 [file 44321_2025_286_MOESM4_ESM.zip › Figure 2/2D/HSFD/TUNEL_HSFD_LE_Merged.tif]

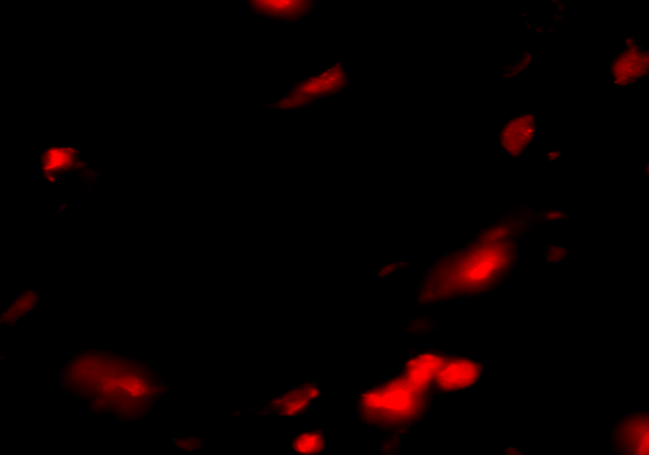

Supplement: Supplementary file 4 — Source data Fig. 2 [file 44321_2025_286_MOESM4_ESM.zip › Figure 2/2D/HSFD/TUNEL_HSFD_LE_TUNEL.tif]

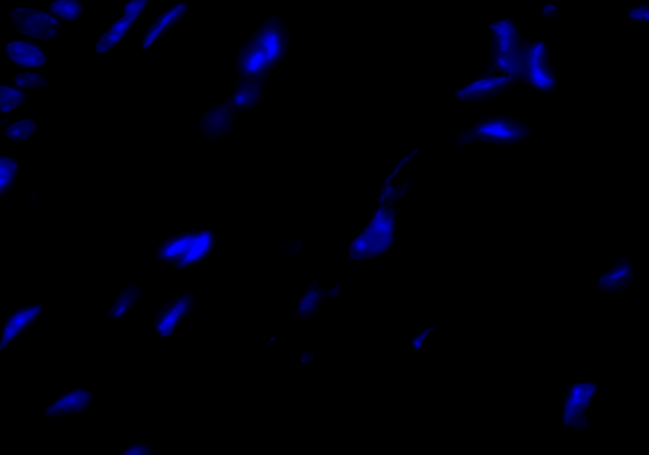

Supplement: Supplementary file 4 — Source data Fig. 2 [file 44321_2025_286_MOESM4_ESM.zip › Figure 2/2D/HSFD/TUNEL_HSFD_Sham_DAPI.tif]

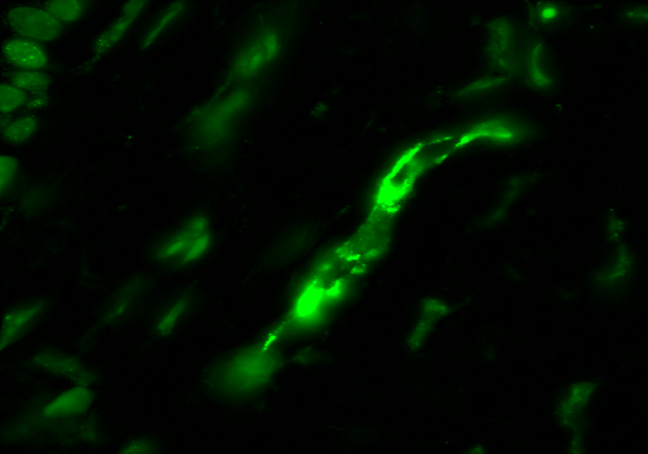

Supplement: Supplementary file 4 — Source data Fig. 2 [file 44321_2025_286_MOESM4_ESM.zip › Figure 2/2D/HSFD/TUNEL_HSFD_Sham_LYVE-1.tif]

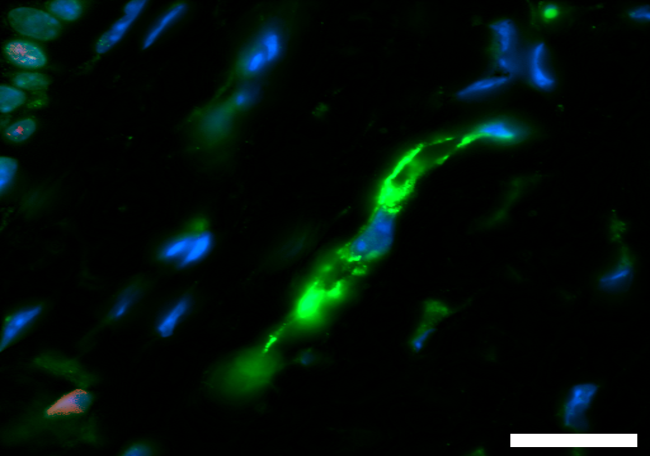

Supplement: Supplementary file 4 — Source data Fig. 2 [file 44321_2025_286_MOESM4_ESM.zip › Figure 2/2D/HSFD/TUNEL_HSFD_Sham_Merged.tif]

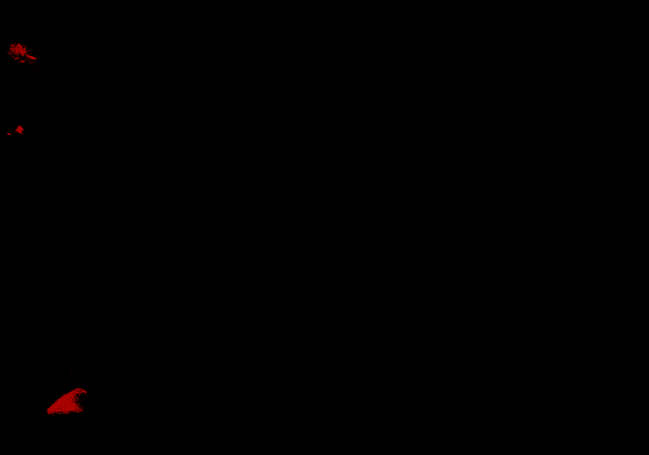

Supplement: Supplementary file 4 — Source data Fig. 2 [file 44321_2025_286_MOESM4_ESM.zip › Figure 2/2D/HSFD/TUNEL_HSFD_Sham_TUNEL.tif]

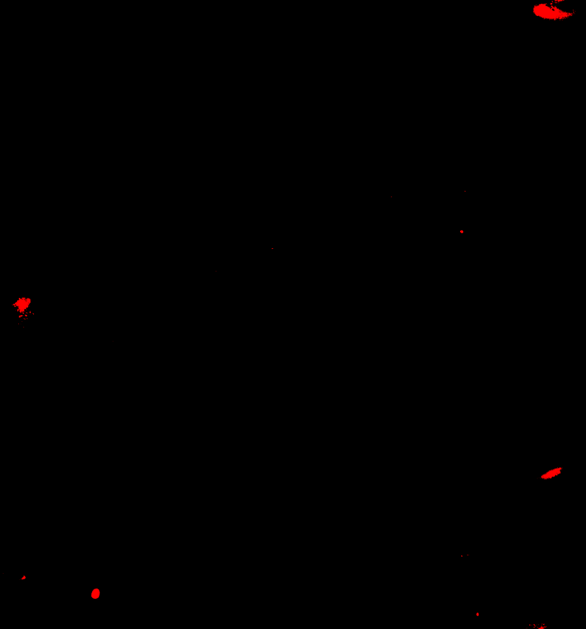

Supplement: Supplementary file 6 — Source data Fig. 4 [file 44321_2025_286_MOESM6_ESM.zip › Figure 4/4A/8-OHdG_CD_LE_8-OHdG.tif]

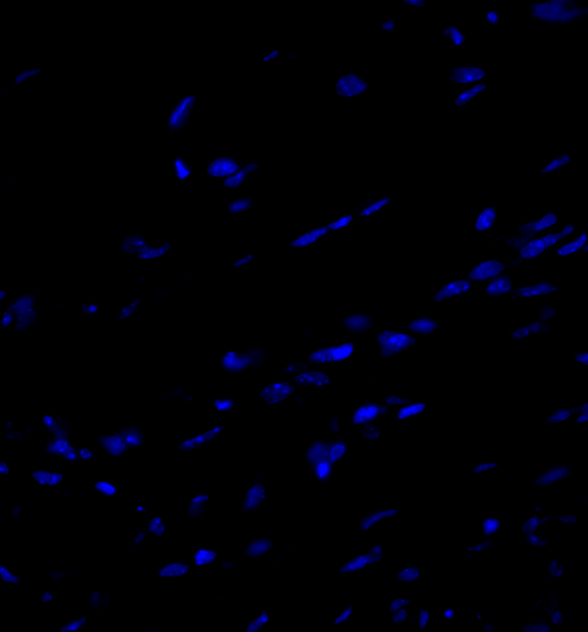

Supplement: Supplementary file 6 — Source data Fig. 4 [file 44321_2025_286_MOESM6_ESM.zip › Figure 4/4A/8-OHdG_CD_LE_DAPI.tif]

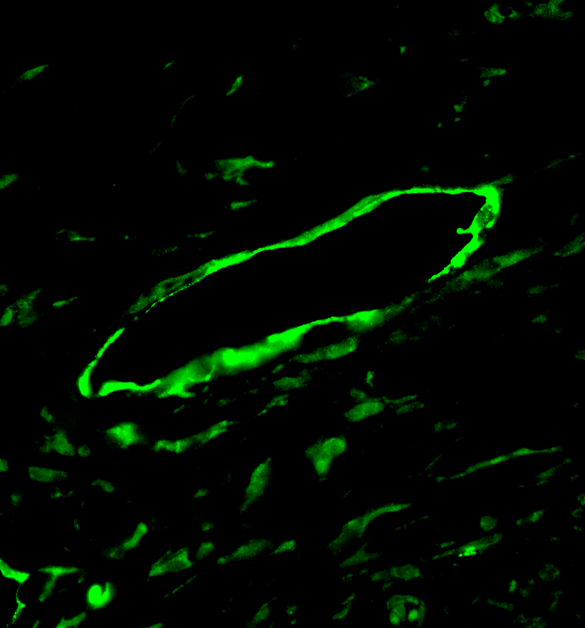

Supplement: Supplementary file 6 — Source data Fig. 4 [file 44321_2025_286_MOESM6_ESM.zip › Figure 4/4A/8-OHdG_CD_LE_LYVE-1.tif]

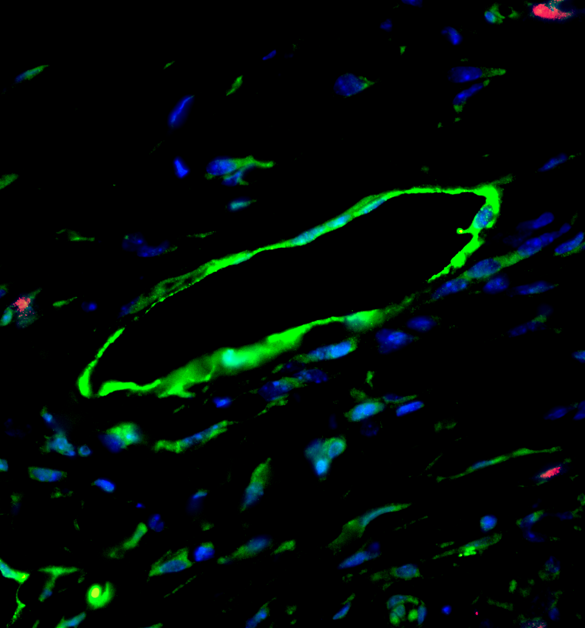

Supplement: Supplementary file 6 — Source data Fig. 4 [file 44321_2025_286_MOESM6_ESM.zip › Figure 4/4A/8-OHdG_CD_LE_Merged.tif]

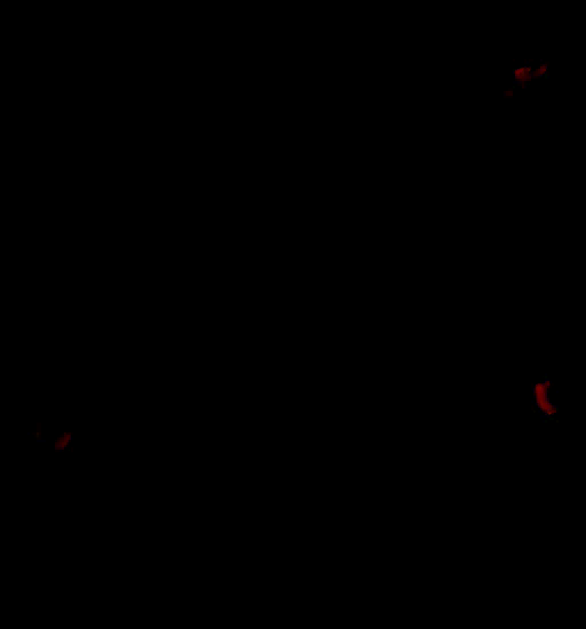

Supplement: Supplementary file 6 — Source data Fig. 4 [file 44321_2025_286_MOESM6_ESM.zip › Figure 4/4A/8-OHdG_CD_Sham_8-OHdG.tif]

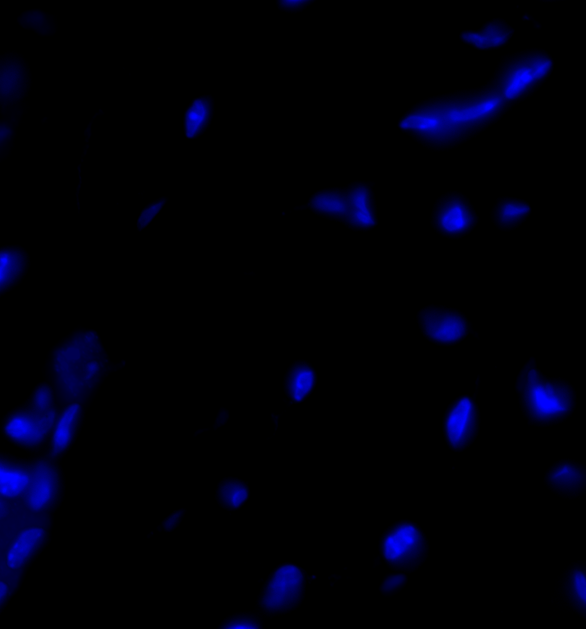

Supplement: Supplementary file 6 — Source data Fig. 4 [file 44321_2025_286_MOESM6_ESM.zip › Figure 4/4A/8-OHdG_CD_Sham_DAPI.tif]

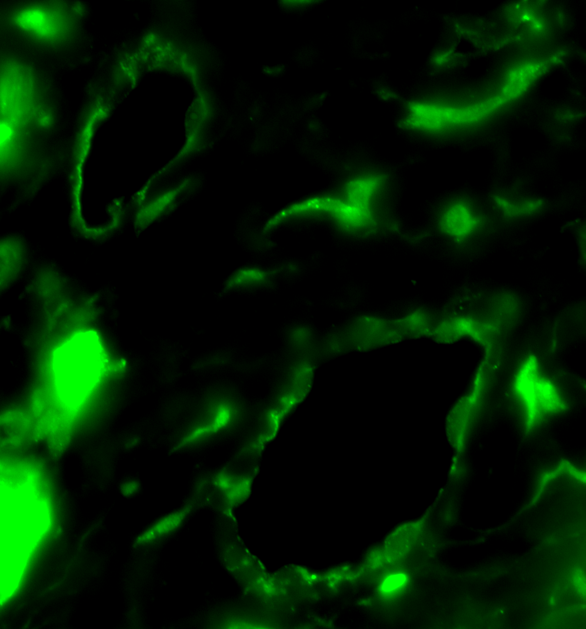

Supplement: Supplementary file 6 — Source data Fig. 4 [file 44321_2025_286_MOESM6_ESM.zip › Figure 4/4A/8-OHdG_CD_Sham_LYVE-1.tif]

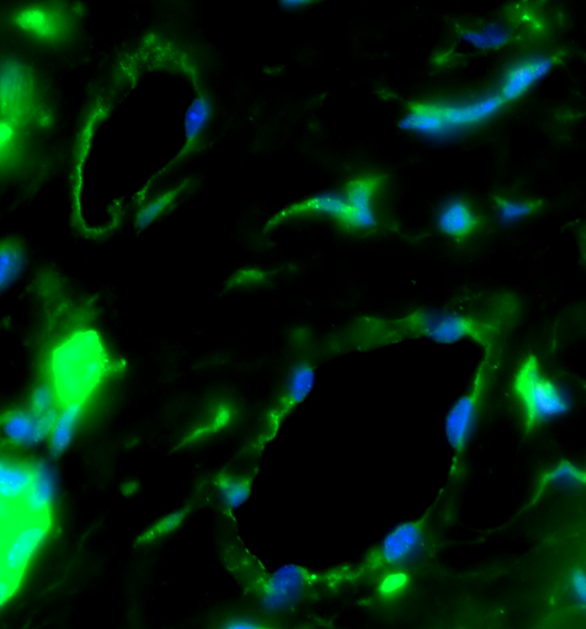

Supplement: Supplementary file 6 — Source data Fig. 4 [file 44321_2025_286_MOESM6_ESM.zip › Figure 4/4A/8-OHdG_CD_Sham_Merged.tif]

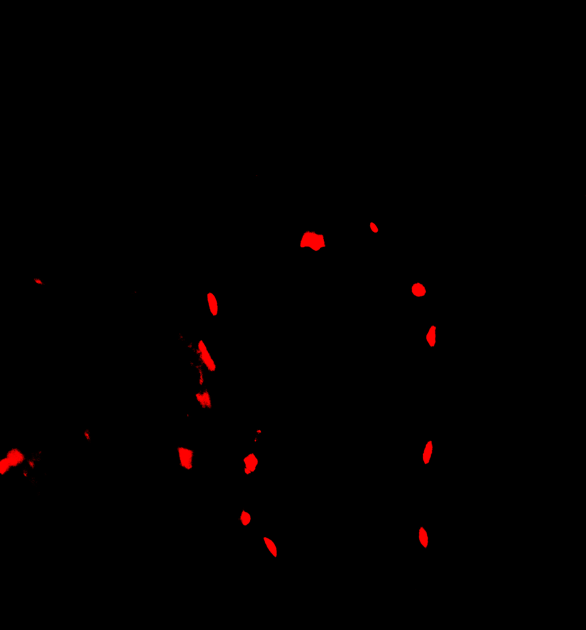

Supplement: Supplementary file 6 — Source data Fig. 4 [file 44321_2025_286_MOESM6_ESM.zip › Figure 4/4A/8-OHdG_HSFD_LE_8-OHdG.tif]

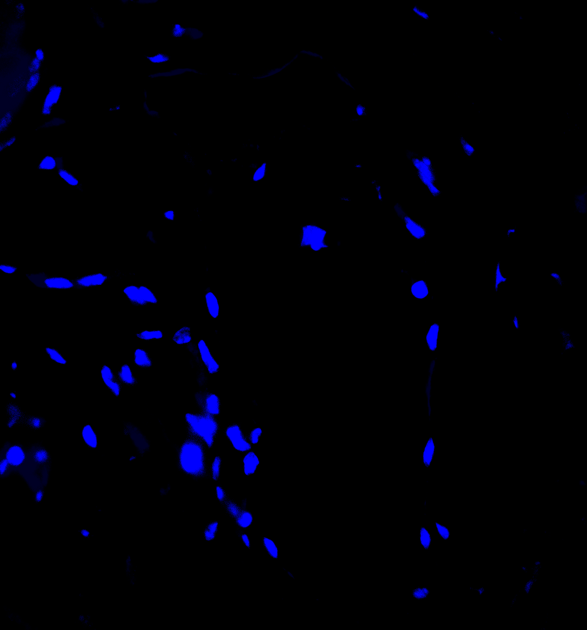

Supplement: Supplementary file 6 — Source data Fig. 4 [file 44321_2025_286_MOESM6_ESM.zip › Figure 4/4A/8-OHdG_HSFD_LE_DAPI.tif]

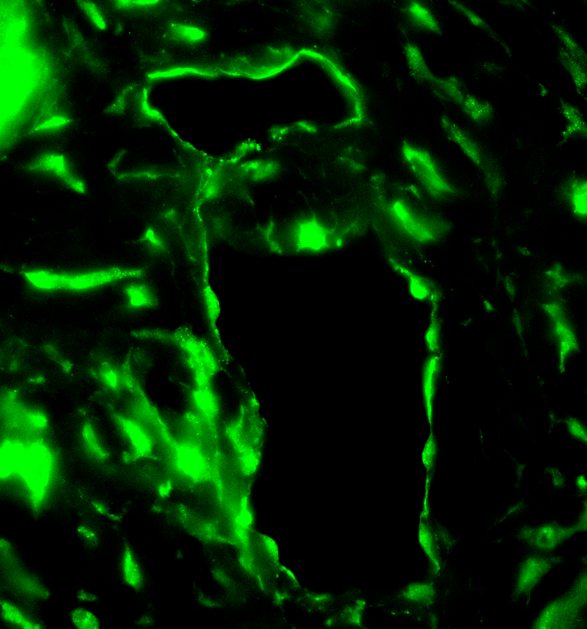

Supplement: Supplementary file 6 — Source data Fig. 4 [file 44321_2025_286_MOESM6_ESM.zip › Figure 4/4A/8-OHdG_HSFD_LE_LYVE-1.tif]

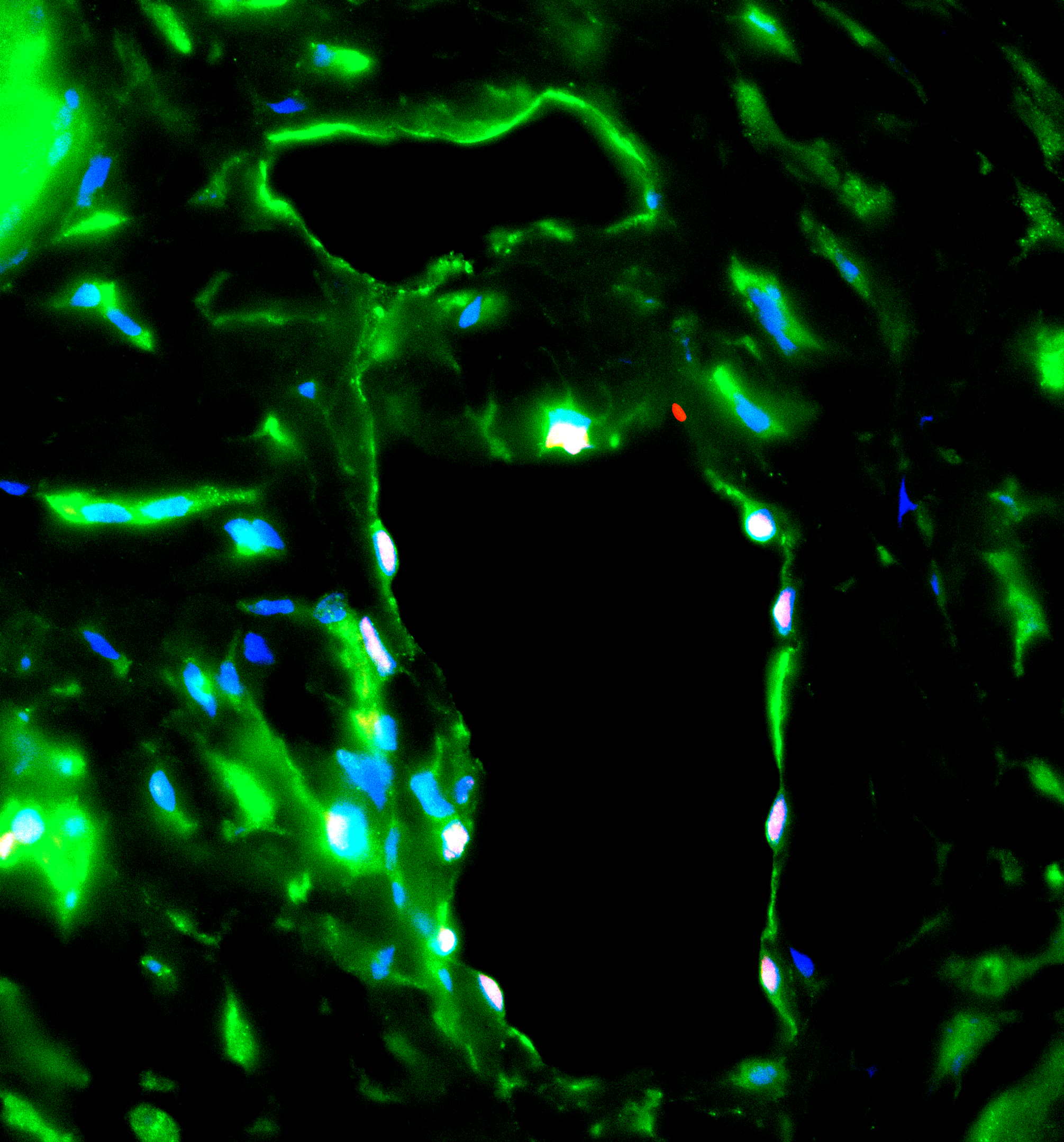

Supplement: Supplementary file 6 — Source data Fig. 4 [file 44321_2025_286_MOESM6_ESM.zip › Figure 4/4A/8-OHdG_HSFD_LE_Merged.tif]

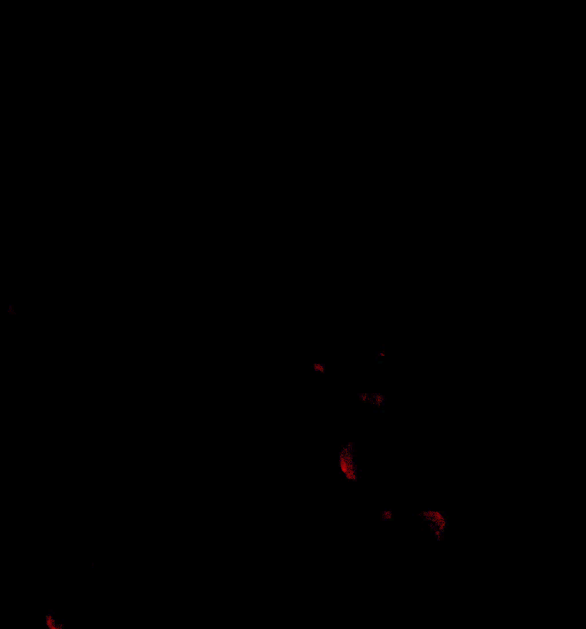

Supplement: Supplementary file 6 — Source data Fig. 4 [file 44321_2025_286_MOESM6_ESM.zip › Figure 4/4A/8-OHdG_HSFD_Sham_8-OHdG.tif]

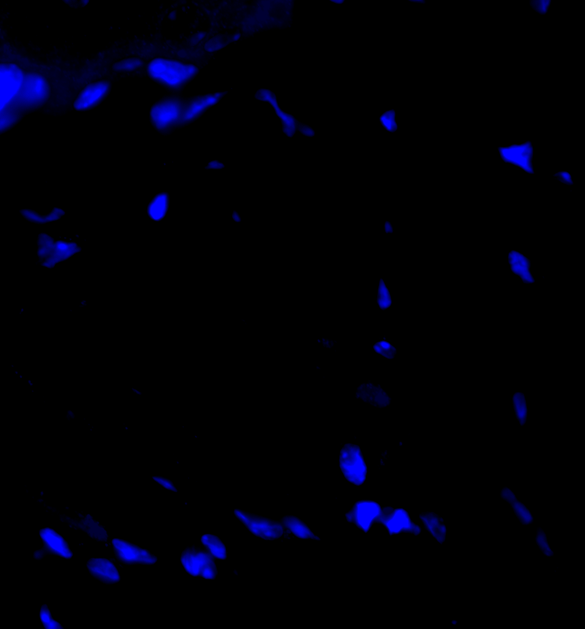

Supplement: Supplementary file 6 — Source data Fig. 4 [file 44321_2025_286_MOESM6_ESM.zip › Figure 4/4A/8-OHdG_HSFD_Sham_DAPI.tif]

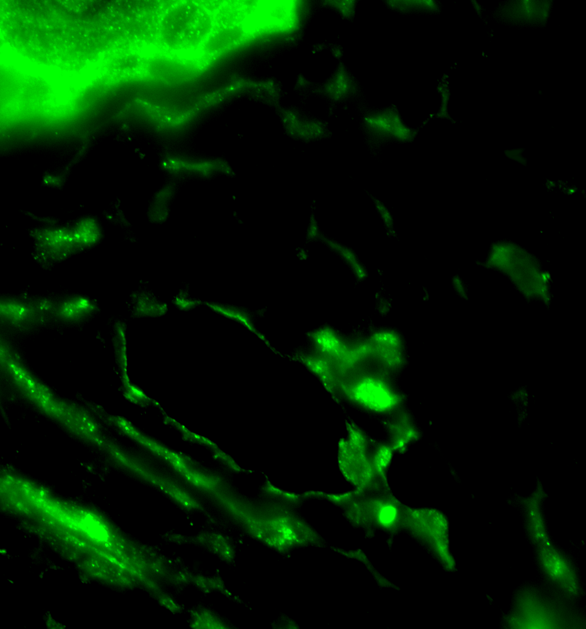

Supplement: Supplementary file 6 — Source data Fig. 4 [file 44321_2025_286_MOESM6_ESM.zip › Figure 4/4A/8-OHdG_HSFD_Sham_LYVE-1.tif]

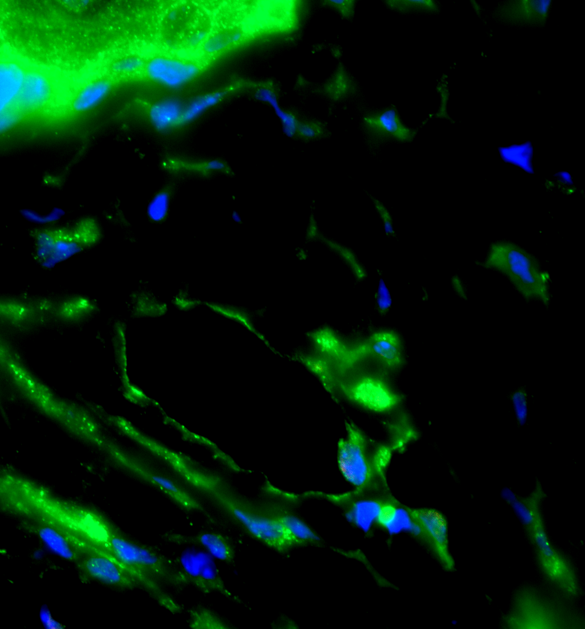

Supplement: Supplementary file 6 — Source data Fig. 4 [file 44321_2025_286_MOESM6_ESM.zip › Figure 4/4A/8-OHdG_HSFD_Sham_Merged.tif]

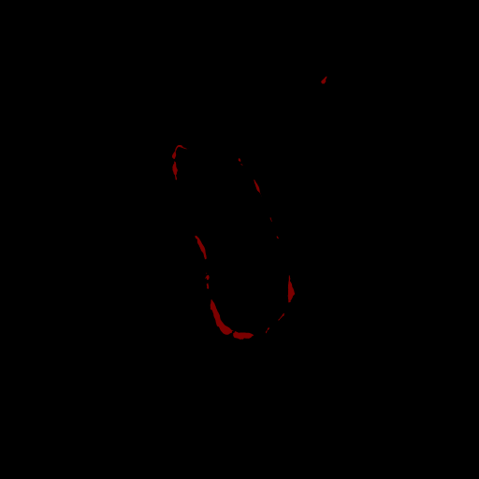

Supplement: Supplementary file 7 — Source data Fig. 5 [file 44321_2025_286_MOESM7_ESM.zip › Figure 5/5C/CHOP_HSFD-CD_LE_CHOP.tif]

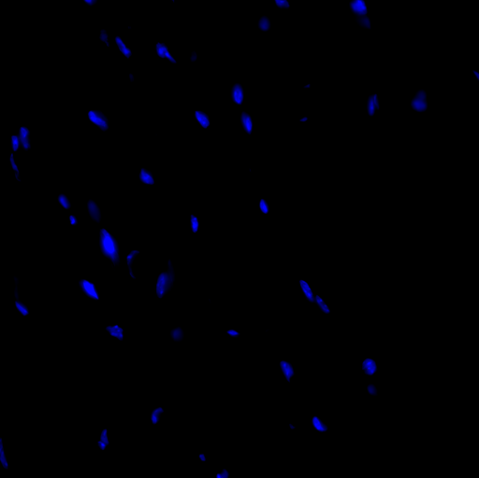

Supplement: Supplementary file 7 — Source data Fig. 5 [file 44321_2025_286_MOESM7_ESM.zip › Figure 5/5C/CHOP_HSFD-CD_LE_DAPI.tif]

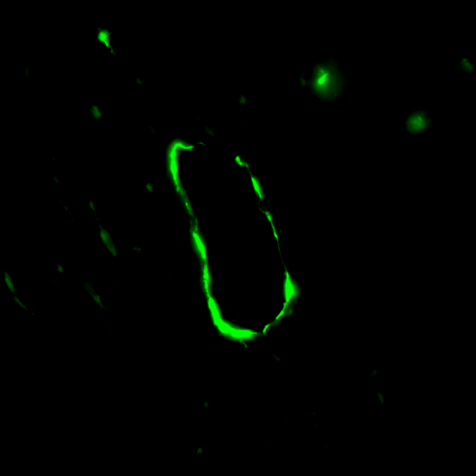

Supplement: Supplementary file 7 — Source data Fig. 5 [file 44321_2025_286_MOESM7_ESM.zip › Figure 5/5C/CHOP_HSFD-CD_LE_LYVE-1.tif]

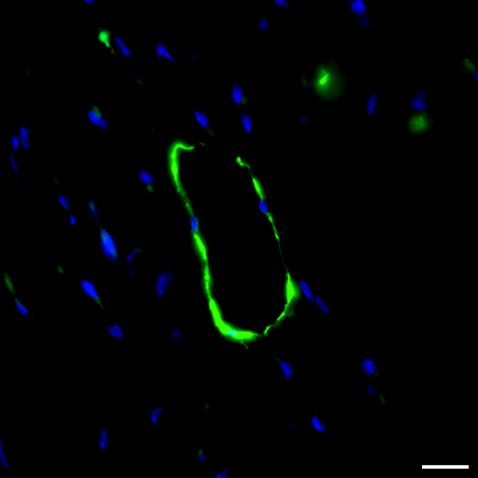

Supplement: Supplementary file 7 — Source data Fig. 5 [file 44321_2025_286_MOESM7_ESM.zip › Figure 5/5C/CHOP_HSFD-CD_LE_Merged.tif]

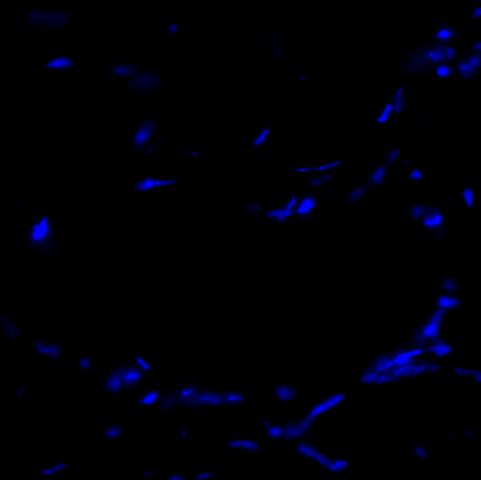

Supplement: Supplementary file 7 — Source data Fig. 5 [file 44321_2025_286_MOESM7_ESM.zip › Figure 5/5C/CHOP_HSFD_LE_DAPI.tif]

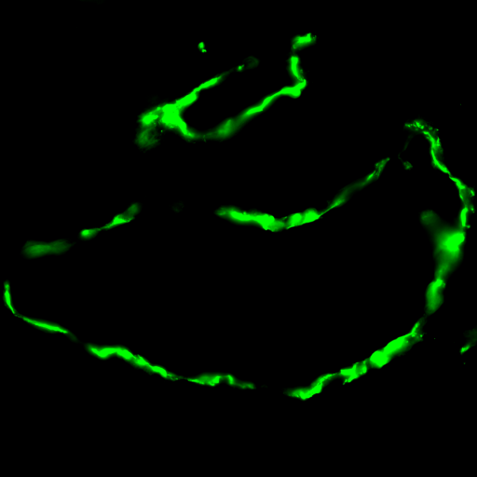

Supplement: Supplementary file 7 — Source data Fig. 5 [file 44321_2025_286_MOESM7_ESM.zip › Figure 5/5C/CHOP_HSFD_LE_LYVE-1.tif]

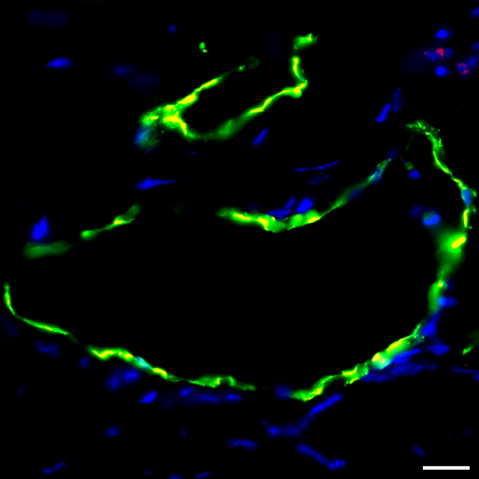

Supplement: Supplementary file 7 — Source data Fig. 5 [file 44321_2025_286_MOESM7_ESM.zip › Figure 5/5C/CHOP_HSFD_LE_Merged.tif]

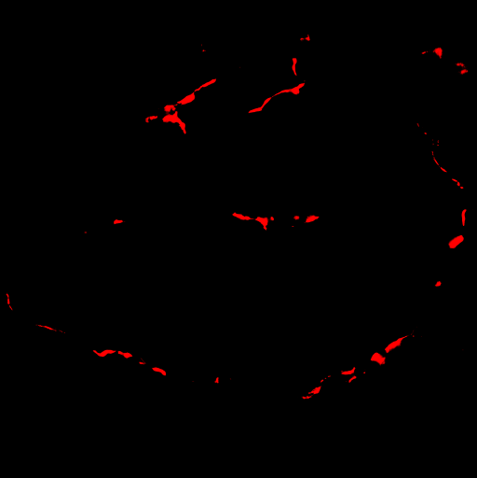

Supplement: Supplementary file 7 — Source data Fig. 5 [file 44321_2025_286_MOESM7_ESM.zip › Figure 5/5C/CHOP_HSFD_LE_sXBP-1.tif]

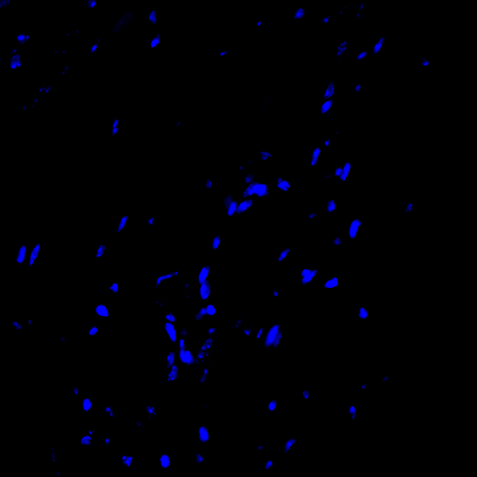

Supplement: Supplementary file 7 — Source data Fig. 5 [file 44321_2025_286_MOESM7_ESM.zip › Figure 5/5C/sXBP-1_HSFD-CD_LE_DAPI.tif]

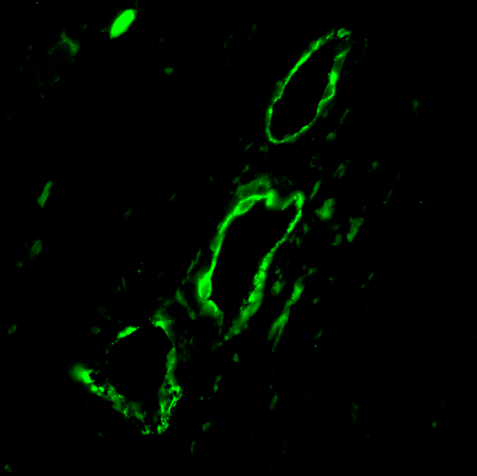

Supplement: Supplementary file 7 — Source data Fig. 5 [file 44321_2025_286_MOESM7_ESM.zip › Figure 5/5C/sXBP-1_HSFD-CD_LE_LYVE-1.tif]

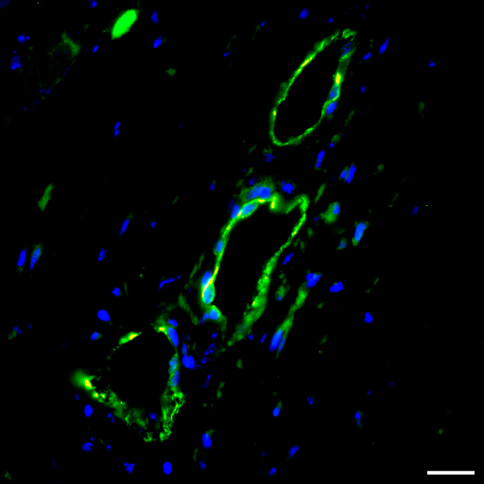

Supplement: Supplementary file 7 — Source data Fig. 5 [file 44321_2025_286_MOESM7_ESM.zip › Figure 5/5C/sXBP-1_HSFD-CD_LE_Merged.tif]

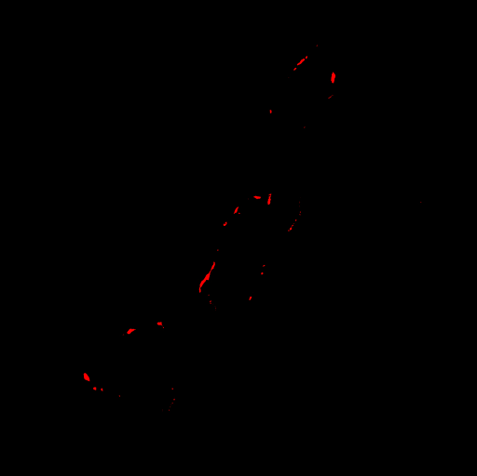

Supplement: Supplementary file 7 — Source data Fig. 5 [file 44321_2025_286_MOESM7_ESM.zip › Figure 5/5C/sXBP-1_HSFD-CD_LE_sXBP-1.tif]

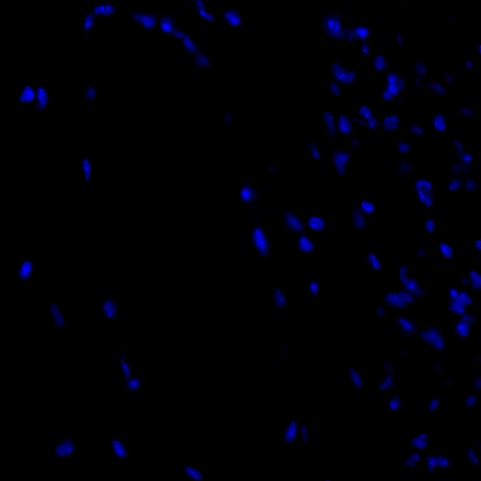

Supplement: Supplementary file 7 — Source data Fig. 5 [file 44321_2025_286_MOESM7_ESM.zip › Figure 5/5C/sXBP-1_HSFD_LE_DAPI.tif]

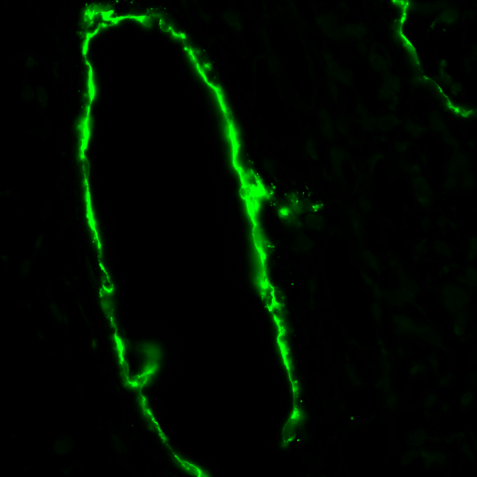

Supplement: Supplementary file 7 — Source data Fig. 5 [file 44321_2025_286_MOESM7_ESM.zip › Figure 5/5C/sXBP-1_HSFD_LE_LYVE-1.tif]

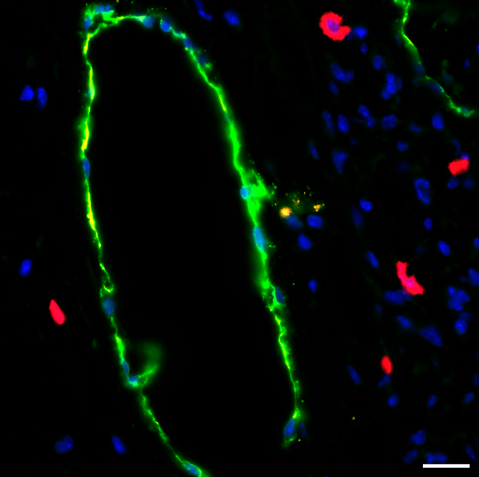

Supplement: Supplementary file 7 — Source data Fig. 5 [file 44321_2025_286_MOESM7_ESM.zip › Figure 5/5C/sXBP-1_HSFD_LE_Merged.tif]

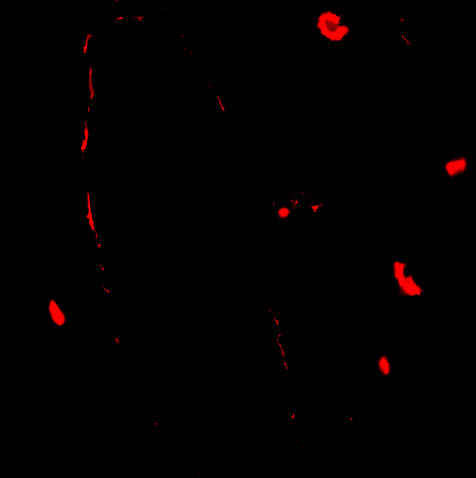

Supplement: Supplementary file 7 — Source data Fig. 5 [file 44321_2025_286_MOESM7_ESM.zip › Figure 5/5C/sXBP-1_HSFD_LE_sXBP-1.tif]

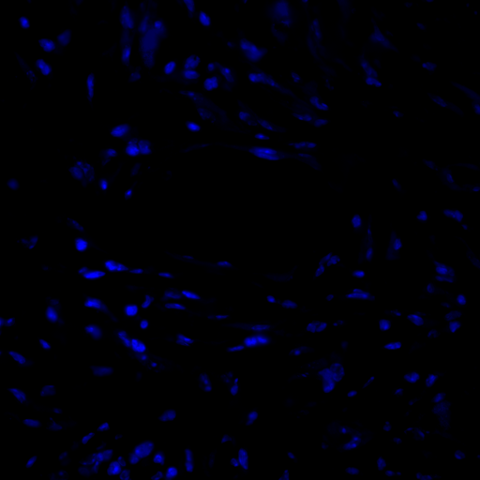

Supplement: Supplementary file 7 — Source data Fig. 5 [file 44321_2025_286_MOESM7_ESM.zip › Figure 5/5C/TUNEL_HSFD-CD_LE_DAPI.tif]

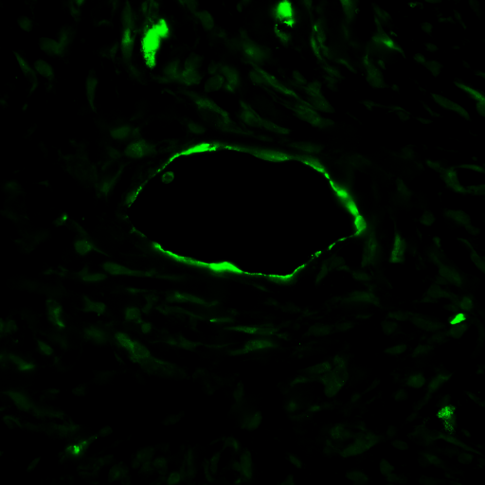

Supplement: Supplementary file 7 — Source data Fig. 5 [file 44321_2025_286_MOESM7_ESM.zip › Figure 5/5C/TUNEL_HSFD-CD_LE_LYVE-1.tif]

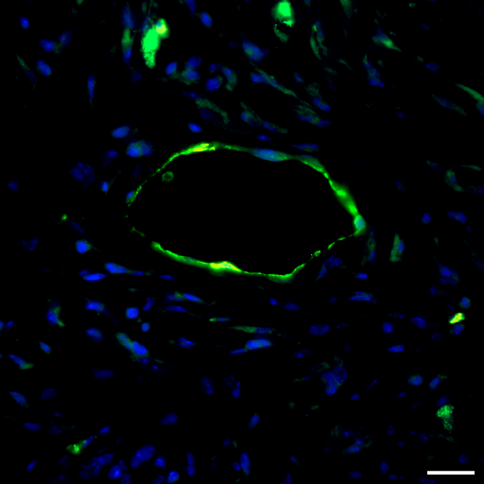

Supplement: Supplementary file 7 — Source data Fig. 5 [file 44321_2025_286_MOESM7_ESM.zip › Figure 5/5C/TUNEL_HSFD-CD_LE_Merged.tif]

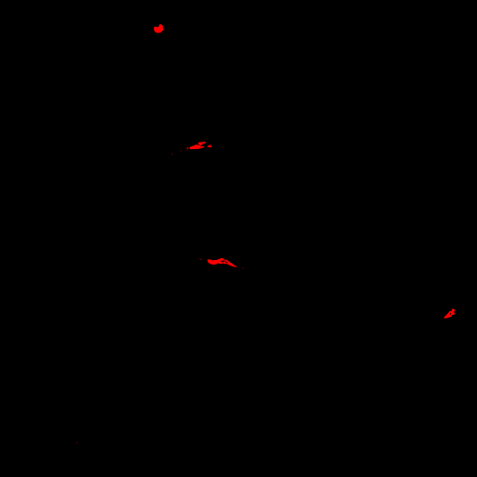

Supplement: Supplementary file 7 — Source data Fig. 5 [file 44321_2025_286_MOESM7_ESM.zip › Figure 5/5C/TUNEL_HSFD-CD_LE_TUNEL.tif]

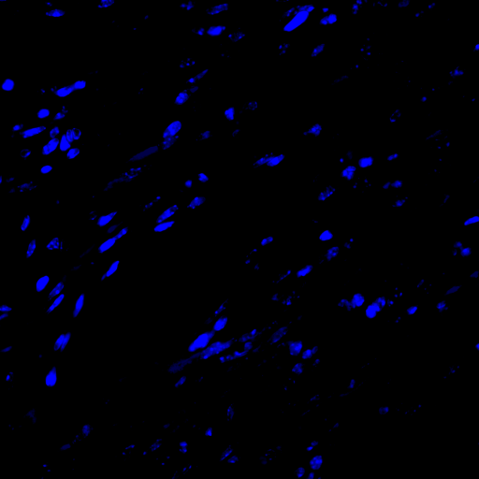

Supplement: Supplementary file 7 — Source data Fig. 5 [file 44321_2025_286_MOESM7_ESM.zip › Figure 5/5C/TUNEL_HSFD_LE_DAPI.tif]

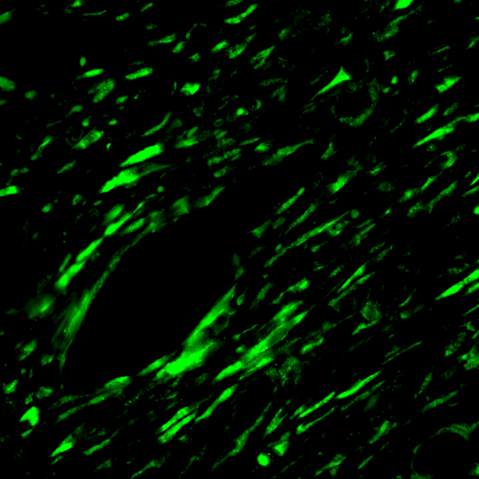

Supplement: Supplementary file 7 — Source data Fig. 5 [file 44321_2025_286_MOESM7_ESM.zip › Figure 5/5C/TUNEL_HSFD_LE_LYVE-1.tif]

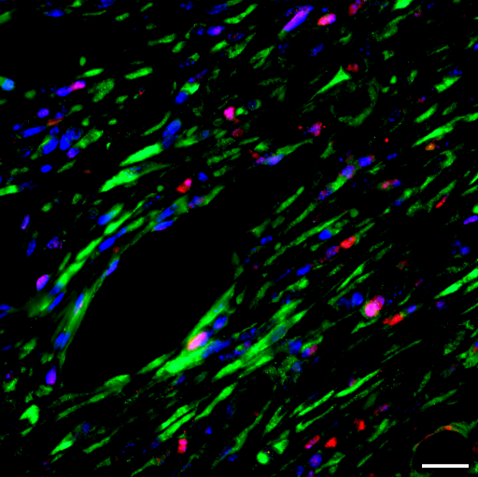

Supplement: Supplementary file 7 — Source data Fig. 5 [file 44321_2025_286_MOESM7_ESM.zip › Figure 5/5C/TUNEL_HSFD_LE_Merged.tif]

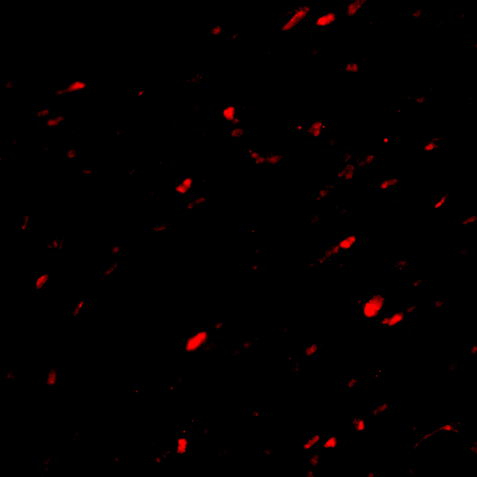

Supplement: Supplementary file 7 — Source data Fig. 5 [file 44321_2025_286_MOESM7_ESM.zip › Figure 5/5C/TUNEL_HSFD_LE_sXBP-1.tif]
