# Supplementary figures and images for: Saturated fatty acids induce lipotoxicity in lymphatic endothelial cells contributing to secondary lymphedema development (part 2 of 3)
Source: EMBO Mol Med. 2025 Aug 4;17(9):2384–408. doi: 10.1038/s44321-025-00286-4 (PMC12423331; doi:10.1038/s44321-025-00286-4)

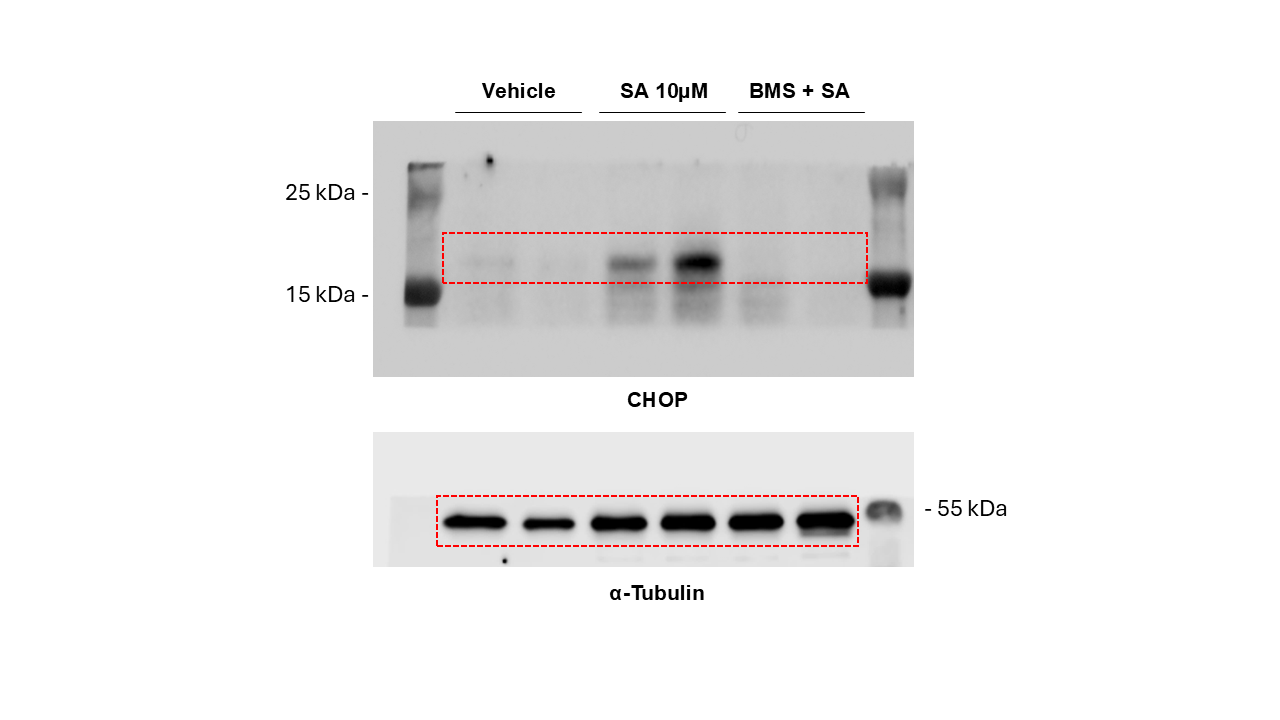

Supplement: Supplementary file 8 — Source data Fig. 6 [file 44321_2025_286_MOESM8_ESM.zip › Figure 6/6C/Chop_WesternBlot.tif]

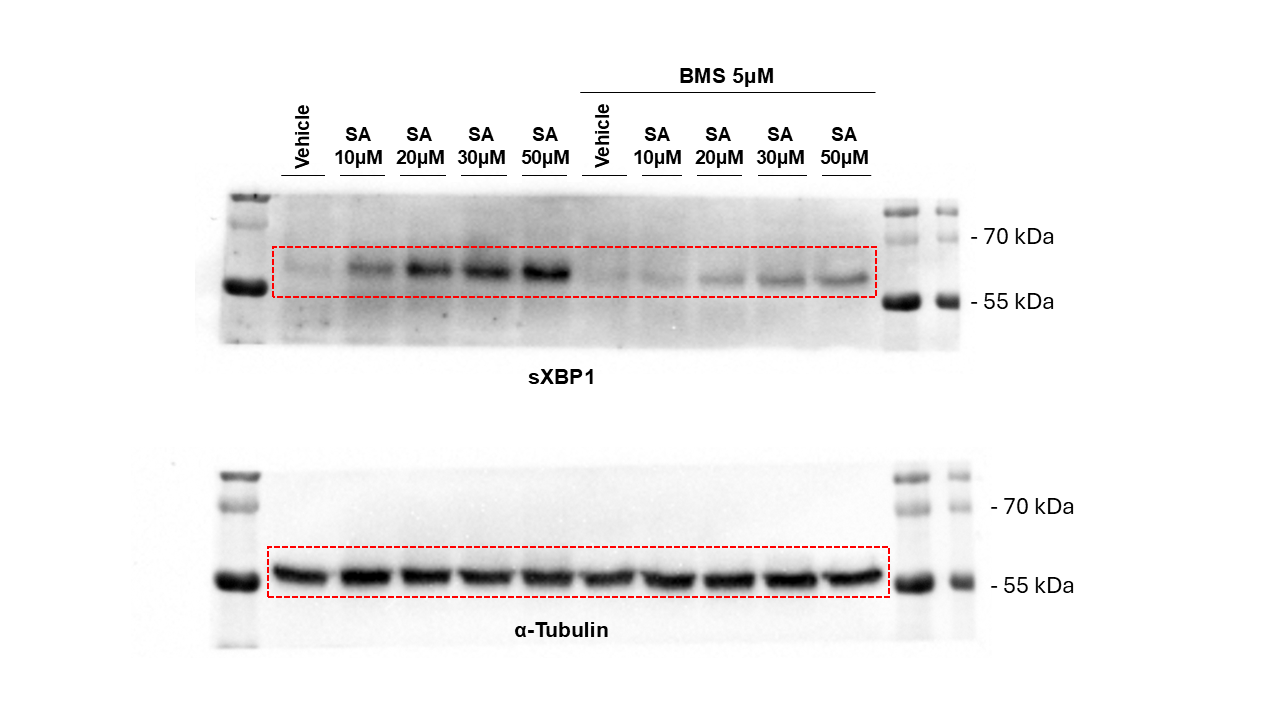

Supplement: Supplementary file 8 — Source data Fig. 6 [file 44321_2025_286_MOESM8_ESM.zip › Figure 6/6C/sXBP-1_WesternBlot.tif]

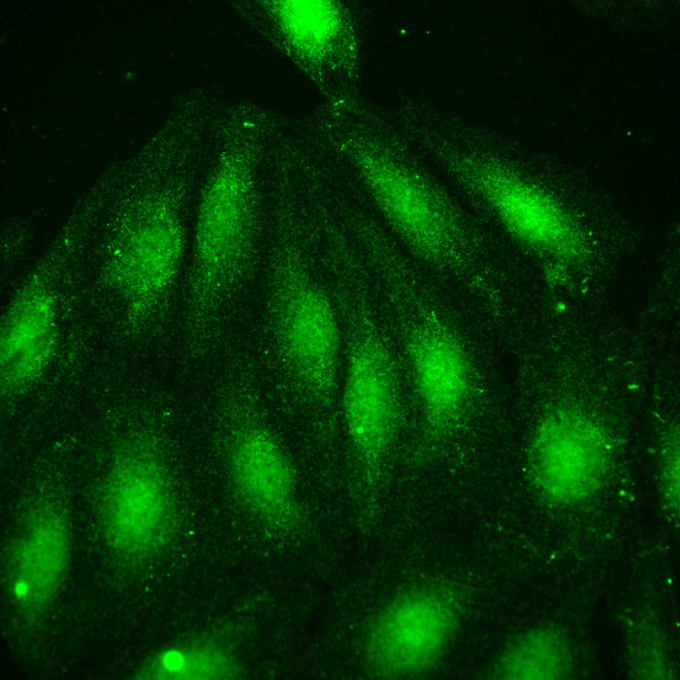

Supplement: Supplementary file 8 — Source data Fig. 6 [file 44321_2025_286_MOESM8_ESM.zip › Figure 6/6D/BMS+SA-calreticulin.tif]

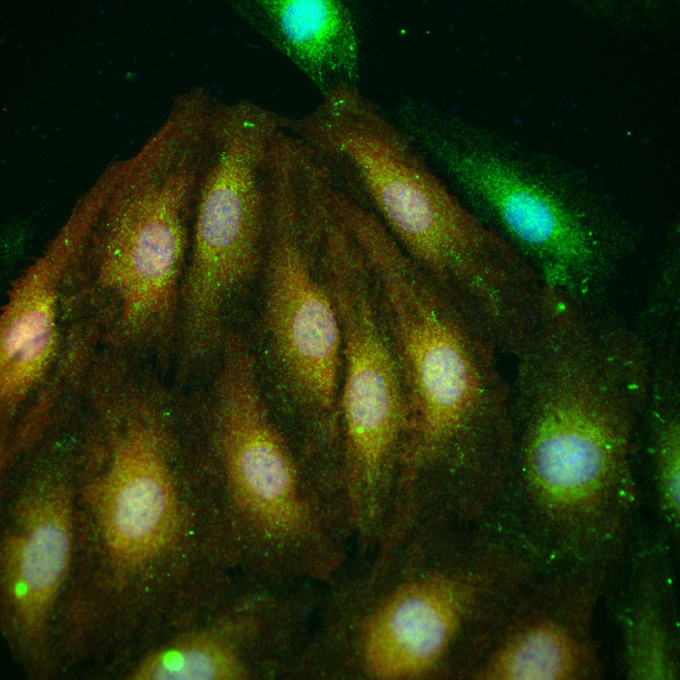

Supplement: Supplementary file 8 — Source data Fig. 6 [file 44321_2025_286_MOESM8_ESM.zip › Figure 6/6D/BMS+SA-composite.tif]

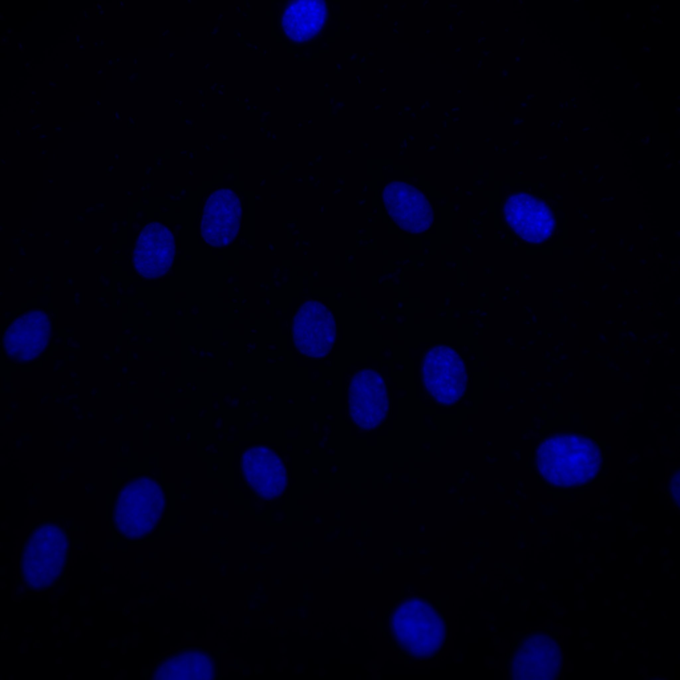

Supplement: Supplementary file 8 — Source data Fig. 6 [file 44321_2025_286_MOESM8_ESM.zip › Figure 6/6D/BMS+SA-DAPI.tif]

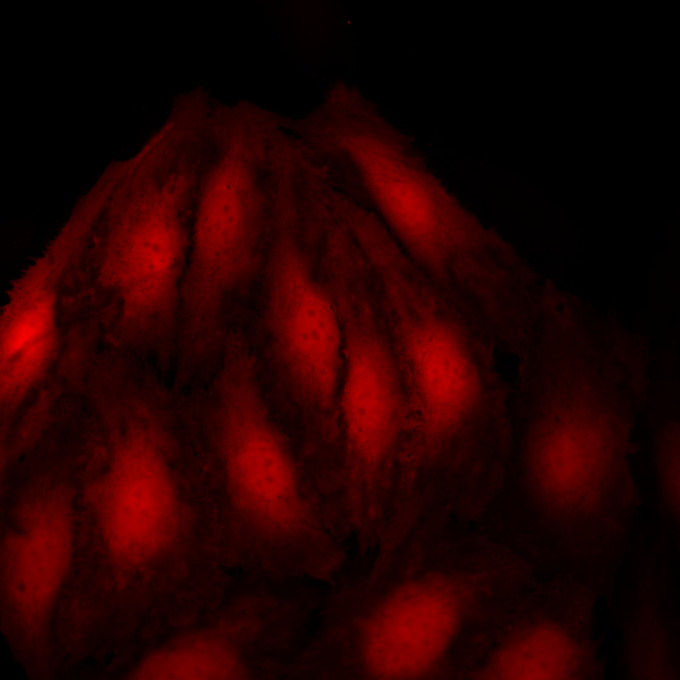

Supplement: Supplementary file 8 — Source data Fig. 6 [file 44321_2025_286_MOESM8_ESM.zip › Figure 6/6D/BMS+SA-FABP4.tif]

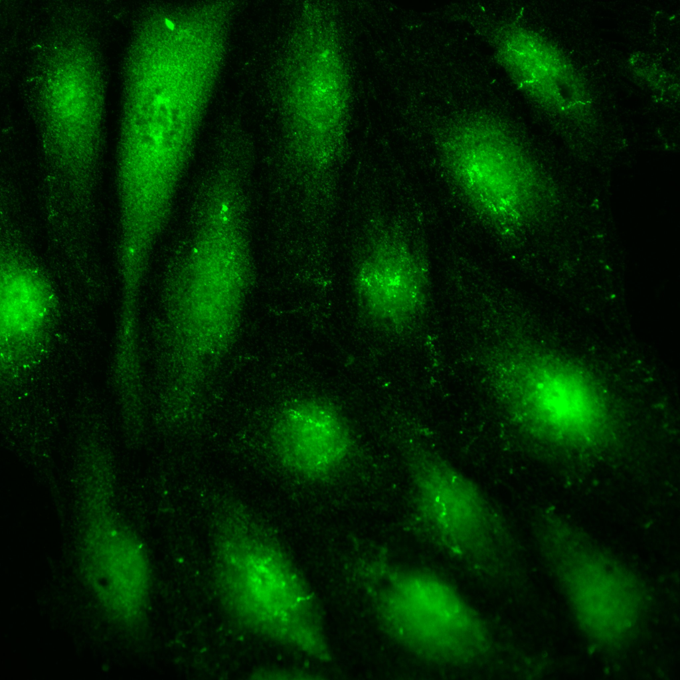

Supplement: Supplementary file 8 — Source data Fig. 6 [file 44321_2025_286_MOESM8_ESM.zip › Figure 6/6D/BMS-calreticulin.tif]

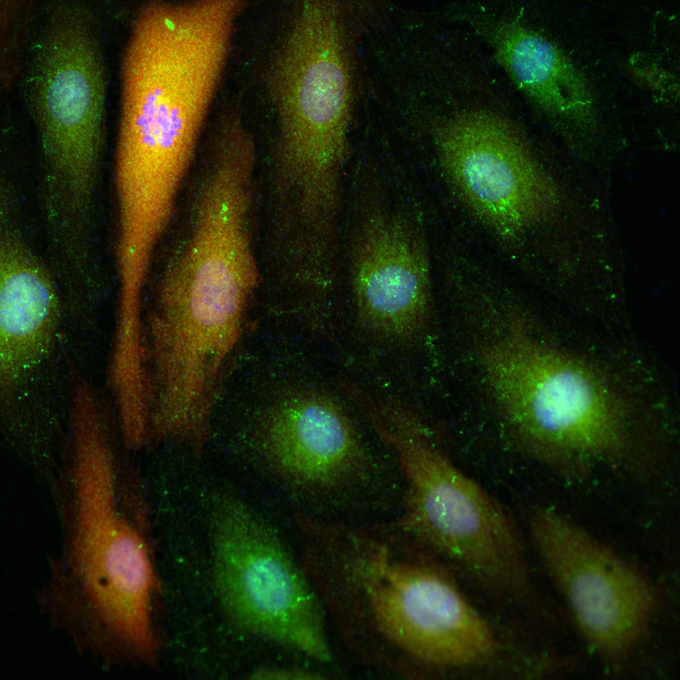

Supplement: Supplementary file 8 — Source data Fig. 6 [file 44321_2025_286_MOESM8_ESM.zip › Figure 6/6D/BMS-composite.tif]

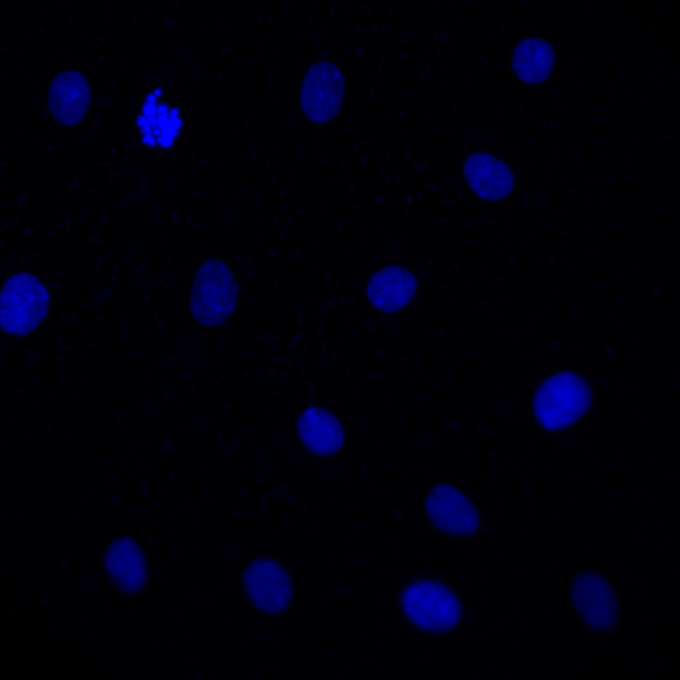

Supplement: Supplementary file 8 — Source data Fig. 6 [file 44321_2025_286_MOESM8_ESM.zip › Figure 6/6D/BMS-DAPI.tif]

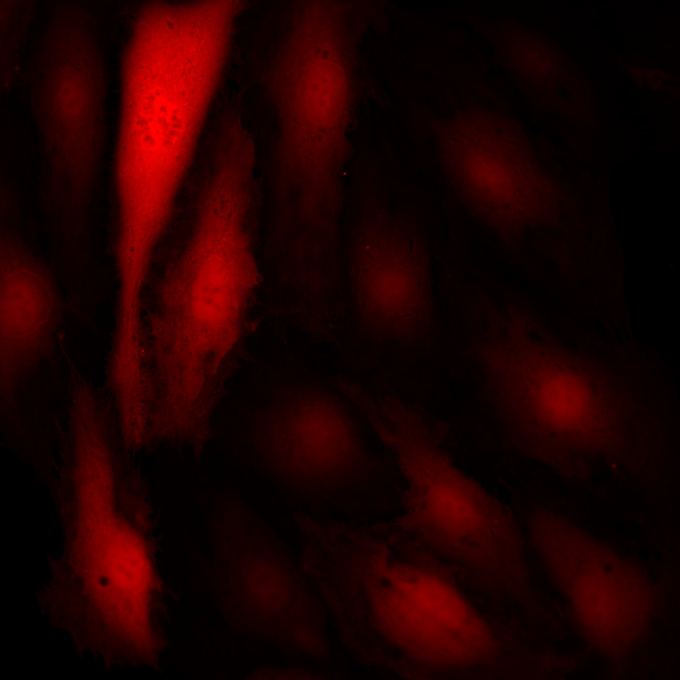

Supplement: Supplementary file 8 — Source data Fig. 6 [file 44321_2025_286_MOESM8_ESM.zip › Figure 6/6D/BMS-FABP4.tif]

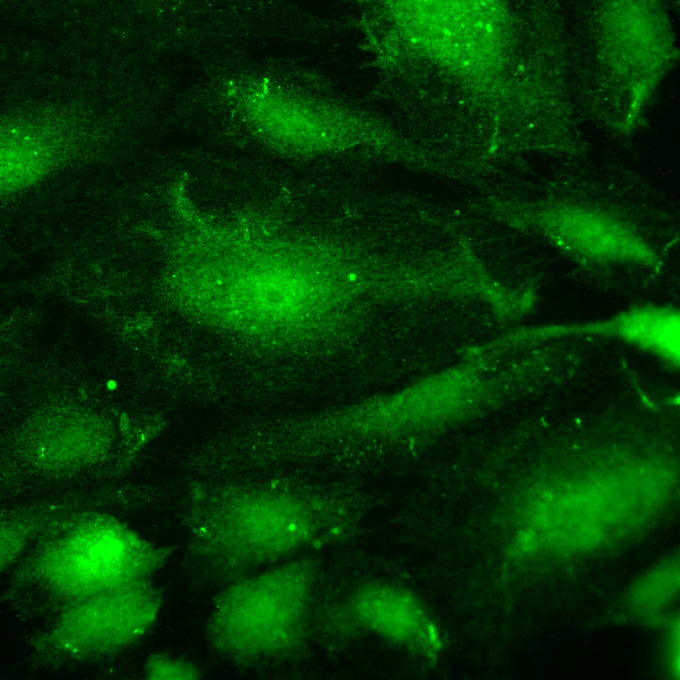

Supplement: Supplementary file 8 — Source data Fig. 6 [file 44321_2025_286_MOESM8_ESM.zip › Figure 6/6D/SA-calreticulin.tif]

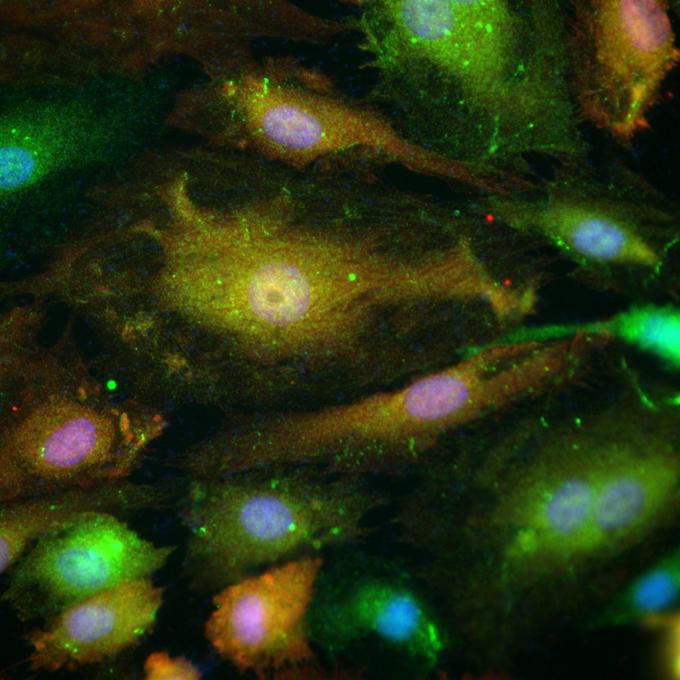

Supplement: Supplementary file 8 — Source data Fig. 6 [file 44321_2025_286_MOESM8_ESM.zip › Figure 6/6D/SA-composite.tif]

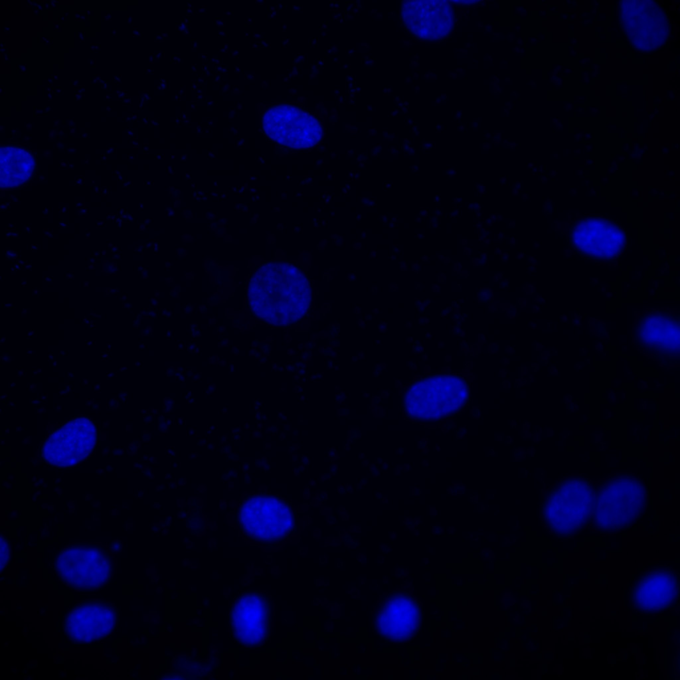

Supplement: Supplementary file 8 — Source data Fig. 6 [file 44321_2025_286_MOESM8_ESM.zip › Figure 6/6D/SA-DAPI.tif]

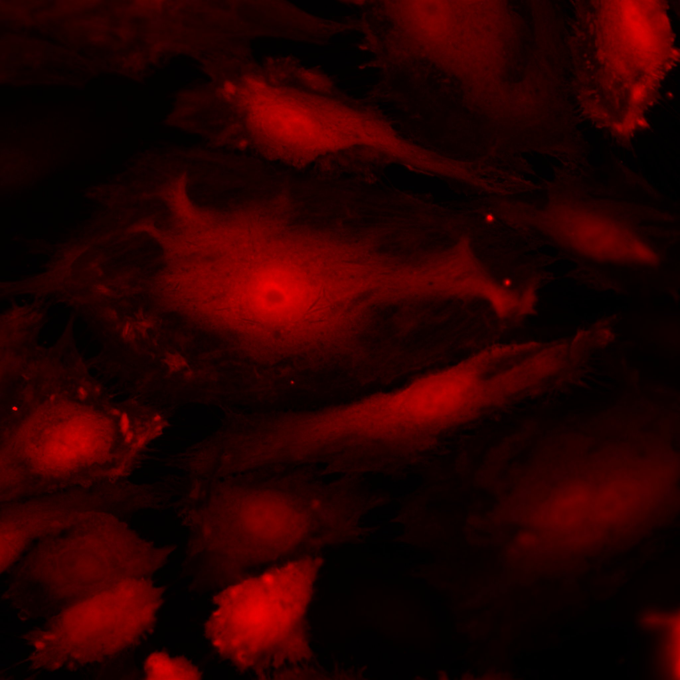

Supplement: Supplementary file 8 — Source data Fig. 6 [file 44321_2025_286_MOESM8_ESM.zip › Figure 6/6D/SA-FABP4.tif]

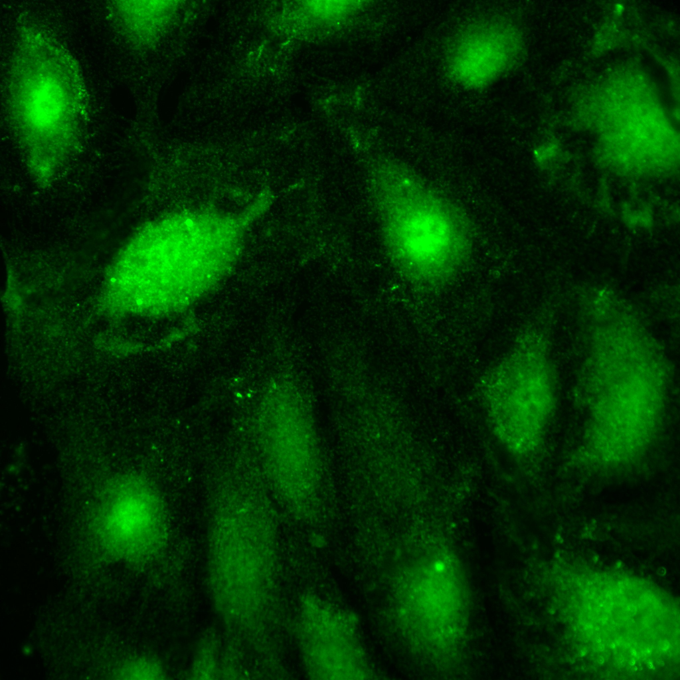

Supplement: Supplementary file 8 — Source data Fig. 6 [file 44321_2025_286_MOESM8_ESM.zip › Figure 6/6D/Vehicle-calreticulin.tif]

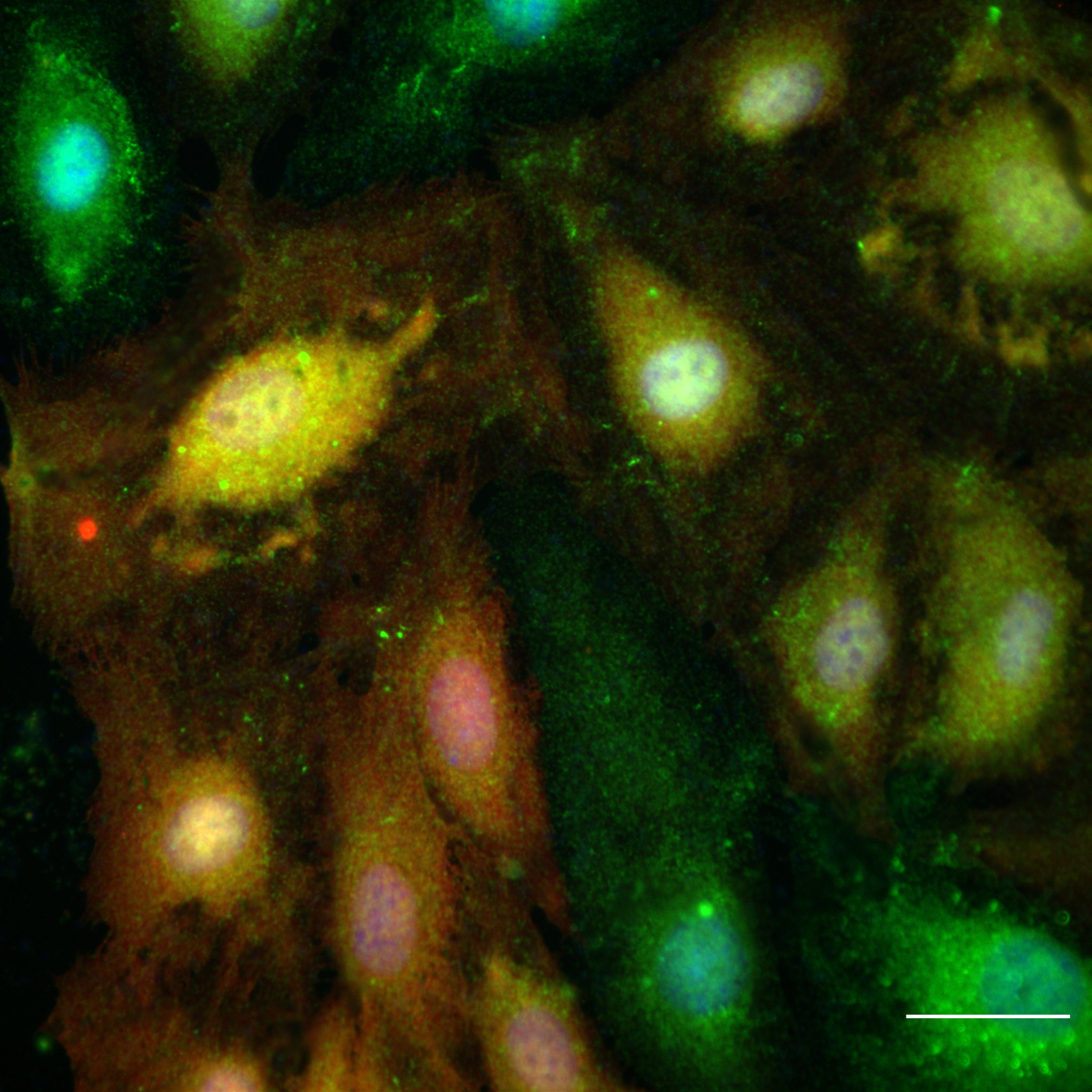

Supplement: Supplementary file 8 — Source data Fig. 6 [file 44321_2025_286_MOESM8_ESM.zip › Figure 6/6D/Vehicle-composite.tif]

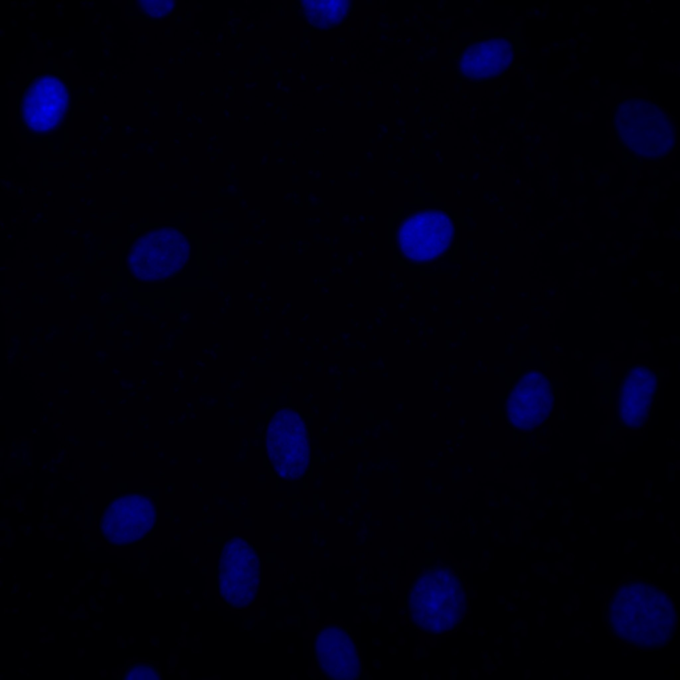

Supplement: Supplementary file 8 — Source data Fig. 6 [file 44321_2025_286_MOESM8_ESM.zip › Figure 6/6D/Vehicle-DAPI.tif]

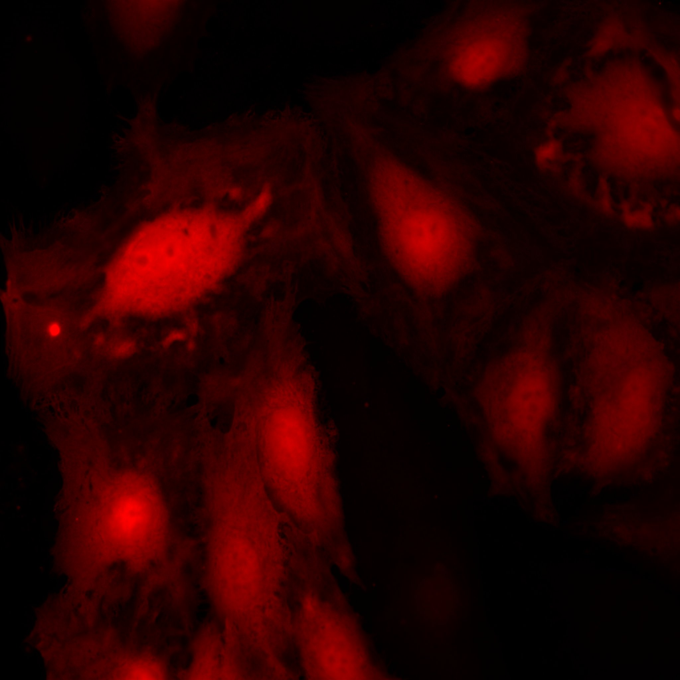

Supplement: Supplementary file 8 — Source data Fig. 6 [file 44321_2025_286_MOESM8_ESM.zip › Figure 6/6D/Vehicle-FABP4.tif]

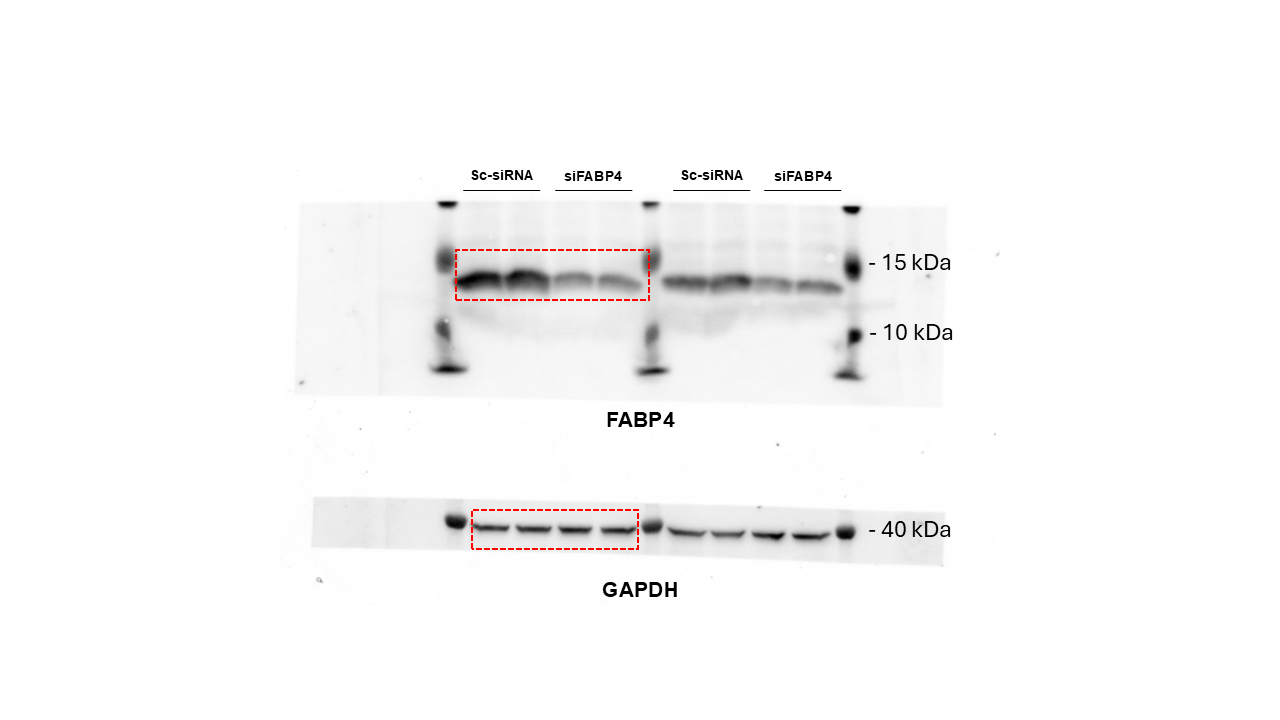

Supplement: Supplementary file 8 — Source data Fig. 6 [file 44321_2025_286_MOESM8_ESM.zip › Figure 6/6G/FABP4_WesternBlot.tif]

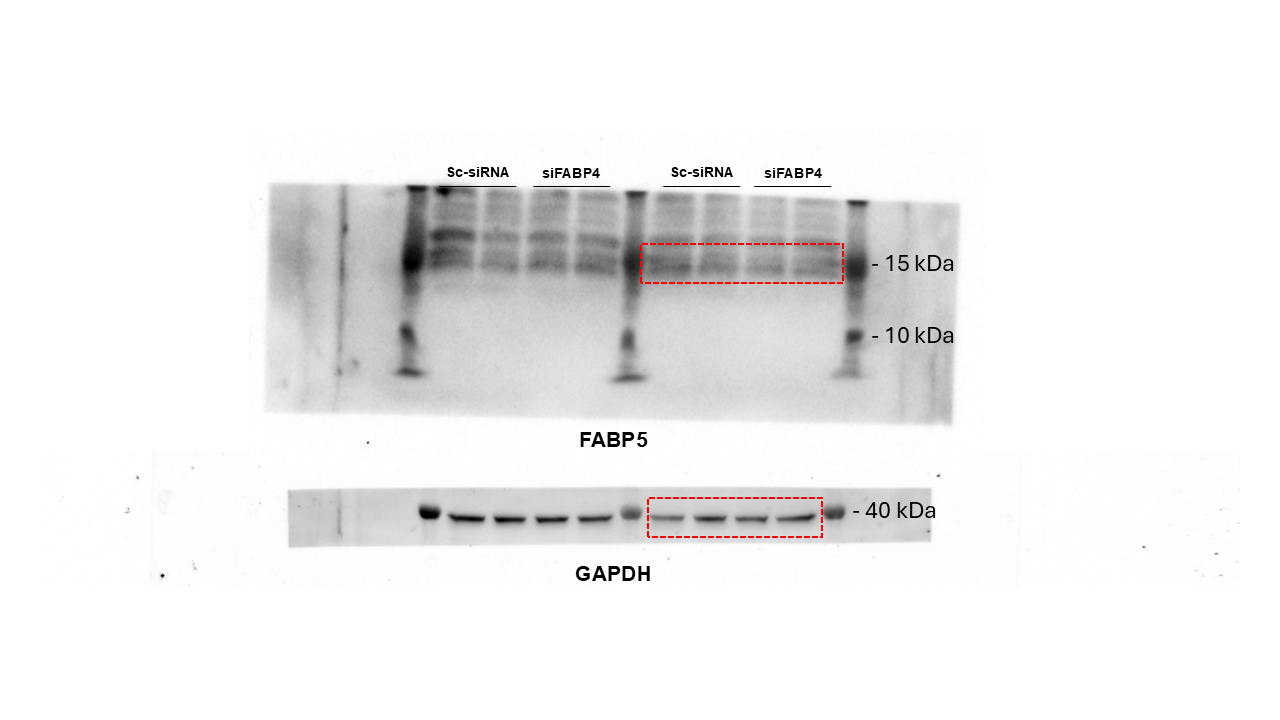

Supplement: Supplementary file 8 — Source data Fig. 6 [file 44321_2025_286_MOESM8_ESM.zip › Figure 6/6G/FABP5_WesternBlot.tif]

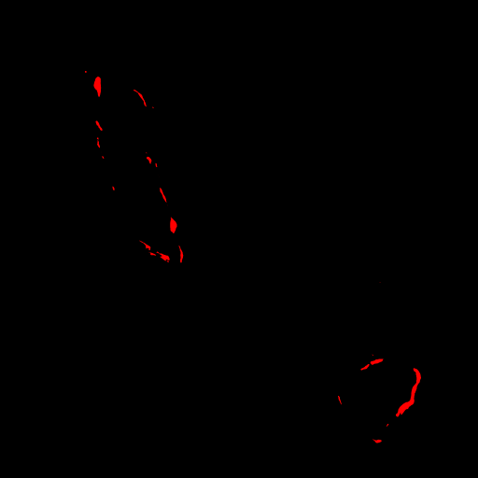

Supplement: Supplementary file 9 — Source data Fig. 7 [file 44321_2025_286_MOESM9_ESM.zip › Figure 7/7C/CHOP_HSFD+BMS_LE_CHOP.tif]

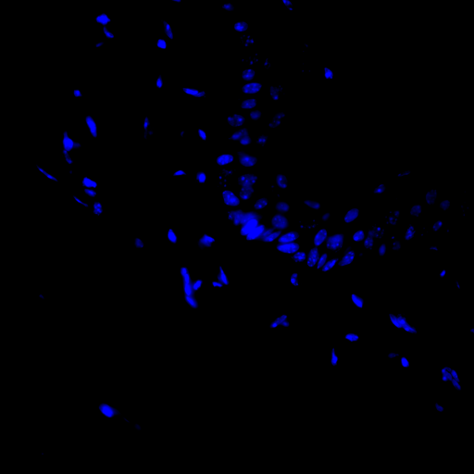

Supplement: Supplementary file 9 — Source data Fig. 7 [file 44321_2025_286_MOESM9_ESM.zip › Figure 7/7C/CHOP_HSFD+BMS_LE_DAPI.tif]

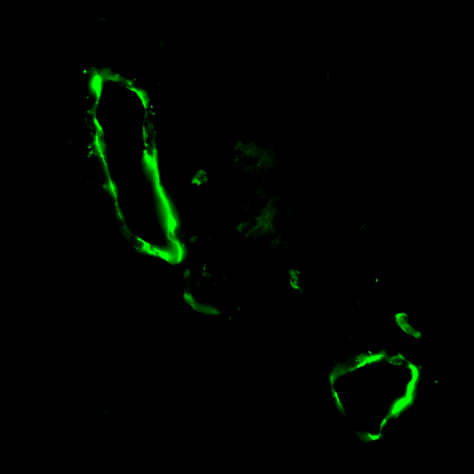

Supplement: Supplementary file 9 — Source data Fig. 7 [file 44321_2025_286_MOESM9_ESM.zip › Figure 7/7C/CHOP_HSFD+BMS_LE_LYVE-1.tif]

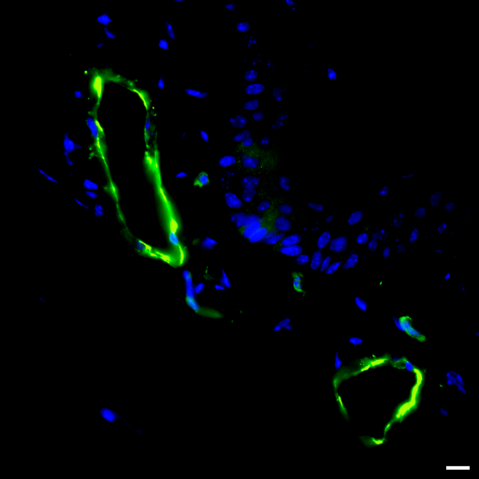

Supplement: Supplementary file 9 — Source data Fig. 7 [file 44321_2025_286_MOESM9_ESM.zip › Figure 7/7C/CHOP_HSFD+BMS_LE_Merged.tif]

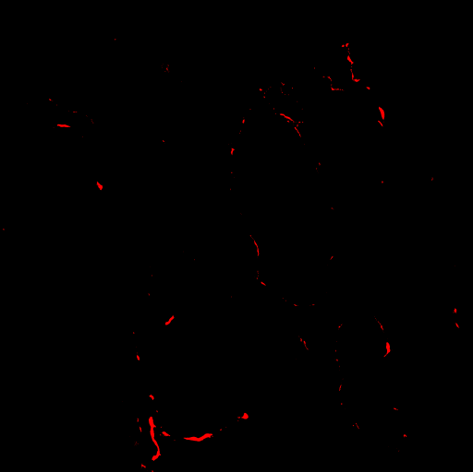

Supplement: Supplementary file 9 — Source data Fig. 7 [file 44321_2025_286_MOESM9_ESM.zip › Figure 7/7C/CHOP_HSFD+PBS_LE_CHOP.tif]

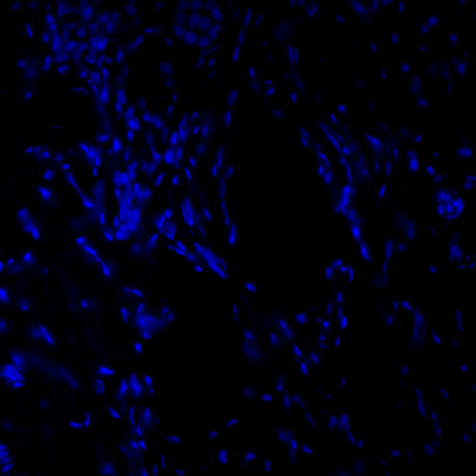

Supplement: Supplementary file 9 — Source data Fig. 7 [file 44321_2025_286_MOESM9_ESM.zip › Figure 7/7C/CHOP_HSFD+PBS_LE_DAPI.tif]

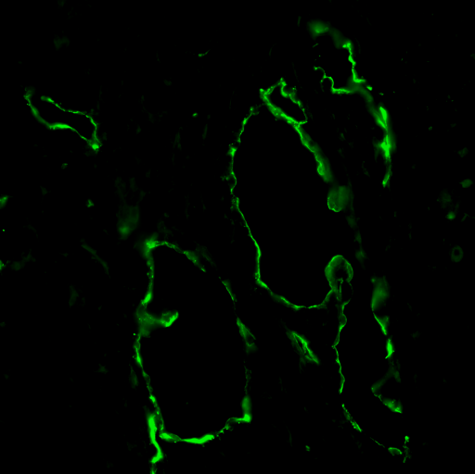

Supplement: Supplementary file 9 — Source data Fig. 7 [file 44321_2025_286_MOESM9_ESM.zip › Figure 7/7C/CHOP_HSFD+PBS_LE_LYVE-1.tif]

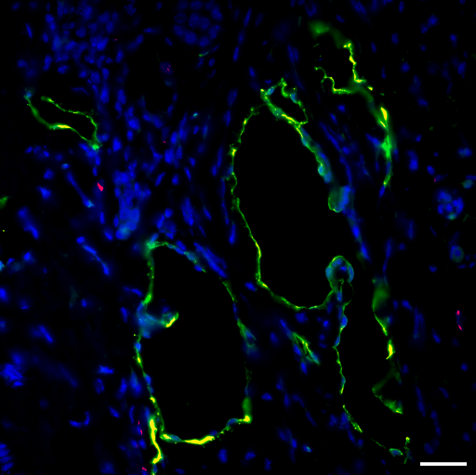

Supplement: Supplementary file 9 — Source data Fig. 7 [file 44321_2025_286_MOESM9_ESM.zip › Figure 7/7C/CHOP_HSFD+PBS_LE_Merged.tif]

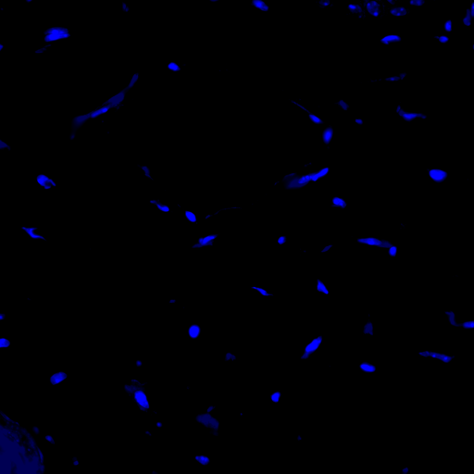

Supplement: Supplementary file 9 — Source data Fig. 7 [file 44321_2025_286_MOESM9_ESM.zip › Figure 7/7C/sXBP-1_HSFD+BMS_LE_DAPI.tif]

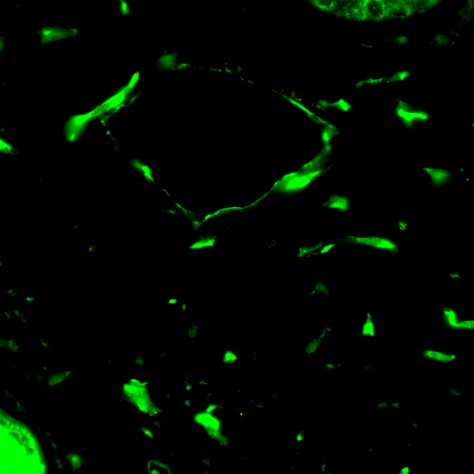

Supplement: Supplementary file 9 — Source data Fig. 7 [file 44321_2025_286_MOESM9_ESM.zip › Figure 7/7C/sXBP-1_HSFD+BMS_LE_LYVE-1.tif]

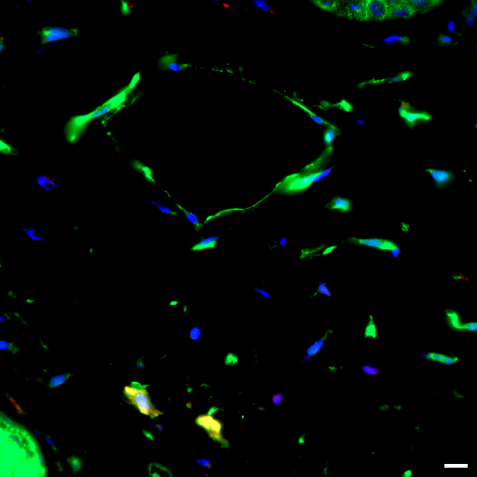

Supplement: Supplementary file 9 — Source data Fig. 7 [file 44321_2025_286_MOESM9_ESM.zip › Figure 7/7C/sXBP-1_HSFD+BMS_LE_Merged.tif]

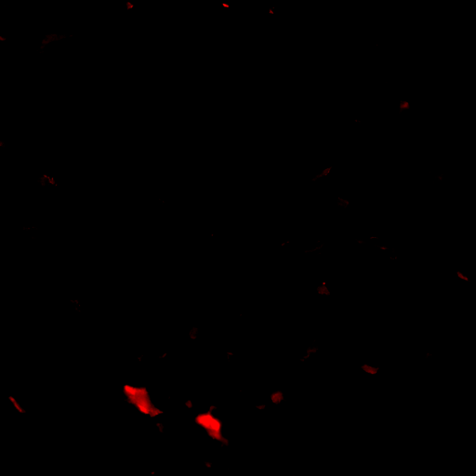

Supplement: Supplementary file 9 — Source data Fig. 7 [file 44321_2025_286_MOESM9_ESM.zip › Figure 7/7C/sXBP-1_HSFD+BMS_LE_sXBP-1.tif]

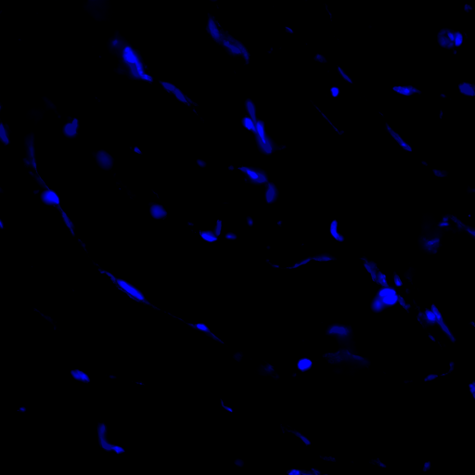

Supplement: Supplementary file 9 — Source data Fig. 7 [file 44321_2025_286_MOESM9_ESM.zip › Figure 7/7C/sXBP-1_HSFD+PBS_LE_DAPI.tif]

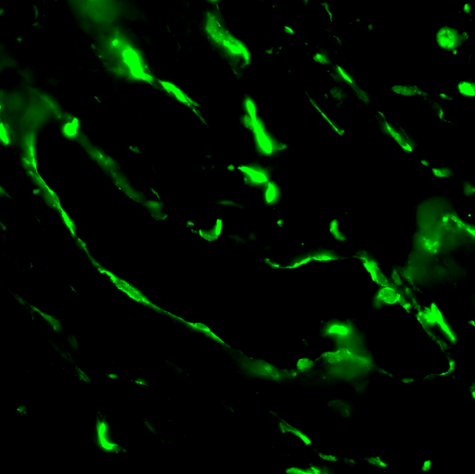

Supplement: Supplementary file 9 — Source data Fig. 7 [file 44321_2025_286_MOESM9_ESM.zip › Figure 7/7C/sXBP-1_HSFD+PBS_LE_LYVE-1.tif]

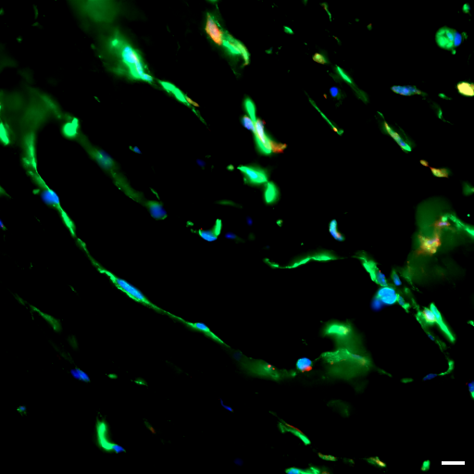

Supplement: Supplementary file 9 — Source data Fig. 7 [file 44321_2025_286_MOESM9_ESM.zip › Figure 7/7C/sXBP-1_HSFD+PBS_LE_Merged.tif]

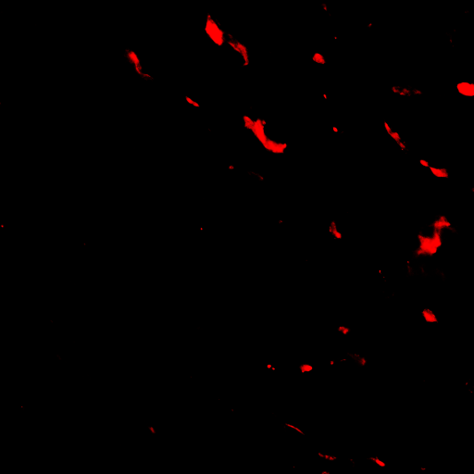

Supplement: Supplementary file 9 — Source data Fig. 7 [file 44321_2025_286_MOESM9_ESM.zip › Figure 7/7C/sXBP-1_HSFD+PBS_LE_sXBP-1.tif]

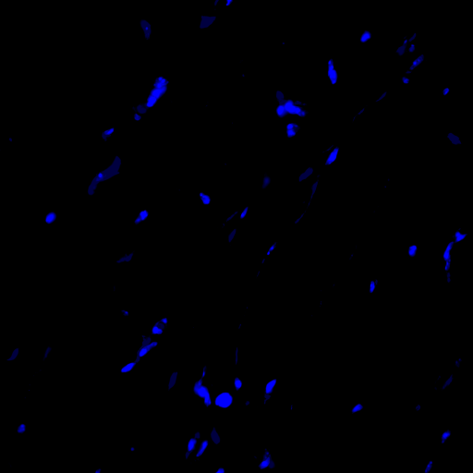

Supplement: Supplementary file 9 — Source data Fig. 7 [file 44321_2025_286_MOESM9_ESM.zip › Figure 7/7C/TUNEL_HSFD+BMS_LE_DAPI.tif]

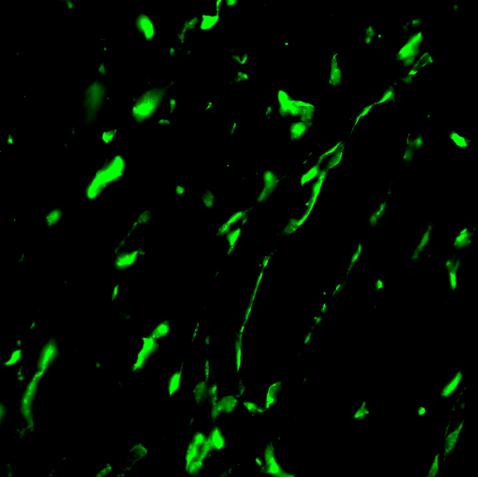

Supplement: Supplementary file 9 — Source data Fig. 7 [file 44321_2025_286_MOESM9_ESM.zip › Figure 7/7C/TUNEL_HSFD+BMS_LE_LYVE-1.tif]

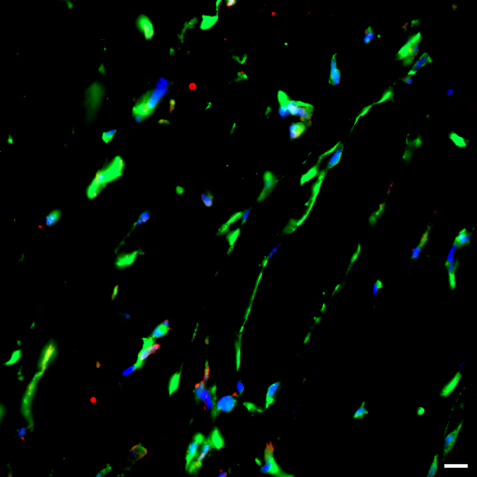

Supplement: Supplementary file 9 — Source data Fig. 7 [file 44321_2025_286_MOESM9_ESM.zip › Figure 7/7C/TUNEL_HSFD+BMS_LE_Merged.tif]

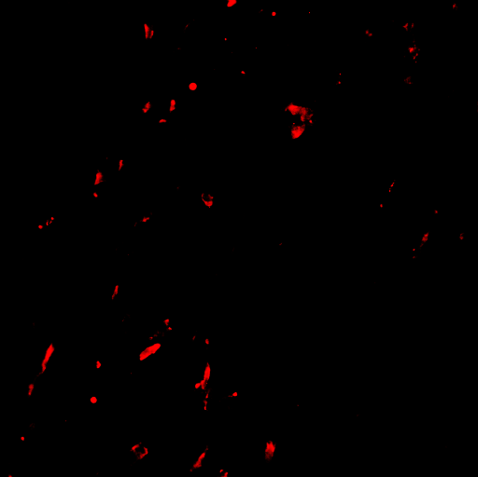

Supplement: Supplementary file 9 — Source data Fig. 7 [file 44321_2025_286_MOESM9_ESM.zip › Figure 7/7C/TUNEL_HSFD+BMS_LE_TUNEL.tif]

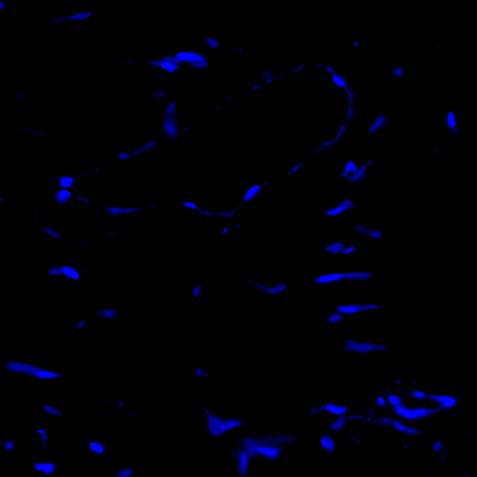

Supplement: Supplementary file 9 — Source data Fig. 7 [file 44321_2025_286_MOESM9_ESM.zip › Figure 7/7C/TUNEL_HSFD+PBS_LE_DAPI.tif]

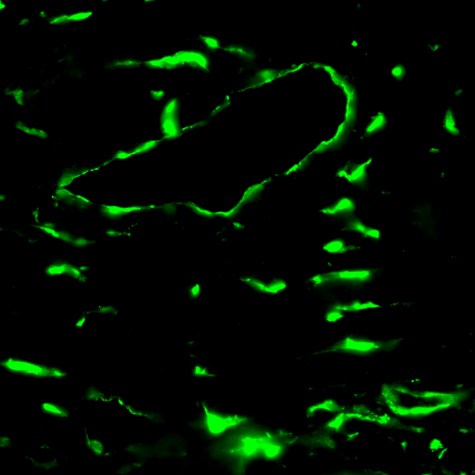

Supplement: Supplementary file 9 — Source data Fig. 7 [file 44321_2025_286_MOESM9_ESM.zip › Figure 7/7C/TUNEL_HSFD+PBS_LE_LYVE-1.tif]

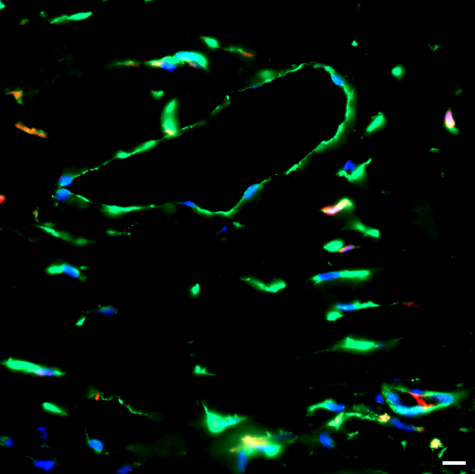

Supplement: Supplementary file 9 — Source data Fig. 7 [file 44321_2025_286_MOESM9_ESM.zip › Figure 7/7C/TUNEL_HSFD+PBS_LE_Merged.tif]

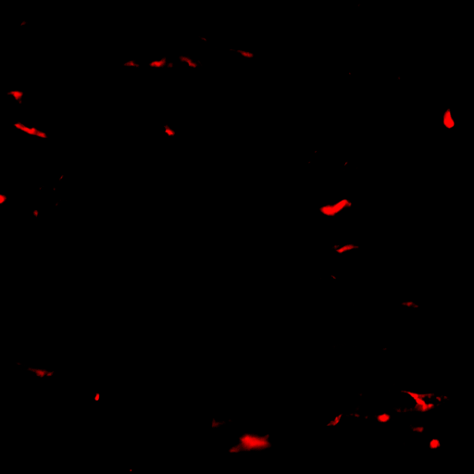

Supplement: Supplementary file 9 — Source data Fig. 7 [file 44321_2025_286_MOESM9_ESM.zip › Figure 7/7C/TUNEL_HSFD+PBS_LE_TUNEL.tif]

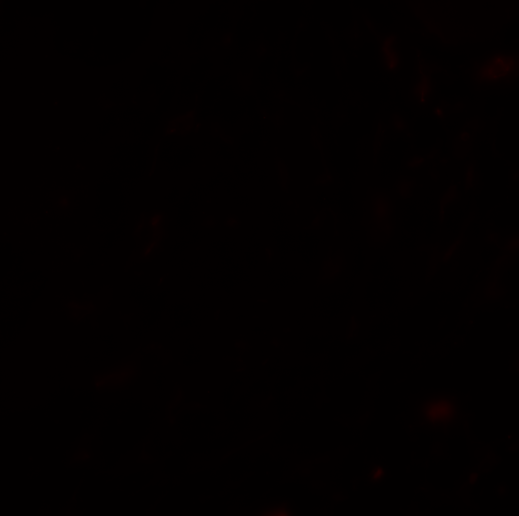

Supplement: Supplementary file 11 — Figure EV 2 Source Data [file 44321_2025_286_MOESM11_ESM.zip › Expanded View Figure 2/2C/CHOP_HFD_LE_CHOP.tif]

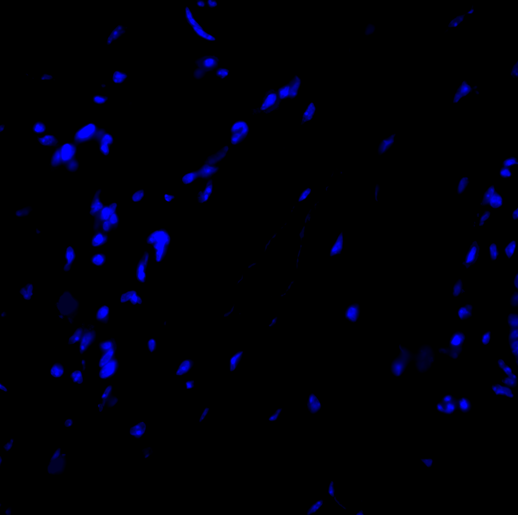

Supplement: Supplementary file 11 — Figure EV 2 Source Data [file 44321_2025_286_MOESM11_ESM.zip › Expanded View Figure 2/2C/CHOP_HFD_LE_DAPI.tif]

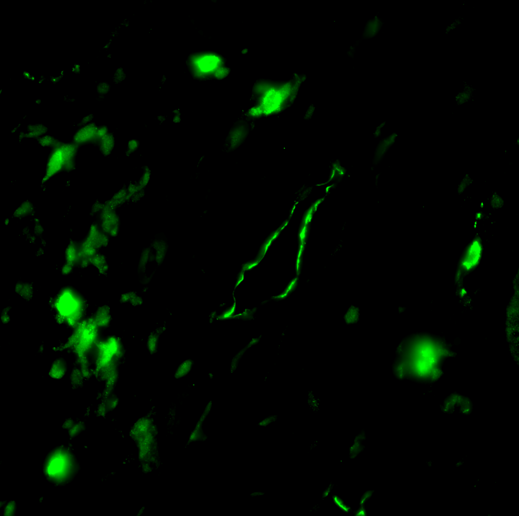

Supplement: Supplementary file 11 — Figure EV 2 Source Data [file 44321_2025_286_MOESM11_ESM.zip › Expanded View Figure 2/2C/CHOP_HFD_LE_LEVY-1.tif]

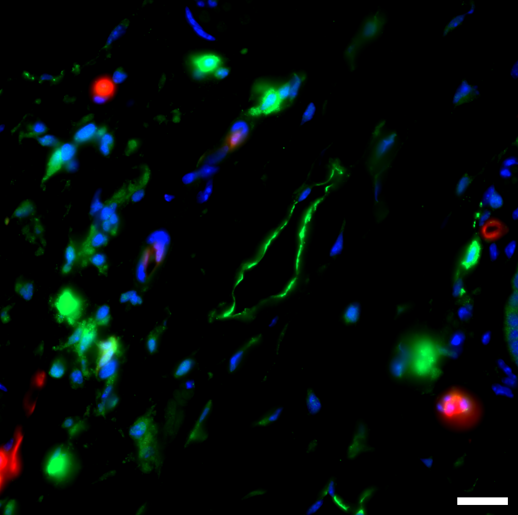

Supplement: Supplementary file 11 — Figure EV 2 Source Data [file 44321_2025_286_MOESM11_ESM.zip › Expanded View Figure 2/2C/CHOP_HFD_LE_Merged.tif]

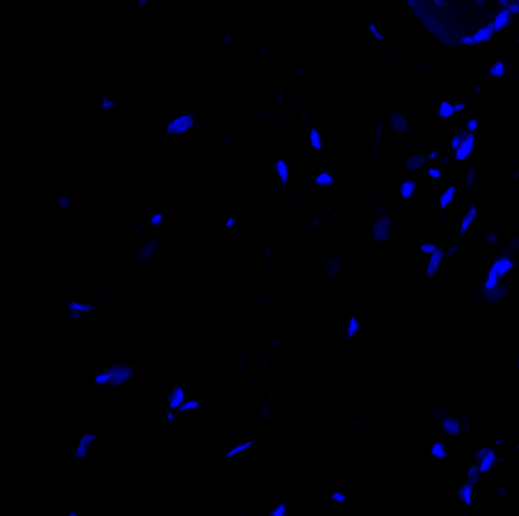

Supplement: Supplementary file 11 — Figure EV 2 Source Data [file 44321_2025_286_MOESM11_ESM.zip › Expanded View Figure 2/2C/CHOP_HFD_Sham_DAPI.tif]

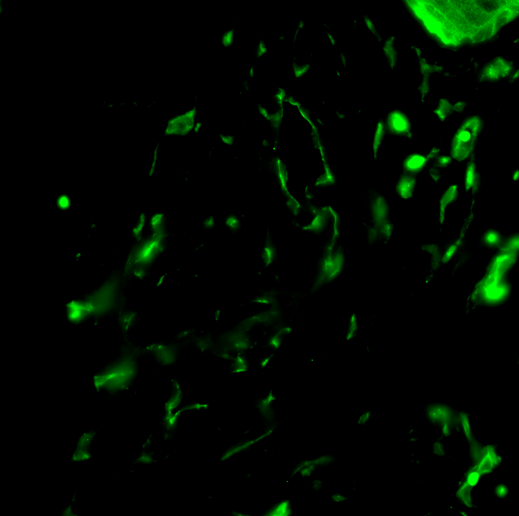

Supplement: Supplementary file 11 — Figure EV 2 Source Data [file 44321_2025_286_MOESM11_ESM.zip › Expanded View Figure 2/2C/CHOP_HFD_Sham_LYVE-1.tif]

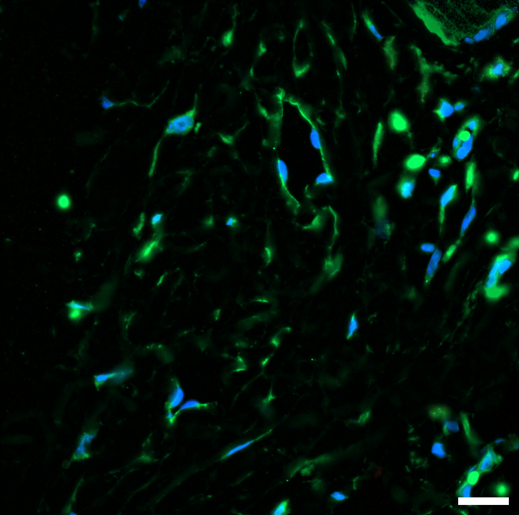

Supplement: Supplementary file 11 — Figure EV 2 Source Data [file 44321_2025_286_MOESM11_ESM.zip › Expanded View Figure 2/2C/CHOP_HFD_Sham_Merged.tif]

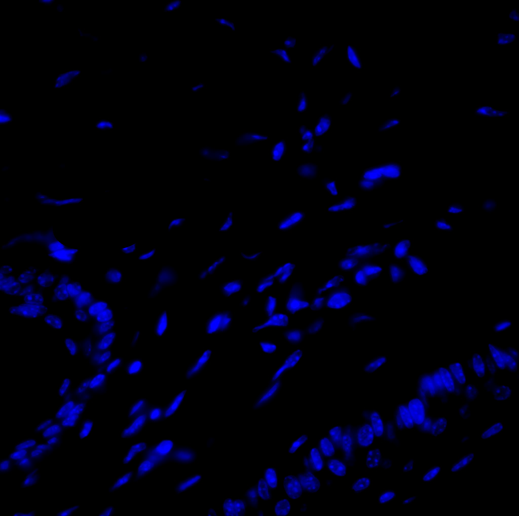

Supplement: Supplementary file 11 — Figure EV 2 Source Data [file 44321_2025_286_MOESM11_ESM.zip › Expanded View Figure 2/2C/sXBP-1_HFD_LE_DAPI.tif]

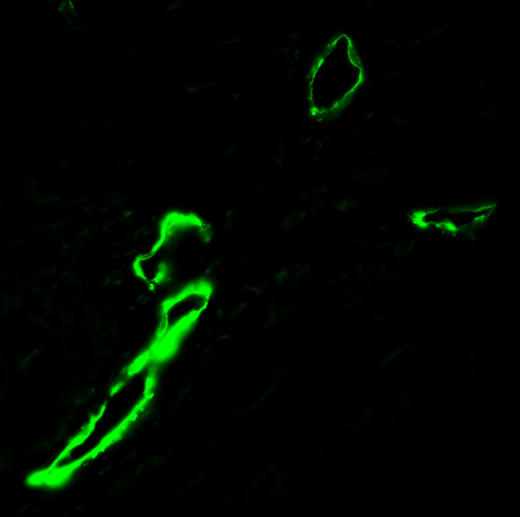

Supplement: Supplementary file 11 — Figure EV 2 Source Data [file 44321_2025_286_MOESM11_ESM.zip › Expanded View Figure 2/2C/sXBP-1_HFD_LE_LYVE-1.tif]

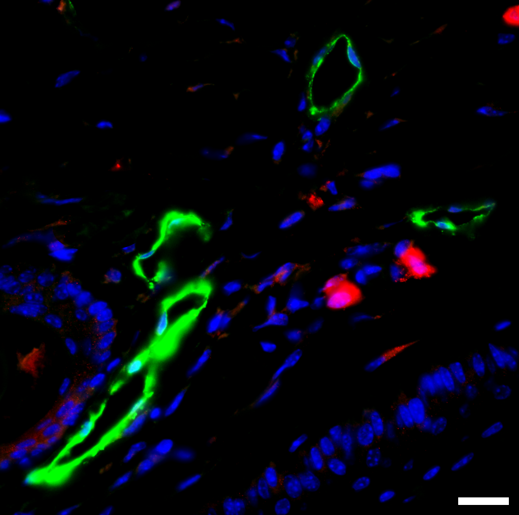

Supplement: Supplementary file 11 — Figure EV 2 Source Data [file 44321_2025_286_MOESM11_ESM.zip › Expanded View Figure 2/2C/sXBP-1_HFD_LE_Merged.tif]

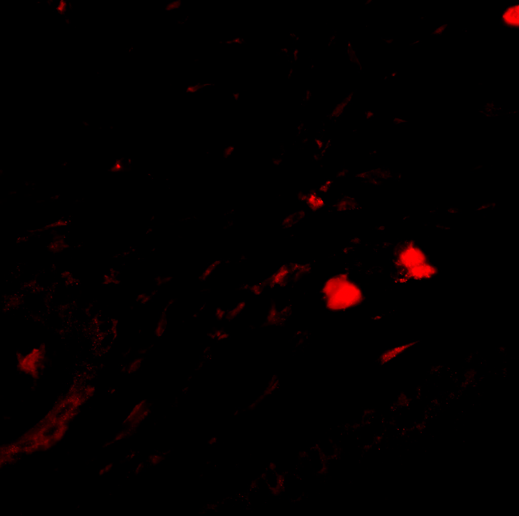

Supplement: Supplementary file 11 — Figure EV 2 Source Data [file 44321_2025_286_MOESM11_ESM.zip › Expanded View Figure 2/2C/sXBP-1_HFD_LE_sXBP-1.tif]

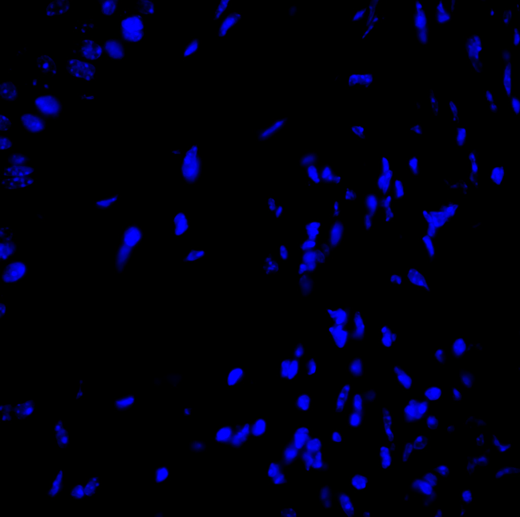

Supplement: Supplementary file 11 — Figure EV 2 Source Data [file 44321_2025_286_MOESM11_ESM.zip › Expanded View Figure 2/2C/sXBP-1_HFD_Sham_DAPI.tif]

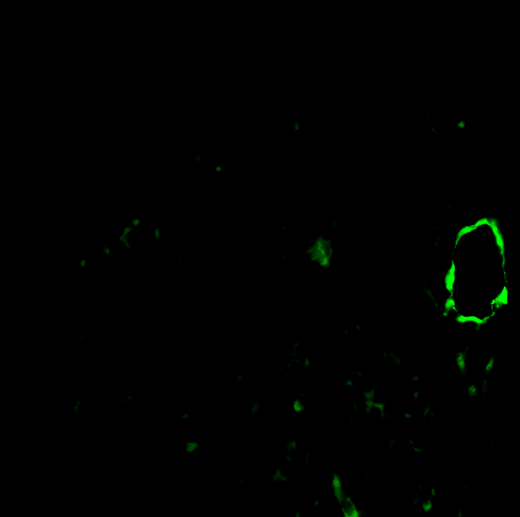

Supplement: Supplementary file 11 — Figure EV 2 Source Data [file 44321_2025_286_MOESM11_ESM.zip › Expanded View Figure 2/2C/sXBP-1_HFD_Sham_LYVE-1.tif]

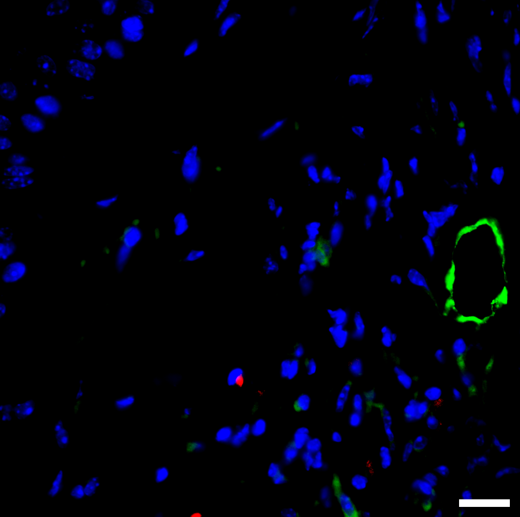

Supplement: Supplementary file 11 — Figure EV 2 Source Data [file 44321_2025_286_MOESM11_ESM.zip › Expanded View Figure 2/2C/sXBP-1_HFD_Sham_Merged.tif]

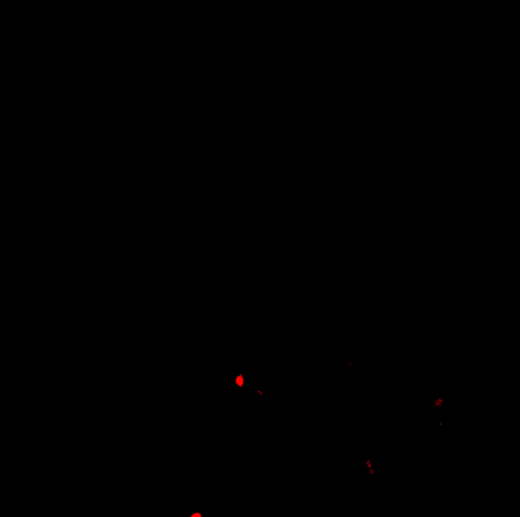

Supplement: Supplementary file 11 — Figure EV 2 Source Data [file 44321_2025_286_MOESM11_ESM.zip › Expanded View Figure 2/2C/sXBP-1_HFD_Sham_sXBP-1.tif]

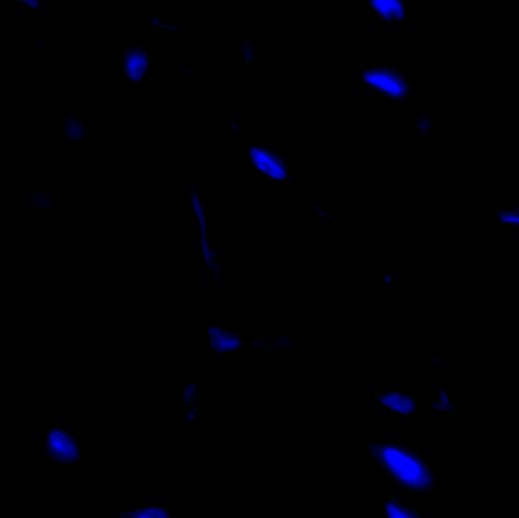

Supplement: Supplementary file 11 — Figure EV 2 Source Data [file 44321_2025_286_MOESM11_ESM.zip › Expanded View Figure 2/2C/TUNEL_HFD_LE_DAPI.tif]

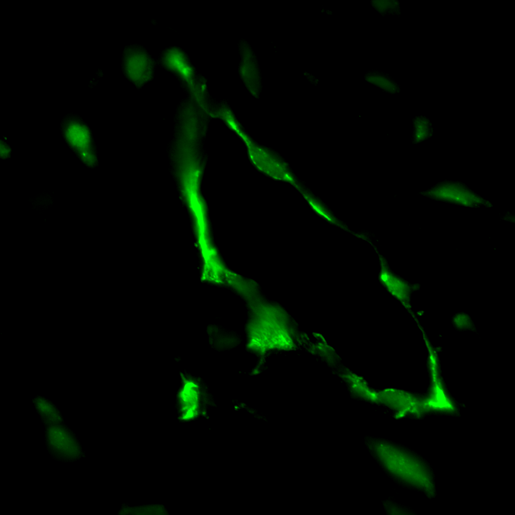

Supplement: Supplementary file 11 — Figure EV 2 Source Data [file 44321_2025_286_MOESM11_ESM.zip › Expanded View Figure 2/2C/TUNEL_HFD_LE_LYVE-1.tif]

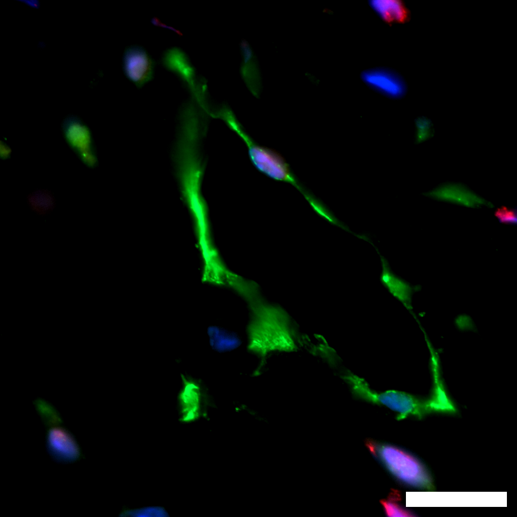

Supplement: Supplementary file 11 — Figure EV 2 Source Data [file 44321_2025_286_MOESM11_ESM.zip › Expanded View Figure 2/2C/TUNEL_HFD_LE_Merged.tif]

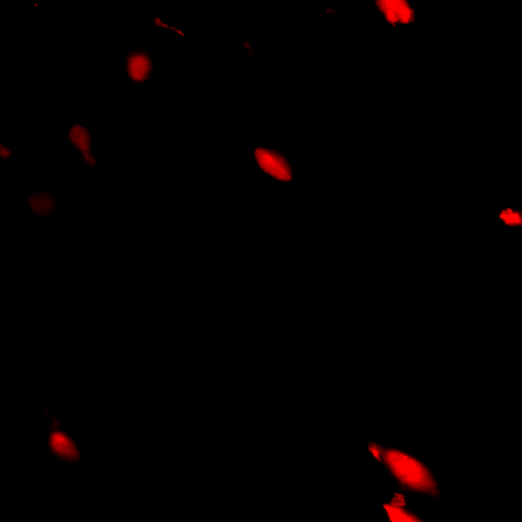

Supplement: Supplementary file 11 — Figure EV 2 Source Data [file 44321_2025_286_MOESM11_ESM.zip › Expanded View Figure 2/2C/TUNEL_HFD_LE_TUNEL.tif]

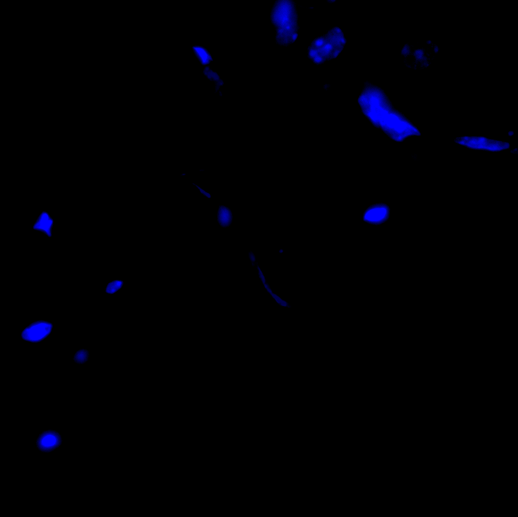

Supplement: Supplementary file 11 — Figure EV 2 Source Data [file 44321_2025_286_MOESM11_ESM.zip › Expanded View Figure 2/2C/TUNEL_HFD_Sham_DAPI.tif]

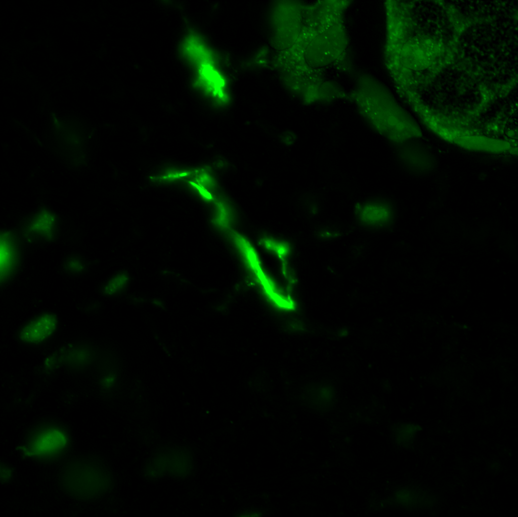

Supplement: Supplementary file 11 — Figure EV 2 Source Data [file 44321_2025_286_MOESM11_ESM.zip › Expanded View Figure 2/2C/TUNEL_HFD_Sham_LYVE-1.tif]

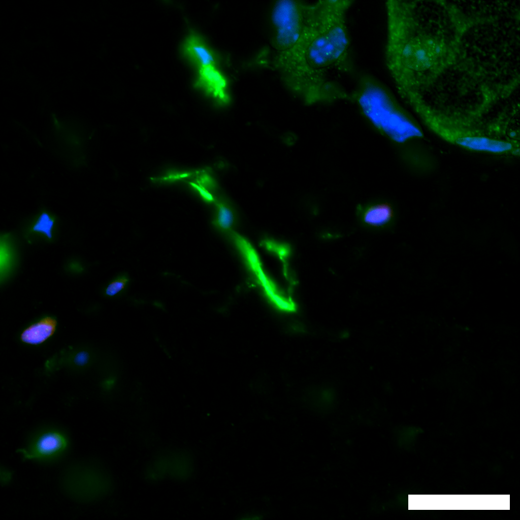

Supplement: Supplementary file 11 — Figure EV 2 Source Data [file 44321_2025_286_MOESM11_ESM.zip › Expanded View Figure 2/2C/TUNEL_HFD_Sham_Merged.tif]

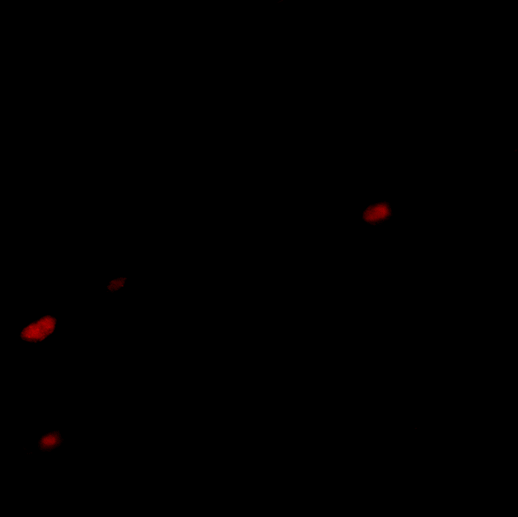

Supplement: Supplementary file 11 — Figure EV 2 Source Data [file 44321_2025_286_MOESM11_ESM.zip › Expanded View Figure 2/2C/TUNEL_HFD_Sham_TUNEL.tif]

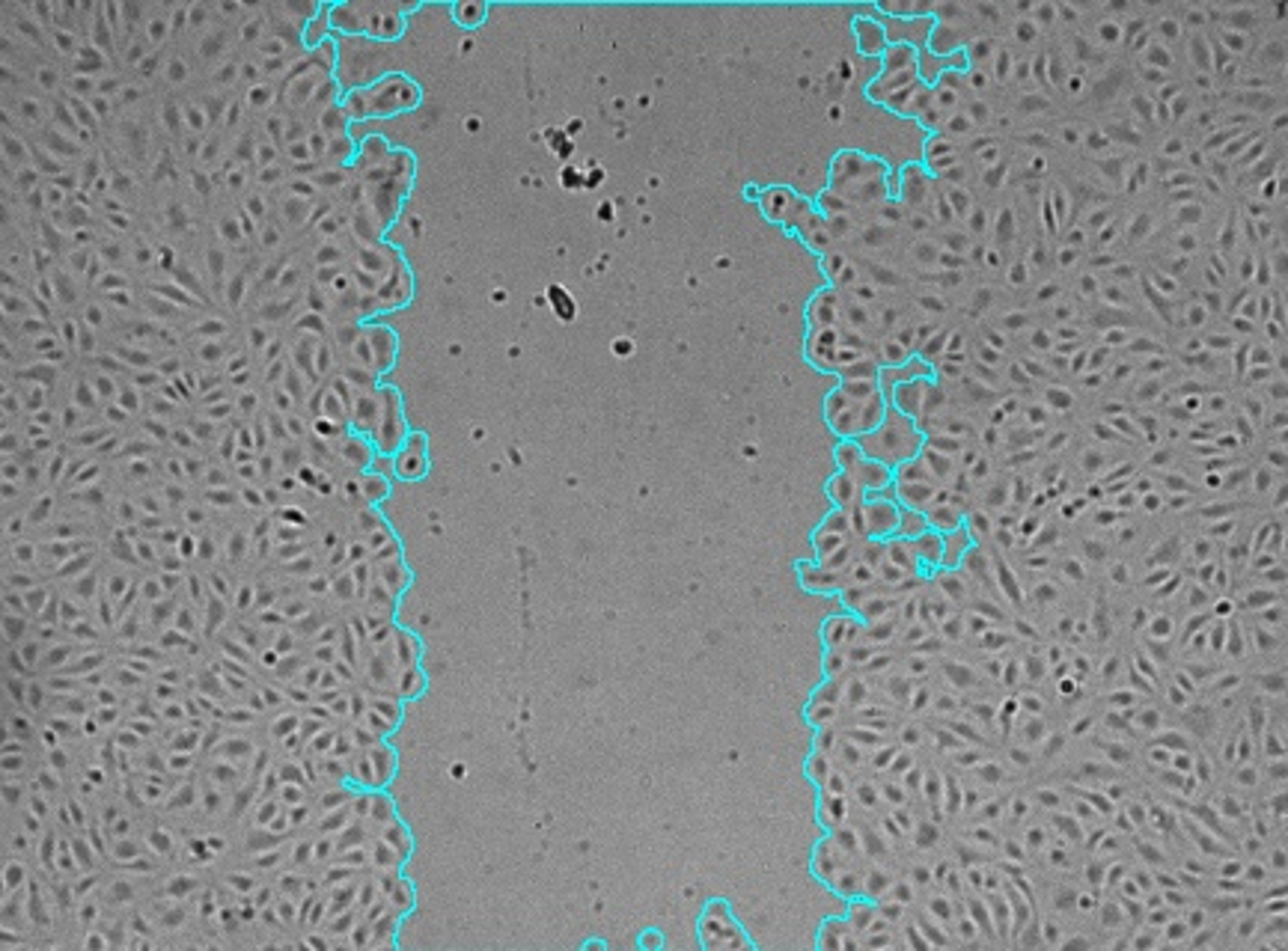

Supplement: Supplementary file 12 — Figure EV 3 Source Data [file 44321_2025_286_MOESM12_ESM.zip › Expanded View Figure 3/3A/HDLEC SA 0h.tif]

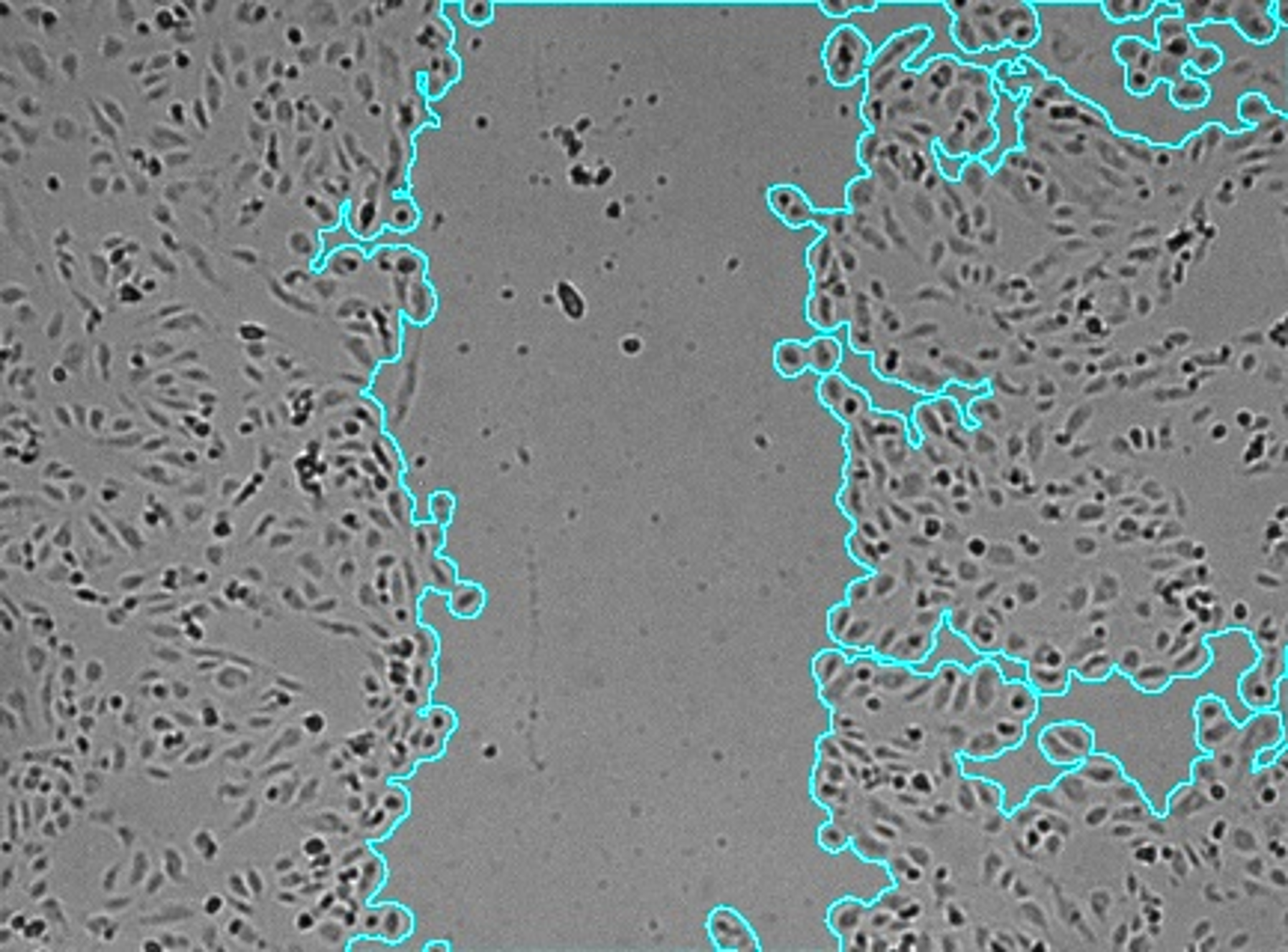

Supplement: Supplementary file 12 — Figure EV 3 Source Data [file 44321_2025_286_MOESM12_ESM.zip › Expanded View Figure 3/3A/HDLEC SA 16h.tif]

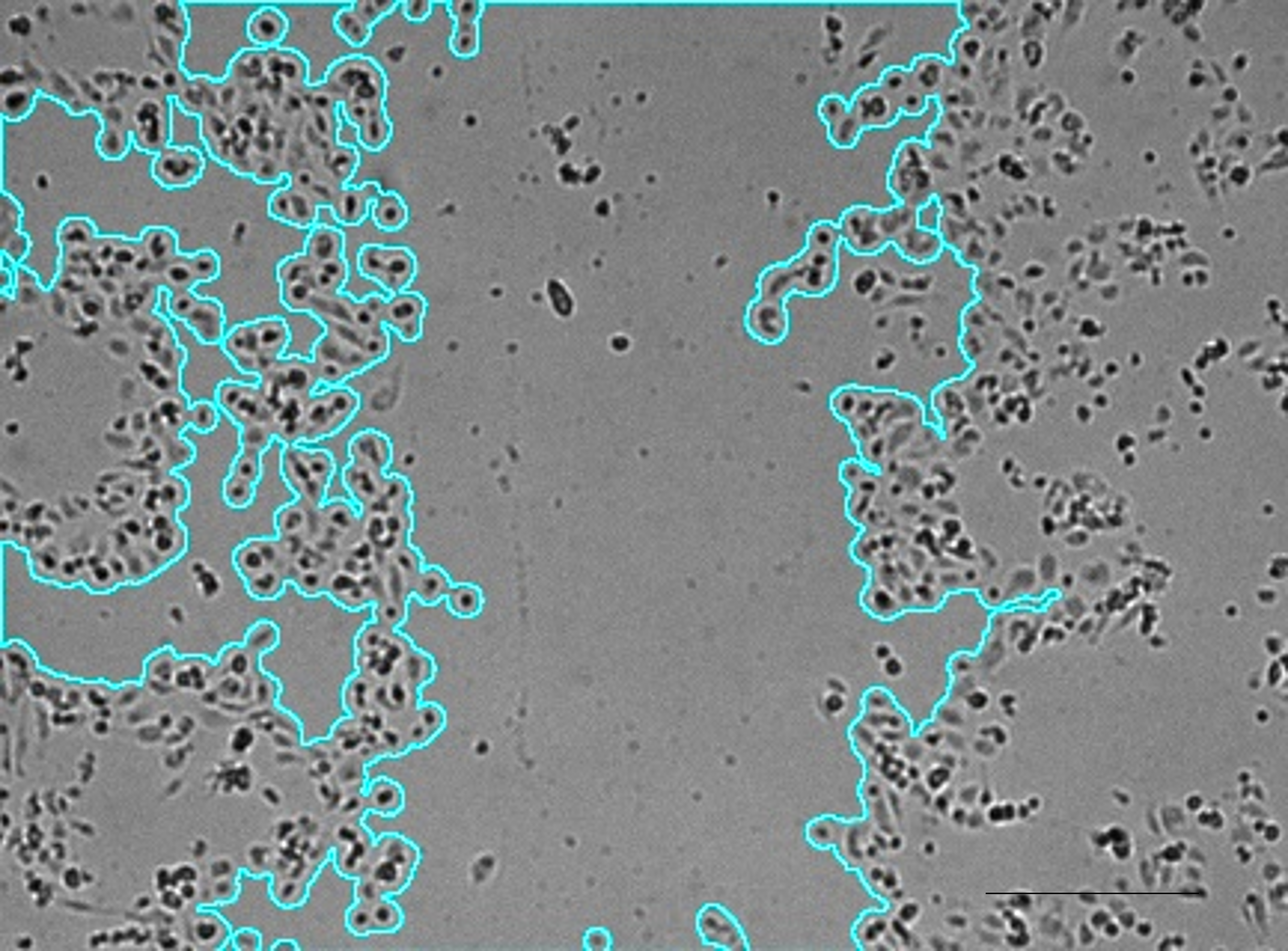

Supplement: Supplementary file 12 — Figure EV 3 Source Data [file 44321_2025_286_MOESM12_ESM.zip › Expanded View Figure 3/3A/HDLEC SA 32h.tif]

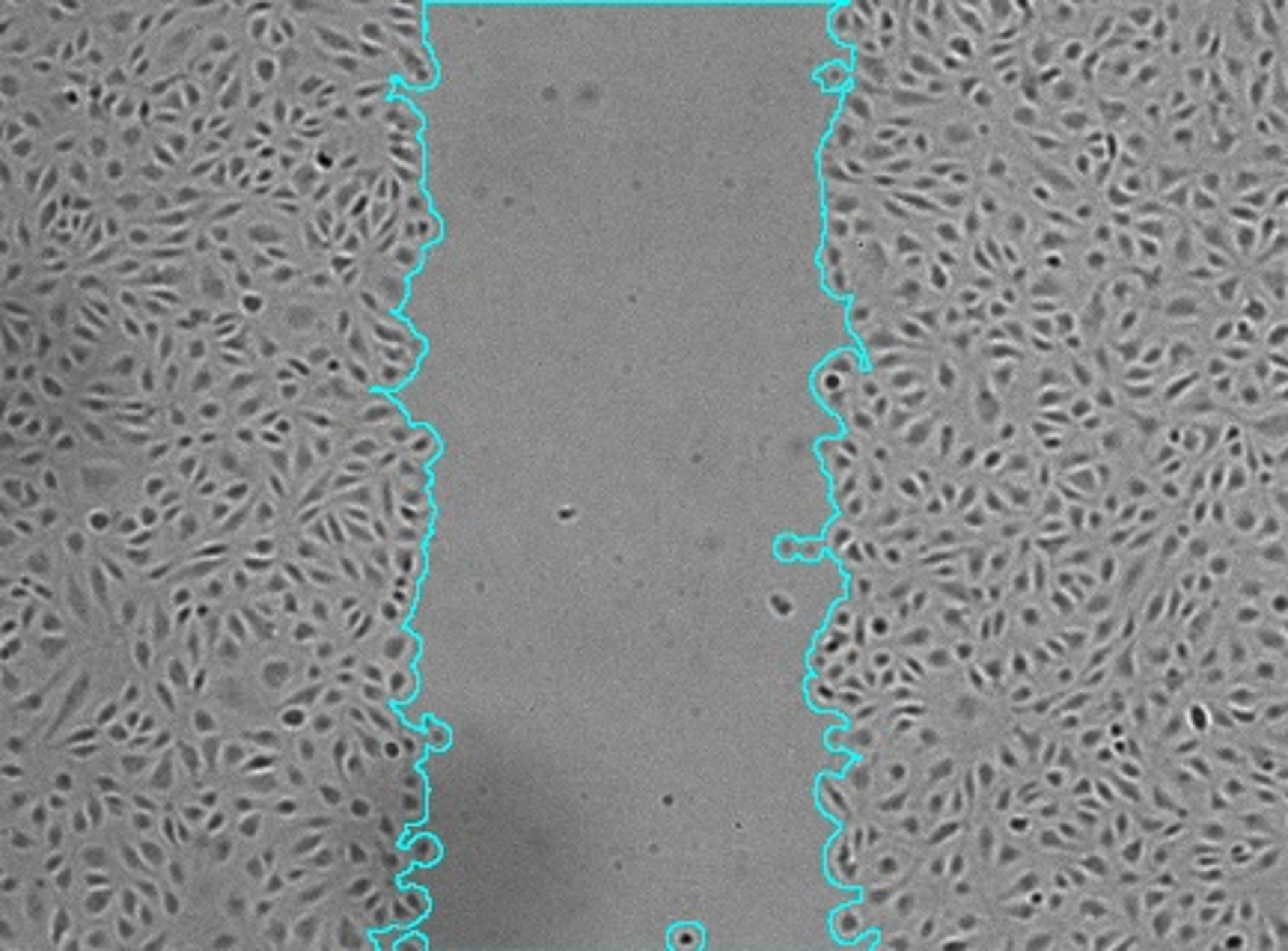

Supplement: Supplementary file 12 — Figure EV 3 Source Data [file 44321_2025_286_MOESM12_ESM.zip › Expanded View Figure 3/3A/HDLEC Vehicle 0h.tif]

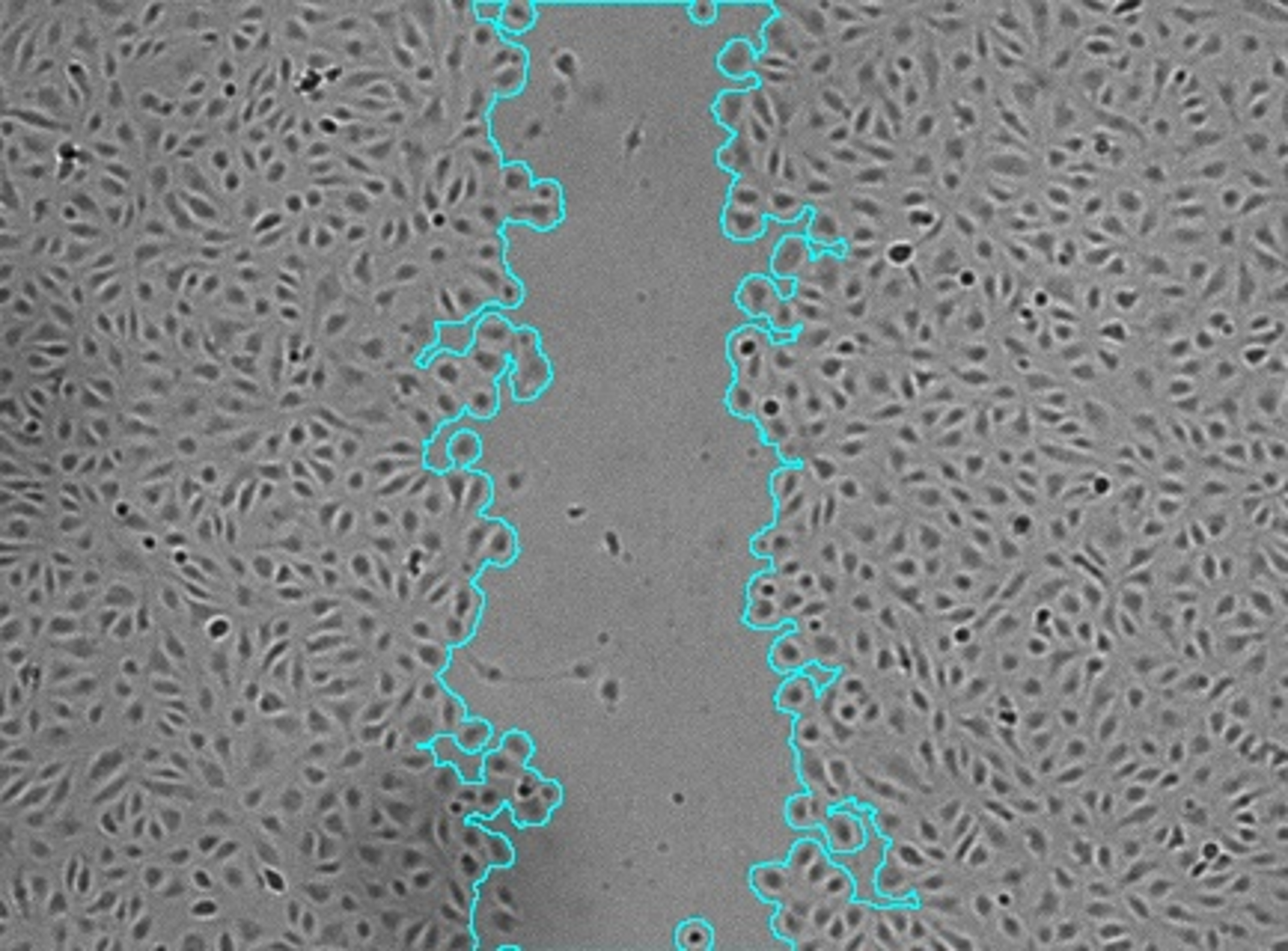

Supplement: Supplementary file 12 — Figure EV 3 Source Data [file 44321_2025_286_MOESM12_ESM.zip › Expanded View Figure 3/3A/HDLEC Vehicle 16h.tif]

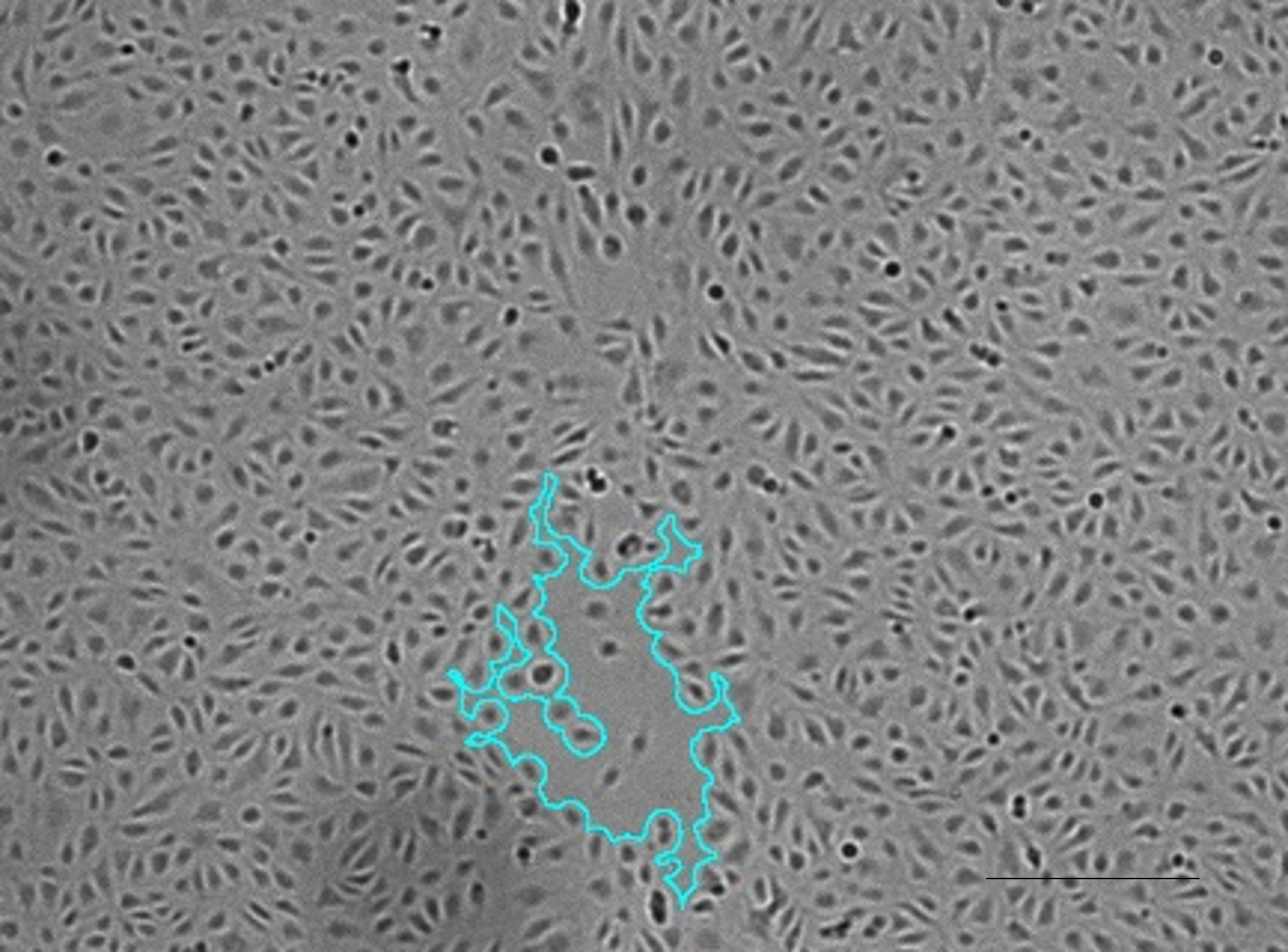

Supplement: Supplementary file 12 — Figure EV 3 Source Data [file 44321_2025_286_MOESM12_ESM.zip › Expanded View Figure 3/3A/HDLEC Vehicle 32h.tif]

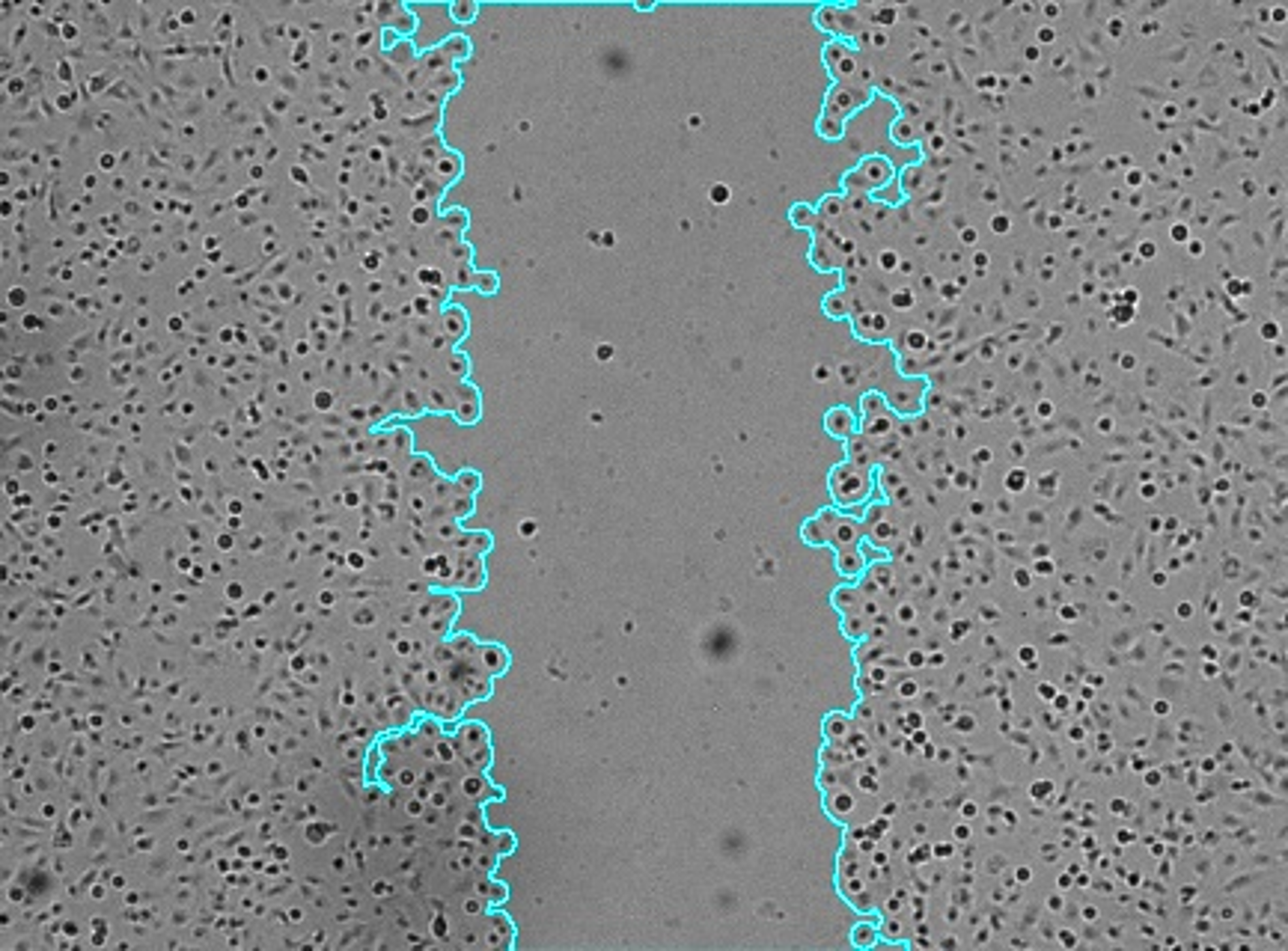

Supplement: Supplementary file 12 — Figure EV 3 Source Data [file 44321_2025_286_MOESM12_ESM.zip › Expanded View Figure 3/3C/HDMEC SA 0h.tif]

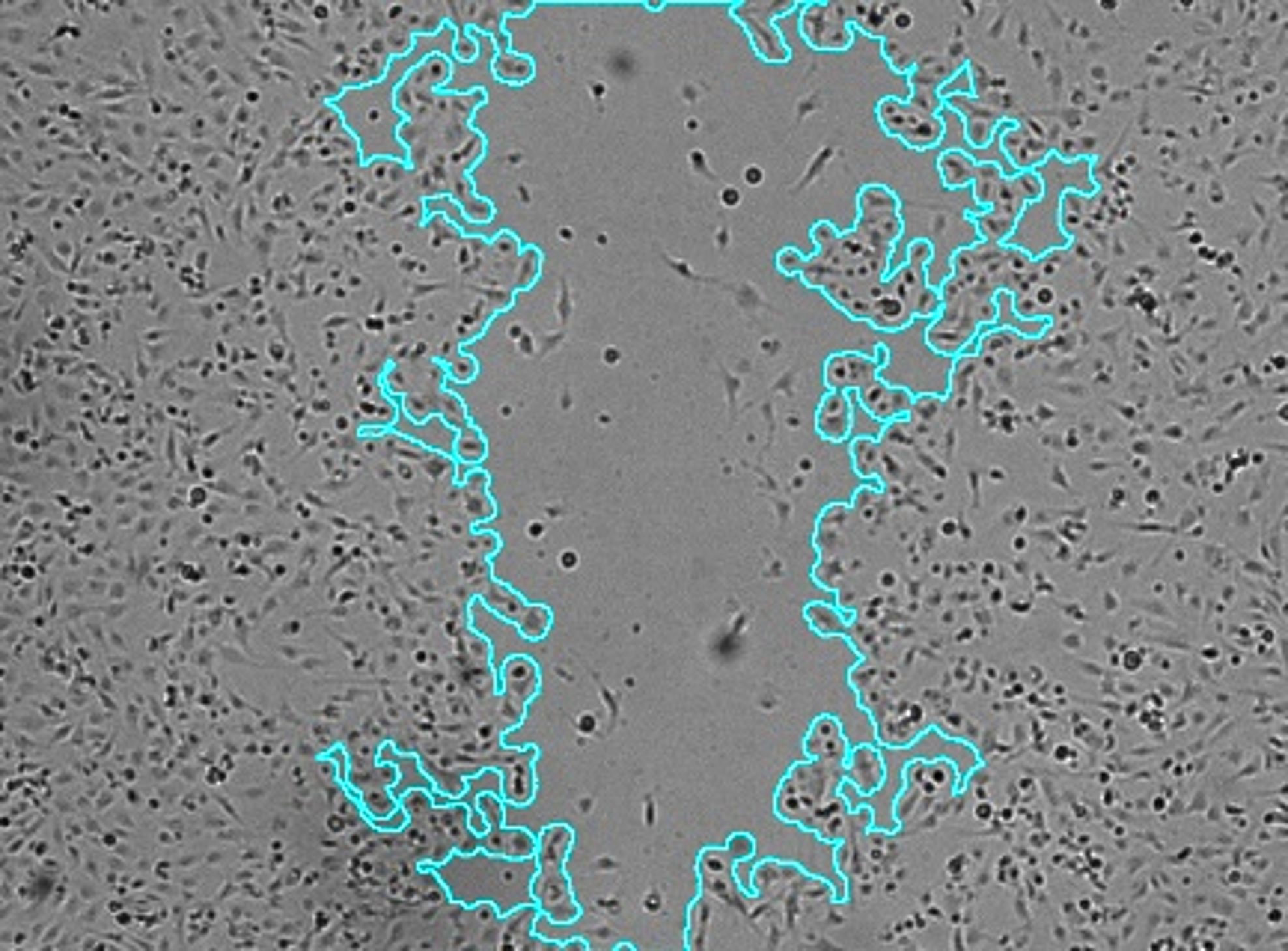

Supplement: Supplementary file 12 — Figure EV 3 Source Data [file 44321_2025_286_MOESM12_ESM.zip › Expanded View Figure 3/3C/HDMEC SA 16h.tif]

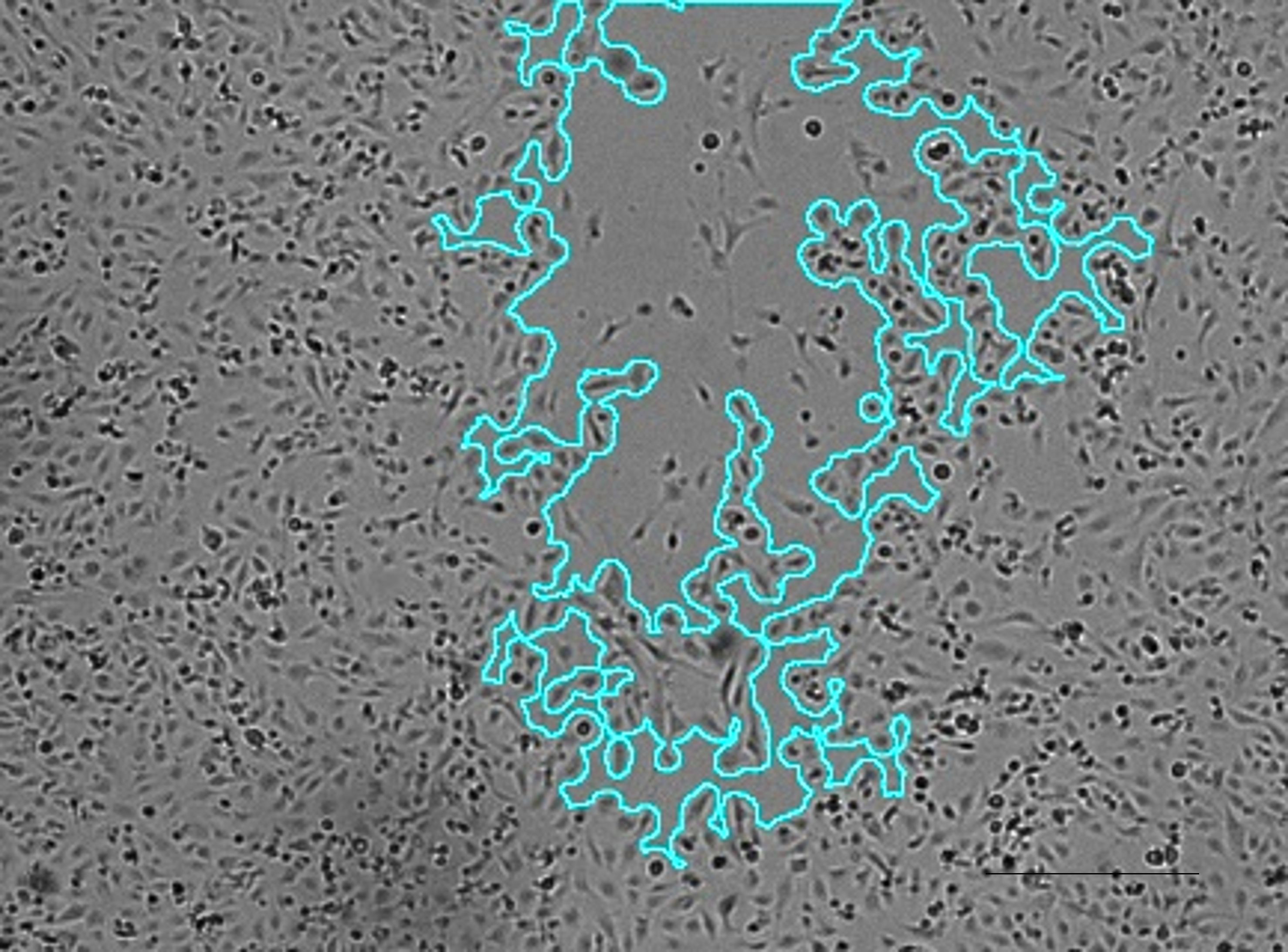

Supplement: Supplementary file 12 — Figure EV 3 Source Data [file 44321_2025_286_MOESM12_ESM.zip › Expanded View Figure 3/3C/HDMEC SA 32h.tif]

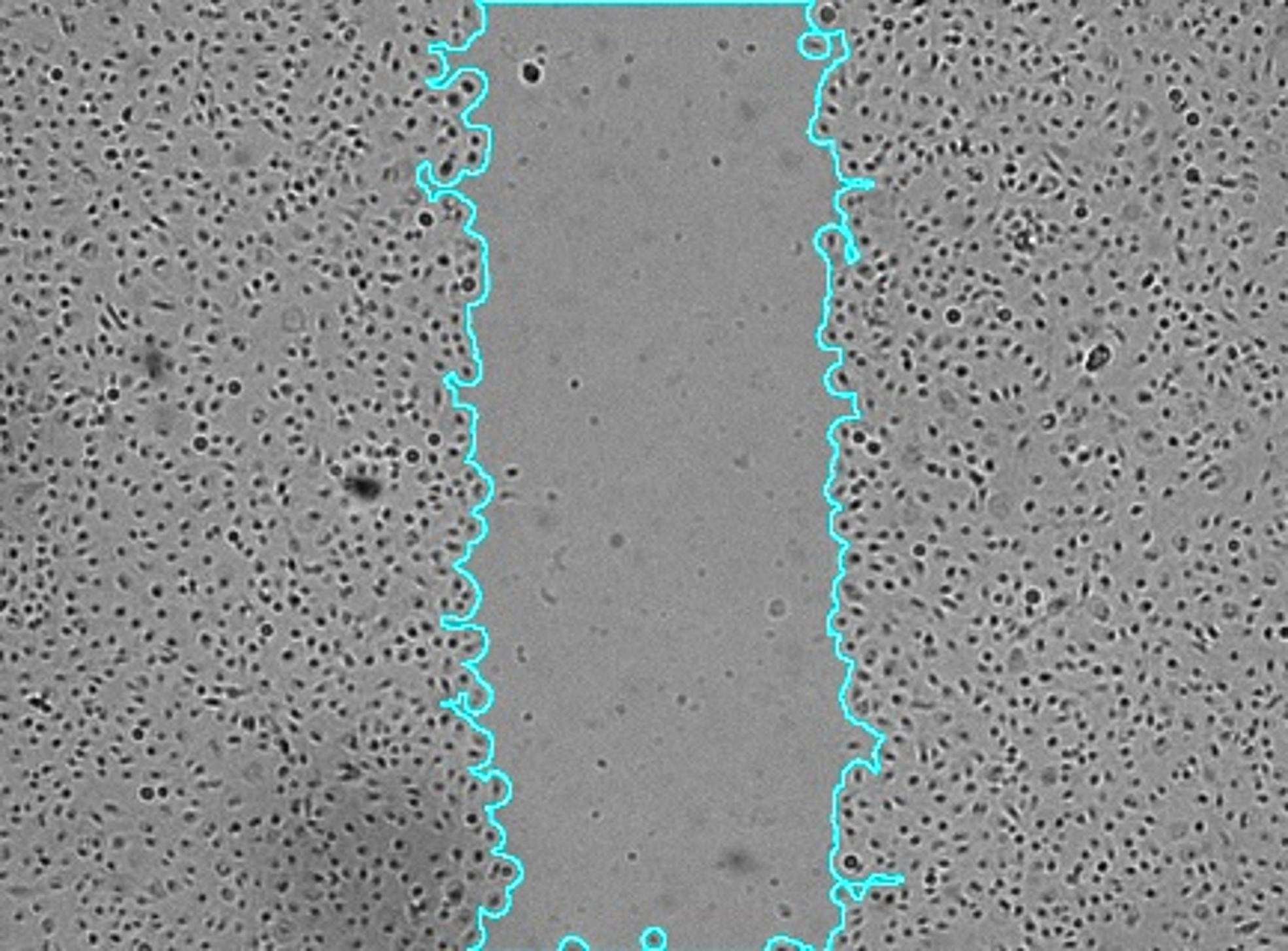

Supplement: Supplementary file 12 — Figure EV 3 Source Data [file 44321_2025_286_MOESM12_ESM.zip › Expanded View Figure 3/3C/HDMEC Vehicle 0h.tif]

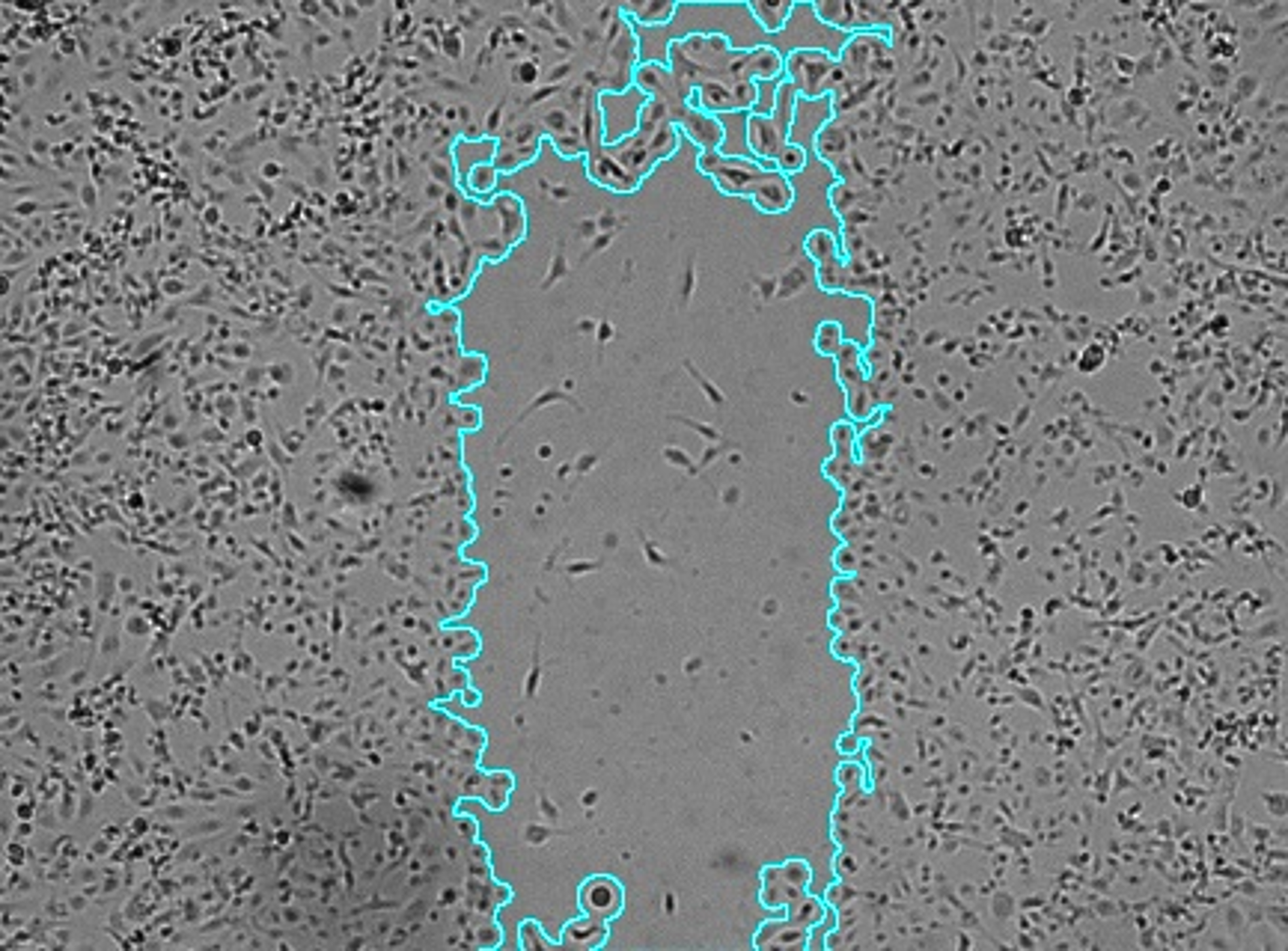

Supplement: Supplementary file 12 — Figure EV 3 Source Data [file 44321_2025_286_MOESM12_ESM.zip › Expanded View Figure 3/3C/HDMEC Vehicle 16h.tif]

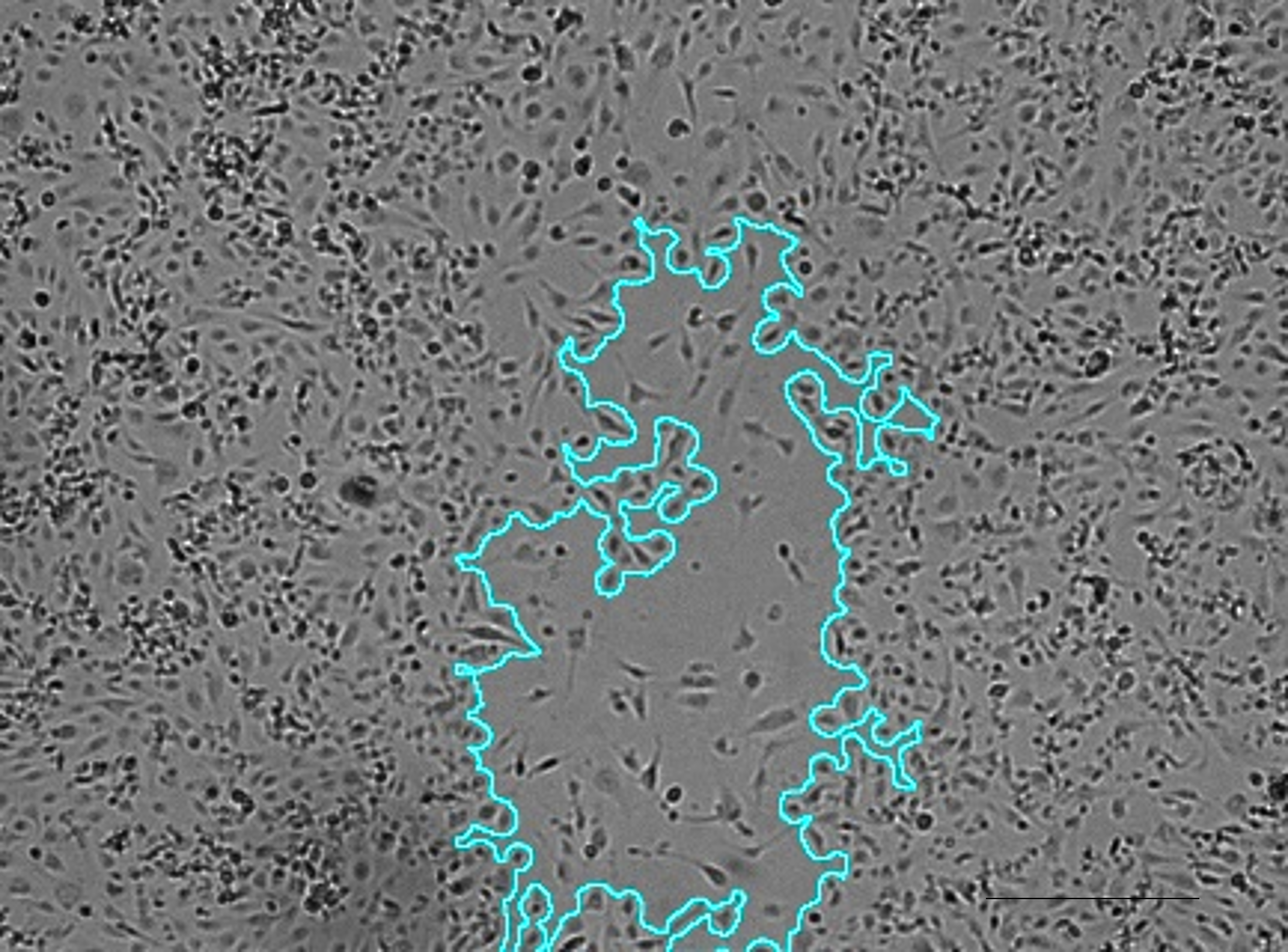

Supplement: Supplementary file 12 — Figure EV 3 Source Data [file 44321_2025_286_MOESM12_ESM.zip › Expanded View Figure 3/3C/HDMEC Vehicle 32h.tif]

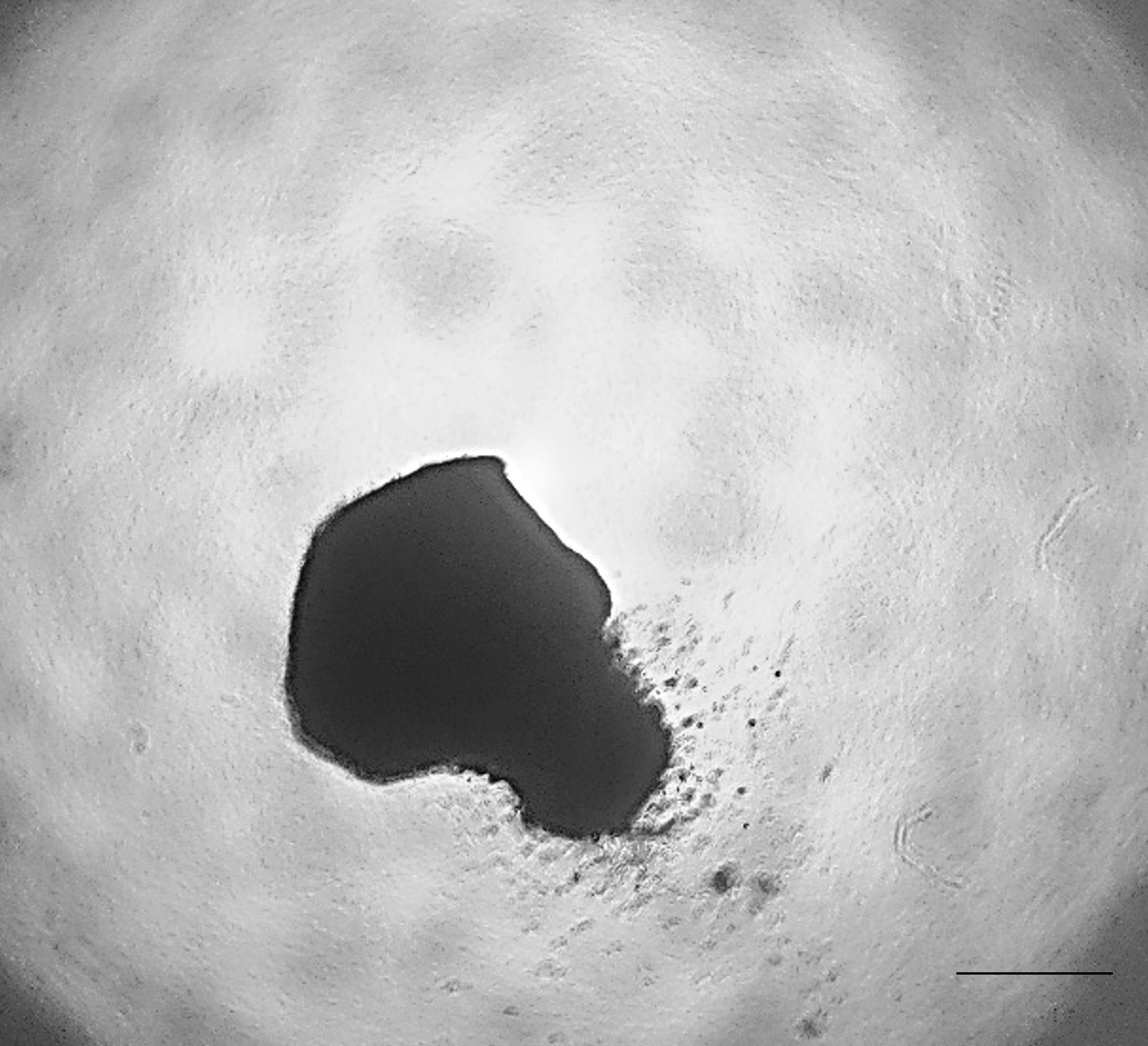

Supplement: Supplementary file 12 — Figure EV 3 Source Data [file 44321_2025_286_MOESM12_ESM.zip › Expanded View Figure 3/3E/SA 1.tif]

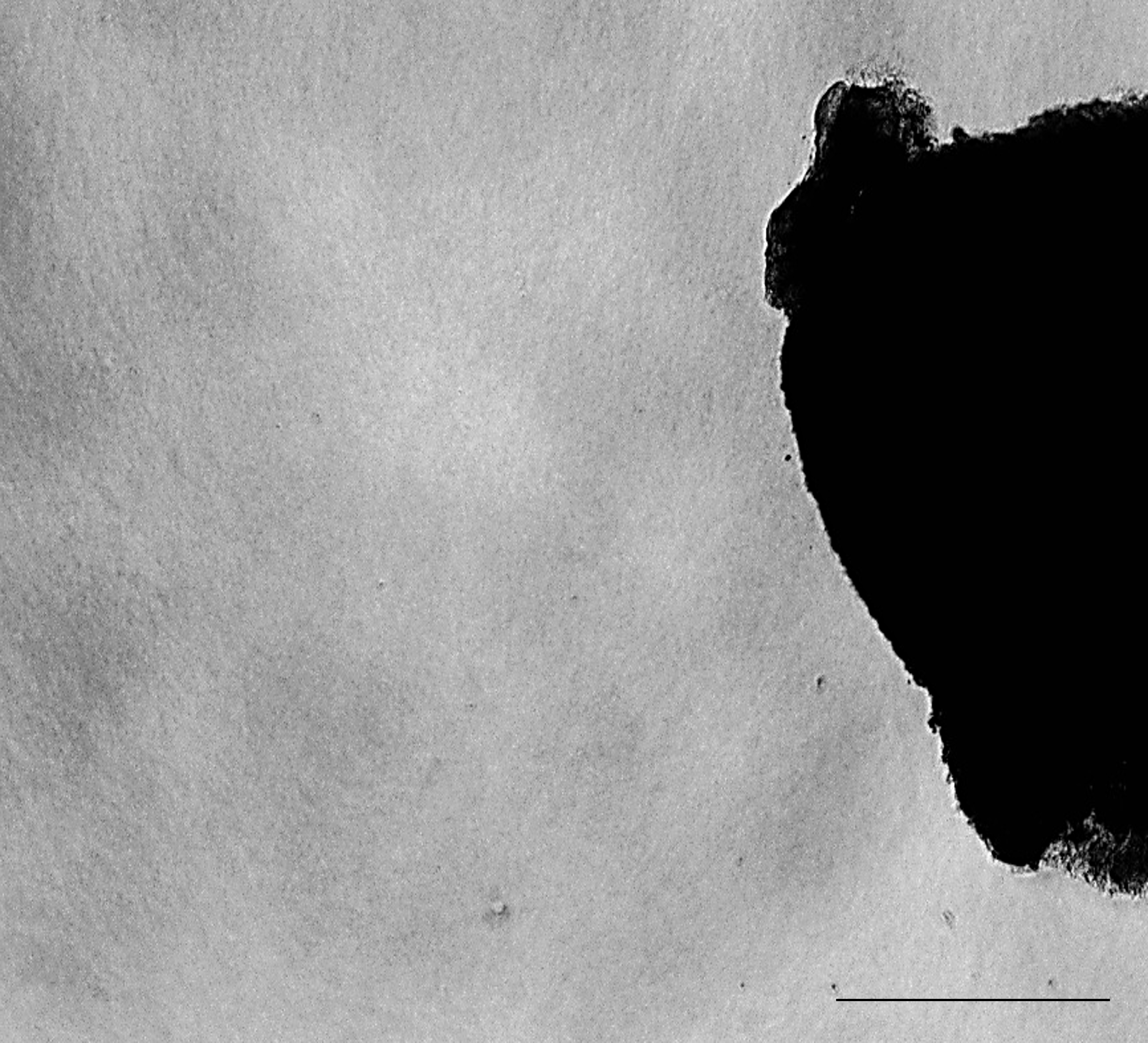

Supplement: Supplementary file 12 — Figure EV 3 Source Data [file 44321_2025_286_MOESM12_ESM.zip › Expanded View Figure 3/3E/SA 2.tif]

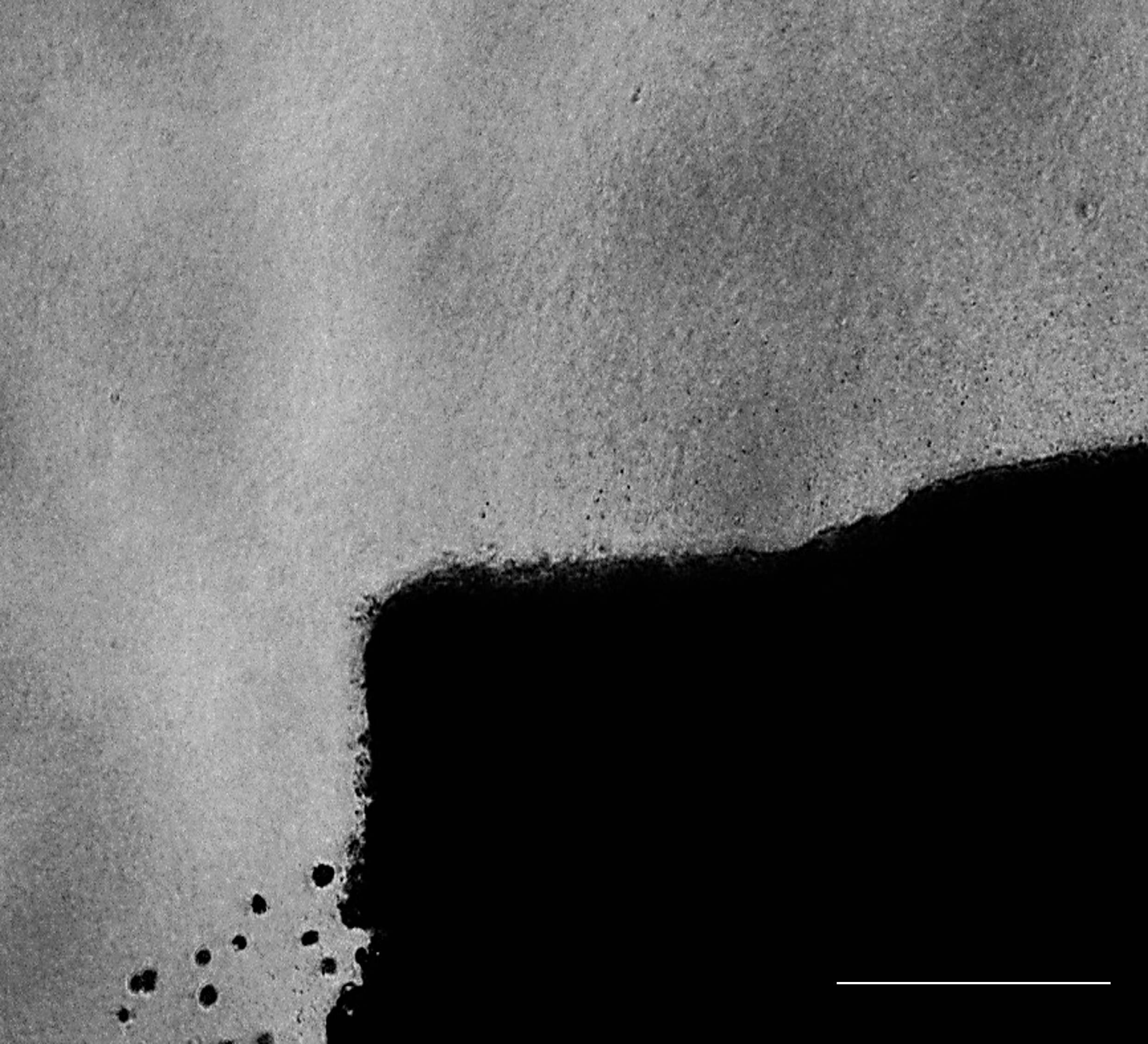

Supplement: Supplementary file 12 — Figure EV 3 Source Data [file 44321_2025_286_MOESM12_ESM.zip › Expanded View Figure 3/3E/SA 3.tif]

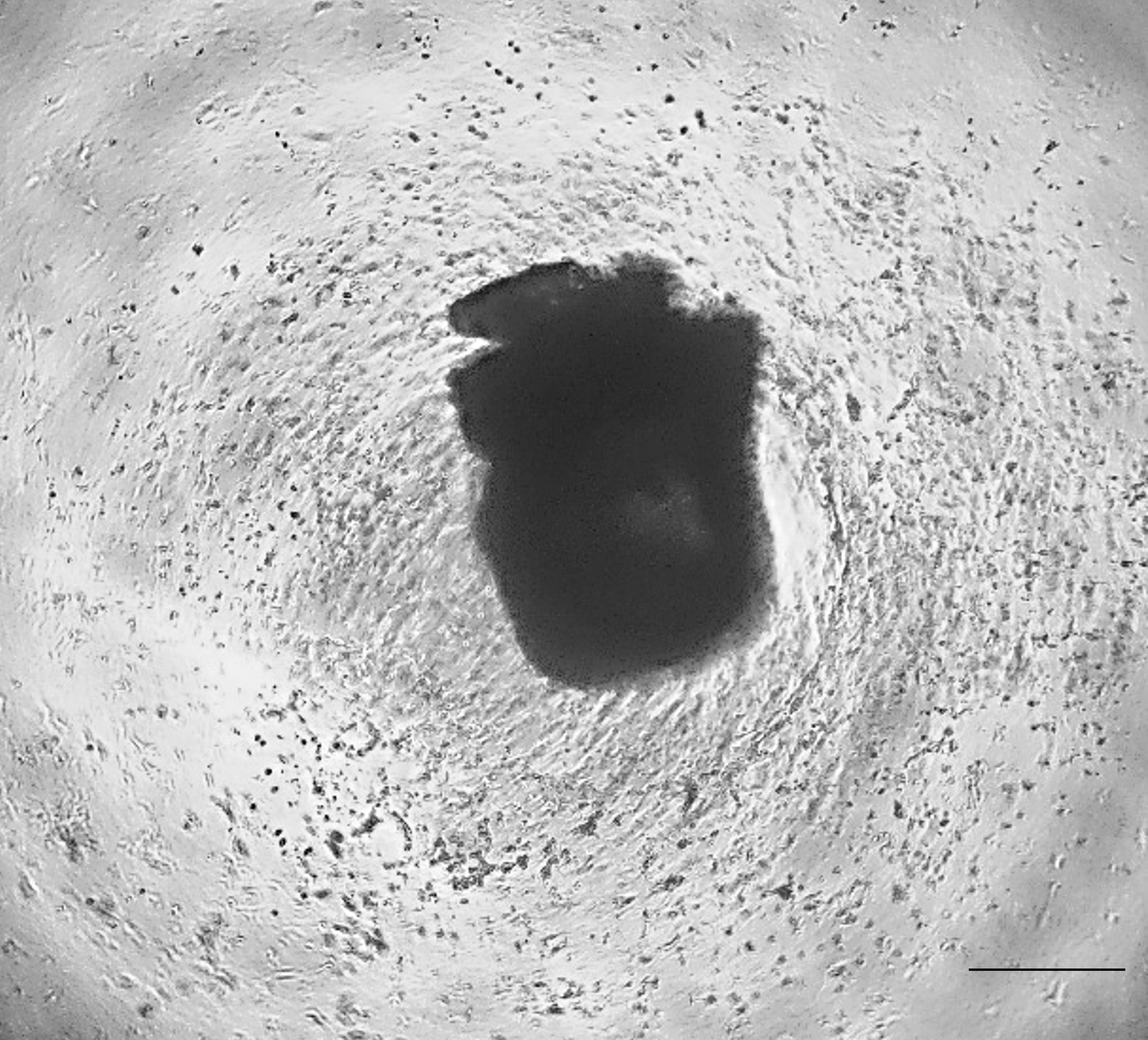

Supplement: Supplementary file 12 — Figure EV 3 Source Data [file 44321_2025_286_MOESM12_ESM.zip › Expanded View Figure 3/3E/Vehicle 1.tif]

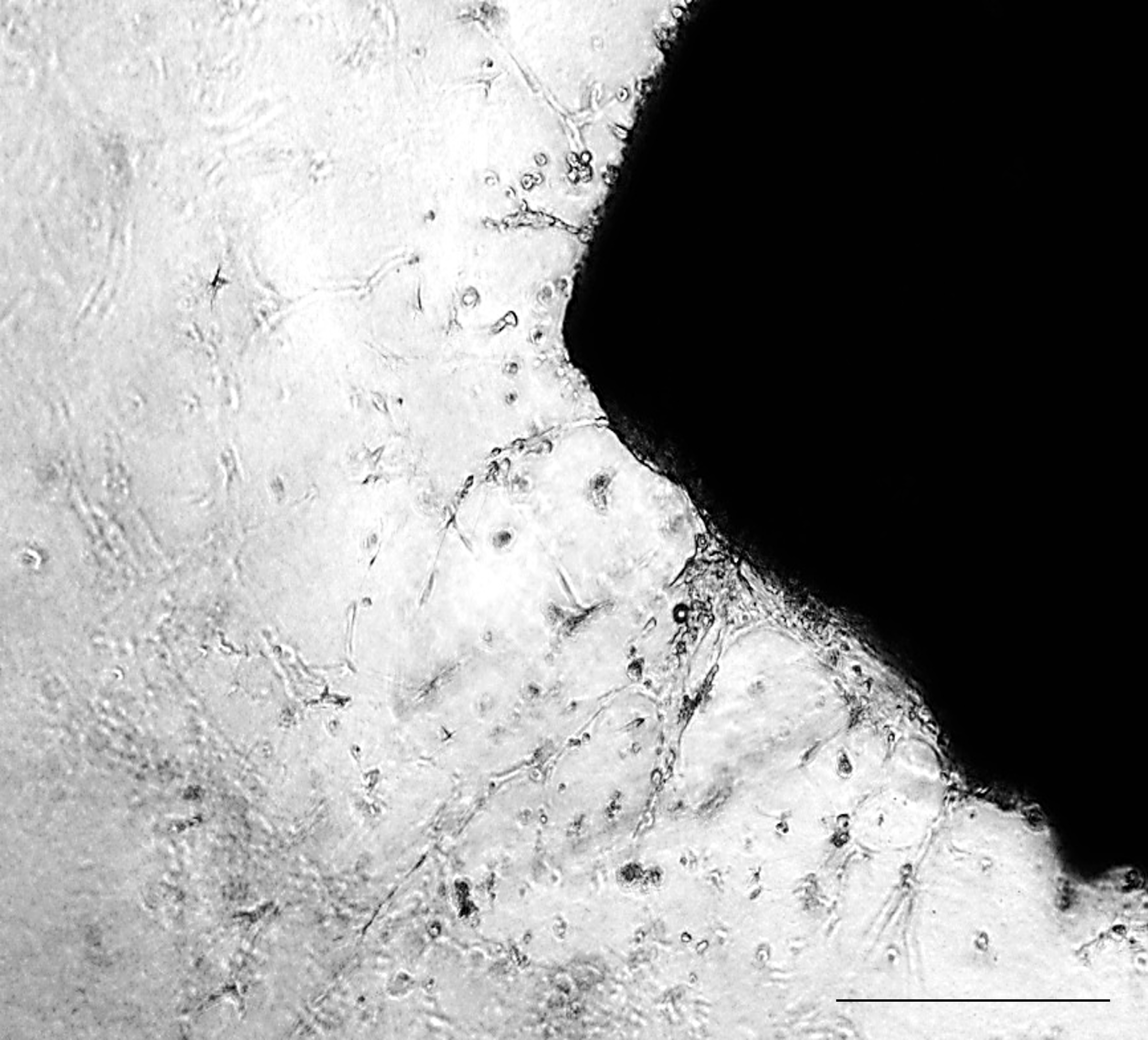

Supplement: Supplementary file 12 — Figure EV 3 Source Data [file 44321_2025_286_MOESM12_ESM.zip › Expanded View Figure 3/3E/Vehicle 2.tif]

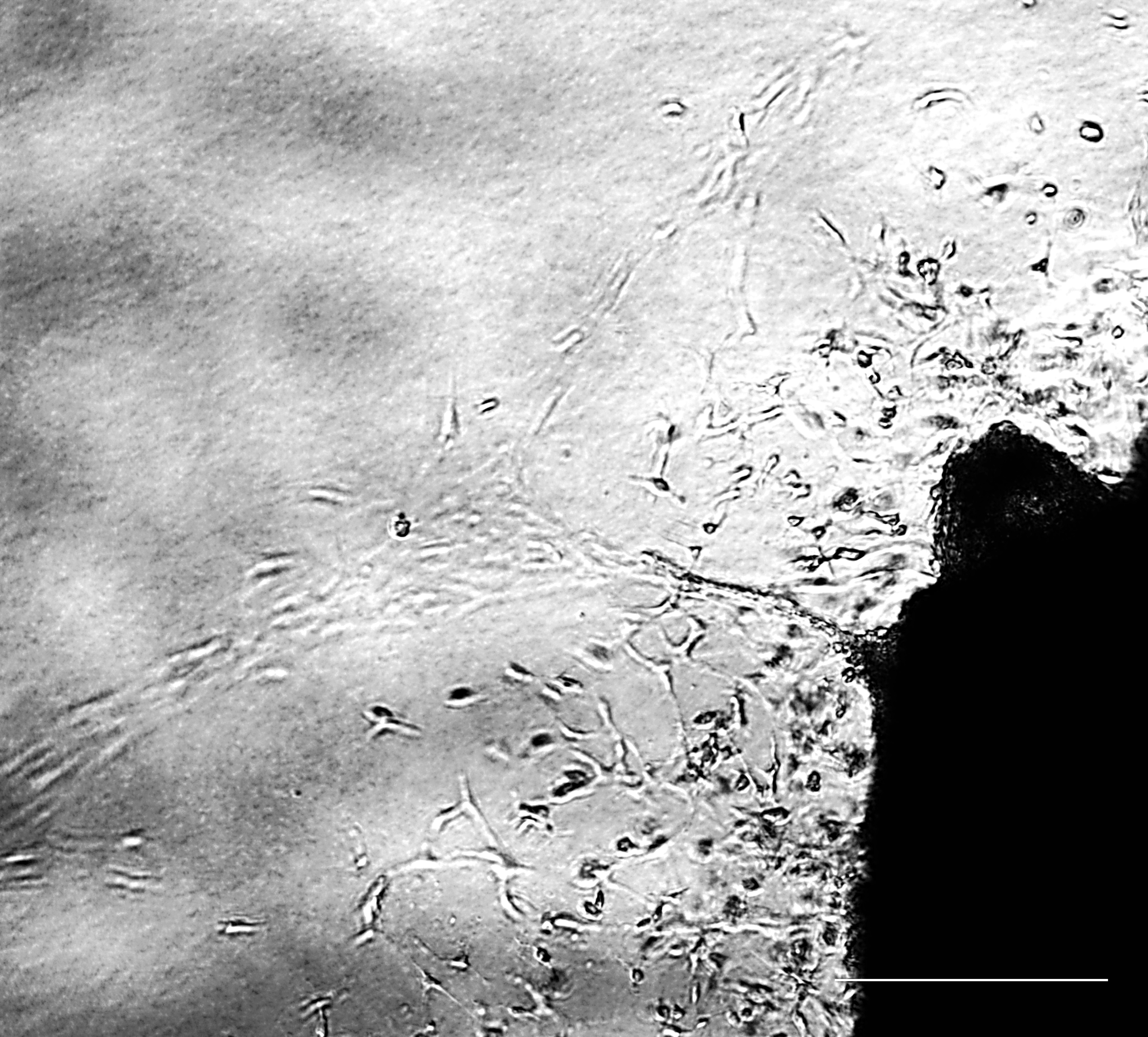

Supplement: Supplementary file 12 — Figure EV 3 Source Data [file 44321_2025_286_MOESM12_ESM.zip › Expanded View Figure 3/3E/Vehicle 3.tif]

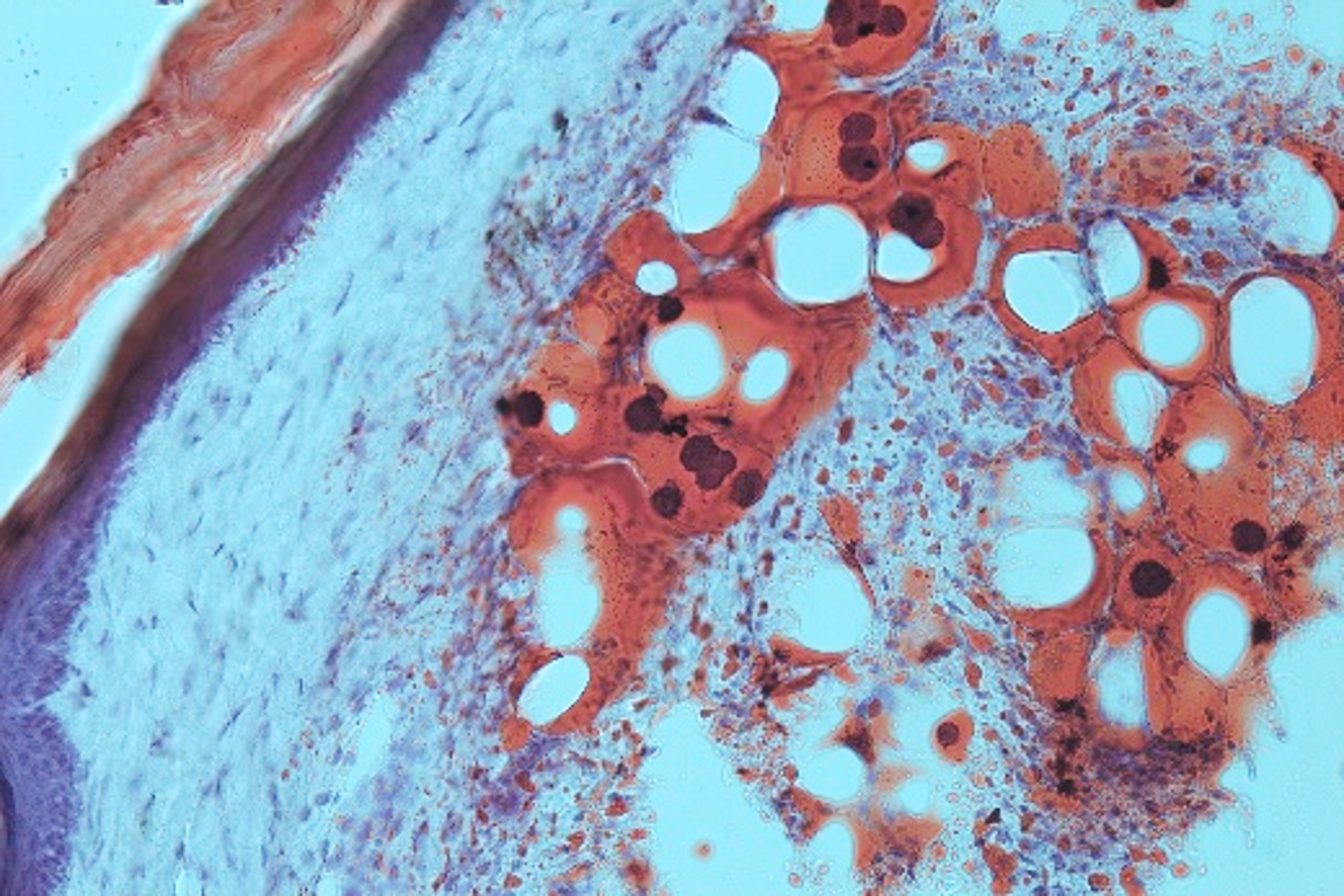

Supplement: Supplementary file 13 — Figure EV 4 Source Data [file 44321_2025_286_MOESM13_ESM.zip › Expanded View Figure 4/4A/HSFD Lymphedema.tif]

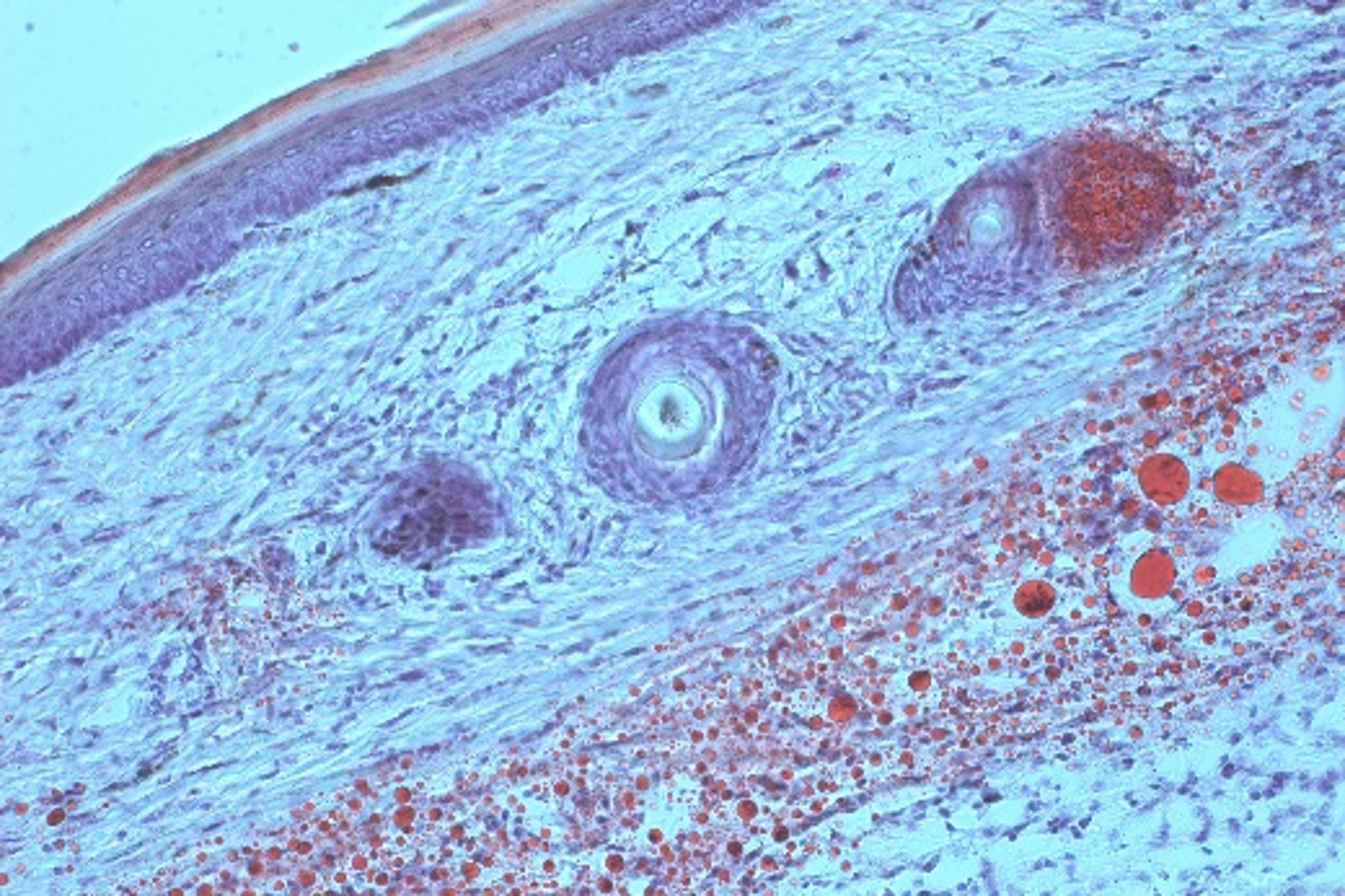

Supplement: Supplementary file 13 — Figure EV 4 Source Data [file 44321_2025_286_MOESM13_ESM.zip › Expanded View Figure 4/4A/HSFD Sham.tif]

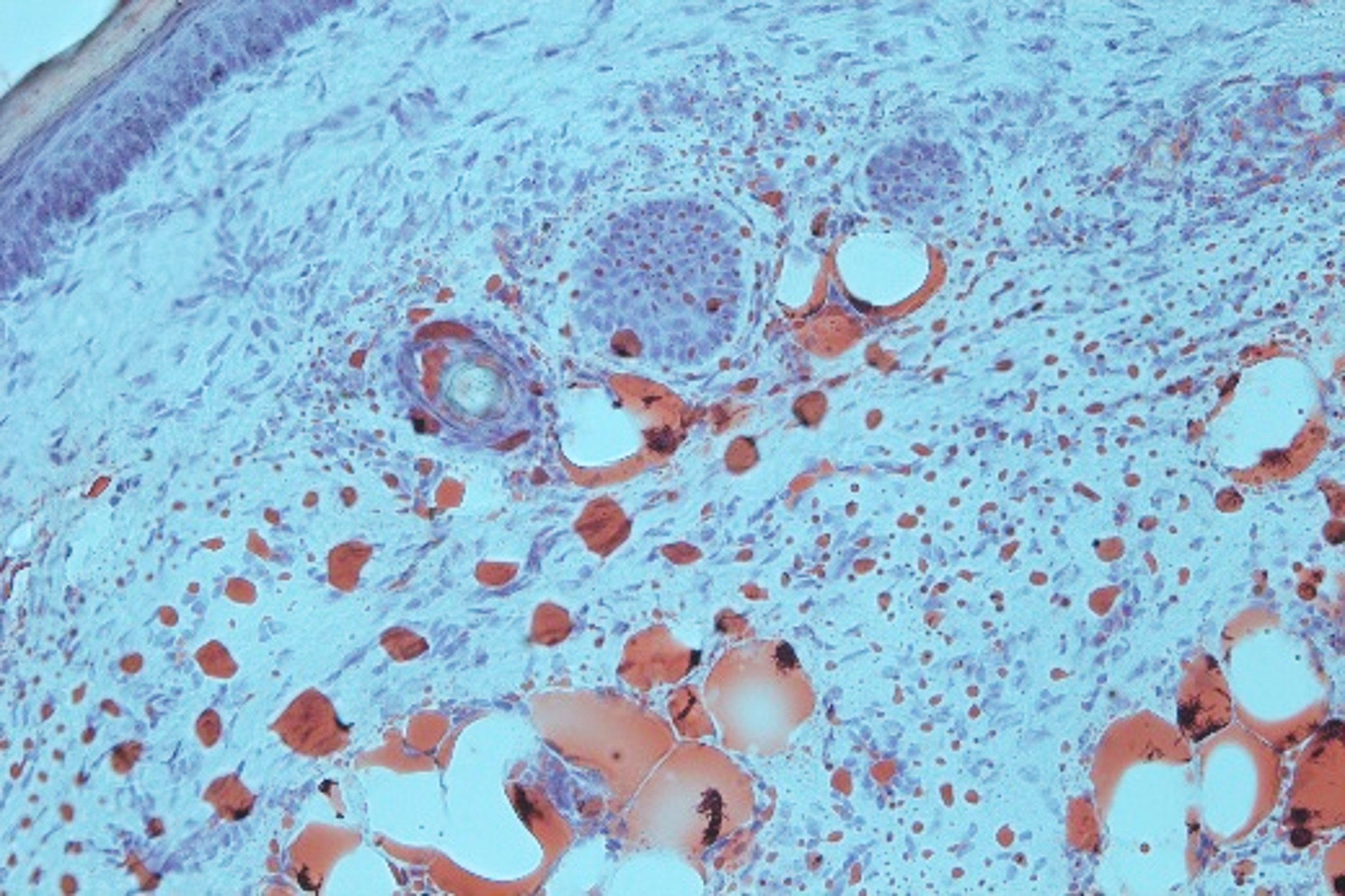

Supplement: Supplementary file 13 — Figure EV 4 Source Data [file 44321_2025_286_MOESM13_ESM.zip › Expanded View Figure 4/4A/HSFD+BMS Lymphedema.tif]

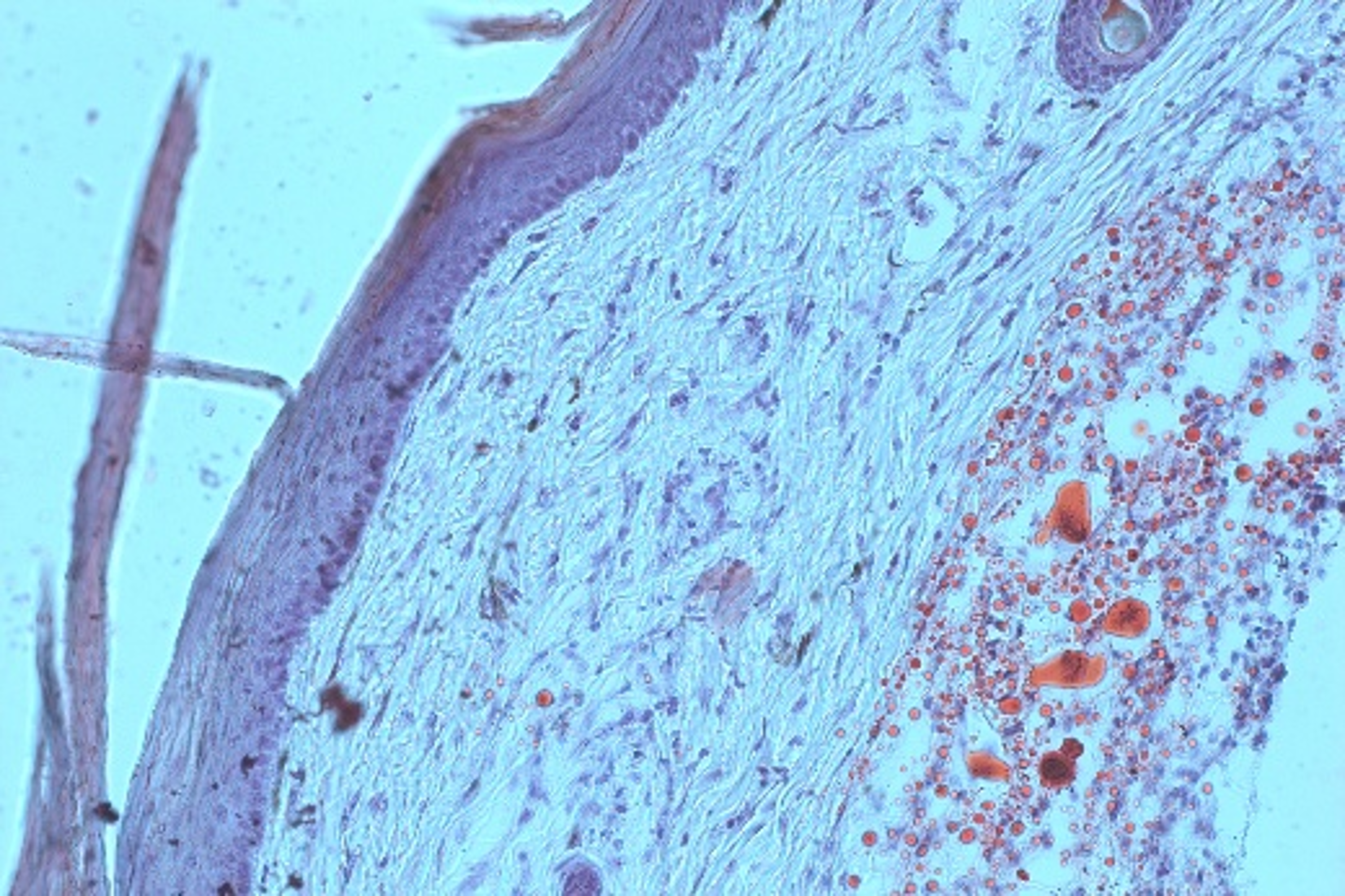

Supplement: Supplementary file 13 — Figure EV 4 Source Data [file 44321_2025_286_MOESM13_ESM.zip › Expanded View Figure 4/4A/HSFD-CD Lymphedema.tif]

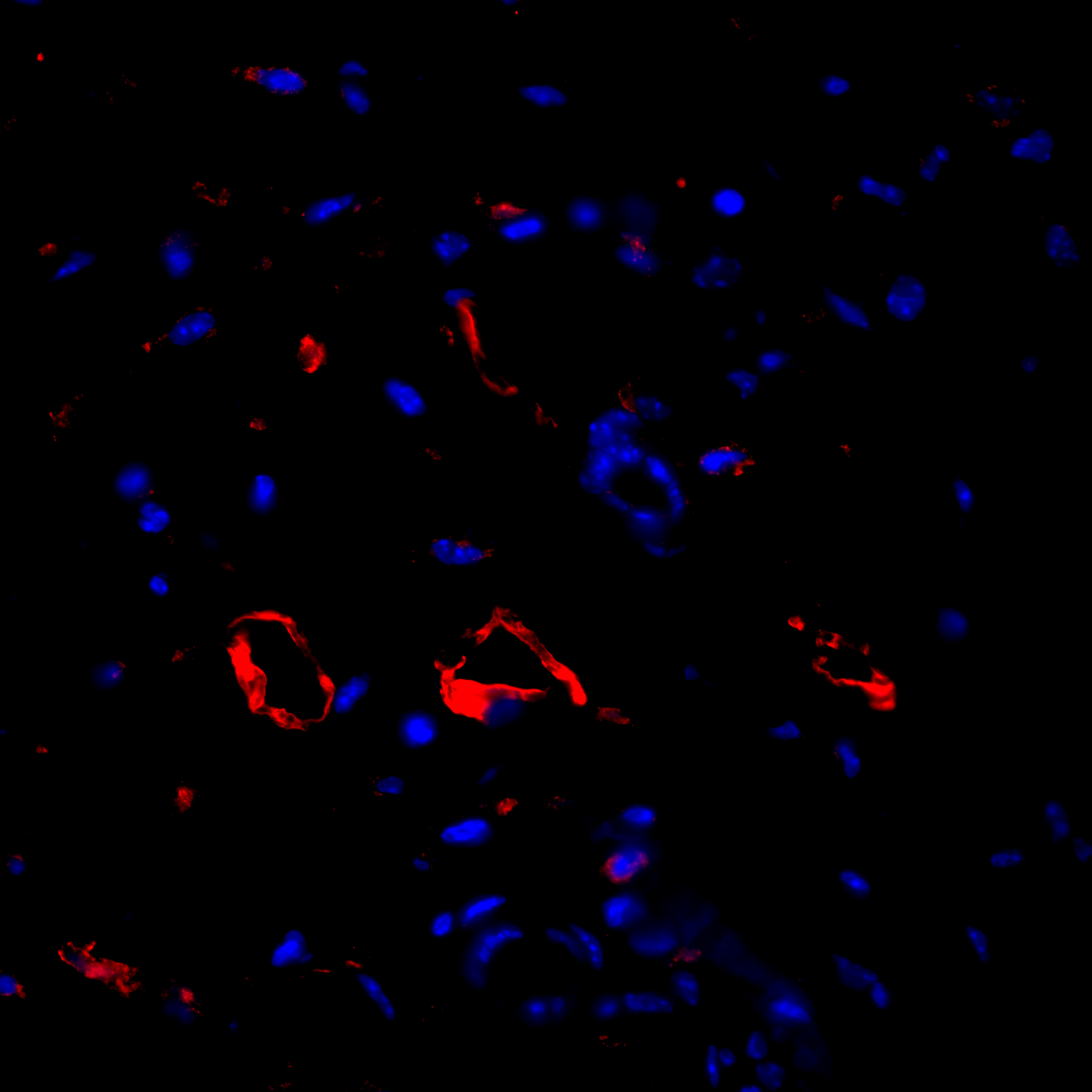

Supplement: Supplementary file 13 — Figure EV 4 Source Data [file 44321_2025_286_MOESM13_ESM.zip › Expanded View Figure 4/4C/CD_Composite.tif]

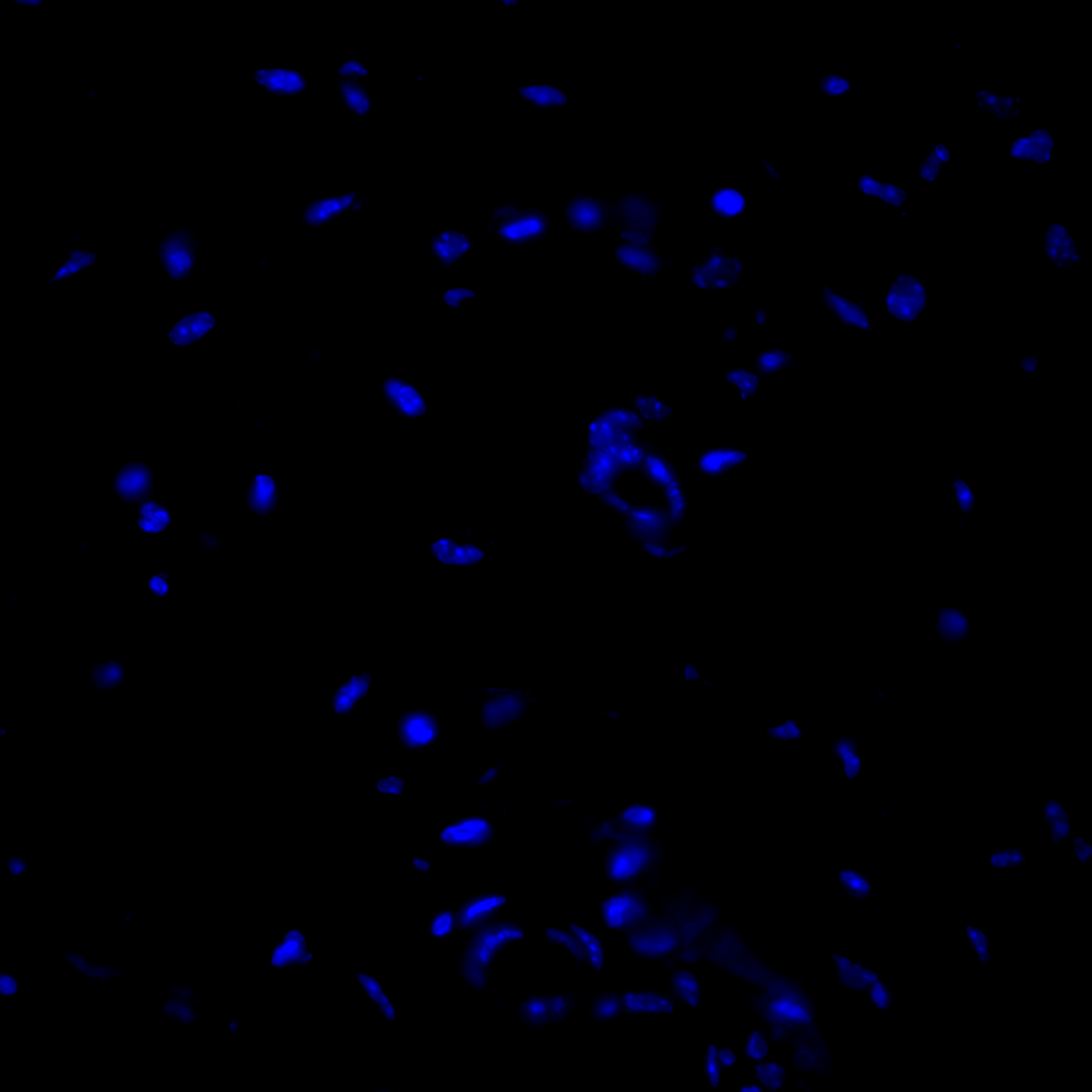

Supplement: Supplementary file 13 — Figure EV 4 Source Data [file 44321_2025_286_MOESM13_ESM.zip › Expanded View Figure 4/4C/CD_DAPI.tif]

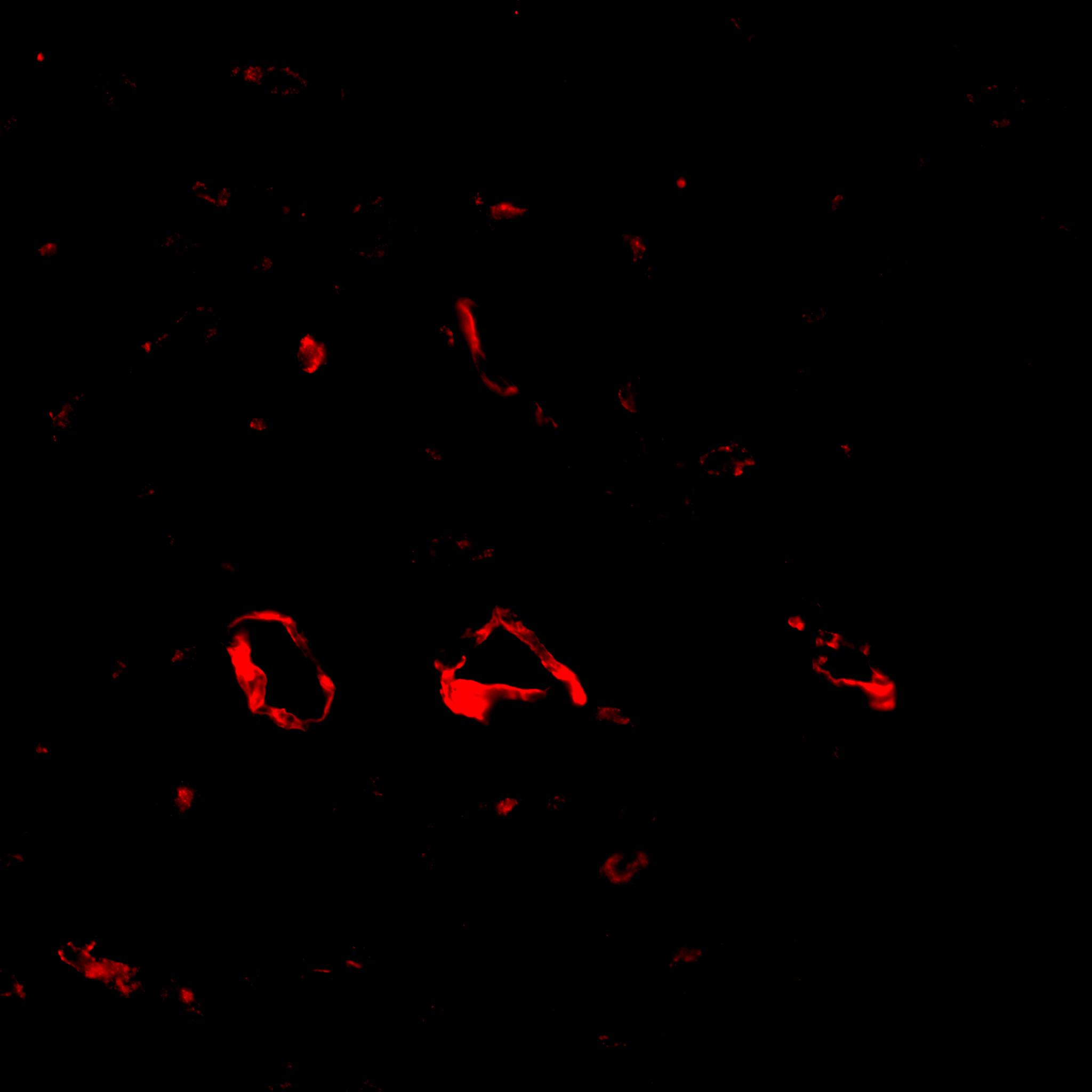

Supplement: Supplementary file 13 — Figure EV 4 Source Data [file 44321_2025_286_MOESM13_ESM.zip › Expanded View Figure 4/4C/CD_FABP4.tif]

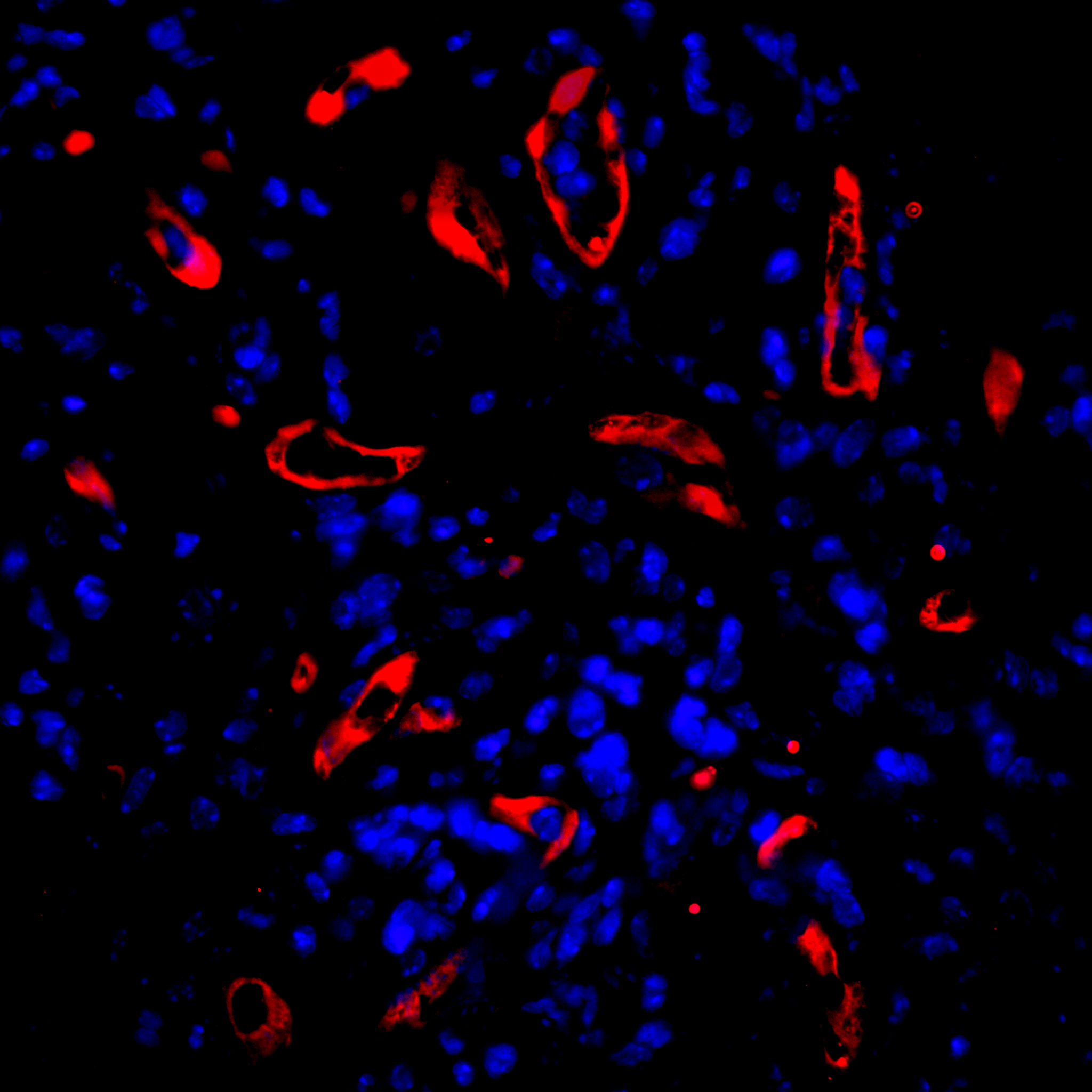

Supplement: Supplementary file 13 — Figure EV 4 Source Data [file 44321_2025_286_MOESM13_ESM.zip › Expanded View Figure 4/4C/HSFD+BMS_Composite.tif]

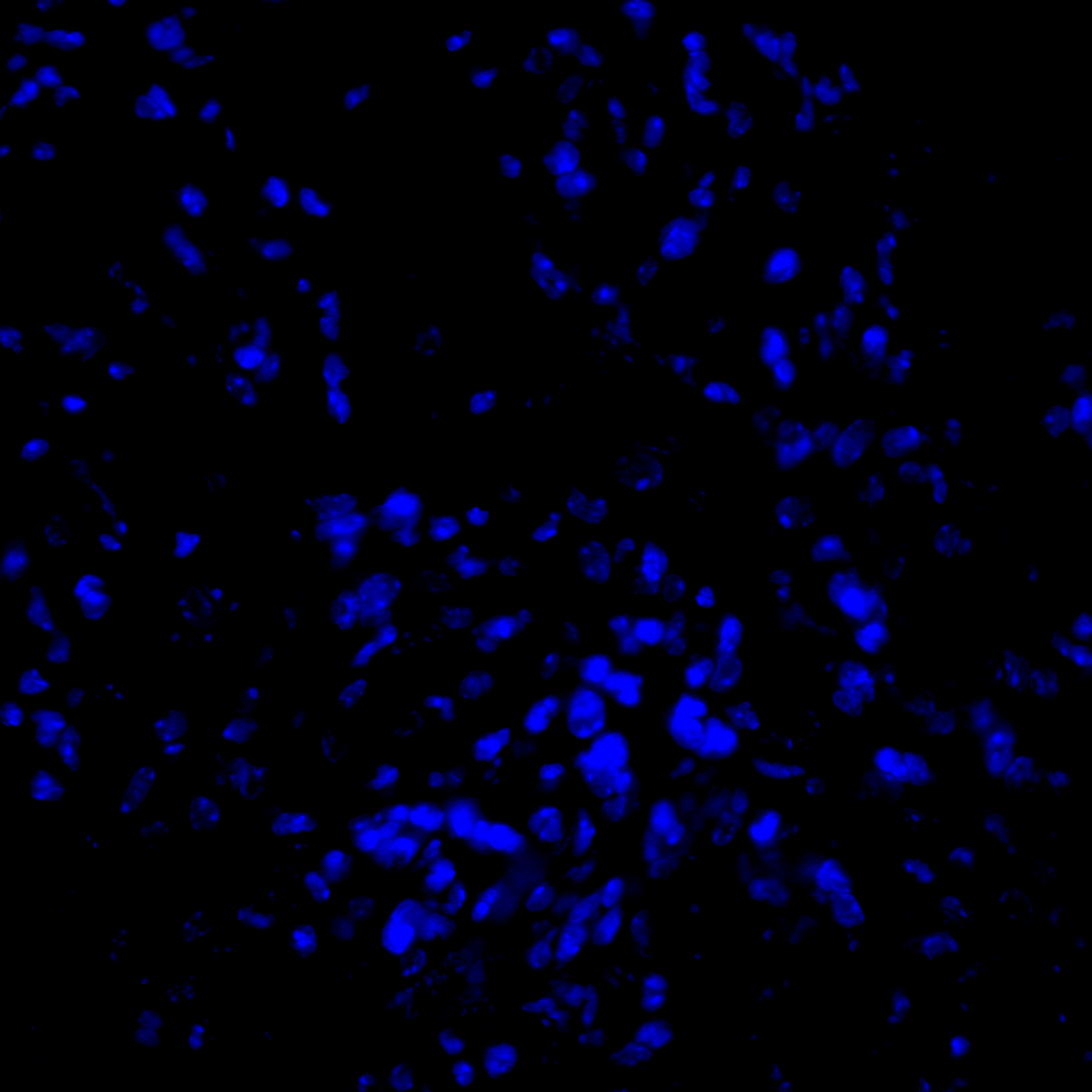

Supplement: Supplementary file 13 — Figure EV 4 Source Data [file 44321_2025_286_MOESM13_ESM.zip › Expanded View Figure 4/4C/HSFD+BMS_DAPI.tif]

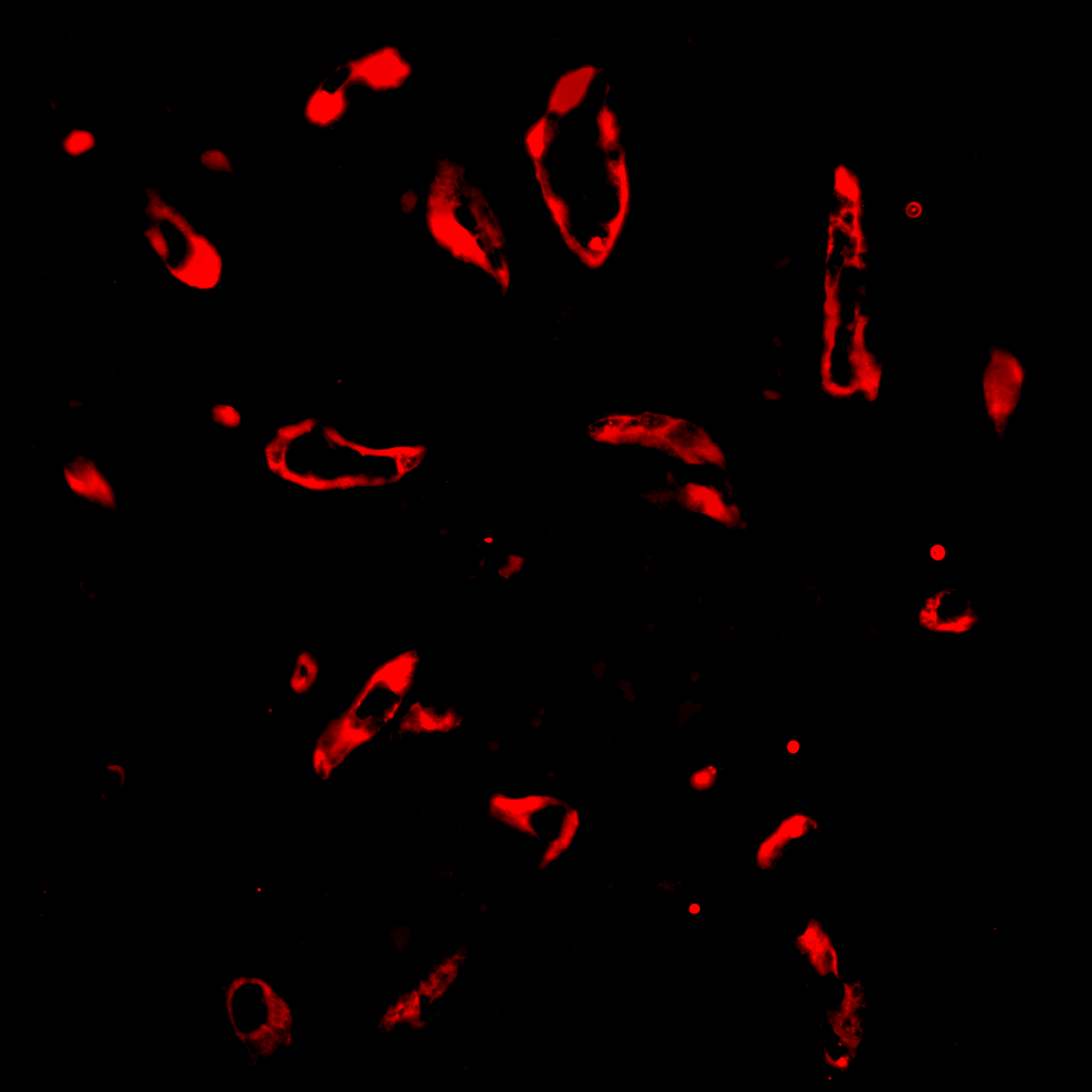

Supplement: Supplementary file 13 — Figure EV 4 Source Data [file 44321_2025_286_MOESM13_ESM.zip › Expanded View Figure 4/4C/HSFD+BMS_FABP4.tif]

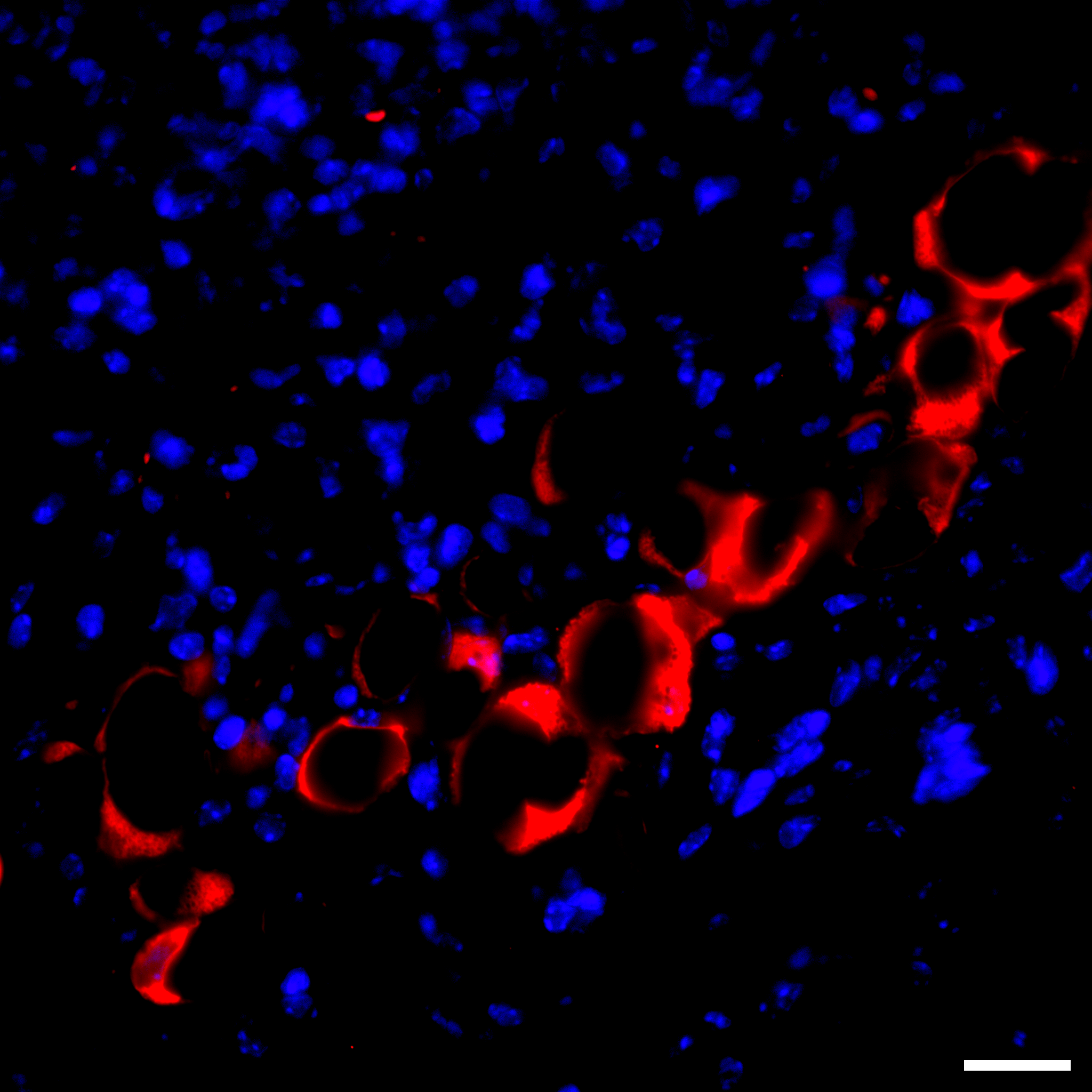

Supplement: Supplementary file 13 — Figure EV 4 Source Data [file 44321_2025_286_MOESM13_ESM.zip › Expanded View Figure 4/4C/HSFD-CD_Composite.tif]

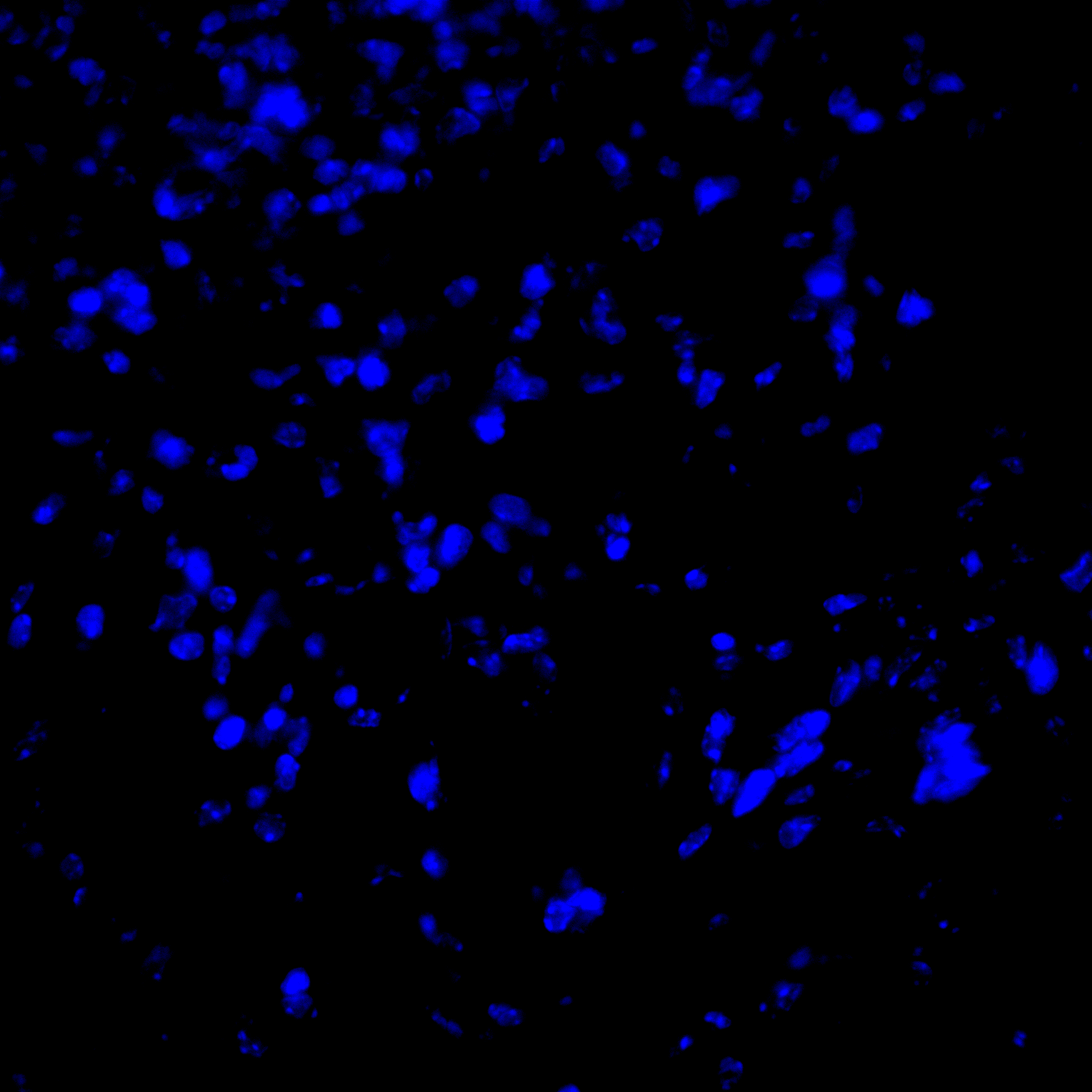

Supplement: Supplementary file 13 — Figure EV 4 Source Data [file 44321_2025_286_MOESM13_ESM.zip › Expanded View Figure 4/4C/HSFD-CD_DAPI.tif]

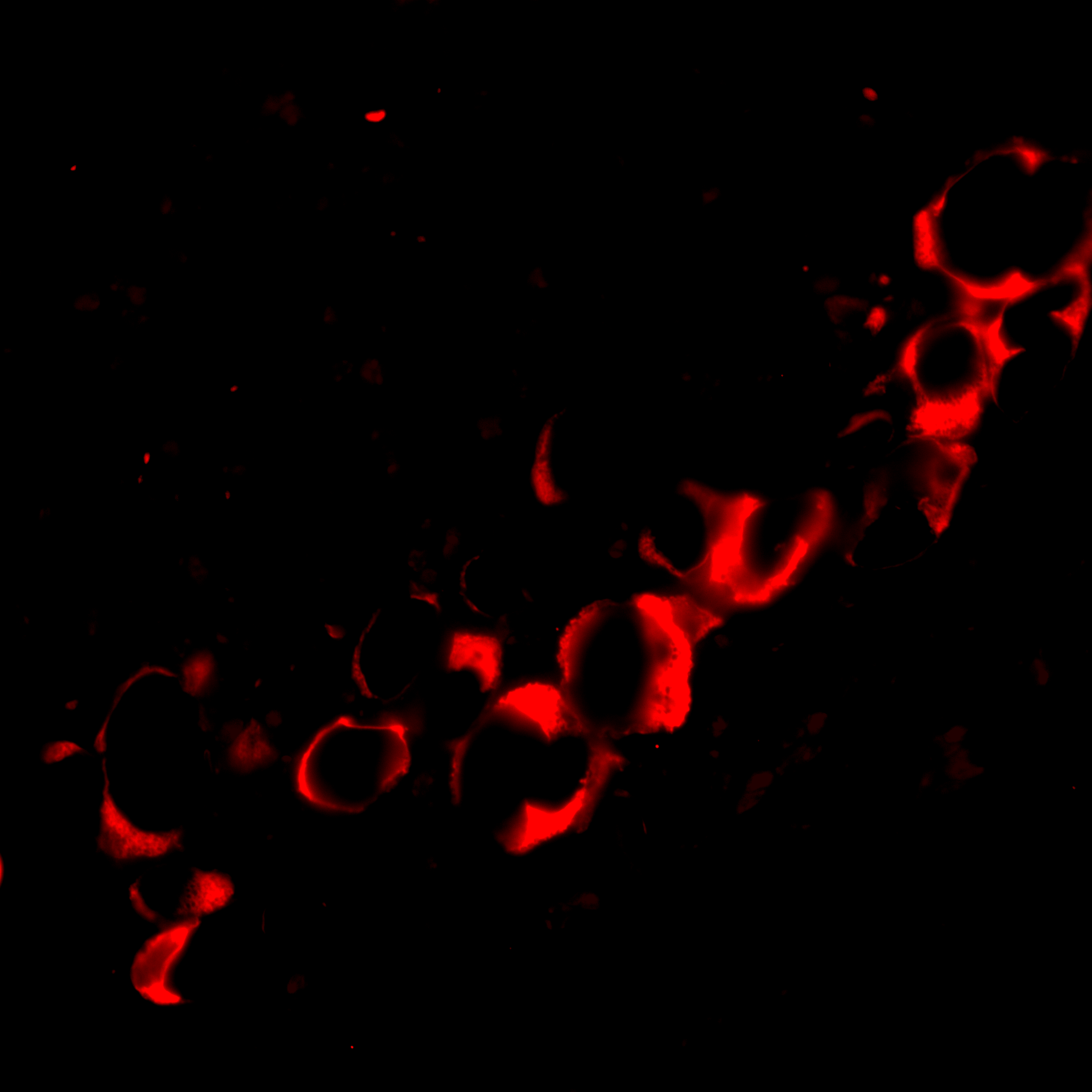

Supplement: Supplementary file 13 — Figure EV 4 Source Data [file 44321_2025_286_MOESM13_ESM.zip › Expanded View Figure 4/4C/HSFD-CD_FABP4.tif]

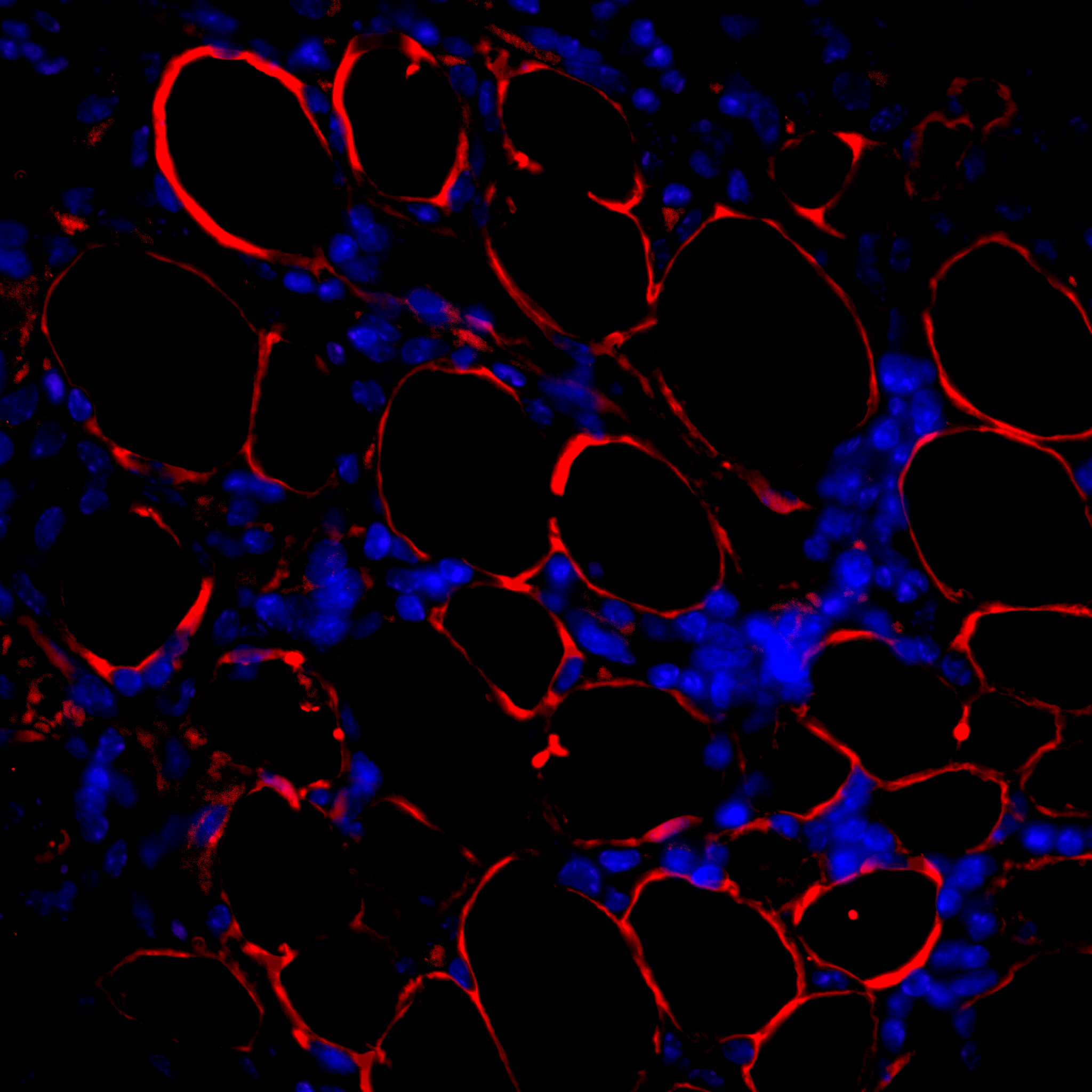

Supplement: Supplementary file 13 — Figure EV 4 Source Data [file 44321_2025_286_MOESM13_ESM.zip › Expanded View Figure 4/4C/HSFD_Composite.tif]

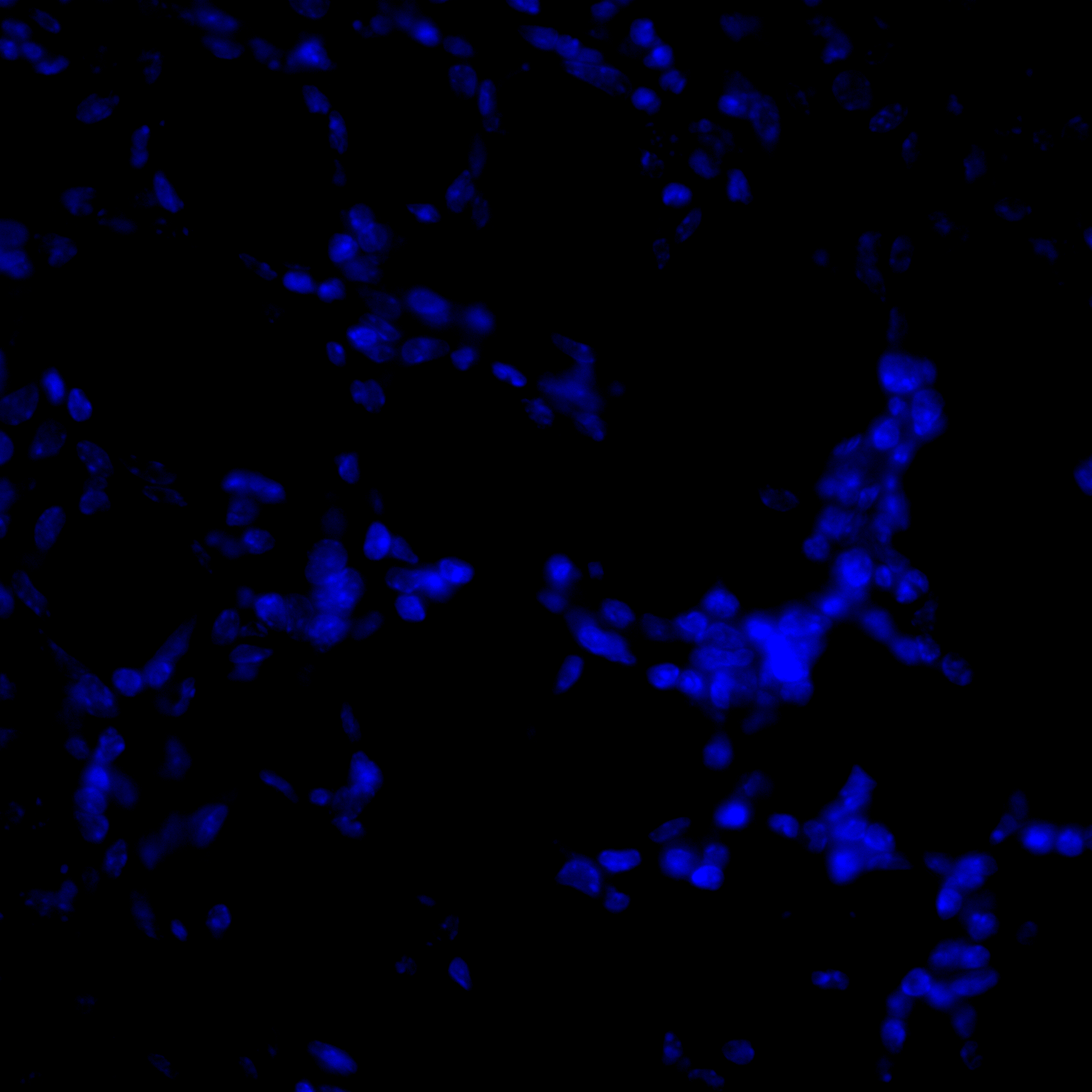

Supplement: Supplementary file 13 — Figure EV 4 Source Data [file 44321_2025_286_MOESM13_ESM.zip › Expanded View Figure 4/4C/HSFD_DAPI.tif]
