# Supplementary figures and images for: Saturated fatty acids induce lipotoxicity in lymphatic endothelial cells contributing to secondary lymphedema development (part 3 of 3)
Source: EMBO Mol Med. 2025 Aug 4;17(9):2384–408. doi: 10.1038/s44321-025-00286-4 (PMC12423331; doi:10.1038/s44321-025-00286-4)

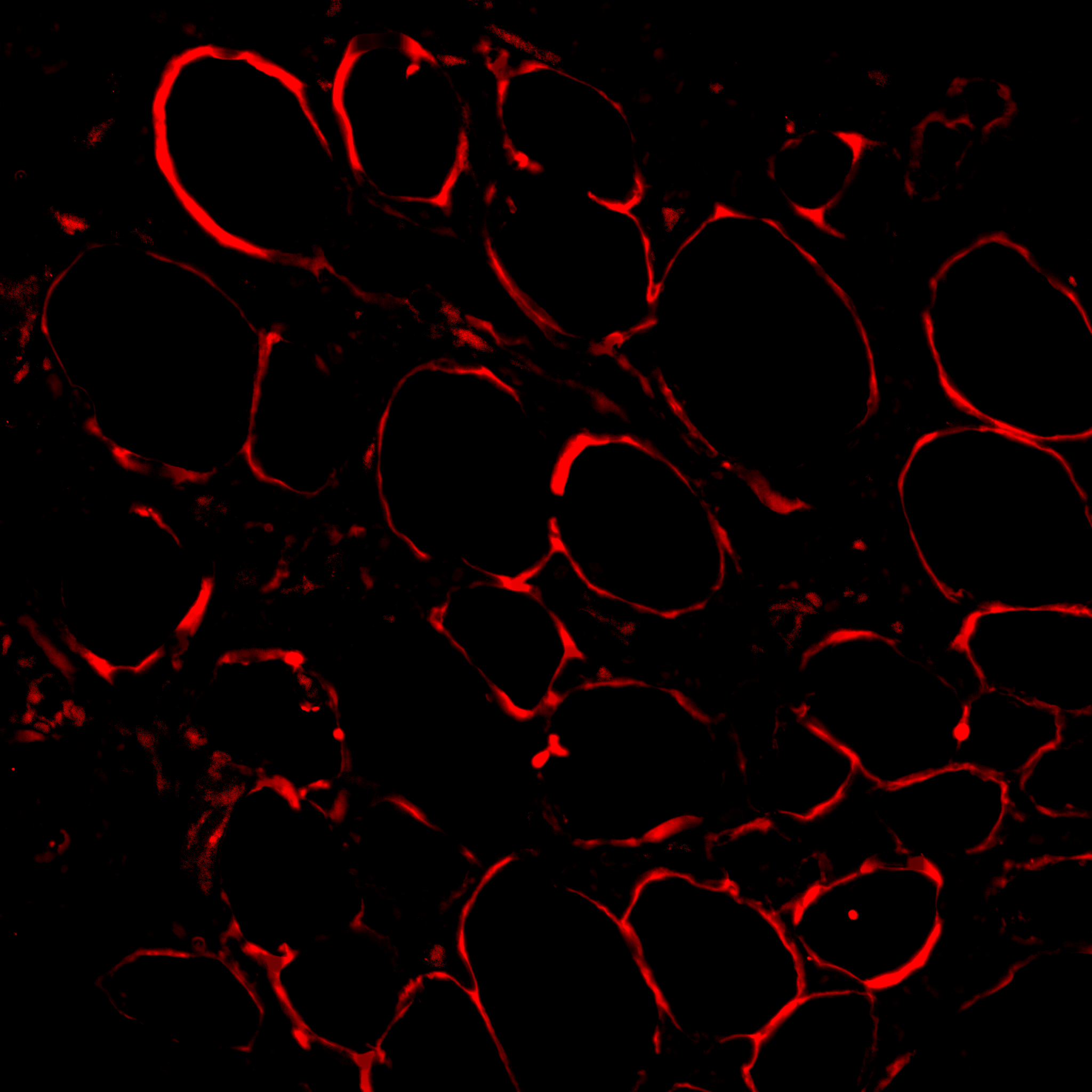

Supplement: Supplementary file 13 — Figure EV 4 Source Data [file 44321_2025_286_MOESM13_ESM.zip › Expanded View Figure 4/4C/HSFD_FABP4.tif]

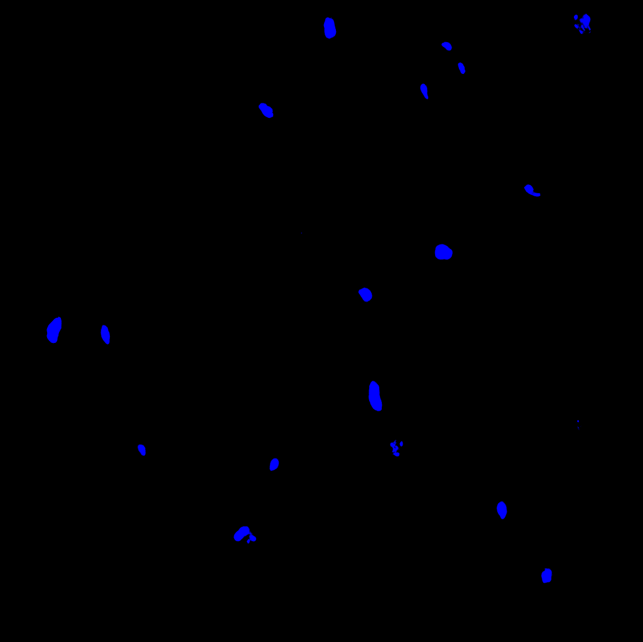

Supplement: Supplementary file 14 — Figure EV 5 Source Data [file 44321_2025_286_MOESM14_ESM.zip › Expanded View Figure 5/5A/CD_DAPI.tif]

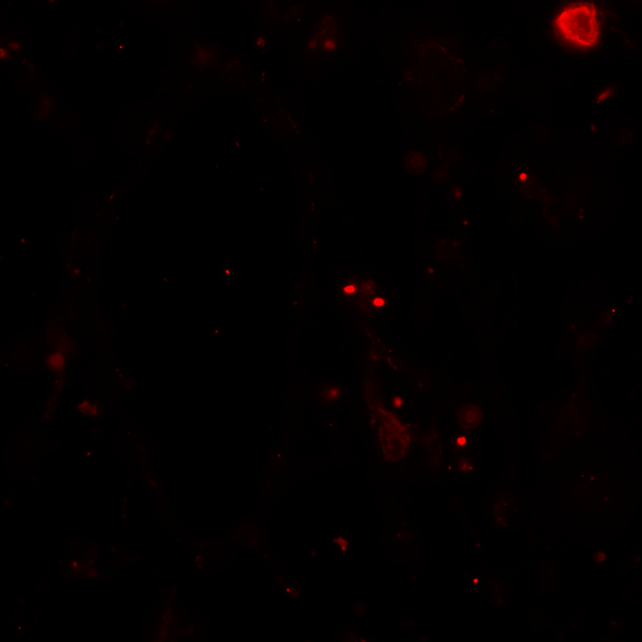

Supplement: Supplementary file 14 — Figure EV 5 Source Data [file 44321_2025_286_MOESM14_ESM.zip › Expanded View Figure 5/5A/CD_F480.tif]

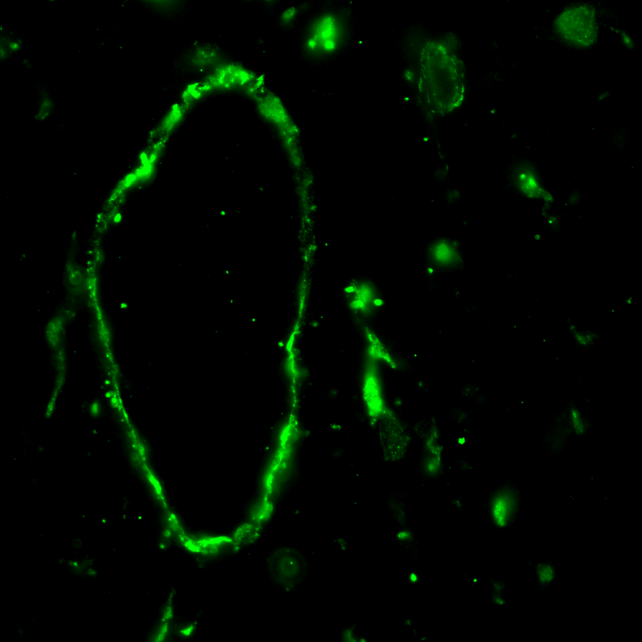

Supplement: Supplementary file 14 — Figure EV 5 Source Data [file 44321_2025_286_MOESM14_ESM.zip › Expanded View Figure 5/5A/CD_LYVE-1.tif]

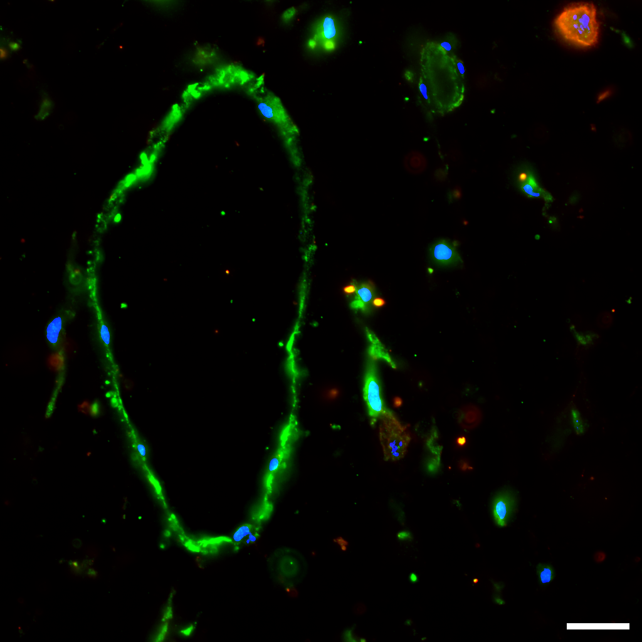

Supplement: Supplementary file 14 — Figure EV 5 Source Data [file 44321_2025_286_MOESM14_ESM.zip › Expanded View Figure 5/5A/CD_Merged.tif]

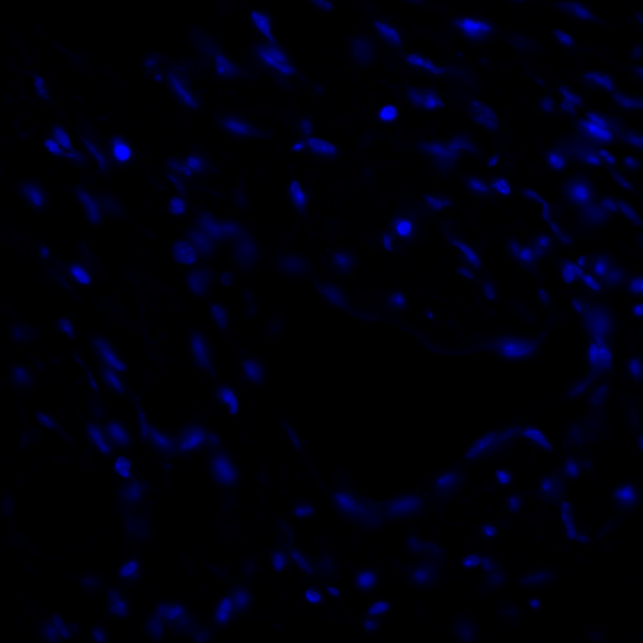

Supplement: Supplementary file 14 — Figure EV 5 Source Data [file 44321_2025_286_MOESM14_ESM.zip › Expanded View Figure 5/5A/HSFD+BMS_DAPI.tif]

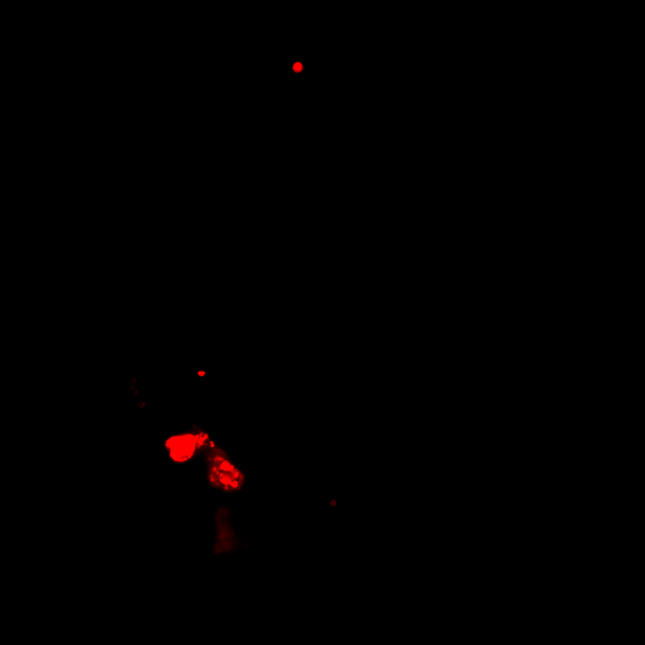

Supplement: Supplementary file 14 — Figure EV 5 Source Data [file 44321_2025_286_MOESM14_ESM.zip › Expanded View Figure 5/5A/HSFD+BMS_F480.tif]

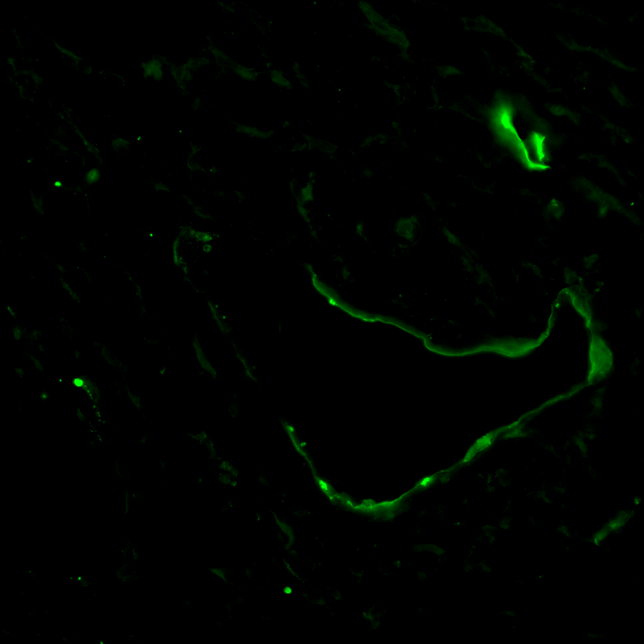

Supplement: Supplementary file 14 — Figure EV 5 Source Data [file 44321_2025_286_MOESM14_ESM.zip › Expanded View Figure 5/5A/HSFD+BMS_LYVE-1.tif]

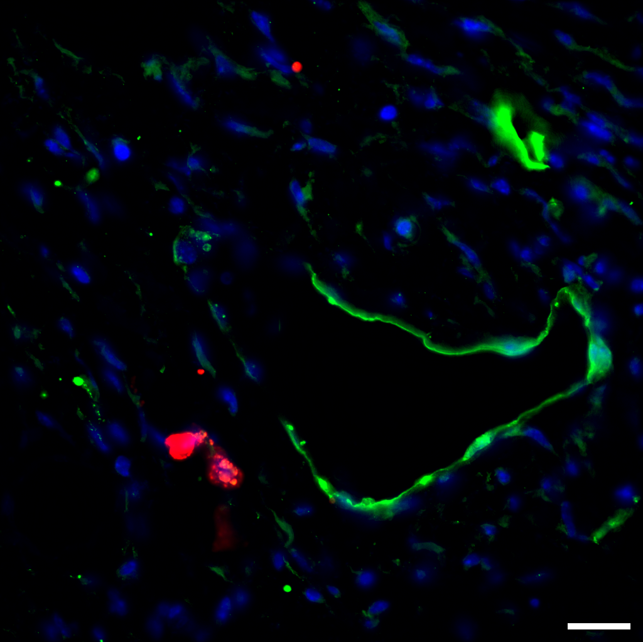

Supplement: Supplementary file 14 — Figure EV 5 Source Data [file 44321_2025_286_MOESM14_ESM.zip › Expanded View Figure 5/5A/HSFD+BMS_Merged.tif]

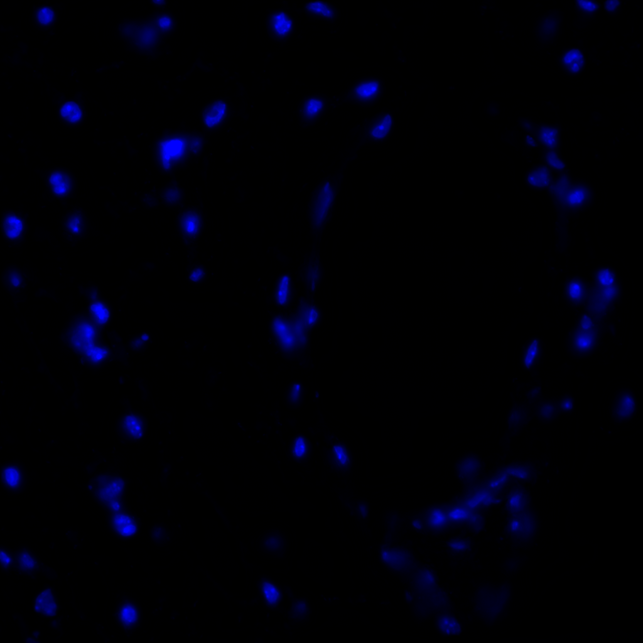

Supplement: Supplementary file 14 — Figure EV 5 Source Data [file 44321_2025_286_MOESM14_ESM.zip › Expanded View Figure 5/5A/HSFD-CD_DAPI.tif]

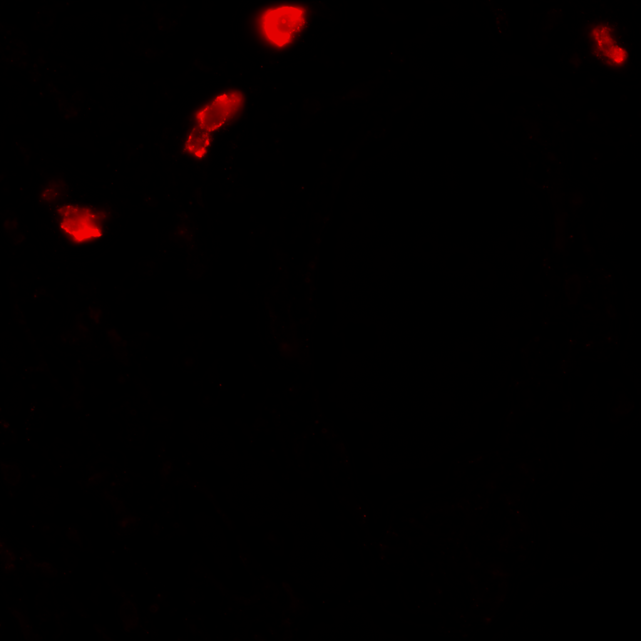

Supplement: Supplementary file 14 — Figure EV 5 Source Data [file 44321_2025_286_MOESM14_ESM.zip › Expanded View Figure 5/5A/HSFD-CD_F480.tif]

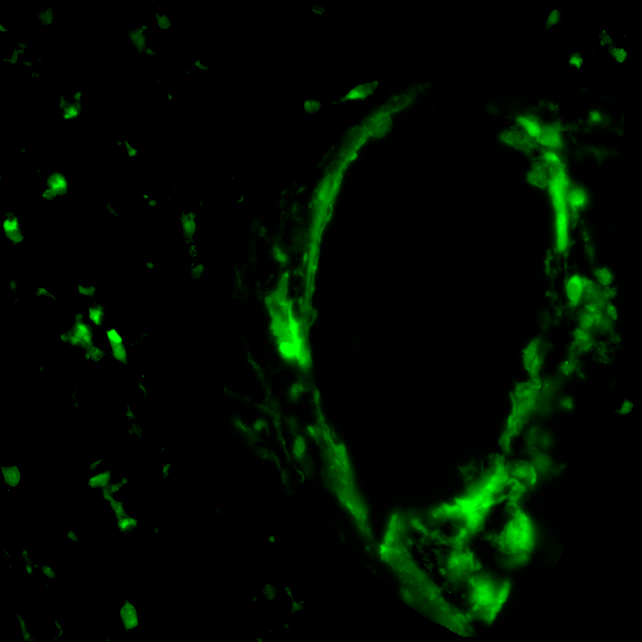

Supplement: Supplementary file 14 — Figure EV 5 Source Data [file 44321_2025_286_MOESM14_ESM.zip › Expanded View Figure 5/5A/HSFD-CD_LYVE-1.tif]

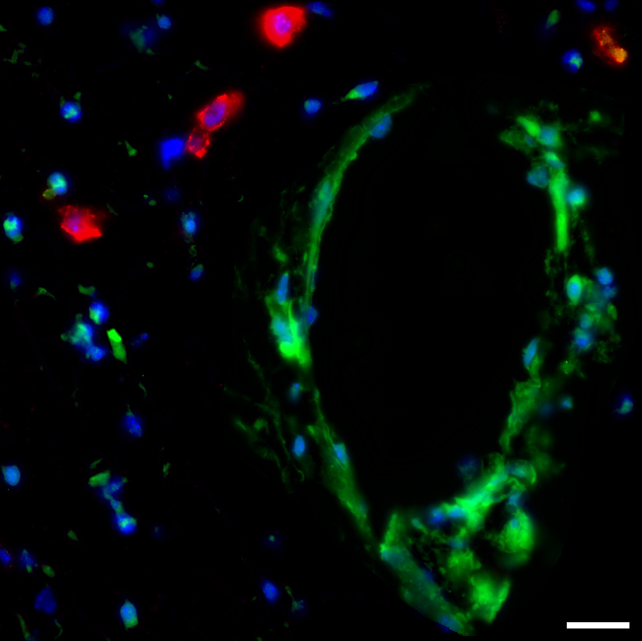

Supplement: Supplementary file 14 — Figure EV 5 Source Data [file 44321_2025_286_MOESM14_ESM.zip › Expanded View Figure 5/5A/HSFD-CD_Merged.tif]

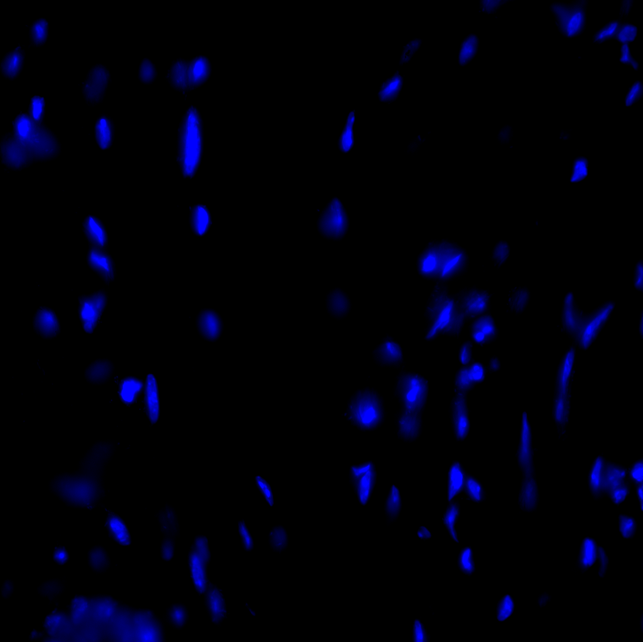

Supplement: Supplementary file 14 — Figure EV 5 Source Data [file 44321_2025_286_MOESM14_ESM.zip › Expanded View Figure 5/5A/HSFD_DAPI.tif]

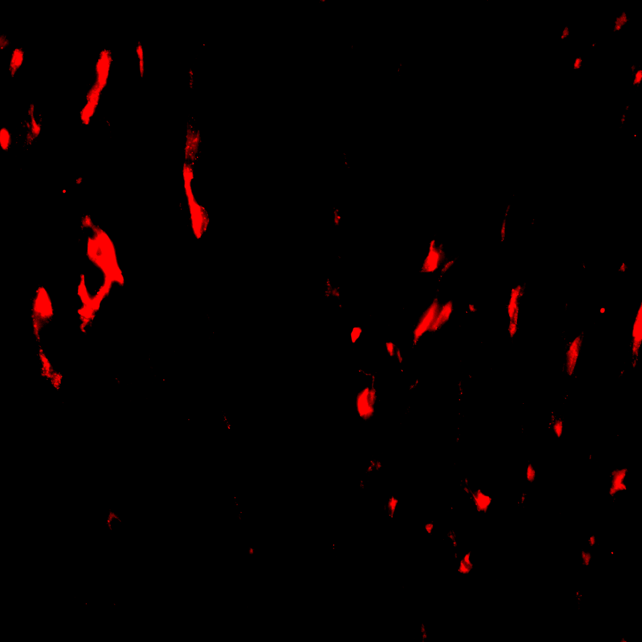

Supplement: Supplementary file 14 — Figure EV 5 Source Data [file 44321_2025_286_MOESM14_ESM.zip › Expanded View Figure 5/5A/HSFD_F480.tif]

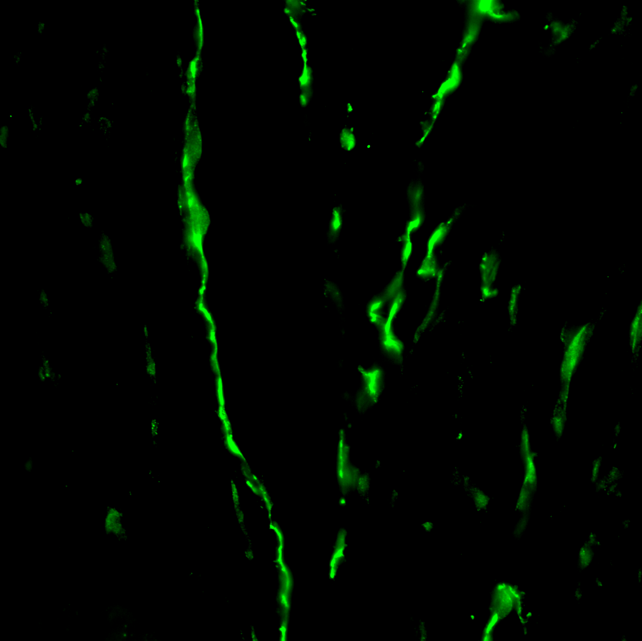

Supplement: Supplementary file 14 — Figure EV 5 Source Data [file 44321_2025_286_MOESM14_ESM.zip › Expanded View Figure 5/5A/HSFD_LYVE-1.tif]

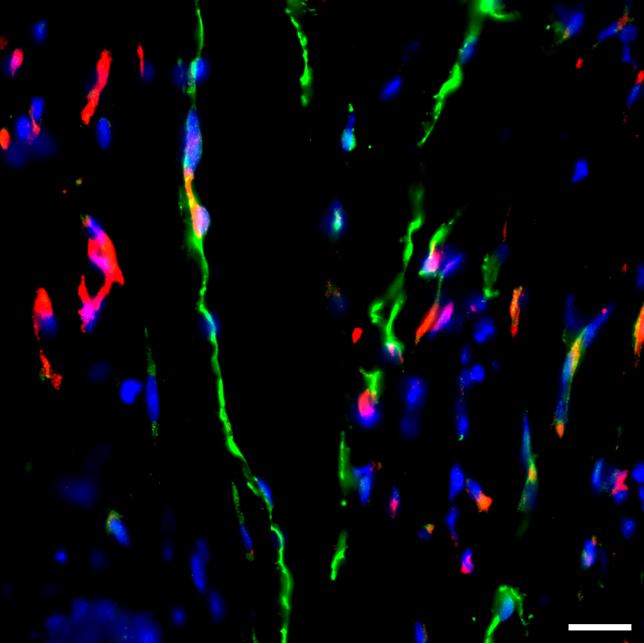

Supplement: Supplementary file 14 — Figure EV 5 Source Data [file 44321_2025_286_MOESM14_ESM.zip › Expanded View Figure 5/5A/HSFD_Merged.tif]

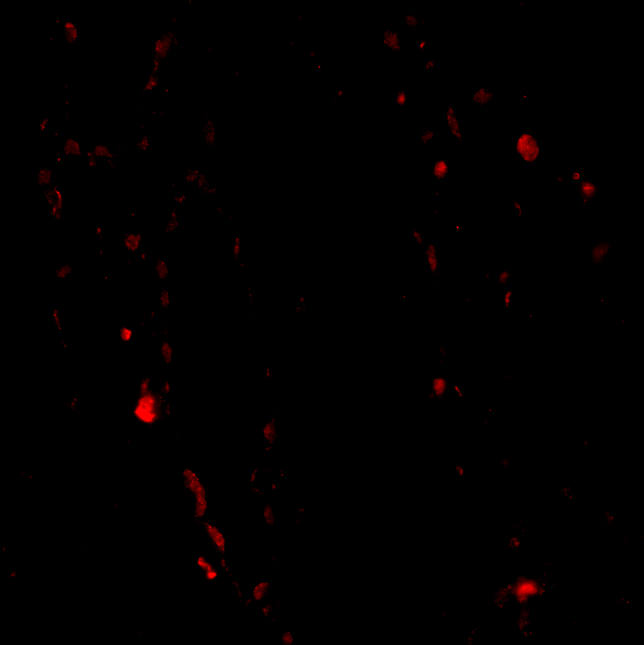

Supplement: Supplementary file 14 — Figure EV 5 Source Data [file 44321_2025_286_MOESM14_ESM.zip › Expanded View Figure 5/5B/CD_CD4.tif]

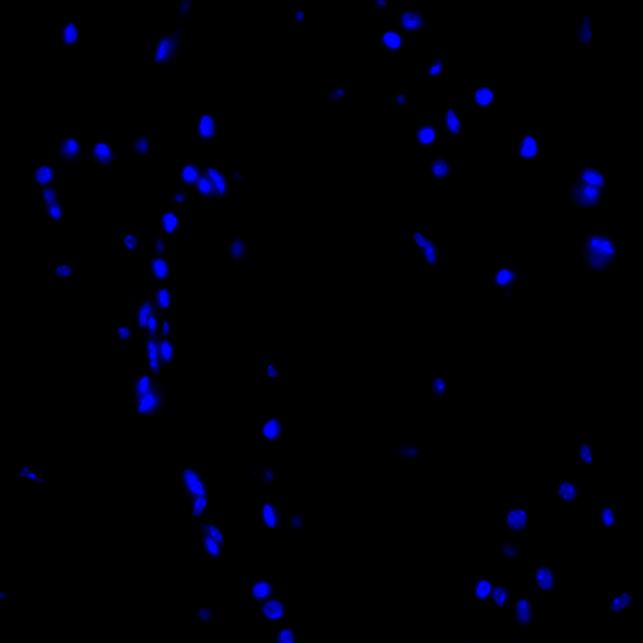

Supplement: Supplementary file 14 — Figure EV 5 Source Data [file 44321_2025_286_MOESM14_ESM.zip › Expanded View Figure 5/5B/CD_DAPI.tif]

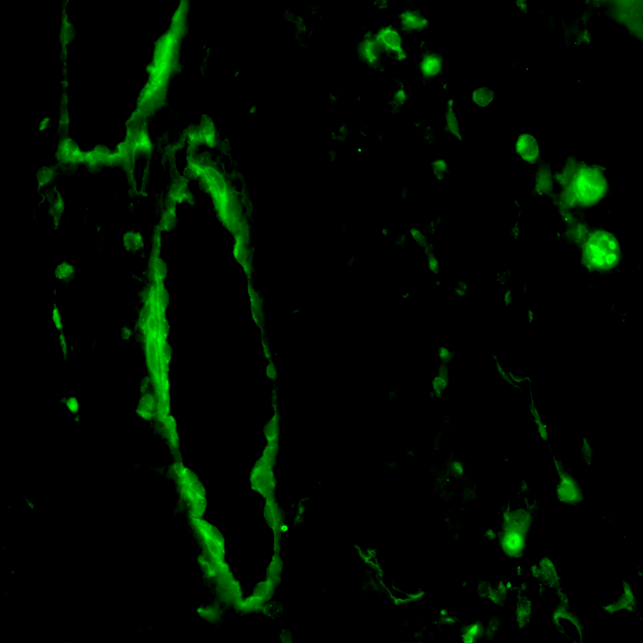

Supplement: Supplementary file 14 — Figure EV 5 Source Data [file 44321_2025_286_MOESM14_ESM.zip › Expanded View Figure 5/5B/CD_LYVE-1.tif]

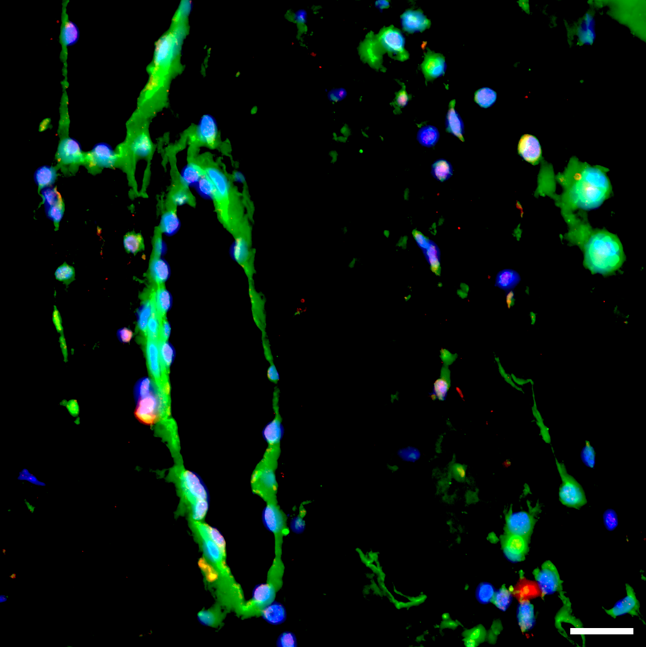

Supplement: Supplementary file 14 — Figure EV 5 Source Data [file 44321_2025_286_MOESM14_ESM.zip › Expanded View Figure 5/5B/CD_Merged.tif]

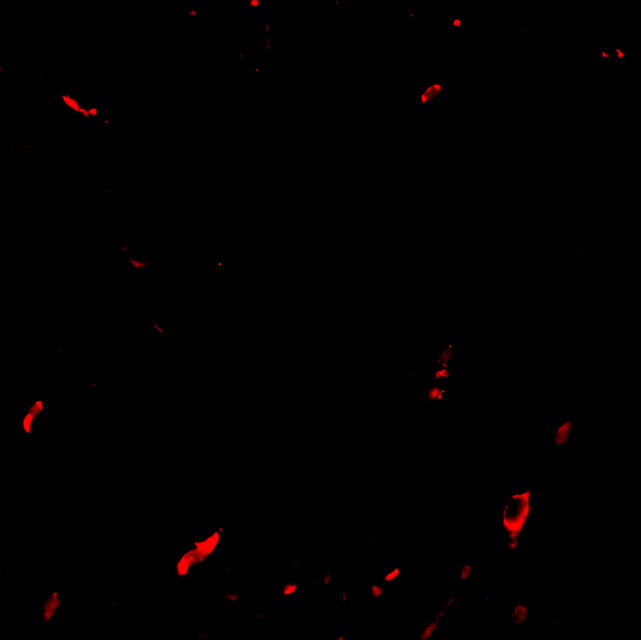

Supplement: Supplementary file 14 — Figure EV 5 Source Data [file 44321_2025_286_MOESM14_ESM.zip › Expanded View Figure 5/5B/HSFD+BMS_CD4.tif]

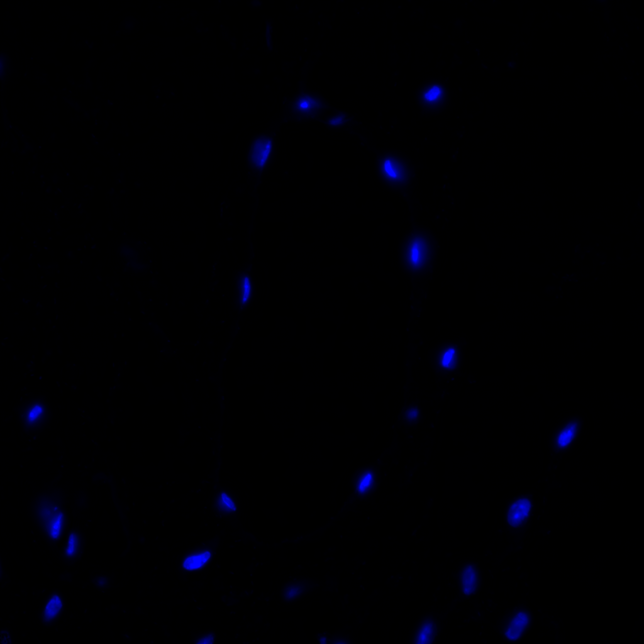

Supplement: Supplementary file 14 — Figure EV 5 Source Data [file 44321_2025_286_MOESM14_ESM.zip › Expanded View Figure 5/5B/HSFD+BMS_DAPI.tif]

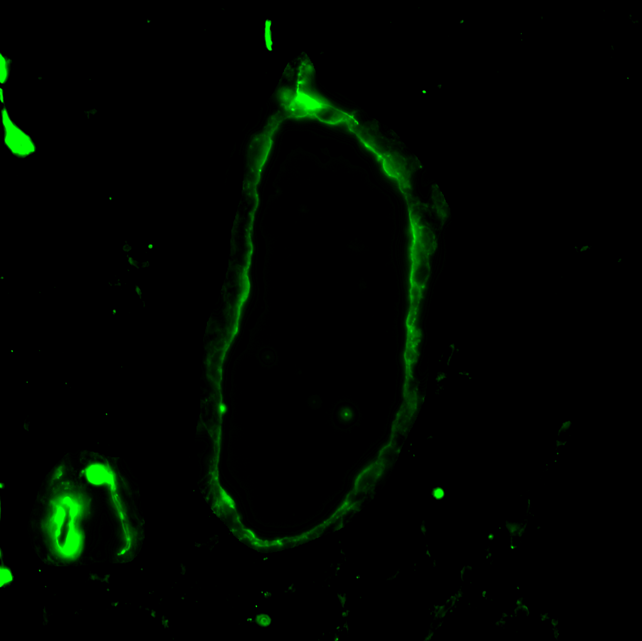

Supplement: Supplementary file 14 — Figure EV 5 Source Data [file 44321_2025_286_MOESM14_ESM.zip › Expanded View Figure 5/5B/HSFD+BMS_LYVE-1.tif]

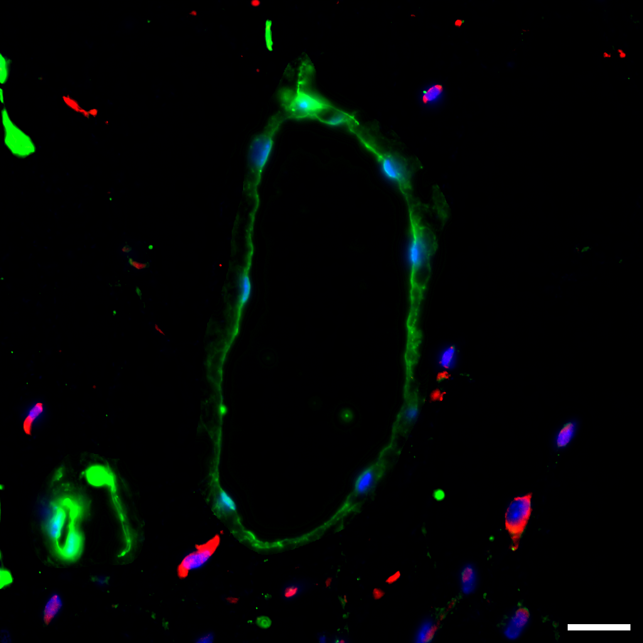

Supplement: Supplementary file 14 — Figure EV 5 Source Data [file 44321_2025_286_MOESM14_ESM.zip › Expanded View Figure 5/5B/HSFD+BMS_Merged.tif]

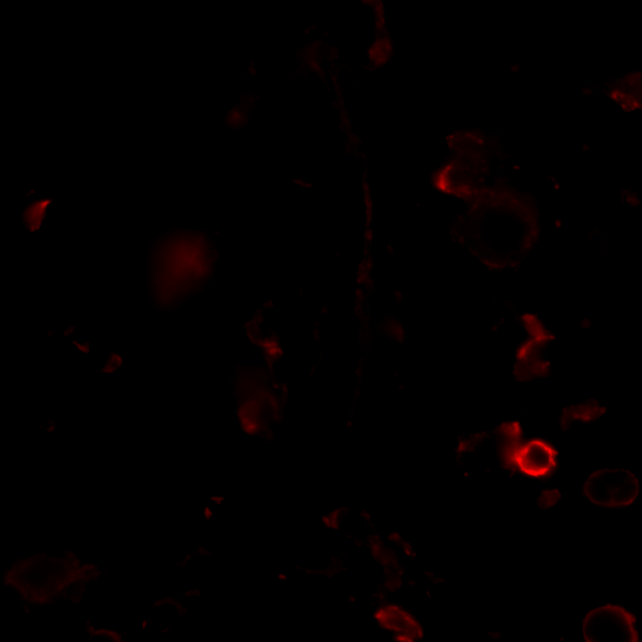

Supplement: Supplementary file 14 — Figure EV 5 Source Data [file 44321_2025_286_MOESM14_ESM.zip › Expanded View Figure 5/5B/HSFD-CD_CD4.tif]

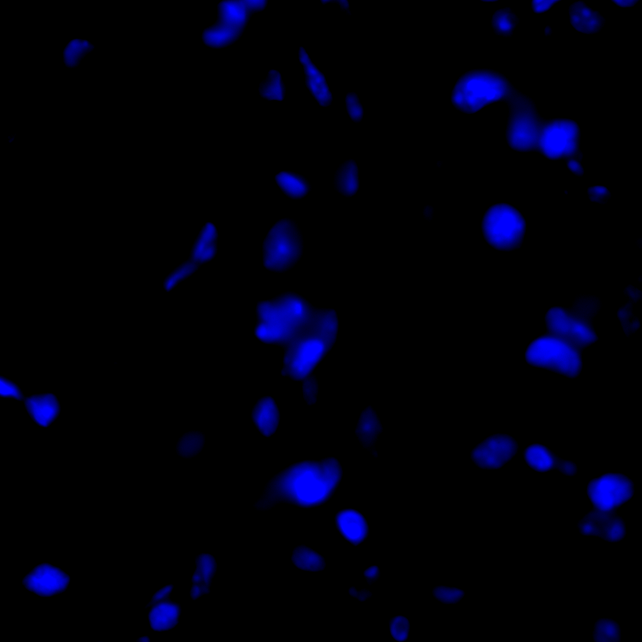

Supplement: Supplementary file 14 — Figure EV 5 Source Data [file 44321_2025_286_MOESM14_ESM.zip › Expanded View Figure 5/5B/HSFD-CD_DAPI.tif]

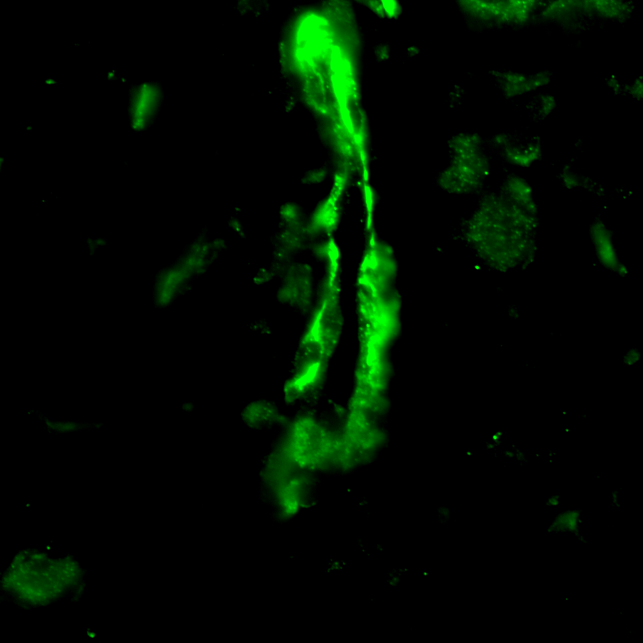

Supplement: Supplementary file 14 — Figure EV 5 Source Data [file 44321_2025_286_MOESM14_ESM.zip › Expanded View Figure 5/5B/HSFD-CD_LYVE-1.tif]

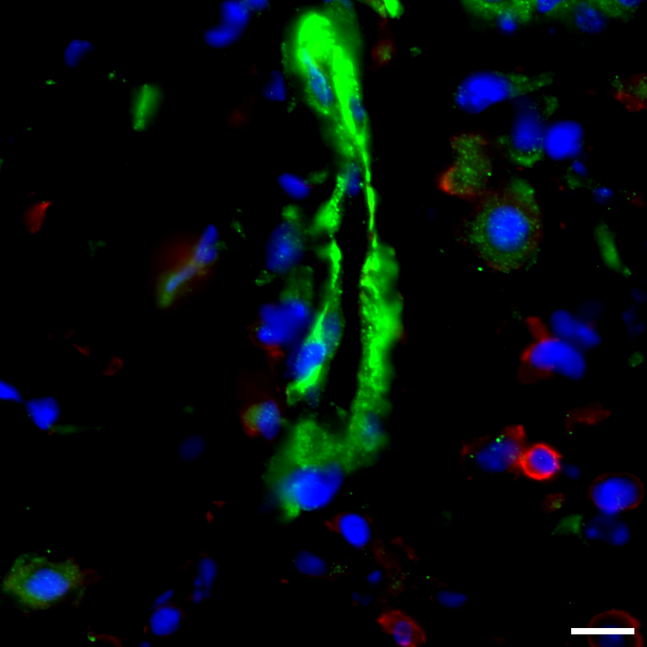

Supplement: Supplementary file 14 — Figure EV 5 Source Data [file 44321_2025_286_MOESM14_ESM.zip › Expanded View Figure 5/5B/HSFD-CD_Merged.tif]

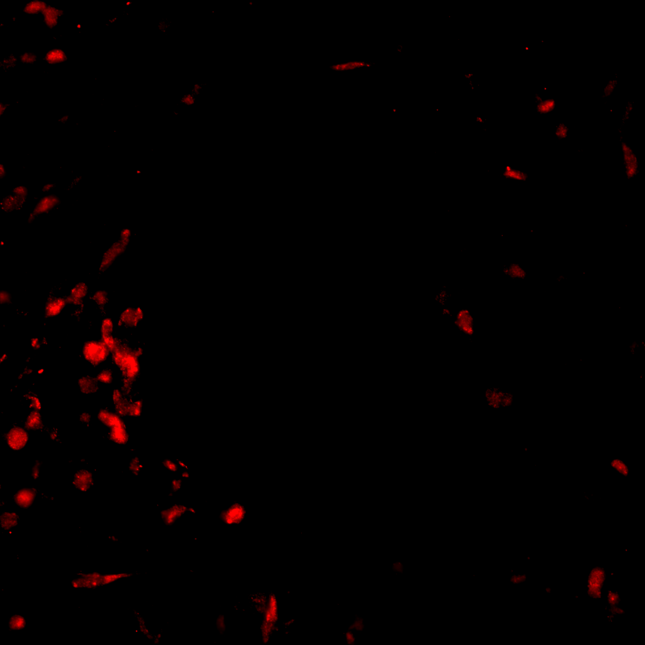

Supplement: Supplementary file 14 — Figure EV 5 Source Data [file 44321_2025_286_MOESM14_ESM.zip › Expanded View Figure 5/5B/HSFD_CD4.tif]

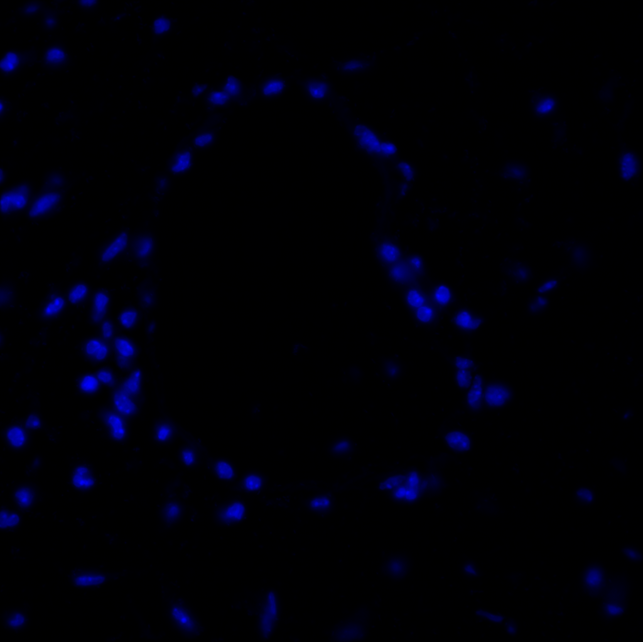

Supplement: Supplementary file 14 — Figure EV 5 Source Data [file 44321_2025_286_MOESM14_ESM.zip › Expanded View Figure 5/5B/HSFD_DAPI.tif]

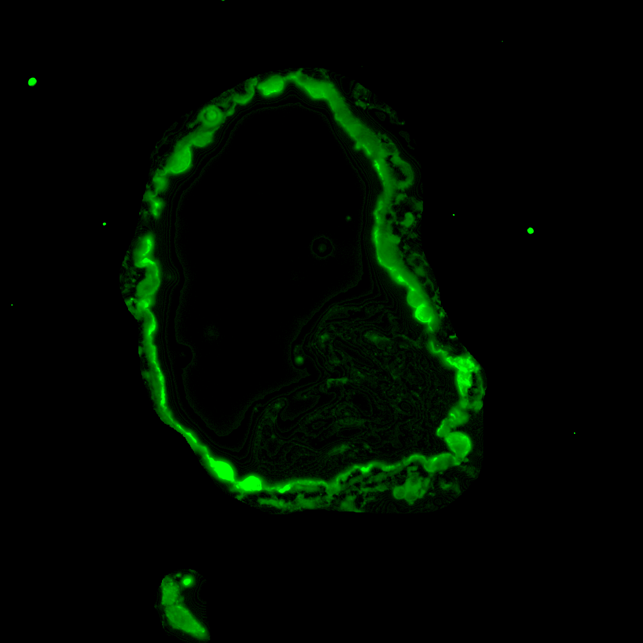

Supplement: Supplementary file 14 — Figure EV 5 Source Data [file 44321_2025_286_MOESM14_ESM.zip › Expanded View Figure 5/5B/HSFD_LYVE-1.tif]

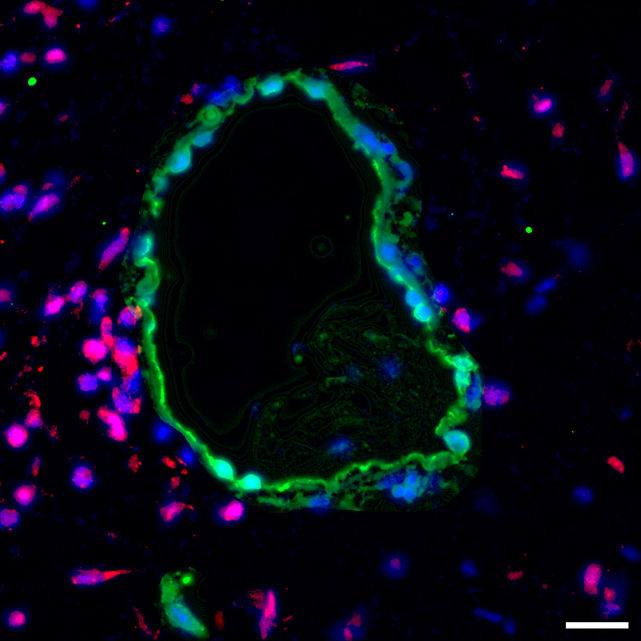

Supplement: Supplementary file 14 — Figure EV 5 Source Data [file 44321_2025_286_MOESM14_ESM.zip › Expanded View Figure 5/5B/HSFD_Merged.tif]
