# Supplementary material for: Saturated fatty acids induce lipotoxicity in lymphatic endothelial cells contributing to secondary lymphedema development
Source: EMBO Mol Med. 2025 Aug 4;17(9):2384–408. doi: 10.1038/s44321-025-00286-4 (PMC12423331; doi:10.1038/s44321-025-00286-4)
Supplement: Supplementary file 15 — Expanded View Figures [file 44321_2025_286_MOESM15_ESM.pdf]

Expanded View Figures

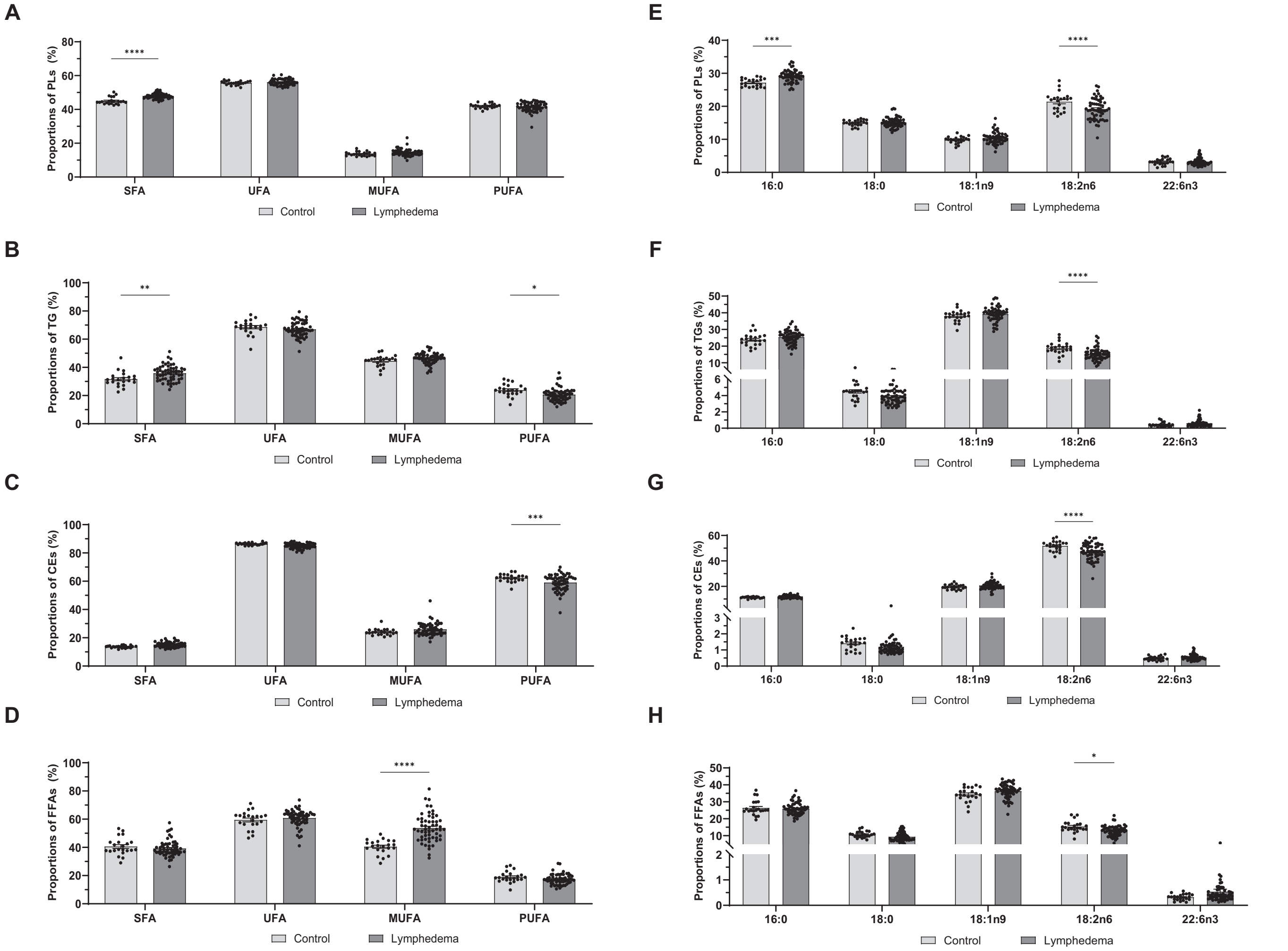

Figure EV1. Fatty acid composition in plasma lipid fractions of patients with lymphedema.

Plasma samples from patients with lymphedema ($n = 57$) and non-lymphedema controls ($n = 22$) were analyzed to assess the distribution of saturated (SFA), unsaturated (UFA), monounsaturated (MUFA), and polyunsaturated fatty acids (PUFA) across major lipid classes: (**A**) phospholipids (PLs), (**B**) triglycerides (TGs), (**C**) cholesterol esters (CEs), and (**D**) free fatty acids (FFAs). (**E–H**) Relative abundance of key fatty acid species—palmitic acid (16:0), stearic acid (18:0), oleic acid (18:1n9), linoleic acid (18:2n6), and docosahexaenoic acid (22:6n3)—within each lipid fraction: (**E**) PLs, (**F**) TGs, (**G**) CEs, and (**H**) FFAs. Data are presented as mean ± SEM. Statistical analysis: two-way ANOVA with Šídák's post hoc test. Significance: *$P < 0.05$, **$P < 0.01$, ***$P < 0.001$, ****$P < 0.0001$. Exact *P* values for the statistical comparisons are shown in Appendix Table S3. Source data are available online for this figure.

© The Author(s)

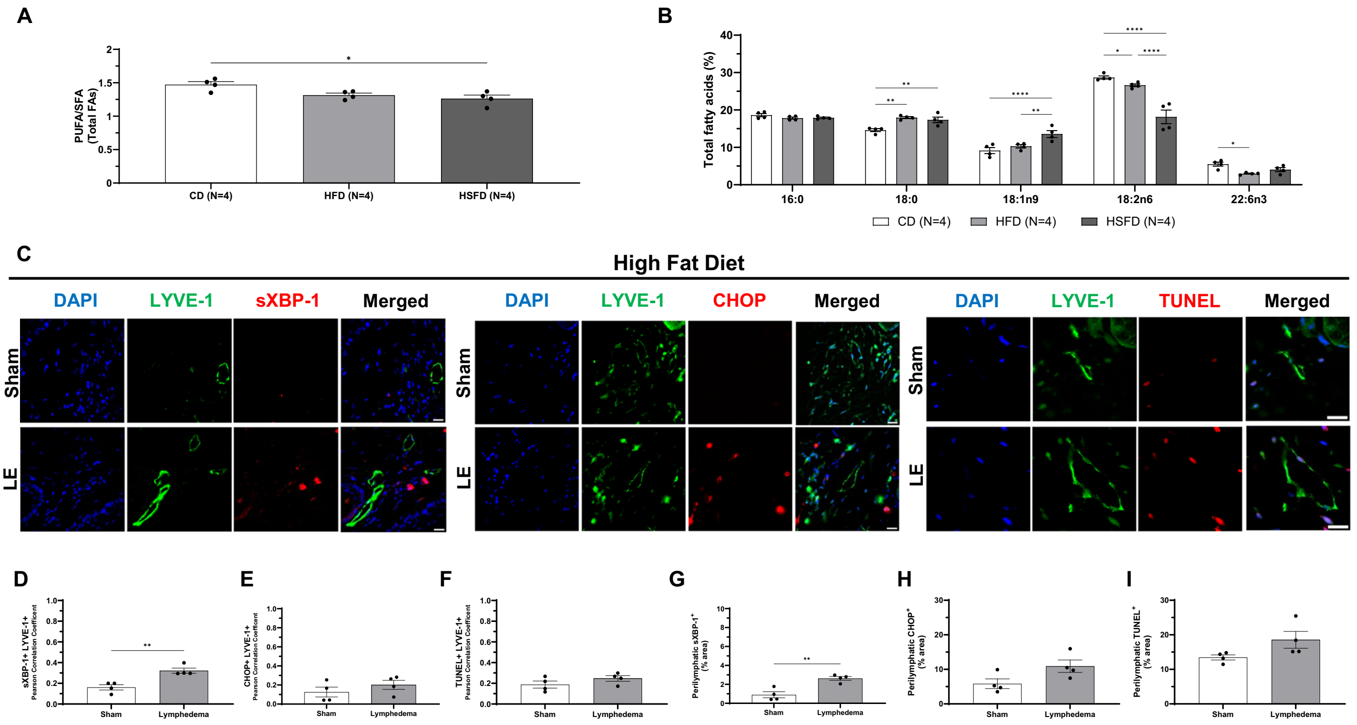

Figure EV2. PUFA/SFA ratio and fatty acid composition in plasma total fatty acids of mice fed different diets.

(**A**) PUFA/SFA ratio in plasma total fatty acids from mice fed a chow diet (CD), high-fat diet (HFD), or high saturated fat diet (HSFD) ($n = 4$ mice). (**B**) Proportions of individual fatty acids: palmitic acid (16:0), stearic acid (18:0), oleic acid (18:1n9), linoleic acid (18:2n6), and docosahexaenoic acid (22:6n3) ($n = 4$ mice). (**C**) Representative images of tail tissue from sham and lymphedema (LE) mice fed a high-fat diet, stained for LYVE-1 (green), sXBP-1, CHOP, or TUNEL (red), and DAPI (blue). (**D–F**) Quantification of sXBP-1, CHOP, and TUNEL signal colocalized with LYVE-1 positive cells ($n = 4$ mice). (**G–I**) Quantification of perilymphatic signal for sXBP-1, CHOP, and TUNEL, showing elevated ER stress and apoptosis in lymphedema tissue ($n = 4$ mice). Data are presented as mean ± SEM. Statistical analysis: one-way ANOVA with Tukey's post hoc test for (**A**); two-way ANOVA with Šídák's post hoc test for (**B**); two-tailed unpaired *t* test for (**D–I**). Significance: $^*P < 0.05$, $^{**}P < 0.01$, $^{***}P < 0.001$, $^{****}P < 0.0001$. Exact *P* values for the statistical comparisons are shown in Appendix Table S3. PUFA polyunsaturated fatty acid, SFA saturated fatty acid, LYVE-1 lymphatic vessel endothelial hyaluronan receptor-1, sXBP-1 spliced X-box binding protein 1, CHOP C/EBP homologous protein, TUNEL terminal deoxynucleotidyl transferase dUTP nick end labeling, DAPI 4′,6-diamidino-2-phenylindole. Scale bars: 50 μm. Source data are available online for this figure.

 © The Author(s)

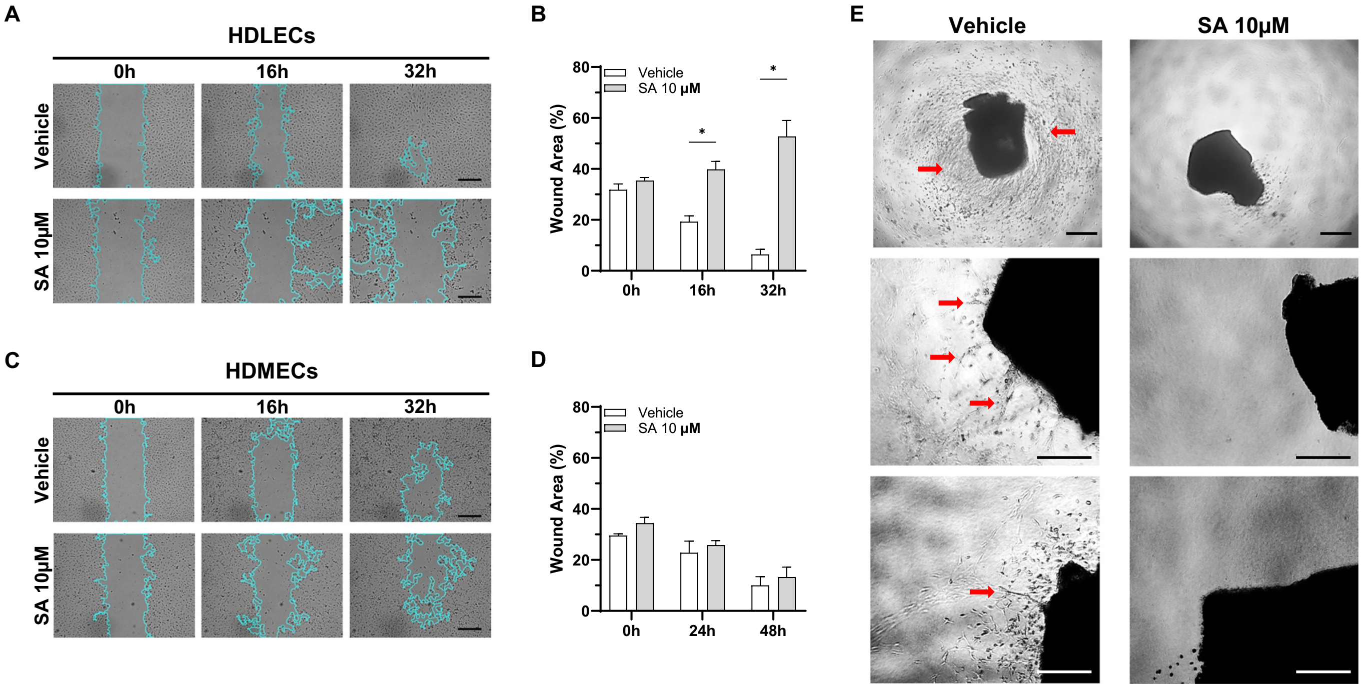

Figure EV3. Stearic acid impairs lymphatic endothelial migration and vessel sprouting.

(**A–D**) Scratch wound assay in human dermal lymphatic endothelial cells (HDLECs) and human dermal microvascular endothelial cells (HDMECs) treated with vehicle or stearic acid (SA, 10 μM). (**A**) Representative bright-field images of HDLECs at 0, 16, and 32 h post-scratch with wound edges outlined in cyan. Scale bars: 200 μm. (**B**) Quantification of wound area over time showing significantly impaired wound closure in SA-treated HDLECs ($n = 4$ independent experiments). (**C**) Representative bright-field images of HDMECs at 0, 24, and 48 h post-scratch with wound edges outlined in cyan. Scale bars: 200 μm. (**D**) Wound area quantification shows minimal effect of SA on HDMEC migration ($n = 4$ independent experiments). (**E**) Lymphatic ring assay using mouse thoracic duct segments embedded in collagen. Vehicle-treated segments show robust endothelial sprouting (red arrows), while SA-treated rings exhibit inhibition of outgrowth and vessel formation ($n = 3$ mice). Scale bars: 200 μm (top images) and 100 μm (bottom images). Data are presented as mean ± SEM. Statistical analysis: one-way ANOVA with Tukey's post hoc test. Significance: $^*P < 0.05$. Exact P values for the statistical comparisons are shown in Appendix Table S3. Source data are available online for this figure.

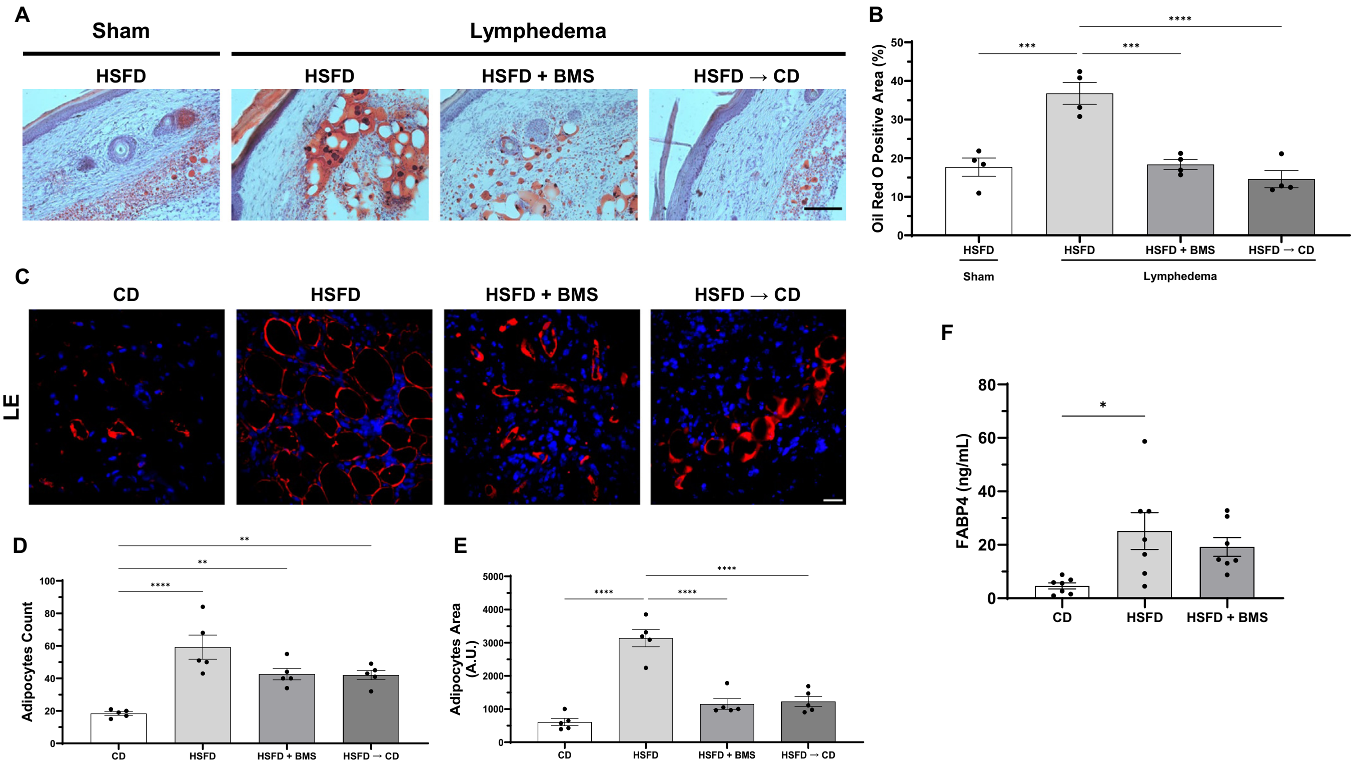

Figure EV4. FABP4 inhibition or dietary transition reduces lipid accumulation and adipocyte expansion in lymphedematous tissue.

(**A**, **B**) Oil Red O staining of tail tissue from sham and lymphedema (LE) mice maintained on a high saturated fat diet (HSFD) or treated with the FABP4 inhibitor BMS-309403 (HSFD + BMS) or switched to a control diet (HSFD → CD) after lymphatic injury. (**A**) Representative images showing lipid accumulation (red) in lymphedematous tails. Scale bars: 100 µm. (**B**) Quantification of Oil Red O positive area reveals increased lipid deposition in lymphedema tissue under HSFD, which is significantly reduced by BMS treatment or dietary transition ($n = 4$ mice). (**C**–**E**) Immunofluorescence staining for FABP4 (red) in tail tissue from CD, HSFD-, HSFD + BMS-, or HSFD → CD-fed lymphedema mice. DAPI (blue) marks nuclei. (**C**) Representative images showing increased FABP4 positive adipocytes in HSFD-fed mice. Scale bars: 50 µm. (**D**) Quantification of FABP4 positive adipocyte number. (**E**) Quantification of FABP4 positive adipocyte area ($n = 4$ mice). (**F**) Circulating FABP4 levels measured in mice with lymphedema, showing increased plasma FABP4 in HSFD-fed mice and partial reduction with BMS treatment ($n = 7$ mice). Data are presented as mean ± SEM. Statistical analysis: one-way ANOVA with Tukey's post hoc test. Significance: *$P < 0.05$, **$P < 0.01$, ***$P < 0.001$, ****$P < 0.0001$. Exact P values for the statistical comparisons are shown in Appendix Table S3. HSFD high saturated fat diet, CD control diet, FABP4 fatty acid-binding protein 4, BMS BMS-309403, DAPI 4′,6-diamidino-2-phenylindole. Source data are available online for this figure.

 © The Author(s)

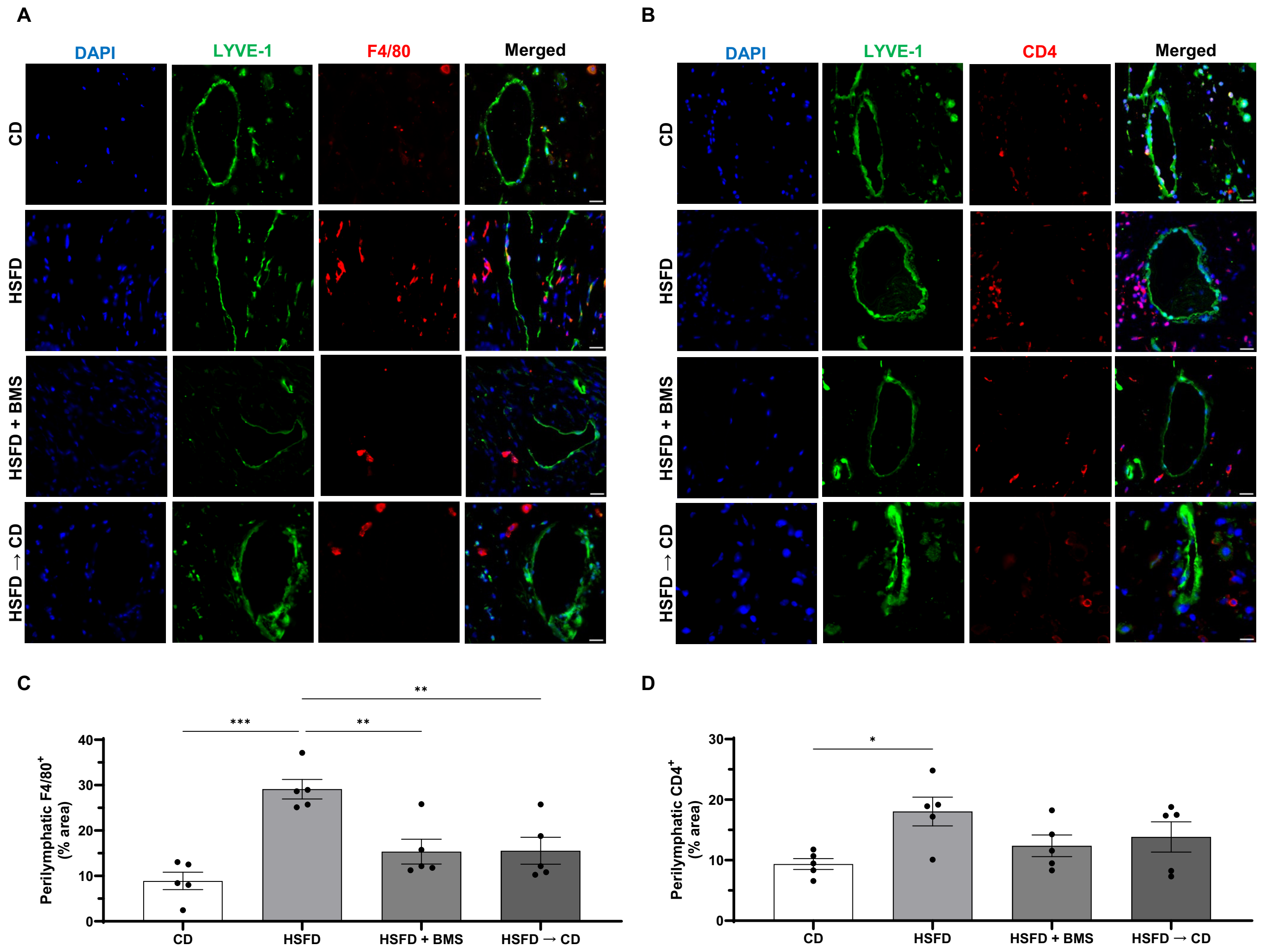

Figure EV5. FABP4 inhibition and diet transition reduce immune cell accumulation in lymphedematous tissue.

Representative immunohistochemistry images showing (**A**) F4/80 positive macrophages and (**B**) CD4 positive T cells in the perilymphatic region of tail tissue from mice with lymphedema fed a high saturated fat diet (HSFD), treated with the FABP4 inhibitor BMS-309403 (HSFD + BMS), or switched to a control diet after lymphatic injury (HSFD → CD). Scale bars: 50 μm. Quantification of immune cell infiltration for (**C**) F4/80 positive macrophages and (**D**) CD4 positive T cells ($n = 5$ mice). HSFD-fed mice exhibited significantly higher perilymphatic immune cell infiltration, which was attenuated by FABP4 inhibition or dietary transition. Data are presented as mean ± SEM. Statistical analysis: one-way ANOVA with Tukey's post hoc test. Significance: $^{*}P < 0.05$, $^{**}P < 0.01$, $^{***}P < 0.001$. Exact P values for the statistical comparisons are shown in Appendix Table S3. HSFD high saturated fat diet, CD control diet, FABP4 fatty acid-binding protein 4. Source data are available online for this figure.

© The Author(s)
